# Enabling the synthesis of multi-payload thio-antibody conjugates through the use of pyridazinediones, *p*-anisidine derivatives and various click chemistries

**DOI:** 10.1039/d6cb00018e

**Published:** 2026-02-03

**Authors:** Clíona McMahon, Christophe J. Queval, Ioanna A. Thanasi, Dawn H. W. Lau, Michael Howell, Ning Wang, Maximillian T. W. Lee, Josephine S. Gaynord, James R. Baker, Vijay Chudasama

**Affiliations:** a Department of Chemistry, University College London 20 Gordon Street London WC1H 0AJ UK; b Screening and Automated Science Laboratory, The Francis Crick Institute London NW1 1AT UK; c MSD (UK) Limited 120 Moorgate London EC2M 6UR UK v.chudasama@ucl.ac.uk

## Abstract

In recent years, antibody–drug conjugates (ADCs) have emerged as a very promising class of targeted therapeutics, but ADC candidates still face issues such as dose-limiting toxicity and drug resistance. It has become increasingly clear that whilst there are general principles for what constitutes an effective ADC, individual ADCs require bespoke optimisation. The optimal drug-to-antibody ratio (DAR) may differ for each drug, antibody and target combination. Most recently, the use of multi-drug bearing ADCs to off-set drug resistance has aroused interest. In view of this, the modular construction of antibody conjugates with different DARs/drug classes is key to enabling the next generation of ADCs. One of the leading antibody scaffolds for making ADCs is antibodies with engineered cysteine residues (thio-antibodies). Typically, it is only the engineered site that is reacted when making ADCs from thio-antibodies, inherently limiting the potential of such conjugates. This work focuses on the development of a platform for the modular and site-selective synthesis of thio-trastuzumab mutant conjugates with defined payload-to-antibody ratios (PARs) of 1, 2, 3, 4, 5, 6, 7 and 8 by modifying engineered and native cysteines (note: in this manuscript the term payload refers to fluorophores or other functional small-molecule entities attached to an antibody). The framework to achieve this is based on using only three key molecules: a diBr-pyridazinedione (PD) bearing a strained alkyne, an azide-functionalised bisPD and a functionalised azido-aniline entity. This approach, combined with the use of clickable payloads, enables the synthesis of the full repertoire of PARs 1–8, and the ability to attach up to three different payloads, in different ratios, to thio-trastuzumab mutants. The distinct PARs and ratios of payloads can be tuned by changing the reagents used and/or the order of reagents used in combination with various clickable payload reagents. Finally, various tri-payload loaded thio-antibody conjugates were appraised *in vitro*, and were shown to bind and internalise selectively to HER2^+^ (BT-474) over HER2^−^ (MCF-7) cells with all payloads successfully delivered into target cells.

## Abstract 2

A strategy for constructing multi-payload thio-antibody conjugates using pyridazinedione scaffolds, *para*-anisidine derivatives, and various click chemistries.

## Graphical abstract

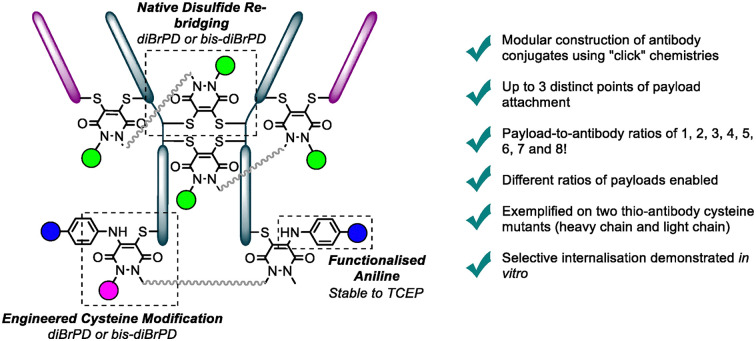

## Introduction

Over the past few decades, antibody–drug conjugates (ADCs) have emerged as a leading class of targeted therapeutics, with 15 ADCs receiving FDA approval over the past 15 years.^1,2^ Despite considerable success, clinical trials have a high failure rate, with ADC candidates being hampered by issues such as off target-toxicity^3^ and drug resistance.^4^ Whilst several new toxins/antibodies/linkers have been introduced in the clinic, conjugation methods lag behind – all FDA approved ADCs use either classical lysine/cysteine conjugation (using NHS esters or maleimides, respectively).^5–7^ These methods are frequently associated with heterogeneity as they indiscriminately target free lysine/cysteine residues.^8^ Homogeneous ADCs, and antibody conjugates more generally, have been widely accepted to display superior properties to their heterogeneous analogues, in terms of efficacy and/or safety.^9^ Considerable research has gone into developing site-selective conjugation methods over the past few years.^10^ Furthermore, it is becoming increasingly clear that whilst there are general principles for what constitutes an effective ADC, individual ADCs require bespoke optimisation. The optimal drug-to-antibody ratio (DAR) may differ for each drug, antibody and target combination. More potent, hydrophobic drugs may benefit from low DARs, whilst less potent drugs may be better suited to high DARs.^11,12^ Most recently, the use of multi-drug bearing ADCs to off-set drug resistance has aroused interest.^13^ In view of this, the modular construction of ADCs with different DARs/drug classes is highly desirable. A stepping stone to achieving this ambitious endeavour would be the plug-and-play construction of antibody-conjugates with distinct payload classes (note: in this manuscript the term payload refers to small-molecule entities such as fluorophores or other functional modules).

**Fig. 1:**
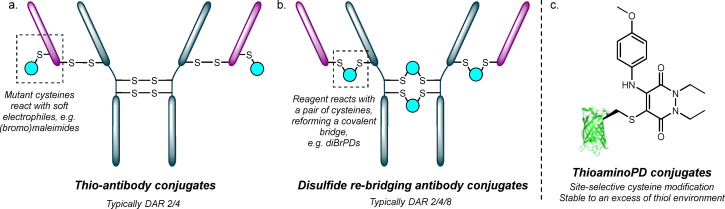
Site-selective antibody conjugates can be synthesised using (a) engineered thio-antibodies or on (b) native antibodies using disulfide re-bridging (amongst other methods); (c) thioaminoPD conjugates (in this case derived from site-selective cysteine modification of GFP S147C and *para*-anisidine) are stable to an excess of thiol environment.^45^

Strategies for site-selective antibody modification tend to be devised for use in either native or engineered antibodies.^8^ That being said, there is no reason why compatible strategies that are used in native antibodies should not also work on engineered antibodies. This includes strategies based on chemoenzymatic ligation of native antibodies (*e.g.* through the use of (engineered) microbial transglutaminase (mTG)^14,15^ and glycan remodelling^16^). Engineered antibodies typically have a unique reactive handle/sequence inserted into the protein which enables site-specific chemical conjugation.^8^ Examples include aldehyde tags^17^ (*e.g.* SMARTag^18^), SNAP-tags,^19^ π-Clamp,^20^ sortase-based methods,^21,22^ GALaXy,^23^ and the use of unnatural amino acids.^24^ Most relevant to this work are antibodies that have been engineered to include reactive cysteines (*e.g.*, THIOMABs^TM^ and other engineered cysteine-containing antibodies, hereafter referred to as thio-antibodies).^25^ Thio-antibodies were originally designed to react selectively with maleimides to form DAR 2 conjugates (Fig. 1a).^25^ Thio-antibody–drug conjugates have been shown to be safer and more efficacious than their non-site specifically conjugated analogues and there are now thio-antibody–drug conjugates in clinical/pre-clinical development.^26–28^ Whilst thio-antibodies offer highly homogenous conjugates, odd-numbered drug loadings are often unobtainable due to the symmetry of antibodies – IgGs are composed of two identical dimers; thus it is not possible to engineer an antibody such that it contains a mutation on only one polypeptide chain. Furthermore, it is difficult to make multi-drug loaded thio-antibodies without further engineering/employing highly complex linkers.^25,29,30^ Site-specific conjugation methods that are compatible with native antibodies include disulfide re-bridging (Fig. 1b),^10^ glycan re-modelling,^31,32^ and traceless affinity peptide labelling.^33,34^ Disulfide re-bridging attempts to alleviate issues associated with conventional cysteine modification of native antibodies (heterogeneity, retro-Michael deconjugation with commonly used maleimides, breaking of disulfide linkages *etc.*).^10^ Reagents developed for disulfide re-bridging include dibromomaleimides,^35^ bissulfone reagents (ThioBridge^TM^),^10,36^ and divinylpyrimidines.^37^ One of the most widely explored class of reagents for this purpose in recent years is dibromopyridazinediones (diBrPDs),^38,39^ with diBrPDs and/or bis(diBr)PDs being used to form (near) homogenous DAR 2/4/8 ADCs.^40–42^ Other DARs, using disulfide re-bridging, have been achieved through other strategies, for example, Dannheim *et al.* used tetra-divinylpyrimidine linkers to make DAR 1 conjugates from a native antibody scaffold.^43^ We also note that DAR 1 conjugates derived from the chemoenzymatic GlycoConnect modification of a native antibody have also been furnished and shown to be highly effective in tumour spheroids.^44^

Whilst work has been carried out to make ADCs with various DARs and/or multi-drug loaded ADCs,^29,46–48^ as well as the complementary work of Journeaux *et al.* on forming quadruple-functionalised antibodies albeit not in the form of ADCs,^29^ very limited work has been carried on enabling such features for the leading class of engineered antibodies for ADCs, *i.e.* thio-antibodies. In almost all cases, it is only the engineered site that is reacted when making ADCs from thio-antibodies, inherently limiting the potential of such conjugates. We theorised that diBrPDs should react with thio-antibodies as (i) they have been shown to react with single cysteine mutants^45^ and (ii) they are structurally similar to bromomaleimides that have been shown to form stable, near-homogenous thio-antibody ADCs.^49^ Moreover, post-conjugation, if the remaining bromine on the pyridazinedione ring could be substituted with an aniline-like molecule, the thioaminoPD small-molecule should be deactivated and stable to TCEP as they have been shown to be stable in a reducing environment (Fig. 1c).^45^ This would enable the native interchain disulfide bonds to be subsequently reduced and then undergo disulfide re-bridging without affecting the previously conjugated mutant cysteines. Hence, by combining these distinct technologies (engineered cysteine modification, native disulfide re-bridging and aniline conjugate addition) the formation of engineered cysteine containing antibody-conjugates with discrete, controlled (multi-)payload loadings in a site-selective, modular manner would be feasible.

In this manuscript, we demonstrate that by using three click handle-containing molecules – a diBrPD bearing a strained alkyne (BCN PD 1), a bisPD containing an azide (ArN_3_ bisPD 2) and an azide functionalised aniline (N_3_ aniline 3) – in combination with clickable payloads, access to a wide range of payload loadings (payload-to-antibody ratio (PAR) from 1 through to 8) is realised. Moreover, by changing the order in which the reactions are carried out/which combination of molecules are used, we show that up to three different entities can be attached site-selectively and in a modular manner. We demonstrate by ELISA that thio-antibody conjugates retained binding affinity for their biological target HER2 (relevant in breast/stomach cancers). Finally, we show that the tri-functional conjugates bind and internalise selectively to HER2^+^ over HER2^−^ cell lines with all three payloads delivered into target cells only. It is acknowledged that the conjugation of toxin payloads can be different compared to the model payloads in this manuscript (*e.g.* biotin and fluorophores), but as we are using well established “click” chemistries to append the payloads, we would anticipate efficient translation from fluorophores/biotin to small-molecule toxins.

## Results and discussion

**Fig. 2:**
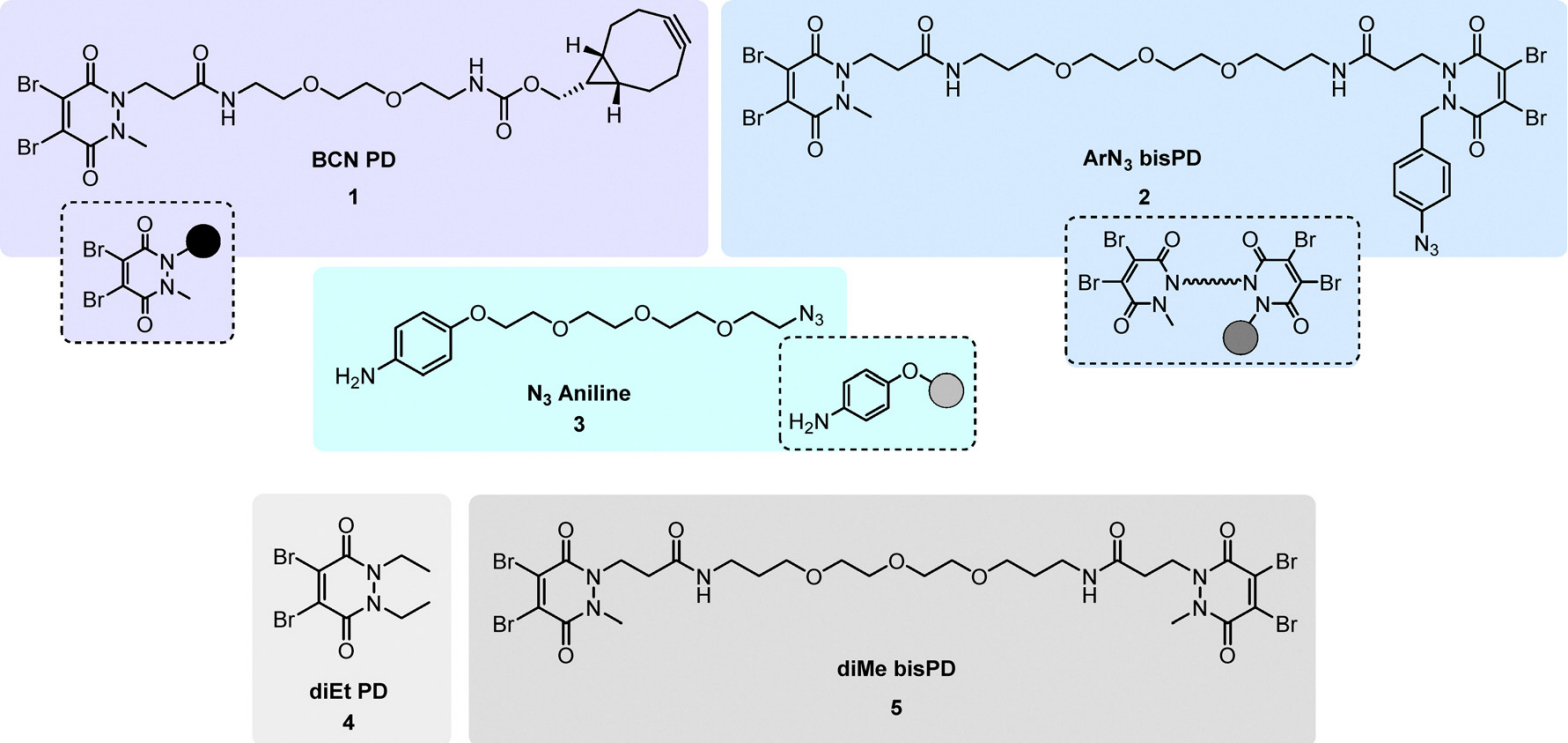
Click-handle containing BCN PD 1 (5 step synthesis from 1-boc-1-methylhydrazine; this compound is also available commercially from various suppliers (*e.g.* Merck and Precise PEG)), ArN3 bisPD 2 (8 step synthesis from 1,2-diboc-hydrazine (di-*tert*-butyl hydrazodiformate); that said, many intermediates in the synthesis are commercially available) and N_3_ aniline 3 (5 step synthesis from *para*-aminophenol and tetraethylene glycol), and model PDs diEt PD 4 (2 step synthesis from 1,2-diboc-hydrazine (di-*tert*-butyl hydrazodiformate)) and diMe bisPD 5 (4 step synthesis from 1-boc-1-methylhydrazine; this compound is also available commercially from Precise PEG). For full synthetic schemes, see SI Fig. S1–S5. Note: many synthetic steps are common in the synthesis of the various PDs.

Our study began with the synthesis of the three aforementioned molecules 1–3 (Fig. 2). Firstly, a bicyclo[6.1.0]non-4-yne (BCN)-containing PD (BCN PD) 1 was synthesised by adapting a protocol described in our previous work (see SI Fig. S1 for full details).^50^ Next, aryl–azide/methyl bisPD (ArN_3_ bisPD) 2 was synthesised by coupling a diBrPD methyl/acid and diBrPD aryl–azide/acid together *via* a bis-amine-PEG_3_ entity (see SI Fig. S2 for full details). For the functionalised aniline, 4-(2-(2-(2-(2-azidoethoxy)ethoxy)ethoxy)ethoxy)aniline (N_3_ aniline 3), a procedure reported in our previous work was adapted for its synthesis (see SI Fig. S3 for full details).^45^ An unfunctionalised diBrPD, dibromo diethyl pyridazinedione (diEt PD) 4, and bisPD, dibromo dimethyl bis-pyridazinedione (diMe bisPD) 5 were also synthesised (in an analogous manner to BCN PD 1/ArN_3_ bisPD 2, respectively, see SI Fig. S4 and S5 for full details), to act as model PDs where necessary (Fig. 2).

**Fig. 3:**
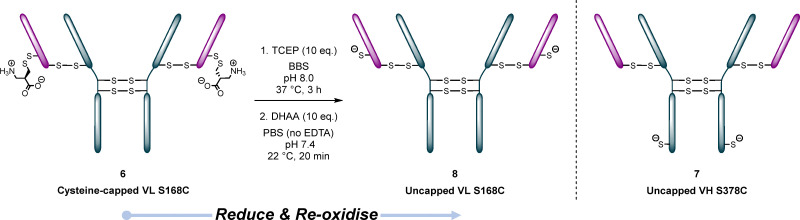
Uncapping of cysteine-capped LC S168C 6 to afford LC S168C 8, and HC S378C 7.

With these molecules in hand, we set about obtaining suitable thio-antibody mutants to appraise our methodology. We obtained two different thio-trastuzumab mutants (from MSD UK): cysteine-capped LC S168C 6, and HC S378C 7. The mutations were on different parts of the antibody as we were especially interested in elucidating any differing reactivity between the two types of mutant. Whilst the engineered cysteine on HC S378C 7 was available for reaction, the LC S168C mutant cysteines were capped with cysteine (calculated as the mass difference between the capped and uncapped form was ∼240 Da); this is common with thio-antibodies as a result of their synthesis.^25^ As such, before the LC S168C 8 mutant could be used, LC S168C 6 had to be “uncapped” whilst retaining the native interstrand disulfides in two-step reduction–oxidation procedure to afford the conjugate (Fig. 3, see SI Tables S6–S8 and Fig. S8–S31 for details on optimisation). The ability to successfully conjugate the engineered cysteines of uncapped LC S168C 8 and HC S378C 7 was confirmed by reaction with *N*-methylmaleimide (see SI conjugates S14 and S15 (respectively) for details).

### PARs 2, 4 and 2 + 2

**Fig. 4:**
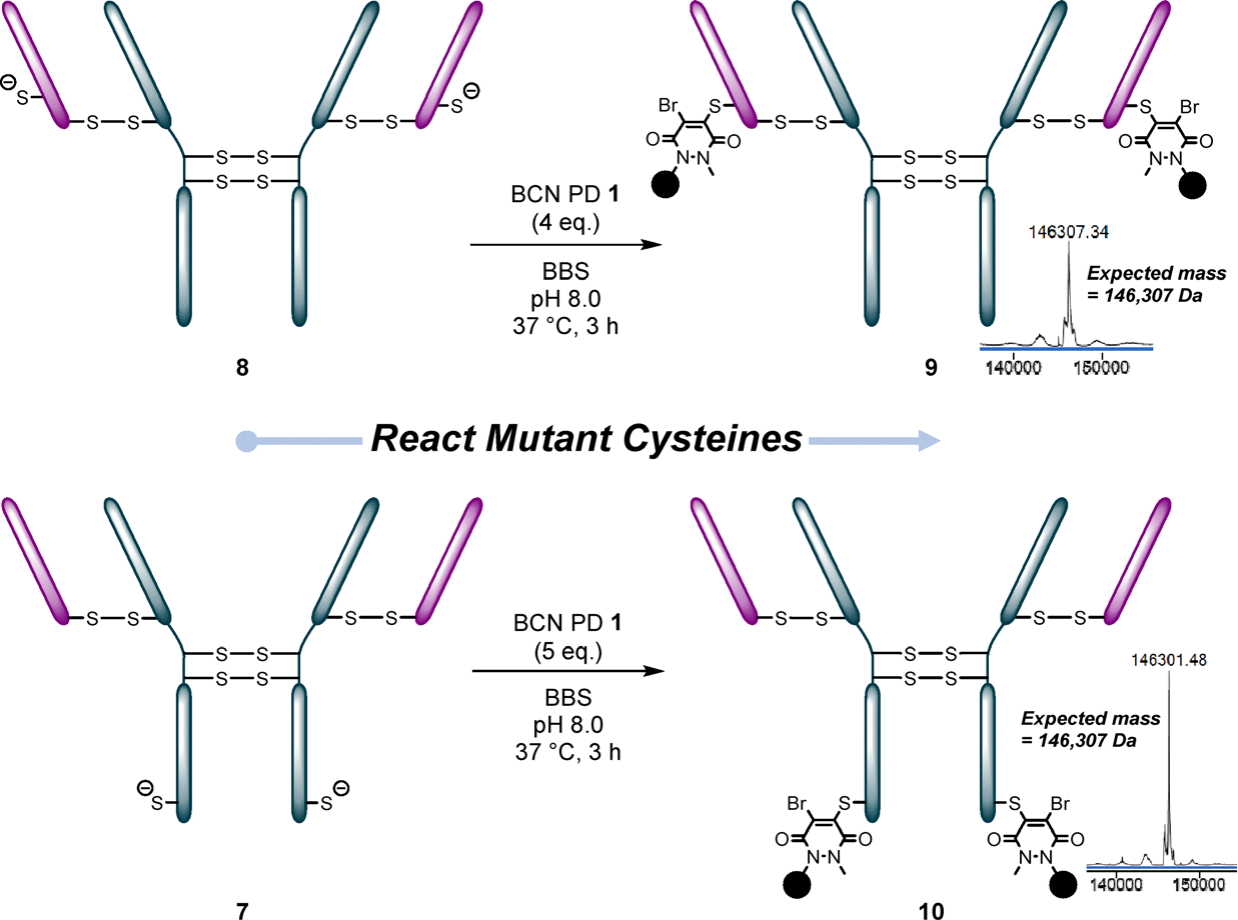
Reaction of the thio-trastzumab mutants LC S168C 8 and HC S378C 7 with BCN PD 1.

**Fig. 5:**
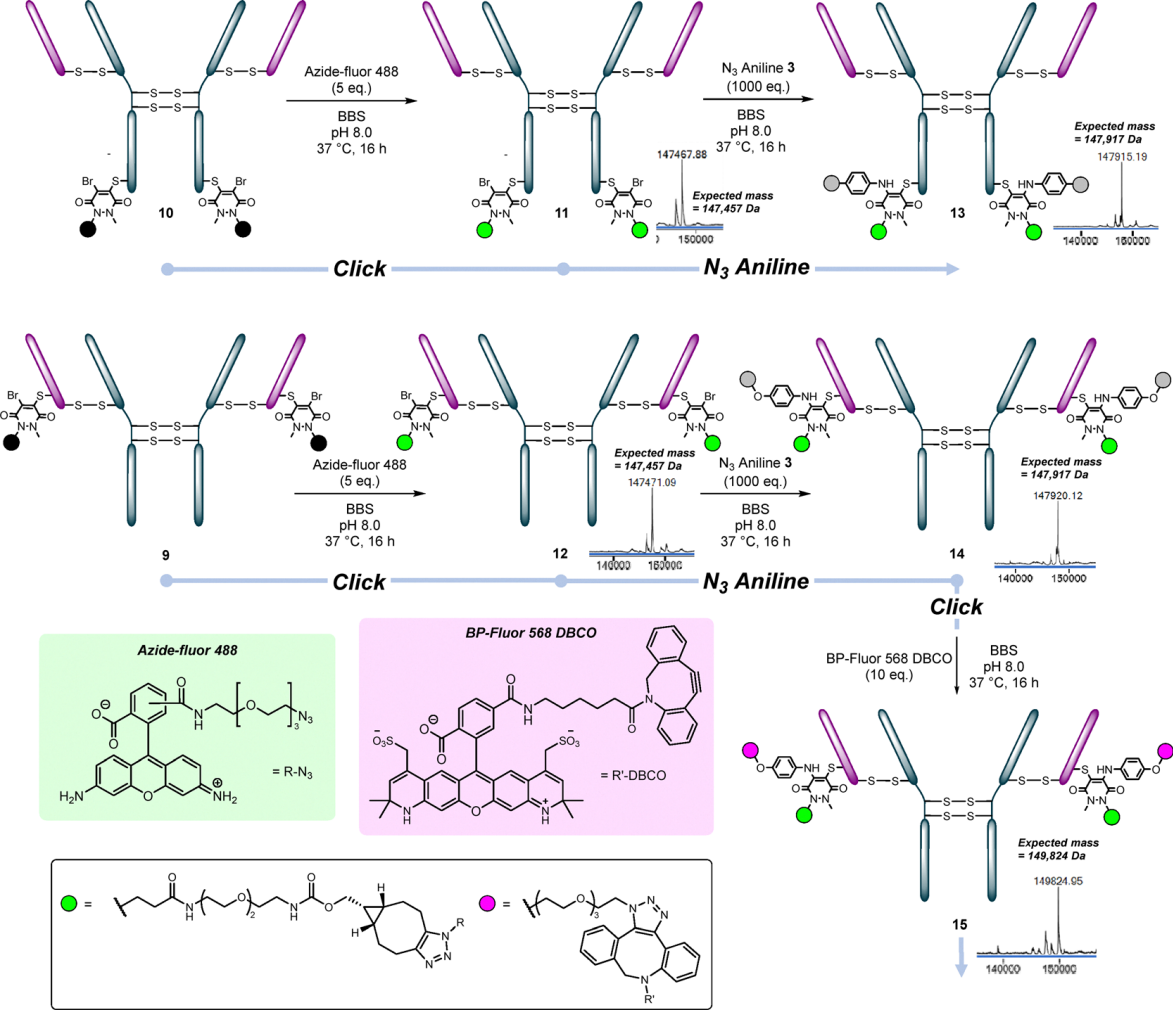
The reactions of conjugates 9 (LC S168C conjugated to BCN PD 1) and 10 (HC S378C conjugated to BCN PD 1) with Azide-fluor 488 and subsequent reactions with N_3_ aniline 3. Deconvoluted, zoomed-in mass spectra are shown beside conjugates.

Uncapped thio-trastuzumab mutants LC S168C 8 and HC S378C 7 were then reacted with BCN PD 1 (Fig. 4). After some minor optimisation for reactions with HC S378C 7 (see SI Table S9 and Fig. S34–S38 for details), conjugates 9 and 10 were formed in excellent conversion yields. However, the main peak on LC-MS for the product was split in two, with the secondary peak −35 Da off the expected mass. We observed this splitting intermittently on both mutants. We believed it to be an artefact related to the LC-MS conditions, as the splitting always disappeared following displacement of the bromines with an aniline-like molecule (details below, Fig. 5).

Both conjugates 9 and 10 were clicked with Azide-Fluor 488 to give HC S378C-Azide Fluor 488 11 and LC S168C-Azide Fluor 488 12 (respectively). We then showed that the free bromines could be displaced by N_3_ aniline 3, affording conjugates 13 and 14, respectively. These conjugates could subsequently be clicked again using strained alkyne bearing payloads, to give either PAR 4 (if the same payload was clicked on) or 2 + 2 (if a different payload was clicked on) conjugates. This was exemplified using BP-Fluor 568 for the LC S168C mutant, giving conjugate 15 (Fig. 5).

### PARs 1, 3 and 1 + 2

As BCN PD 1 reacted well with the thio-trastuzumab mutants, it was theorised that bisDiBrPDs could react across the engineered cysteines if the linker between the PDs was long enough/the two mutant cysteines were sufficiently close in space. If successful, this would be a simple method of synthesising thio-antibody conjugates with the unusual DARs of 1 and 3 if the bisDiBrPD had one click handle and anilines bearing a click handle could react with the thioBrPDs.

The distance between the reactive sites on ArN_3_ bisPD 2 was measured to be *ca.* 22.5 Å by MolView. The distance between the HC mutant cysteines on HC S378C 7 was measured to be *ca.* 22.5 Å by PyMol (using PDB: 1HZH (human IgG1 b12) as there is no available crystal structure for full trastuzumab, see SI Fig. S50 for details), making the HC S378C mutant an excellent candidate for this reaction. On the other hand, the distance between the LC mutant cysteines was measured to be *ca.* 87.1 Å by PyMol (using PDB: 1HZH (human IgG1 b12) as there is no available crystal structure for full trastuzumab, see SI Fig. S50 for details), so we judged that it was unlikely that the desired PAR 1 product would be obtained when reacting ArN_3_ bisPD 2 with LC S168C 8. That all being said, it was appreciated that antibodies are flexible and the distances in space may in fact be different when dynamic antibodies are in solution.

Reaction of model diMe bisPD 5 with LC S168C 8 and HC S378C 7 gave results that were somewhat consistent with expectations. Reaction of LC S168C 8 with diMe bisPD 5 resulted in the formation of an appreciable amount of one bis PD per mutant cysteine – likely formed due to a lack of favourability of the bridging the bisPD across the mutant cysteines (see SI Fig. S64 and S65 for details). We hypothesise that by using a bisPD with a longer PEG chain connecting the two PDs, re-bridging across the mutant cysteine could be attainable, but this is outside the scope of this study.

**Fig. 6:**
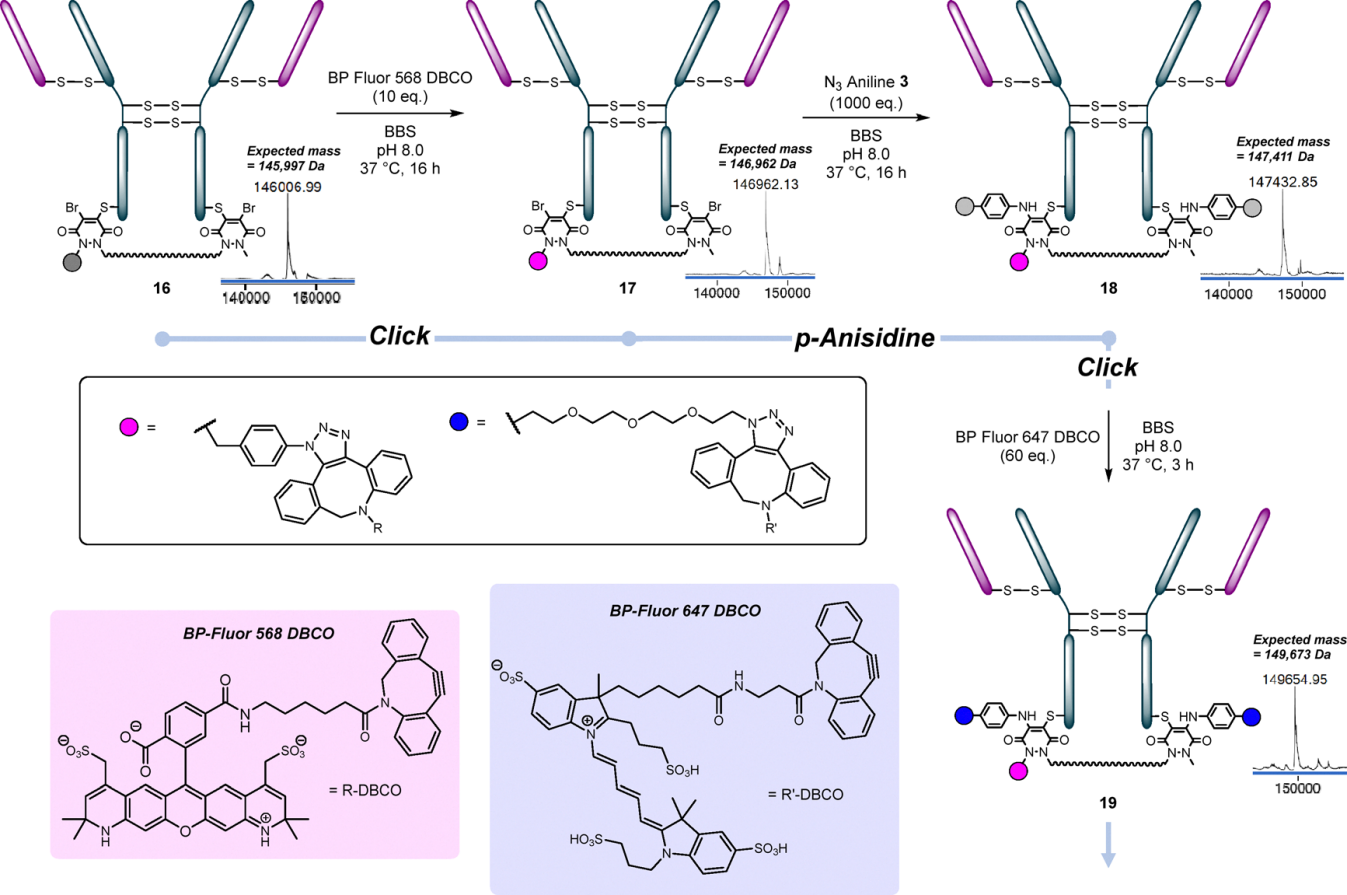
Reactions of HC S378C 7 with ArN_3_ bisPD 2 followed by reaction with various DBCO fluorophores and N_3_ aniline 3.

In contrast to the light chain mutant, reaction of diMe bisPD 5 (5 eq.) with HC S378C 7 at 37 °C for 3 h in BBS (pH 8) afforded the desired conjugate S16 (see SI Fig. S49 for details) in excellent conversion. Moreover, the subsequent reaction of this conjugate 7 with *p*-anisidine (1000 eq.) at 37 °C for 16 h in BBS (pH 8) also worked well in excellent conversion (see SI Fig. S51 and S52 for details). Pleasingly, ArN_3_ bisPD 2 reacted with HC S378C mutant 7 in a similar manner to diMe bisPD 5 under analogous conditions, giving conjugate 16 in excellent conversion. Subsequently clicking the azide of conjugate 16 with BP-Fluor 568 DBCO afforded desired homogenous PAR 1 conjugate 17. Displacement of the bromines of the thioBrPDs with N_3_ aniline 3 (conjugate 18) and subsequent clicking of the two azides with BP Fluor 647 DBCO (to form conjugate 19) provided access to a PAR 1 + 2 (or 3) conjugate (Fig. 6). Alternatively, conjugate 16 could be clicked with DBCO biotin, then the bromines displaced with N_3_ aniline 3, and the two azides subsequently clicked to BP Fluor 568 DBCO to form conjugate 20 (Fig. 11). As the two heavy chain mutant cysteines are relatively close in space, and potentially quite sterically hindered (especially as they had already been clicked), a large excess of BP Fluor 647 DBCO (to form conjugate 19) and BP Fluor 568 DBCO (to form conjugate 20) were used to ensure full conversion.

### Disulfide re-bridging of trastuzumab

As many of the further proposed thio-antibody conjugates rely on the ability of PDs to successfully re-bridge the native disulfide bonds of trastuzumab, we wanted to ensure we had a robust protocol for re-bridging trastuzumab with both BCN PD 1 and ArN_3_ bisPD 2 before attempting further work on the thio-antibodies. We trialled a variety of different conditions on native trastuzumab (Ontruzant®), see SI Table S10 and Fig. S66–S86, S89 and S90. Our optimised conditions for re-bridging with both BCN PD 1 or the ArN_3_ bisPD 2 were as follows: the antibody (20 µM in BBS, pH 8.0) was reduced with TCEP (10 eq., 37 °C) for 1.5 h. After this, TCEP was removed and the reduced antibody reacted with (bis)PD (10 eq., 37 °C) for 3 h. This protocol consistently gave us a near homogenous conjugate with a PD-to-antibody ratio (PDAR 4) for BCN PD; this could be then clicked with Azide-fluor 488 with full conversion (fluorophore-to-antibody ratio (FAR) = 4.0). The same protocol resulted in a clean PDAR 2 when using the ArN_3_ bisPD 2. This conjugate could be clicked too (exemplified with DBCO-biotin). In both cases, the corresponding half antibody conjugate species were also present as the minor product; this does not affect the PDARs. We also note that the formation of analogous half antibody conjugate species, owing to some non-native PD rebridging in the hinge region when reacting the reduced native disulfide bonds of thio-antibodies (or conjugates) thereof with BCN PD 1 and/or ArN_3_ bisPD 2 (in all relevant experiments described below), is observed – this will be denoted by a dagger symbol (†) for each relevant figure.

### Concurrent reaction of the disulfides and mutant cysteines of thio-antibodies: PARs 6, 8 and 6 + 2

**Fig. 7:**
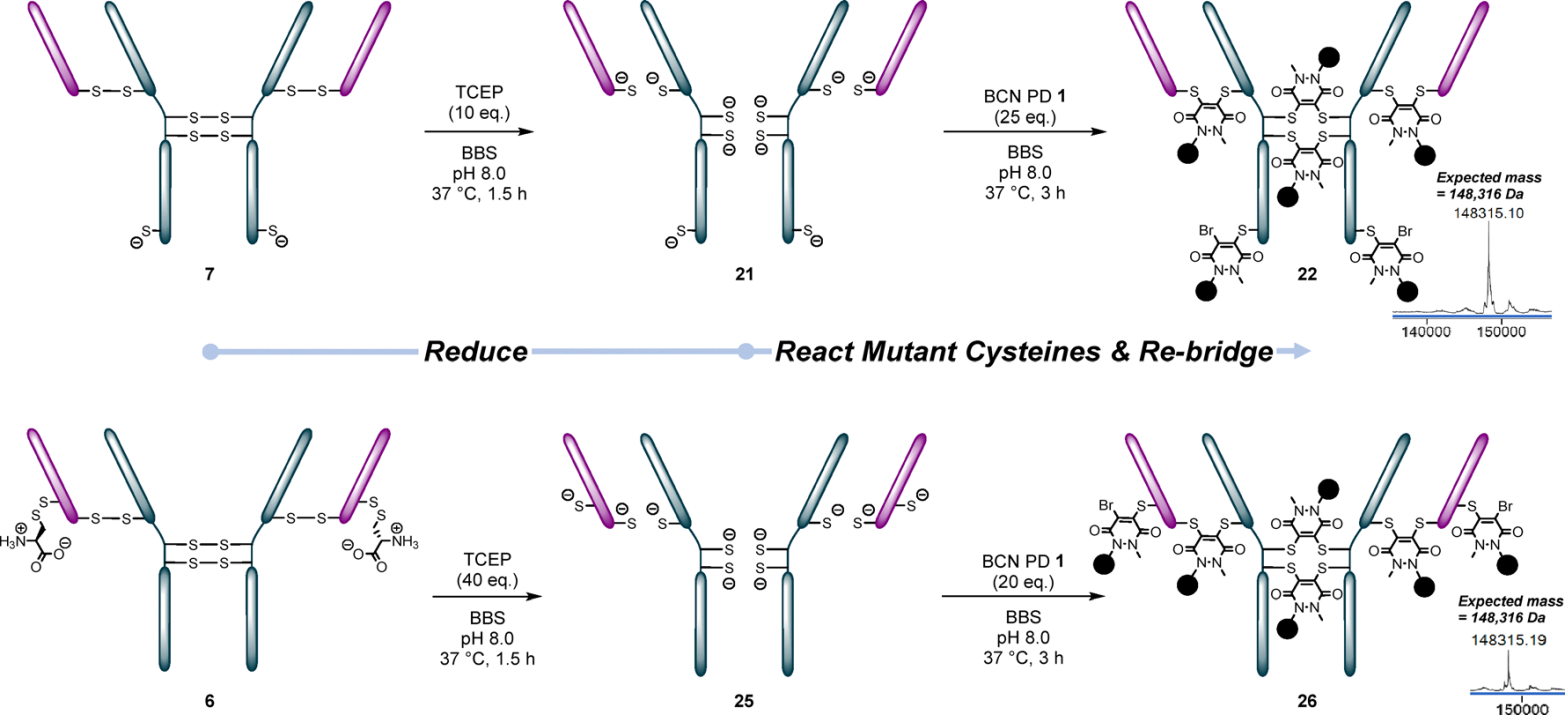
One-pot reaction of reduced thio-trastuzumab mutants 7 and 6 with BCN PD 1 to afford conjugates 22 and 26.^†^

In general, when uncapping thio-antibodies, reducing the interchain disulfides is generally unavoidable, so a re-oxidation step is needed (see Fig. 3). This extra step is usually undesirable, and we hypothesised that if a diBrPD was added straight after reduction of a thio-antibody (capped or uncapped) that disulfide re-bridging as well as conjugation to the mutant cysteines would occur (Fig. 7). For this to occur, the mutant cysteines would have to be sufficiently far from the interchain disulfides so that one PD could not react with a mutant cysteine and one liberated from a reduced interchain disulfide bond.

**Fig. 8:**
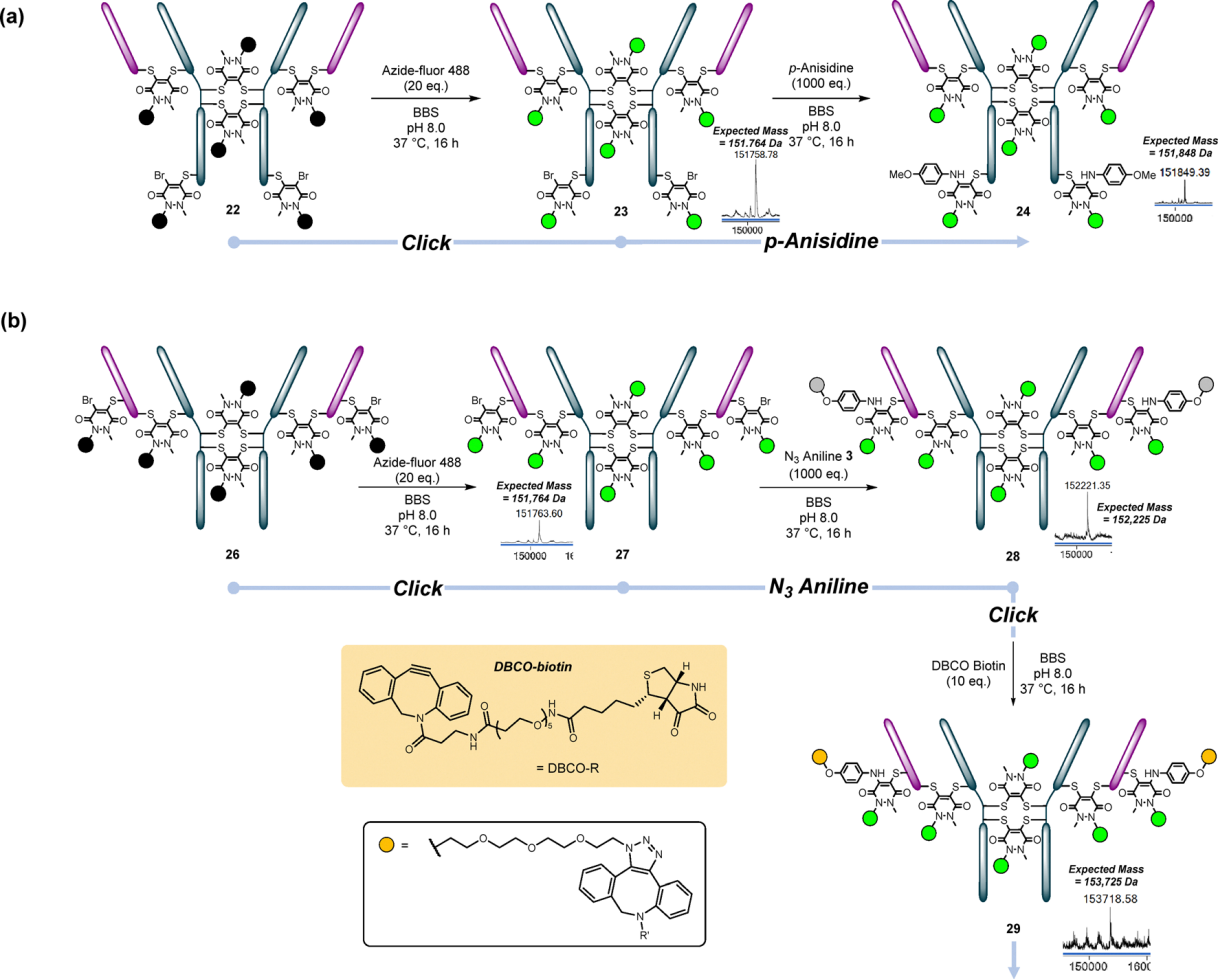
(a) Reactions of conjugate 22 (HC S378C reacted/re-bridged with BCN PD 1) with *p*-anisidine/N_3_ aniline 3 and Azide-fluor 488. (b) Reactions of conjugate 26 (LC S168C reacted/re-bridged with BCN PD 1) with *p*-anisidine/N_3_ aniline 3 and Azide-fluor 488.^†^

This was first attempted on the HC S378C mutant 7. Although already uncapped, this would show us whether it was possible for the PDs to differentially react with the mutant cysteines and the disulfides. The mutant was reduced using 10 eq. TCEP, the excess TCEP then removed by ultrafiltration and the reduced antibody (conjugate 21) then subsequently reacted with 25 eq. BCN PD 1 to afford the desired PDAR 6 conjugate 22. This conjugate could then be clicked with Azide-fluor 488 and then reacted with *p*-anisidine to create conjugates 23 and 24, providing proof of concept for PAR 8 (6 + 2) (Fig. 8).

Next, the one-pot reaction was performed using cysteine-capped LC S168C mutant 6. This time, the mutant was uncapped and reduced using 40 eq. TCEP to ensure that the antibody was both uncapped and fully reduced. The reduced antibody 25 was subsequently reacted with 20 eq. BCN PD 1 after removal of the excess TCEP by ultrafiltration. This reaction worked well, giving 26 (PDAR 6) as the main product. A very small amount of PDAR 5 (one mutant cysteine and all four disulfides reacted) was also visible on LC-MS. This conjugate could then be clicked and reacted with N_3_ aniline 3, creating conjugates 27 and 28. N_3_ aniline conjugate 28 was clicked with DBCO-biotin to form PAR 8 conjugate 29. This gave us our proof-of-concept for PAR 6, 8 and 6 + 2 conjugates (Fig. 8).

### Disulfide re-bridging of mutant cysteine modified thio-antibody conjugates

In order to synthesise trifunctional thio-trastuzumab-PD conjugates, and to access PARs 5 and 7, the PD-conjugated mutant cysteines would need to be stable to TCEP reducing conditions, at least under the conditions required for full reduction of the interchain disulfides (10 eq. TCEP, 1.5 h, 37 °C). To test this, the HC S378C thio-trastuzumab mutant was reacted with a model PD, diEt PD 4, followed by reaction with *p*-anisidine. This thioaminoPD conjugate was then incubated with TCEP under disulfide reduction conditions. After 1.5 h, excess reagents were removed and the conjugate analysed by LC-MS. To our delight, no apparent deconjugation of the thioaminoPD linkage was observed. We also independently corroborated this reactivity profile on model protein GFP S147C (see SI Fig. S114, S116 and S118 for details of these stability tests). These results suggested that thioaminoPD-conjugates of thio-trastuzumab antibodies would be stable to TCEP reduction conditions required to reduce the native interstrand disulfide bonds of the thio-antibody. With this in mind, we moved towards attempting the proposed antibody disulfide re-bridging reactions where the interstrand disulfide bonds were to be reduced in the presence of a thioaminoPD linkage.

### Proof-of-concept for PAR 6: 2 + 4

**Fig. 9:**
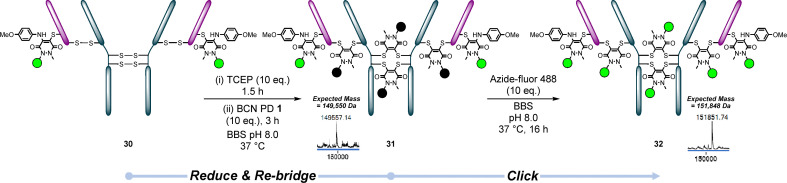
Conjugate 30 (LC S168C reacted with BCN PD 1 clicked with Azide-fluor 488 and *p*-anisidine) was re-bridged with BCN PD 1 to form conjugate 31, then clicked with Azide-fluor 488 to form conjugate 32.^†^

We began by synthesising LC S168C-BCN PD-*p*-anisidine–Azide Fluor 488 conjugate 30. The native disulfides of this conjugate were then reduced and re-bridged with BCN PD 1, using our optimised disulfide re-bridging protocol, to give re-bridged conjugate 31. Pleasingly, this worked well and the subsequent click reaction with 10 eq. Azide-fluor 488 gave a clean conjugate, 32, providing proof-of-concept of these two-step reactions, as well as for PARs 6 and 2 + 4 (Fig. 9).

### PAR 6: 2 + 2 + 2

**Fig. 10:**
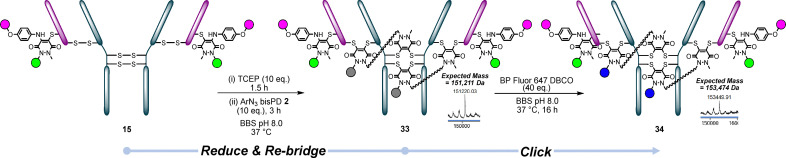
Conjugate 15 (LC S168C reacted with BCN PD clicked with Azide-fluor 488, reacted with N_3_ aniline 3 which was clicked with BP Fluor 568) was re-bridged with BCN PD 1 to form conjugate 33, which was then clicked with BP Fluor 647 to form trifunctional conjugate 34.^†^

Next, we attempted to disulfide re-bridge conjugate 15 (BCN PD-Azide-fluor 488-N_3_ aniline-BP Fluor 589-LC S168C) with ArN_3_ bisPD 2 across pairs of native disulfide bonds. Once again, this reaction proceeded smoothly, forming conjugate 33 and, following a click with BP Fluor 647, affording conjugate 34, providing access to 2 + 2 + 2 (PAR 6) (Fig. 10).

With this result in-hand, we decided to move onto appraising the reaction of ArN_3_ bisPD-HC S378C conjugates 19 and 20 with disulfide re-bridging PDs, as this would theoretically provide access to our final remaining PARs: 5 and 7.

### PAR 5: 2 + 1 + 2

**Fig. 11:**
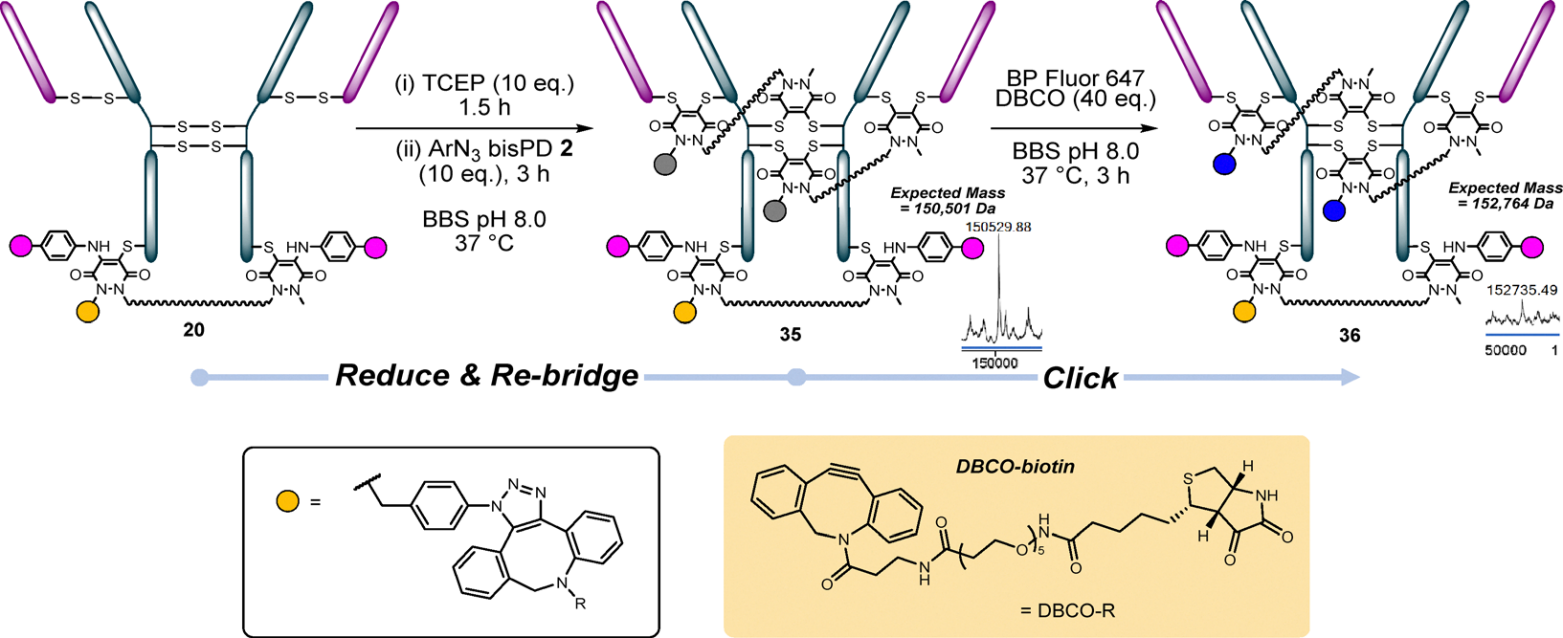
Conjugate 20 (HC S378C reacted with ArN_3_ PD 2 clicked with DBCO biotin and N_3_ aniline 3 which was clicked with BP Fluor 568) was re-bridged with ArN_3_ bis PD 2 to form conjugate 35, which was then clicked with BP Fluor 647 to form conjugate 36.^†^

To form these conjugates we followed a similar protocol as for the formation of the aforementioned PAR 8 and 6 conjugates. First, we reduced conjugate 20, then disulfide re-bridged it with ArN_3_ bisPD 2 to form conjugate 35. This was successfully clicked with BP Fluor 647 to form conjugate 36, thus providing access to 2 + 1 + 2 (PAR 5) (Fig. 11).

### PAR 7: 2 + 1 + 4

**Fig. 12:**
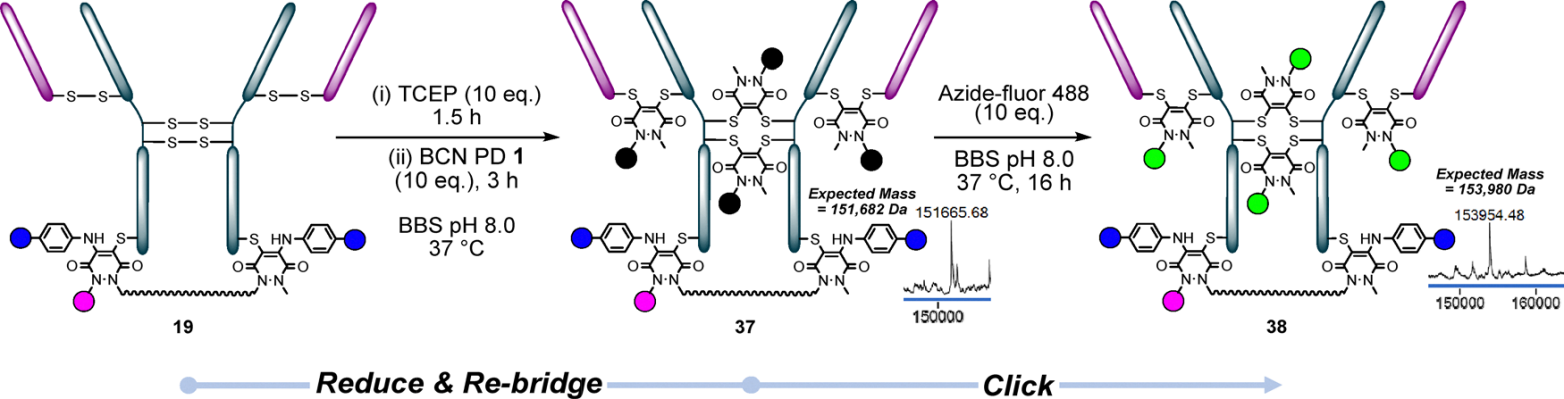
Conjugate 19 (HC S378C reacted with ArN_3_ PD 2 clicked with BP Fluor 568, reacted with N_3_ aniline 3 which was clicked with BP Fluor 647) was re-bridged with BCN PD 1 to form conjugate 37, which was then clicked with Azide-fluor 488 to form trifunctional conjugate 38.

To form our final unmet PAR conjugate target, we reduced conjugate 19 (ArN_3_ bisPD-BP Fluor 568-N_3_ aniline-BP Fluor 647 HC S378C) and then re-bridged the reduced disulfides with BCN PD 1, to form conjugate 37. Finally, we clicked this with Azide-Fluor 488 to form trifunctional conjugate 38, thus providing access to 2 + 1 + 4 (PAR 7) (Fig. 12).

### ELISAs and internalisation studies

Having formed thio-antibody conjugates with PARs 1–8 in a modular and site-selective manner, we next evaluated if our conjugation strategies had compromised antibody–antigen binding. To appraise this, we performed ELISA analysis on PDAR 6 LC S168C conjugate S34 (see SI conjugate S34 for synthesis and characterisation) and HC S378C conjugate 16 using HER2 antigen with the unmodified thio-trastuzumab antibodies acting as controls. These conjugates were chosen as they were representative of the range of conjugates synthesised – one light chain conjugate and one heavy chain conjugate, one with a bisPD, one with all disulfides re-bridged and with six modules attached. Gratifyingly, we found no significant difference in HER2 binding between our conjugates and the native thio-trastuzumab controls (see ELISA section in the SI for details).

Finally, we performed internalisation studies on selected conjugates. The conjugates chosen for this were trifunctional LC S168C conjugate 34 and HC S378C conjugate 38. Trifunctional conjugates were chosen for these assays as visualising the three different fluorophores would allow us to determine if all three modules were internalised into targeted cells treated with the conjugates. The three different fluorophores – Azide-fluor 488, BP Fluor 568 DBCO and BP Fluor 647 DBCO were chosen as they each fluoresce under a different excitation/emission wavelength and could be independently captured by fluorescence microscopy.

**Fig. 13:**
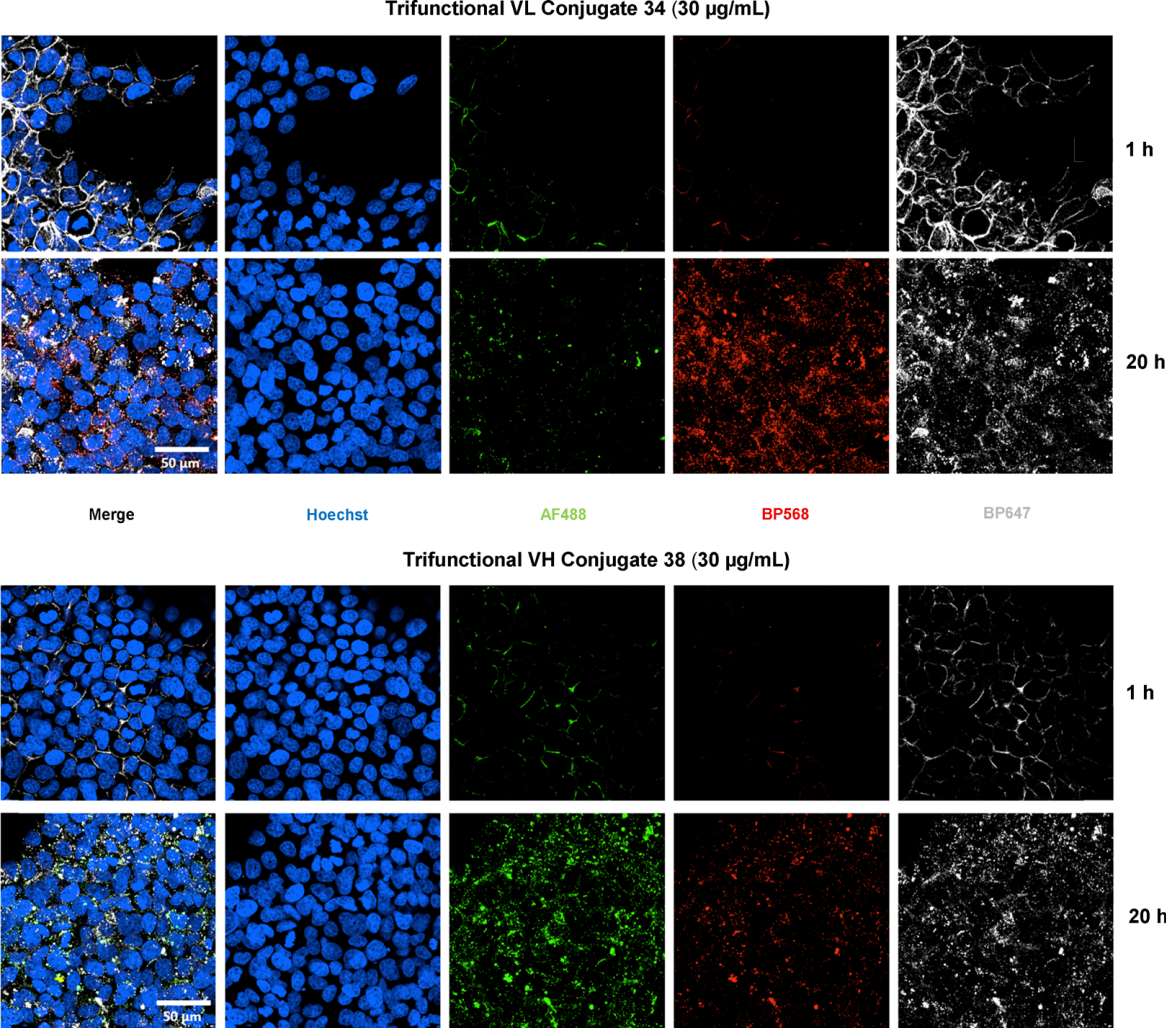
High magnification (63×) images of HER2^+^ (BT-474) cells 1 h and 20 h post treatment with LC S168C conjugate 34 and HC S378C conjugate 38 at a concentration of 30 µg mL^−1^. The three fluorophores are visualised as follows: Dapi (nuclei) in blue; Azide-fluor 488 in green; BP Fluor 568 in red and BP Fluor 647 in white-grey.

For these studies, BT-474 was used as the HER2^+^ cell line, and MCF-7 as the HER2^−^ control cell line. To test binding/internalisation, LC S168C conjugate 34 and HC S378C conjugate 38 were added and the cells incubated for 1 h at 4 °C to allow binding but preventing endocytosis. The cells were then washed with PBS to remove unbound antibody conjugates, and then incubated in fresh medium at 37 °C. As expected, conjugates 34 and 38 were bound and internalised only to the HER2^+^ cells. Interestingly, the HC conjugate showed lower fluorescence at the same dose. After 1 h at 4 °C some cells were fixed, and this showed that the conjugates were clustered around the cell membrane of HER2^+^ (BT-474) cells (Fig. 13). After switching to 37 °C, and fixing the cells at 20 h, the fluorescence appeared to be mostly concentrated in dots within the cells, suggesting internalisation into cellular compartments (Fig. 13). No binding or internalisation was observed in the negative control MCF-7 cells (see SI Fig. S143 for details).

In both cases, the BP Fluor 647 gave a much brighter signal than the Azide-fluor 488 or the BP Fluor 568. Indeed, the extinction coefficient of BP Fluor 647 is much higher than the extinction coefficient of the other two fluorophores, which could explain this difference of intensity. Despite this difference, a signal from all three fluorophores was detected. The three fluorescent probes were initially detected around the cell membranes but over time became concentrated within intracellular vacuoles. Most importantly, all three fluorophores are detectable within the cell after 20 h of treatment, and presented a similar dotted-distribution within the cytoplasm (Fig. 13).

This study clearly shows that for both trifunctional thio-antibody conjugates, the three fluorophores traffic over time from the cellular membrane to the cytosol and persist at least up to 20 h post-treatment. The co-distribution of each fluorophore present within the intracellular vacuoles after 20 h of treatment was quantified (see SI Fig. S144 for details). It was not only confirmed that the fluorescence of each fluorophore was still detectable, but also shown that some combinations of fluorophore still co-exist in close vicinity, likely sharing the same vacuolar space.

## Conclusions

**Fig. 14:**
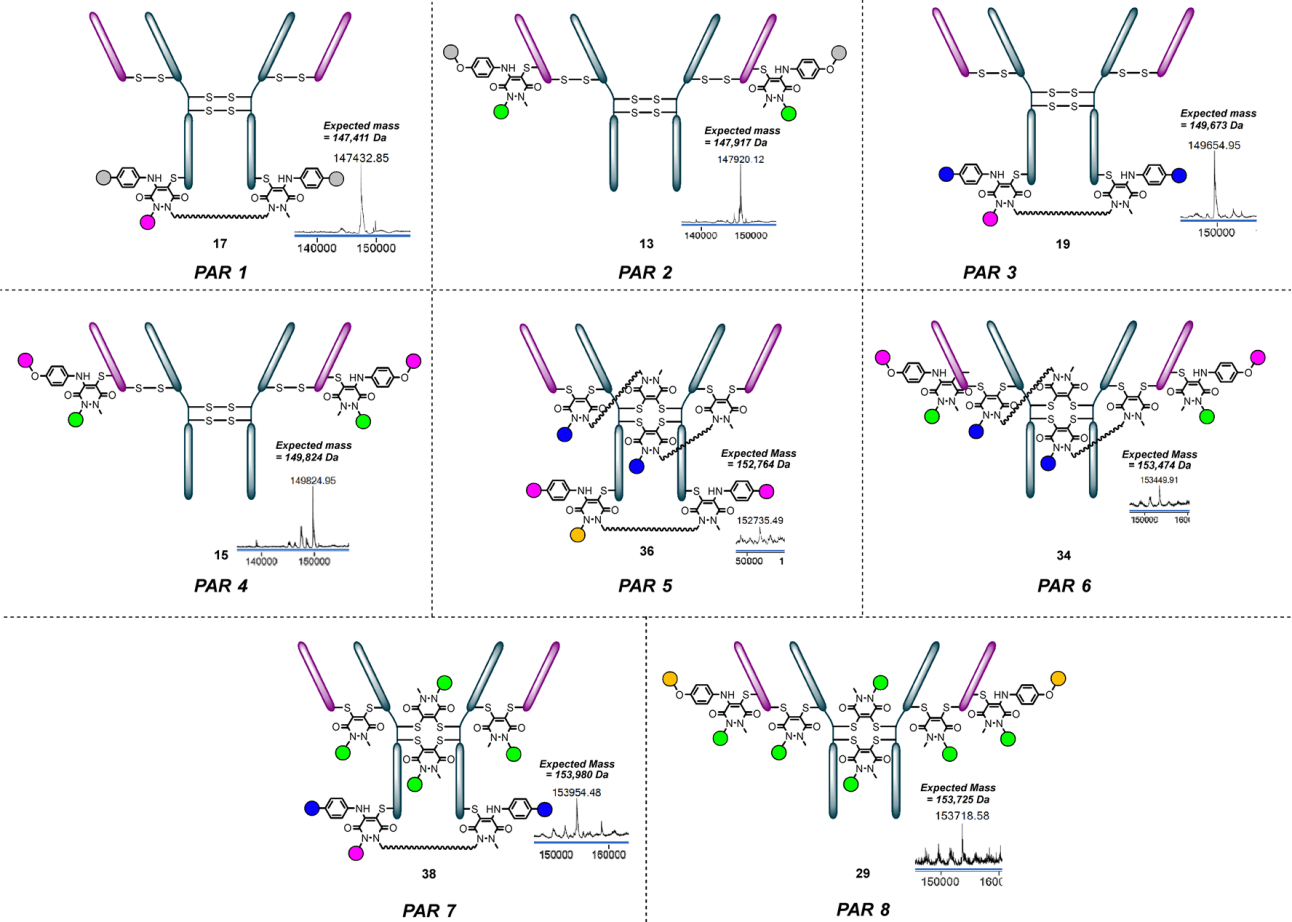
PAR 1–8 conjugates were accessed by using different combinations of Mepstra PD 1, PhN_3_ bisPD 2 and N_3_ aniline 3, in combination with various clickable payloads, on thio-trastuzumab mutants.^†^

In this manuscript we disclose a platform for the modular and site-selective construction of thio-trastuzumab mutant conjugates with payload-to-antibody ratios (PARs) of 1, 2, 3, 4, 5, 6, 7 and 8 (Fig. 14). Using only BCN PD 1, ArN_3_ bis PD 2, and N_3_ aniline 3, the framework to achieve all these PARs was enabled, including the ability to append up to three different payloads. The modularity of the chemistry and exploiting various chemoselective reactions enabled various ratios of different classes of payloads to be realised in a facile manner. Moreover, the use of click chemistry to attach payloads also adds a further level of modularity to the plug-and-play platform. The retention of binding of key representative thio-antibody conjugates to HER2 was demonstrated by ELISA, and tri-payload loaded thio-antibody conjugates were shown to internalise selectively to HER2^+^ (BT-474) over HER2^−^ (MCF-7) cells with all payloads successfully delivering into target cells only. Overall, we have demonstrated how our platform can enable the facile synthesis (multi-)payload bearing thio-antibody conjugates with PARs of 1 through to 8 with characterisation *via* LC-MS, SDS-PAGE, ELISA and on relevant cells *in vitro*.

## Author contributions

C. M. synthesised the small-molecules and carried out the bioconjugation reactions and ELISAs. I. A. T. synthesised the diMe bisPD. N. W. provided advice on bioconjugation reactions and the ELISAs. C. J. Q., M. H. and D. H. W. L. designed the imaging experiments. C. J. Q. carried out the imaging experiments. J. S. G. and M. T. W. L. helped to support C. M. at MSD and during their PhD. C. M., M. T. W. L., J. R. B. and J. S. G. and V. C. conceived and designed the project/experiments. C. M. and V. C. co-wrote the manuscript. All authors read and approved the final manuscript.

## Conflicts of interest

There are no conflicts to declare.

## Supplementary Material

CB-007-D6CB00018E-s001

CB-007-D6CB00018E-s002

## Data Availability

Synthetic chemistry experimental details, including synthetic procedures and compound characterisation studies, *i.e.*, NMR, IR and MS spectra, chemical biology experimental details, including bioconjugation procedures, LC-MS methodology, full LC-MS spectra including TIC and raw data, and full details of the *in vitro* studies have been included in the supplementary information (SI). See DOI: https://doi.org/10.1039/d6cb00018e.

## References

[cit1] Liu K., Li M., Li Y., Li Y., Chen Z., Tang Y., Yang M., Deng G., Liu H. (2024). A review of the clinical efficacy of FDA-approved antibody–drug conjugates in human cancers. Mol. Cancer.

[cit2] Mullard A. (2025). FDA approves TROP2-targeted antibody–drug conjugate for breast cancer. Nat. Rev. Drug Discovery.

[cit3] Nguyen T. D., Bordeau B. M., Balthasar J. P. (2023). Mechanisms of ADC Toxicity and Strategies to Increase ADC Tolerability. Cancers.

[cit4] Khoury R., Saleh K., Khalife N., Saleh M., Chahine C., Ibrahim R., Lecesne A. (2023). Mechanisms of Resistance to Antibody-Drug Conjugates. Int. J. Mol. Sci..

[cit5] Joubert N., Beck A., Dumontet C., Denevault-Sabourin C. (2020). Antibody-Drug Conjugates: The Last Decade. Pharmaceuticals.

[cit6] Calabretta E., Hamadani M., Zinzani P. L., Caimi P., Carlo-Stella C. (2022). The antibody-drug conjugate loncastuximab tesirine for the treatment of diffuse large B-cell lymphoma. Blood.

[cit7] Arn A.-C. C. R., Halla M. F.-C. K. J., (Hons) R., Gill S. (2023). Tisotumab Vedotin Safety and Tolerability in Clinical Practice: Managing Adverse Events. J. Adv. Pract. Oncol..

[cit8] Chudasama V., Maruani A., Caddick S. (2016). Recent advances in the construction of antibody–drug conjugates. Nat. Chem..

[cit9] Wang L., Amphlett G., Blättler W. A., Lambert J. M., Zhang W. (2005). Structural characterization of the maytansinoid-monoclonal antibody immunoconjugate, huN901-DM1, by mass spectrometry. Protein Sci..

[cit10] Walsh S. J., Bargh J. D., Dannheim F. M., Hanby A. R., Seki H., Counsell A. J., Ou X., Fowler E., Ashman N., Takada Y., Isidro-Llobet A., Parker J. S., Carroll J. S., Spring D. R. (2021). Site-selective modification strategies in antibody–drug conjugates. Chem. Soc. Rev..

[cit11] Conilh L., Sadilkova L., Viricel W., Dumontet C. (2023). Payload diversification: a key step in the development of antibody–drug conjugates. J. Hematol. Oncol..

[cit12] White J. B., Fleming R., Masterson L., Ruddle B. T., Zhong H., Fazenbaker C., Strout P., Rosenthal K., Reed M., Muniz-Medina V., Howard P., Dixit R., Wu H., Hinrichs M. J., Gao C., Dimasi N. (2019). Design and characterization of homogenous antibody-drug conjugates with a drug-to-antibody ratio of one prepared using an engineered antibody and a dual-maleimide pyrrolobenzodiazepine dimer. MAbs.

[cit13] Journeaux T., Bernardes G. J. L. (2024). Homogeneous multi-payload antibody–drug conjugates. Nat. Chem..

[cit14] Dickgiesser S., Rieker M., Mueller-Pompalla D., Schröter C., Tonillo J., Warszawski S., Raab-Westphal S., Kühn S., Knehans T., Könning D., Dotterweich J., Betz U. A. K., Anderl J., Hecht S., Rasche N. (2020). Site-Specific Conjugation of Native Antibodies Using Engineered Microbial Transglutaminases. Bioconjugate Chem..

[cit15] Dennler P., Chiotellis A., Fischer E., Brégeon D., Belmant C., Gauthier L., Lhospice F., Romagne F., Schibli R. (2014). Transglutaminase-Based Chemo-Enzymatic Conjugation Approach Yields Homogeneous Antibody–Drug Conjugates. Bioconjugate Chem..

[cit16] van Geel R., Wijdeven M. A., Heesbeen R., Verkade J. M. M., Wasiel A. A., van Berkel S. S., van Delft F. L. (2015). Chemoenzymatic Conjugation of Toxic Payloads to the Globally Conserved N-Glycan of Native mAbs Provides Homogeneous and Highly Efficacious Antibody-Drug Conjugates. Bioconjugate Chem..

[cit17] Carrico I. S., Carlson B. L., Bertozzi C. R. (2007). Introducing genetically encoded aldehydes into proteins. Nat. Chem. Biol..

[cit18] Drake P. M., Albers A. E., Baker J., Banas S., Barfield R. M., Bhat A. S., de Hart G. W., Garofalo A. W., Holder P., Jones L. C., Kudirka R., McFarland J., Zmolek W., Rabuka D. (2014). Aldehyde tag coupled with HIPS chemistry enables the production of ADCs conjugated site-specifically to different antibody regions with distinct in vivo efficacy and PK outcomes. Bioconjugate Chem..

[cit19] Hussain A. F., Kampmeier F., von Felbert V., Merk H.-F., Tur M. K., Barth S. (2011). SNAP-Tag Technology Mediates Site Specific Conjugation of Antibody Fragments with a Photosensitizer and Improves Target Specific Phototoxicity in Tumor Cells. Bioconjugate Chem..

[cit20] Zhang C., Welborn M., Zhu T., Yang N. J., Santos M. S., Van Voorhis T., Pentelute B. L. (2016). π-Clamp-mediated cysteine conjugation. Nat. Chem..

[cit21] Beerli R. R., Hell T., Merkel A. S., Grawunder U. (2015). Sortase Enzyme-Mediated Generation of Site-Specifically Conjugated Antibody Drug Conjugates with High *In Vitro* and *In Vivo* Potency. PLoS One.

[cit22] Stefan N., Gébleux R., Waldmeier L., Hell T., Escher M., Wolter F. I., Grawunder U., Beerli R. R. (2017). Highly Potent, Anthracycline-based Antibody–Drug Conjugates Generated by Enzymatic, Site-specific Conjugation. Mol. Cancer Ther..

[cit23] Murray T. V., Kozakowska-McDonnell K., Tibbles A., Taylor A., Higazi D., Rossy E., Rossi A., Genapathy S., Tamburrino G., Rath N., Tigue N., Lindo V., Vaughan T., Papworth M. A. (2021). An efficient system for bioconjugation based on a widely applicable engineered O-glycosylation tag. MAbs.

[cit24] Axup J. Y., Bajjuri K. M., Ritland M., Hutchins B. M., Kim C. H., Kazane S. A., Halder R., Forsyth J. S., Santidrian A. F., Stafin K., Lu Y., Tran H., Seller A. J., Biroc S. L., Szydlik A., Pinkstaff J. K., Tian F., Sinha S. C., Felding-Habermann B., Smider V. V., Schultz P. G. (2012). Synthesis of site-specific antibody-drug conjugates using unnatural amino acids. Proc. Natl. Acad. Sci. U. S. A..

[cit25] Junutula J. R., Raab H., Clark S., Bhakta S., Leipold D. D., Weir S., Chen Y., Simpson M., Tsai S. P., Dennis M. S., Lu Y., Meng Y. G., Ng C., Yang J., Lee C. C., Duenas E., Gorrell J., Katta V., Kim A., McDorman K., Flagella K., Venook R., Ross S., Spencer S. D., Lee Wong W., Lowman H. B., Vandlen R., Sliwkowski M. X., Scheller R. H., Polakis P., Mallet W. (2008). Site-specific conjugation of a cytotoxic drug to an antibody improves the therapeutic index. Nat. Biotechnol..

[cit26] Daver N. G., Montesinos P., DeAngelo D. J., Wang E. S., Papadantonakis N., Todisco E., Sweet K. L., Pemmaraju N., Lane A. A., Torres-Miñana L., Thompson J. E., Konopleva M. Y., Sloss C. M., Watkins K., Bedse G., Du Y., Malcolm K. E., Zweidler-McKay P. A., Kantarjian H. M. (2024). Pivekimab sunirine (IMGN632), a novel CD123-targeting antibody–drug conjugate, in relapsed or refractory acute myeloid leukaemia: a phase 1/2 study. Lancet Oncol..

[cit27] Mirzaei Y., Hussein Mer A., Fattah Maran B., Omidvar L., Misamogooe F., Amirkhani Z., Javaheri Haghighi N., Bagheri N., Keshtkaran Z., Rezaei B., Bargrizaneh F., Jahandideh S., Barpour N., Shahsavarani H., Bazyari A., Abdollahpour-Alitappeh M. (2024). Clinical and preclinical advances in PSMA-Directed Antibody-Drug conjugates (ADCs): Current status and hope for the future. Bioorg. Chem..

[cit28] Shin S. H., Ju E. J., Park J., Ko E. J., Kwon M. R., Lee H. W., Son G. W., Park Y.-Y., Kim Y. J., Song S. Y., Lee S., Seo B. S., Song J.-A., Lim S., Jung D., Kim S., Lee H., Park S. S., Jeong S.-Y., Choi E. K. (2023). ITC-6102RO, a novel B7-H3 antibody-drug conjugate, exhibits potent therapeutic effects against B7-H3 expressing solid tumors. Cancer Cell Int..

[cit29] Journeaux T., Geeson M. B., Murray T. V., Papworth M. A., Gothard M., Kettle J. G., Vasco A. V., Bernardes G. J. L. (2025). Site-Specific Quadruple-Functionalised Antibodies. Angew. Chem. Int. Ed..

[cit30] Zacharias N., Podust V. N., Kajihara K. K., Leipold D., Del Rosario G., Thayer D., Dong E., Paluch M., Fischer D., Zheng K., Lei C., He J., Ng C., Su D., Liu L., Masih S., Sawyer W., Tinianow J., Marik J., Yip V., Li G., Chuh J., Morisaki J. H., Park S., Zheng B., Hernandez-Barry H., Loyet K. M., Xu M., Kozak K. R., Phillips G. L., Shen B.-Q., Wu C., Xu K., Yu S.-F., Kamath A., Rowntree R. K., Reilly D., Pillow T., Polson A., Schellenberger V., Hazenbos W. L. W., Sadowsky J. (2022). A homogeneous high-DAR antibody–drug conjugate platform combining THIOMAB antibodies and XTEN polypeptides. Chem. Sci..

[cit31] Zhou Q., Stefano J. E., Manning C., Kyazike J., Chen B., Gianolio D. A., Park A., Busch M., Bird J., Zheng X., Simonds-Mannes H., Kim J., Gregory R. C., Miller R. J., Brondyk W. H., Dhal P. K., Pan C. Q. (2014). Site-Specific Antibody–Drug Conjugation through Glycoengineering. Bioconjugate Chem..

[cit32] van Geel R., Wijdeven M. A., Heesbeen R., Verkade J. M. M., Wasiel A. A., van Berkel S. S., van Delft F. L. (2015). Chemoenzymatic Conjugation of Toxic Payloads to the Globally Conserved N-Glycan of Native mAbs Provides Homogeneous and Highly Efficacious Antibody–Drug Conjugates. Bioconjugate Chem..

[cit33] Yamada K., Shikida N., Shimbo K., Ito Y., Khedri Z., Matsuda Y., Mendelsohn B. A. (2019). AJICAP: Affinity Peptide Mediated Regiodivergent Functionalization of Native Antibodies. Angew. Chem. Int. Ed..

[cit34] Ohata J., Ball Z. T. (2017). A Hexa-rhodium Metallopeptide Catalyst for Site-Specific Functionalization of Natural Antibodies. J. Am. Chem. Soc..

[cit35] Schumacher F. F., Nunes J. P. M., Maruani A., Chudasama V., Smith M. E. B., Chester K. A., Baker J. R., Caddick S. (2014). Next generation maleimides enable the controlled assembly of antibody-drug conjugates via native disulfide bond bridging. Org. Biomol. Chem..

[cit36] Badescu G., Bryant P., Bird M., Henseleit K., Swierkosz J., Parekh V., Tommasi R., Pawlisz E., Jurlewicz K., Farys M., Camper N., Sheng X., Fisher M., Grygorash R., Kyle A., Abhilash A., Frigerio M., Edwards J., Godwin A. (2014). Bridging Disulfides for Stable and Defined Antibody Drug Conjugates. Bioconjugate Chem..

[cit37] Walsh S. J., Iegre J., Seki H., Bargh J. D., Sore H. F., Parker J. S., Carroll J. S., Spring D. R. (2020). General dual functionalisation of biomacromolecules via a cysteine bridging strategy. Org. Biomol. Chem..

[cit38] Bahou C., Chudasama V. (2022). The use of bromopyridazinedione derivatives in chemical biology. Org. Biomol. Chem..

[cit39] Chudasama V., Smith M. E. B., Schumacher F. F., Papaioannou D., Waksman G., Baker J. R., Caddick S. (2011). Bromopyridazinedione-mediated protein and peptide bioconjugation. Chem. Commun..

[cit40] Javaid F., Pilotti C., Camilli C., Kallenberg D., Bahou C., Blackburn J., Baker J. R., Greenwood J., Moss S. E., Chudasama V. (2021). Leucine-rich alpha-2-glycoprotein 1 (LRG1) as a novel ADC target. RSC Chem. Biol..

[cit41] Maruani A., Smith M. E. B., Miranda E., Chester K. A., Chudasama V., Caddick S. (2015). A plug-and-play approach to antibody-based therapeutics via a chemoselective dual click strategy. Nat. Commun..

[cit42] Lee M. T. W., Maruani A., Richards D. A., Baker J. R., Caddick S., Chudasama V. (2017). Enabling the controlled assembly of antibody conjugates with a loading of two modules without antibody engineering. Chem. Sci..

[cit43] Dannheim F. M., Walsh S. J., Orozco C. T., Hansen A. H., Bargh J. D., Jackson S. E., Bond N. J., Parker J. S., Carroll J. S., Spring D. R. (2022). All-in-one disulfide bridging enables the generation of antibody conjugates with modular cargo loading. Chem. Sci..

[cit44] de Bever L., Popal S., van Schaik J., Rubahamya B., van Delft F. L., Thurber G. M., van Berkel S. S. (2023). Generation of DAR1 Antibody-Drug Conjugates for Ultrapotent Payloads Using Tailored GlycoConnect Technology. Bioconjugate Chem..

[cit45] Bahou C., Szijj P. A., Spears R. J., Wall A., Javaid F., Sattikar A., Love E. A., Baker J. R., Chudasama V. (2021). A Plug-and-Play Platform for the Formation of Trifunctional Cysteine Bioconjugates that also Offers Control over Thiol Cleavability. Bioconjugate Chem..

[cit46] Walker J. A., Bohn J. J., Ledesma F., Sorkin M. R., Kabaria S. R., Thornlow D. N., Alabi C. A. (2019). Substrate Design Enables Heterobifunctional, Dual “Click” Antibody Modification via Microbial Transglutaminase. Bioconjugate Chem..

[cit47] Hanby A. R., Walsh S. J., Counsell A. J., Ashman N., Mortensen K. T., Carroll J. S., Spring D. R. (2022). Antibody dual-functionalisation enabled through a modular divinylpyrimidine disulfide rebridging strategy. Chem. Commun..

[cit48] Thoreau F., Rochet L. N. C., Baker J. R., Chudasama V. (2023). Enabling the formation of native mAb, Fab′ and Fc-conjugates using a bis-disulfide bridging reagent to achieve tunable payload-to-antibody ratios (PARs). Chem. Sci..

[cit49] Nunes J. P. M., Vassileva V., Robinson E., Morais M., Smith M. E. B., Pedley R. B., Caddick S., Baker J. R., Chudasama V. (2017). Use of a next generation maleimide in combination with THIOMABTM antibody technology delivers a highly stable, potent and near homogeneous THIOMABTM antibody-drug conjugate (TDC). RSC Adv..

[cit50] Bahou C., Richards D. A., Maruani A., Love E. A., Javaid F., Caddick S., Baker J. R., Chudasama V. (2018). Highly homogeneous antibody modification through optimisation of the synthesis and conjugation of functionalised dibromopyridazinediones. Org. Biomol. Chem..

