# Supplementary material for: Enabling the synthesis of multi-payload thio-antibody conjugates through the use of pyridazinediones, p-anisidine derivatives and various click chemistries
Source: RSC Chem Biol. 2026 Feb 3;7(4):656–68. doi: 10.1039/d6cb00018e (PMC12917607; doi:10.1039/d6cb00018e)
Supplement: CB-007-D6CB00018E-s001 [file CB-007-D6CB00018E-s001.pdf]

Supplementary Information (SI) for RSC Chemical Biology.
This journal is © The Royal Society of Chemistry 2026

Enabling the synthesis of multi-payload thio-antibody conjugates through the use of pyridazinediones, *p*-anisidine derivatives and various click chemistries

Supplementary Information

General Experimental

Chemicals

All reagents were purchased from Sigma Aldrich, Alfa Aesar or ThermoFisher Scientific and were used as received unless otherwise stated. Solvents were used as received unless otherwise stated. GFP S147C was kindly supplied by Dr Ioanna Thanasi (UCL Chemistry), having been expressed according to literature.[1]

Chromatography

All reactions were magnetically stirred and monitored by thin-layer chromatography (TLC) on pre-coated silica gel plates (254 µm). Silica gel plates were initially examined under UV light and then developed using aqueous potassium permanganate/ninhydrin/bromocresol green stains. Flash column chromatography was carried out with pre-loaded FlashPure™ cartridges on a Biotage® Isolera Spektra One flash chromatography system.

Spectroscopy

Nuclear magnetic resonance (NMR) spectra were obtained using Bruker Avance Neo 700, Avance III 600, DRX 500 or Avance III 400 NMR instruments. All samples were run at 25 °C. Chemical shifts (δ) are quoted on a parts per million (ppm) scale relative to tetramethylsilane, calibrated using residual signals of the solvent. In the case of amide rotamers and where possible, only the major rotamer has been assigned, and areas underneath all rotameric peaks have been considered for integration calculations. Coupling constants (*J*) are reported in Hertz (Hz) and are reported as $3J_{H\text{-}H}$ couplings between protons. Infrared (IR) spectra were obtained on a Perkin Elmer Spectrum 100 FTIR spectrometer operating in ATR mode. Small molecule mass spectra were obtained from the UCL mass spectroscopy service on an Orbitrap Q Exactive mass spectrometer.

LC-MS Method 1

Molecular masses of all protein conjugates were measured using an Agilent 6510 QTOF LC-MS system (Agilent, UK) (Method 1a) or an Agilent 6530 QTOF LC-MS system (Agilent, UK) (Method 1b).

Method 1a

The Agilent 1200 HPLC system was equipped with an Agilent PLRP-S, 1000A, 8 µM, 150 mm × 2.1 120 mm column. 10 µL of a protein sample (diluted to 5 µM in LC-MS grade water) was separated on the column using mobile phase A (water-0.1% formic acid) and B (acetonitrile-0.1% formic acid) with an eluting gradient (Table S1) at a flow rate of 0.3 mL/min. The oven temperature was maintained at 60 °C. The column effluent was continuously electrosprayed into capillary ESI source of the Agilent 6510 QTOF mass spectrometer.

Time (min)	Solvent A (%)	Solvent B (%)
0	85	15

3	68	32
14	65	35
18	5	95
22	85	15
25	85	15

Table S1. LC-MS mobile phase gradient for A/B elution.

The Agilent 6510 QTOF mass spectrometer was operated in a positive polarity mode, coupled with an ESI ion source. The ion source parameters were set up with a VCap of 3500 V, a gas temperature at 350 °C, a dry gas flow rate at 10 L/min and a nebulizer of 30 psig. MS Tof was acquired under conditions of a fragmentor at 350 V, a skimmer at 65 V and an acquisition rate at 0.5 spectra/s in a profile mode, within a scan range between 700 and 4500 m/z. The data were analysed by deconvoluting a spectrum to a zero-charge mass spectrum using a maximum entropy deconvolution algorithm using MassHunter Qualitative Analysis version B.07.00.

Method 1a*

The Agilent 1200 HPLC system was equipped with an Agilent PLRP-S, 1000A, 8 µM, 150 mm × 2.1 120 mm column. 10 µL of a protein sample (diluted to 0.2 mg/mL in LC-MS grade H_2O) was separated on the column using mobile phase A (water-0.1% formic acid) and B (acetonitrile-0.1% formic acid) with an eluting gradient (Table S2) at a flow rate of 0.3 mL/min. The oven temperature was maintained at 60 °C. The column effluent was continuously electrosprayed into capillary ESI source of the Agilent 6510 QTOF mass spectrometer.

Time (min)	Solvent A (%)	Solvent B (%)
0	75	25
2	75	25
16	1	99
18	1	99
18.1	75	25
20	75	25

Table S2. LCMS mobile phase gradient for A/B elution.

The Agilent 6510 QTOF mass spectrometer was operated in a positive polarity mode, coupled with an ESI ion source. The ion source parameters were set up with a VCap of 4000 V, a gas temperature at 350 °C, a dry gas flow rate at 10 L/min and a nebuliser of 35 psig. MS Tof was acquired under conditions of a fragmentor at 175 V, a skimmer at 65 V and an acquisition rate at 1 spectra/s in a profile mode, within a scan range between 100 and 3100 m/z. The data were analysed by deconvoluting a spectrum to a zero-charge mass spectrum using a maximum entropy deconvolution algorithm using MassHunter Qualitative Analysis version B.07.00.

Method 1b

Agilent 1290 Infinity II UHPLC system was equipped with an Agilent PLRP-s, 1000 Å, 8 µm 50 mm × 2.1 mm column. 2 µL of protein sample (diluted to 5 µM in LC-MS grade water) was separated on the column using mobile phase A (water, 0.1% formic acid) and B (acetonitrile, 0.1% formic acid) with an eluting gradient (shown below) at a flow rate of 0.8 mL/min. Oven temperature was maintained at 60 °C. LC-MS mobile phase gradient for A/B elution:

Time (min)	Solvent A (%)	Solvent B (%)
0	80	20
1	80	20
6.5	40	60
7.5	40	60
7.6	80	20
8.5	80	20

Table S3. LC-MS mobile phase gradient for A/B elution.

Agilent 6530 QTOF mass spectrometer was operated in positive polarity mode, coupled with an ESI ion source. The ion source parameters were set up with a VCap of 4000 V, a gas temperature at 350 °C, a dry gas flow rate at 10 L/min and a nebulizer of 35 psig. MS TOF was acquired under conditions of a fragmentor at 175 V, a skimmer at 65 V and an acquisition rate at 1 spectra/s in a profile mode, within a scan range between 100 and 7000 m/z. The data were then analysed by deconvoluting a spectrum to a zero-charge mass spectrum using a maximum entropy deconvolution algorithm within the MassHunter software version B.07.00.

LC-MS analyses are shown as mentioned here: TIC LC-MS trace (top), non-deconvoluted LC-MS trace (upper middle), wide range deconvoluted MS data (lower middle) and zoomed in deconvoluted data (bottom) for each species. It is acknowledged that the signal intensity of the mass spectra of conjugates decreases as one goes through a reaction sequence - we believe this is due to the loss material over the sequence of steps.

Antibody samples (40 µL, 5 µM, in ammonium acetate buffer (0.2 M, pH 6.8)/water) were deglycosylated by incubating with PNGase F (0.67 µL, New England BioLabs) at 22 °C for 16 h.

LC-MS Method 2

LCMS was performed using a Waters Acquity UPLC connected to a Waters Acquity Single Quad Detector (SQD). Separations were performed on Column: XBridge OST C18 Column, 130Å, 2.5 µm, 2.1 mm X 50 mm at a flow rate of 0.6 mL/min; with UV detection wavelength set at 214 nm; with a mobile phase consisting of 0.1% formic acid in water (solvent A) and 0.1% formic acid in acetonitrile (solvent B). The eluting gradient is shown below (Table S4). Mass spectra were analysed using MassLynx software.

Time (min)	Solvent A (%)	Solvent B (%)

0	95	5
0.5	95	5
3.5	5	95
4	5	95
4.5	95	5
5	95	5

Table S4. LCMS mobile phase gradient for A/B elution.

UV-Vis Spectroscopy

UV-Vis spectroscopy was used to determine protein concentrations and PARs (PDARs/FARs) using a NanoDrop™ One microvolume spectrophotometer operating at room temperature. Sample buffer was used as blank for baseline correction. Extinction coefficients for proteins (at A_{280}) and payloads (at $A_{\lambda Max}$) are listed below. Where a value was not available (either from the literature or the chemical datasheet), one was calculated from the protein sequence using ProtParam. A correction factor was applied in the event that the conjugated payload had a competing absorption at A_{280} (Table S5). PAR values were calculated by comparing concentrations of the payloads and the protein (calculated with corrected A_{280} values).

Molecule	Extinction Coefficient $\varepsilon_{\lambda max}$ (M^{-1} cm^{-1})	Correction Factor at A_{280}
Trastuzumab	215,000 (ε_{280})[2]	-
GFP S147C	20,500 (ε_{280})[3]	-
PD Scaffold	9,100 (ε_{335})[2]	0.25
Azide-fluor 488	74,000 (ε_{505})	0.11
BP Fluor 568	88,000 (ε_{578})	0.46
BP Fluor 647	270,000 (ε_{671})	0.03

Table S5. Extinction coefficients of proteins and payloads used.

Concentration (c) and PDARs of conjugates were calculated by UV-Vis using the following equations:

$$A_{280} = c(\varepsilon_{280(Ab)} + 0.25 \cdot \varepsilon_{335(PD)})$$

$$PDAR = \frac{\varepsilon_{280(Ab)} \cdot A_{335}}{A_{280} \cdot \varepsilon_{335(PD)} - 0.25 \cdot A_{335} \cdot \varepsilon_{335(PD)}}$$

FARs were calculated using the following equation:

$$FAR = \frac{A_{\lambda_{max}}|(\varepsilon_{280(Ab)} + 0.25 \cdot n \cdot \varepsilon_{335(PD)})}{\varepsilon_{\lambda_{max}(fluorophore)}(A_{280} - 0.11 \cdot A_{\lambda_{max}})}$$

where *n* = PDAR as calculated directly after re-bridging and λ_{Max} = wavelength of max absorption for the fluorophore.

SDS-PAGE

Non-reducing glycine-SDS-PAGE 12% or 4–15% acrylamide gels were performed following standard lab procedures. A 6% stacking gel was used and a broad-range molecular weight marker (10-250 kDa, Prestained PageRuler Plus Protein Standards, ThermoScientific) was run alongside the samples to estimate protein weights. Samples (3 µL at ~ 6 µM) were mixed with loading buffer (3 µL, composition for 5 × SDS: 1 g SDS, 3 mL glycerol, 6 mL 0.5 M Tris buffer pH 6.8, 2 mg bromophenol blue in 10 mL), heated at 80 °C for 5 min, and centrifuged at 10,000 rcf for 5 min. Samples were subsequently loaded into the wells in a volume of 6 µL. All gels were run at a constant voltage of 200 V for 60 min using 1 × SDS running buffer. Gels were stained using a modified Coomassie stain (25 g ammonium sulfate, 250 mg Coomasie G-250, 8.8 mL 85% orthophosphoric acid, 50 mL ethanol, made up to a total of 250 mL with dH_2O) at 21 °C for 16 h.

All ladders are from the same gel in which samples were run. In some cases, lanes are cut out where they were occupied by samples from other reactions run in different lanes in the same gel and are between the sample in question and the ladder. In this case, there will be a space between the image of the ladder and the image of the sample.

Densitometry was performed using imageJ software. Background subtraction was achieved using the built-in plugin with a rolling ball radius of 30, sliding paraboloid, and smoothing. Brightness and contrast settings were auto-adjusted within the software.

Antibodies

Native trastuzumab refers to Ontruzant®.

The HC S378C and LC S168C engineered cysteine mutants were kindly provided by Merck Sharp & Dohme Corp. UK (MSD UK). The HC S378C thio-trastuzumab mutant refers to Humanized x [ERBB2_H] mAb (Trastuzumab VH S378C/VL WT, often referred to herein as HC S378C) IgG_1/Kappa antibodies. This mutant was originally supplied in buffer solution (20 mM sodium acetate, pH 5.5, 9% sucrose buffer). The LC S168C thio-trastuzumab mutant refers to Humanized x [ERBB2_H] mAb (Trastuzumab VH WT/VL S168C, often referred to herein as LC S168C) IgG_1/Kappa antibodies. This mutant was originally supplied in buffer solution (60 mM sodium acetate, 14 mM Tris; 3% sucrose, pH 5.5 buffer).

In the case of thio-antibody mutants, +1445 peaks on LC-MS correspond to incomplete deglycosylation.

Where 4 °C is written, the reaction was incubated in a ThermoMixer® set to 4 °C, however, in many cases the actual temperature of the ThermoMixer® was 7-12 °C due to difficulty cooling down.

Antibody conjugate isomers

We note that the formation of half antibody conjugate species, owing to some non-native PD rebridging in the hinge region when reacting the reduced native disulfide bonds of thio-antibodies (or conjugates) thereof with BCN PD **1** and/or ArN_3 bisPD **2** or derivatives thereof (in all relevant experiments described below), is likely, but is typical when using disulfide rebridging reagents to rebridge antibody disulfide bonds.

Synthesis

Synthesis of BCN PD 1

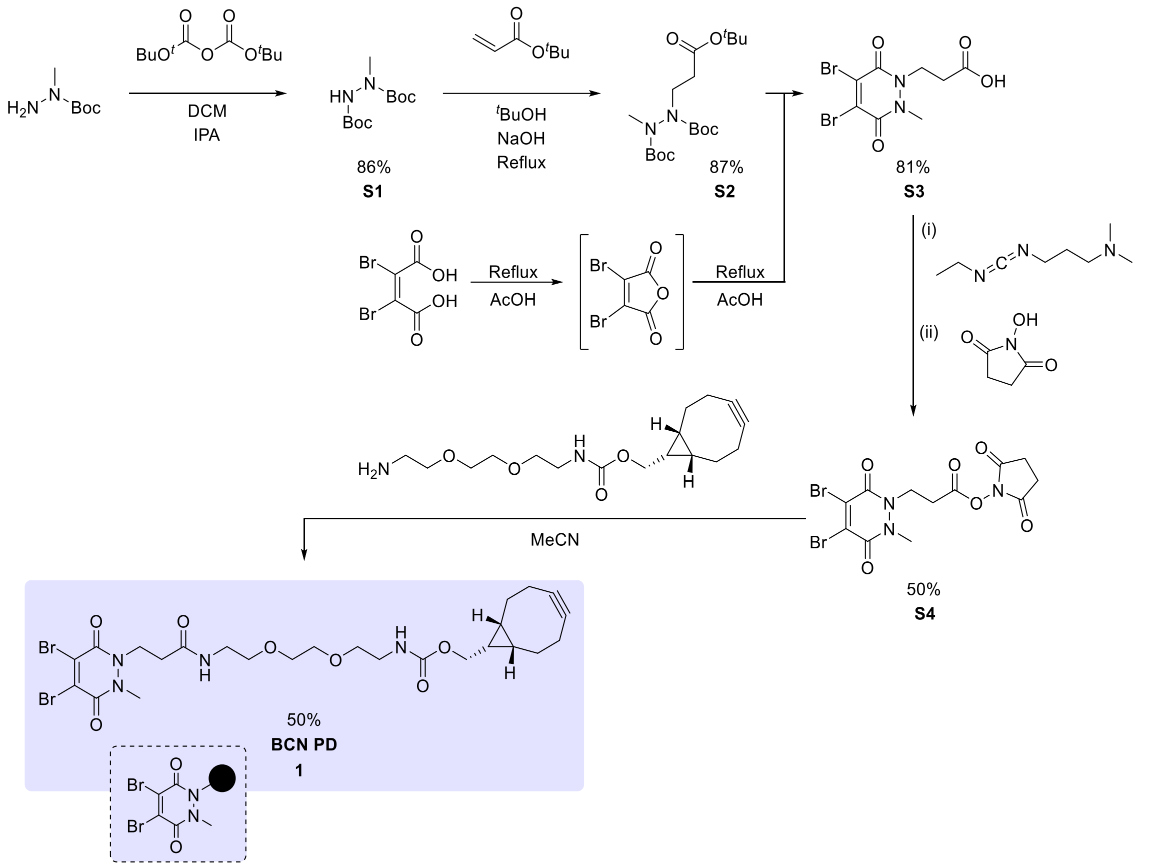

Figure S1. Synthetic Route to BCN PD, **1**. Firstly, 1-boc-1-methylhydrazine is protected using Boc_2O, to form protected hydrazine **S1**. *Tert*-butyl acrylate is then reacted with **S1** to form the acid hydrazine **S2**, which is refluxed with dibromomaleic anhydride to form MetPAc **S3**. MetPAc **S3** is activated with EDC then NHS and the methyl/NHS ester PD **S4** isolated. **S4** is subsequently reacted with BCN amine to form **1**.

Di-*tert*-butyl-1-methylhydrazine-1,2-dicarboxylate S1[2]

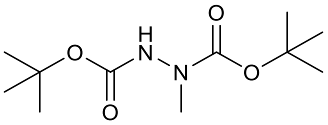

S1

To a solution of 1-boc-1-methylhydrazine (3.00 mL, 20.2 mmol) in IPA (16 mL) was added dropwise over 30 min di-*tert*-butyl decarbonate (5.00 g, 22.9 mmol) pre-dissolved in DCM (12 mL). The reaction was then stirred at 21 °C for 18 h. Following this, the solvents were removed *in vacuo* and the crude residue purified by flash column chromatography (0% to 20% EtOAc/cyclohexane) to afford di-*tert*-butyl-1-methylhydrazine1,2-dicarboxylate **S1** (4.29 g, 17.4 mmol, 86%) as a white solid.

^{1}H NMR (600 MHz, $CDCl_3$, major rotamer) δ 6.40 (s, 1H) 3.10 (s, 3H), 1.46 (s, 18H).

^{13}C NMR (150 MHz, $CDCl_3$, major rotamer) δ 155.9 (C), 81.1 (C), 37.6 (CH_3), 28.3 (CH_3).

IR (solid) 3314, 2980, 2933, 1702 cm^{-1}.

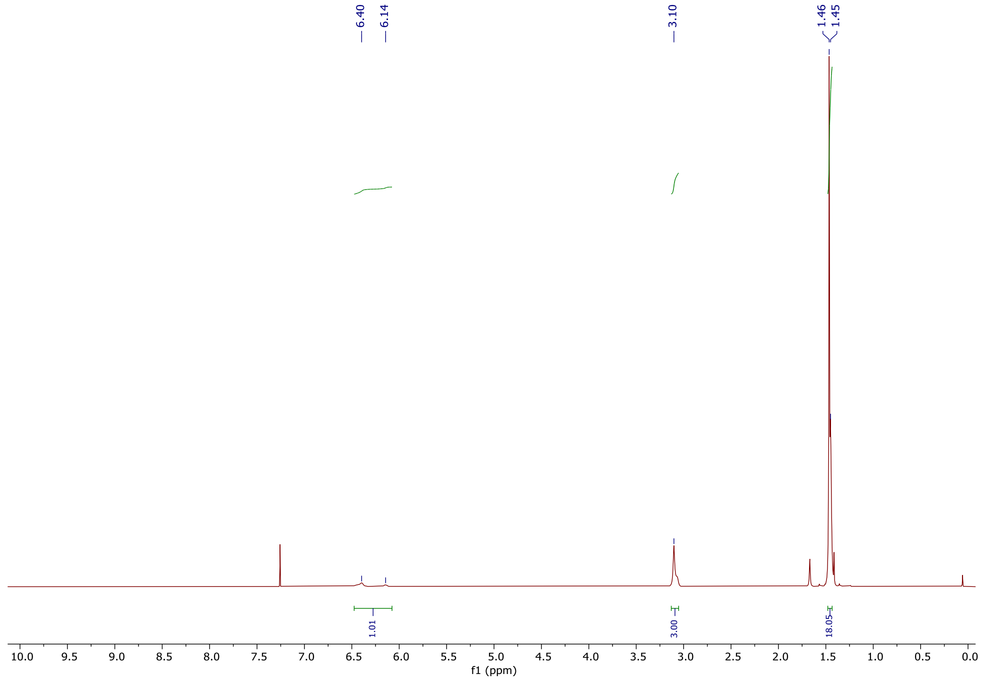

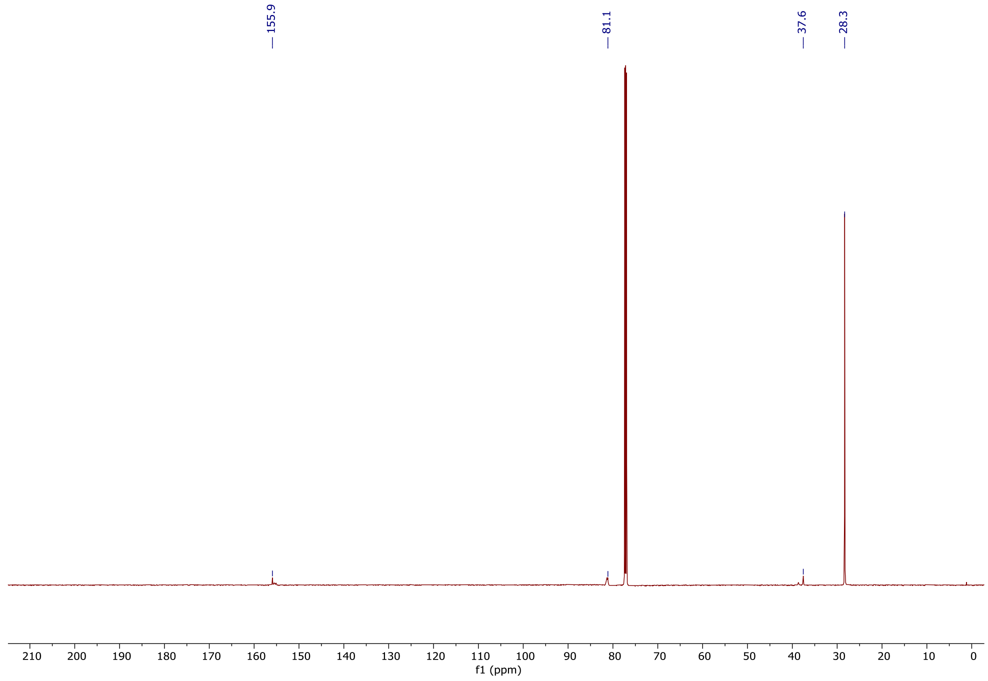

Di-*tert*-butyl-1-(3-(*tert*-butoxy)-3-oxopropyl)-2-methylhydrazine-1,2-dicarboxylate S2[2]

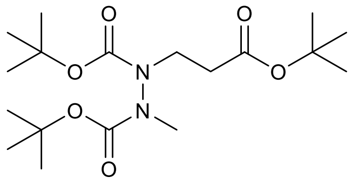

S2

To a solution of di-*tert*-butyl 1-methylhydrazine-1,2-dicarboxylate **S1** (3.75 g, 15.2 mmol) in *tert*-butanol (25 mL), was added 2 M NaOH (0.5 mL), and the reaction mixture stirred at 21 °C for 10 min. After this, *tert*-butyl acrylate (6.63 mL, 45.7 mmol) was added to the solution and the reaction mixture was heated under reflux for 68 h. The solvent was then removed *in vacuo* and the product suspended in water (50 mL). The product was then extracted into ethyl acetate (8 × 50 mL) and dried ($MgSO_4$) to give di-*tert*-butyl-1-(3-(*tert*-butoxy)-3-oxopropyl)-2-methylhydrazine-1,2-dicarboxylate **S2** as a clear oil (4.95 g, 13.2 mmol, 87%).

^{1}H NMR (600 MHz, $CDCl_3$, rotamers) δ 3.84–3.51 (m, 2H), 3.05–2.98 (m, 3H), 2.52-2.49 (m, 2H), 1.47–1.42 (m, 27H).

^{13}C NMR (150 MHz, $CDCl_3$) δ 171.1 (C), 155.5 (C), 154.5 (C), 81.1 (C), 80.9 (C), 44.6 (CH_2), 36.7 (CH_3), 34.2 (CH_2), 28.4 (CH_3), 28.2 (CH_3).

IR (thin film) 2977, 2933, 1709 cm^{-1}.

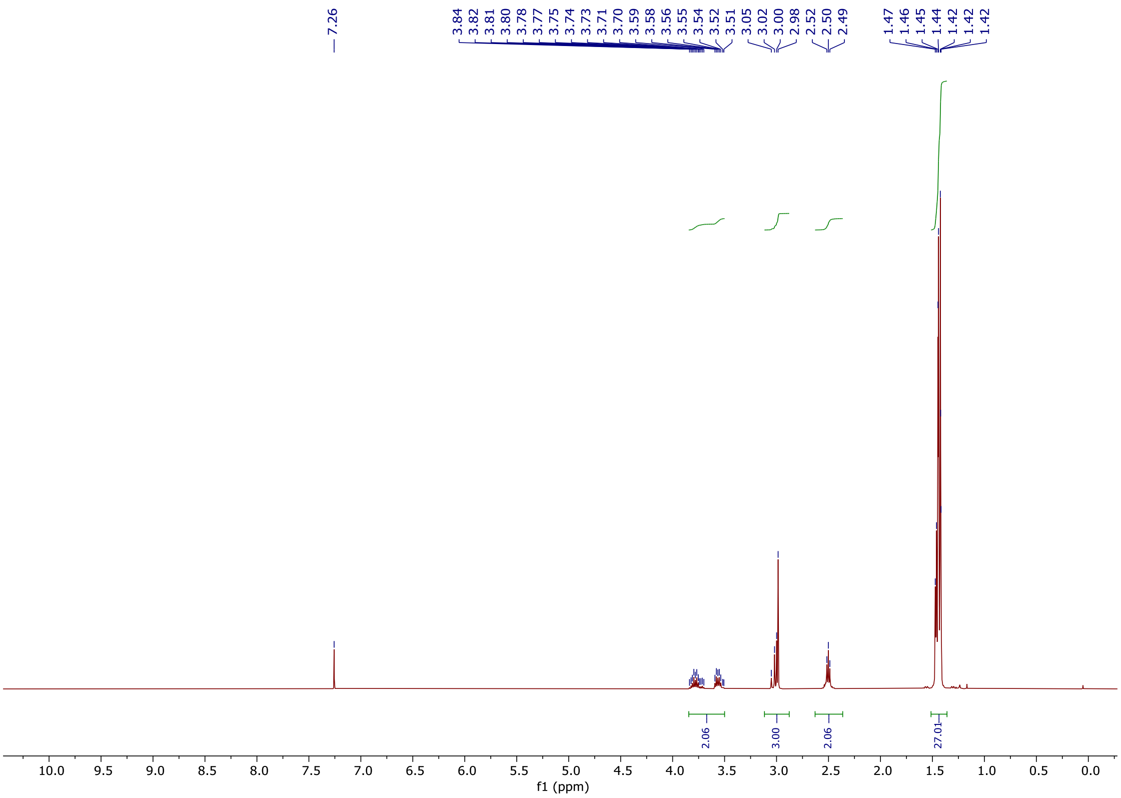
7.26
3.84
3.82
3.81
3.80
3.78
3.77
3.75
3.74
3.73
3.71
3.70
3.59
3.58
3.56
3.55
3.54
3.52
3.51
3.05
3.02
3.00
2.98
2.52
2.50
2.49
1.47
1.46
1.45
1.44
1.42
1.42
1.42
2.06
3.00
2.06
27.01
10.0 9.5 9.0 8.5 8.0 7.5 7.0 6.5 6.0 5.5 5.0 4.5 4.0 3.5 3.0 2.5 2.0 1.5 1.0 0.5 0.0
f1 (ppm)

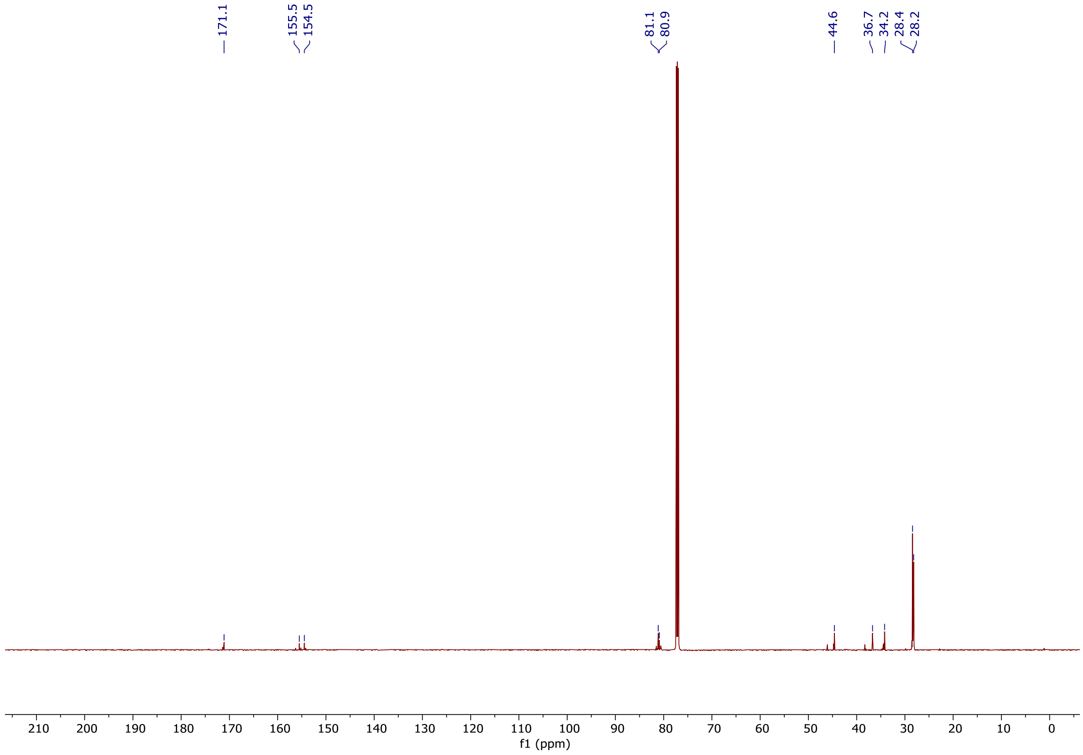
171.1
155.5
154.5
81.1
80.9
44.6
36.7
34.2
28.4
28.2
210 200 190 180 170 160 150 140 130 120 110 100 90 80 70 60 50 40 30 20 10 0
f1 (ppm)

3-(4,5-Dibromo-2-methyl-3,6-dioxo-3,6-dihydropyridazin-1(2*H*)-yl) propanoic acid S3[2]

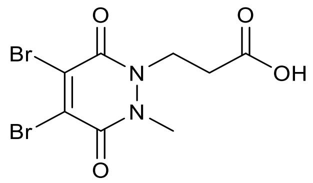

S3

Dibromomaleic acid (5.32 g, 19.4 mmol) was dissolved in AcOH (100 mL) and the solution heated under reflux for 30 min. To this solution was added di-*tert*-butyl-1-(3-(*tert*-butoxy)-3-oxopropyl)-2-methylhydrazine-1,2-dicarboxylate **S2** (6.08 g, 16.2 mmol) and the reaction heated under reflux for a further 6 h. After this time, the reaction mixture was concentrated *in vacuo* with toluene co-evaporation (3 × 30 mL, as an azeotrope) and chloroform co-evaporation (30 mL) and the crude residue purified by flash column chromatography (50% to 100% EtOAc/cyclohexane (1% AcOH)) to afford 3-(4,5-dibromo-2-methyl-3,6-dioxo-3,6-dihydropyridazin-1(2*H*)-yl) propanoic acid **S3** (4.66 g, 13.1 mmol, 81%) as a yellow solid.

^{1}H NMR (600 MHz, MeOD) δ 4.43 (t, *J* = 7.3 Hz, 2H), 3.69 (s, 3H), 2.74 (t, *J* = 7.3 Hz, 2H).

^{13}C NMR (150 MHz, MeOD) δ 173.8 (C), 154.7 (C), 154.5 (C), 136.7 (C), 136.4 (C), 44.9 (CH_3), 35.4 (CH_2), 32.5 (CH_2).

IR (thin film) 3226, 2945, 2835, 1731, 1660, 1572 cm^{-1}.

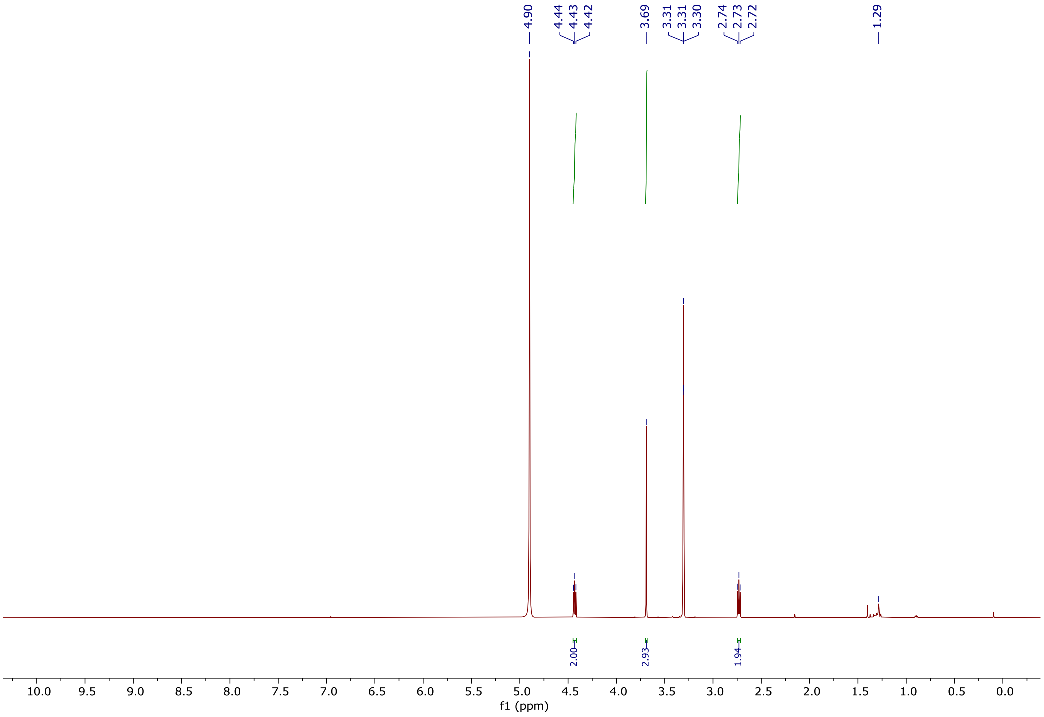
4.90
4.44
4.43
4.42
3.69
3.31
3.31
3.30
2.74
2.73
2.72
1.29
2.00
2.93
1.94
10.0 9.5 9.0 8.5 8.0 7.5 7.0 6.5 6.0 5.5 5.0 4.5 4.0 3.5 3.0 2.5 2.0 1.5 1.0 0.5 0.0
f1 (ppm)

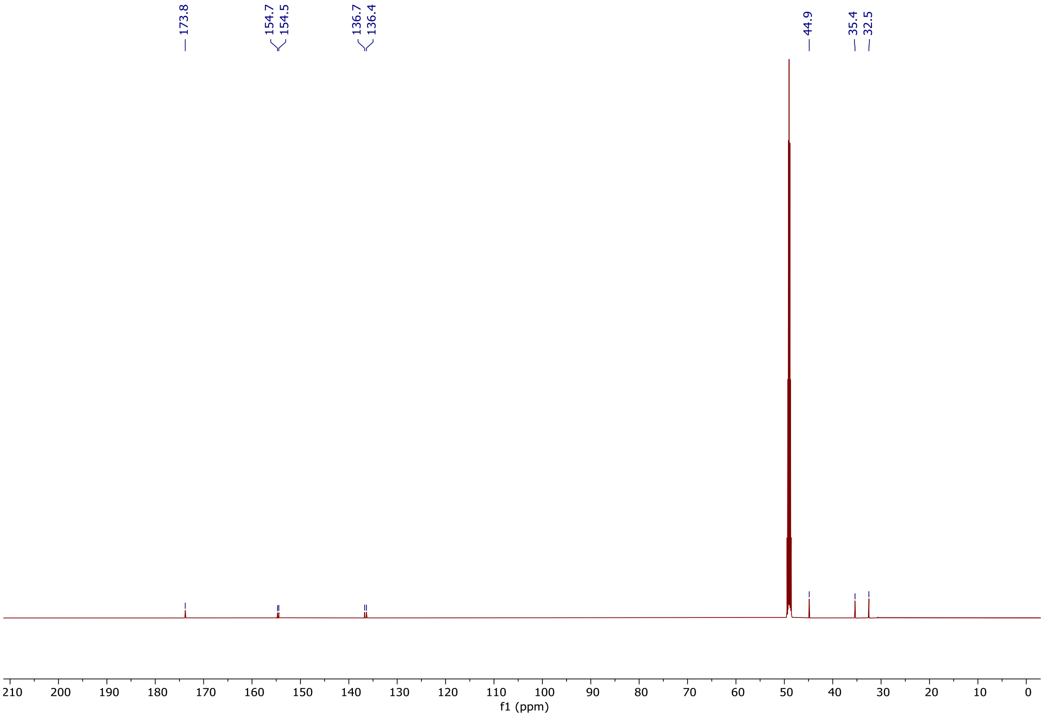
173.8
154.7
154.5
136.7
136.4
44.9
35.4
32.5
210 200 190 180 170 160 150 140 130 120 110 100 90 80 70 60 50 40 30 20 10 0
f1 (ppm)

2,5-Dioxopyrrolidin-1-yl 3-(4,5-dibromo-2-methyl-3,6-dioxo-3,6-dihydropyridazin1(2*H*)-yl) propanoate S4[2]

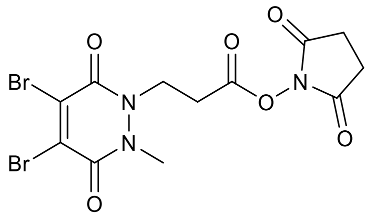

S4

To a solution of 3-(4,5-dibromo-2-methyl-3,6-dioxo-3,6-dihydropyridazin-1(2*H*)-yl) propanoic acid **S3** (704 mg, 1.98 mmol) in THF (20 mL), pre-cooled to 0 °C, was added *N*-(3-dimethylaminopropyl)-*N*′-ethylcarbodiimide (414 mg, 2.16 mmol). The homogeneous solution was then stirred at 0 °C for 30 min. Following this, was added *N*-hydroxysuccinimide (249 mg, 2.16 mmol) and the reaction stirred at 21 °C for a further 18 h. The solvent was then removed *in vacuo* and the crude residue dissolved in DCM. This was then filtered, and the filtrate concentrated *in vacuo*. Purification of the crude residue by flash column chromatography (50% to 100% EtOAc/cyclohexane) afforded 2,5-dioxopyrrolidin-1-yl 3-(4,5-dibromo-2-methyl-3,6-dioxo-3,6-dihydropyridazin-1(2*H*)-yl) propanoate **S4** (488 mg, 0.99 mmol, 50%) as a yellow solid.

^{1}H NMR (600 MHz, $CDCl_3$) δ 4.47 (t, *J* = 6.9 Hz, 2H), 3.67 (s, 3H), 3.10 (t, *J* = 6.9 Hz, 2H), 2.84 (s, 4H).

^{13}C NMR (151 MHz, $CDCl_3$) δ 168.8 (C), 166.1 (C), 153.3 (C), 153.1 (C), 136.9 (C), 135.3 (C), 43.0 (CH_2), 35.3 (CH_3), 29.1 (CH_2), 25.7 (CH_2).

IR (solid) 2919, 2852, 1810, 1780, 1732, 1624, 1577 cm^{-1}.

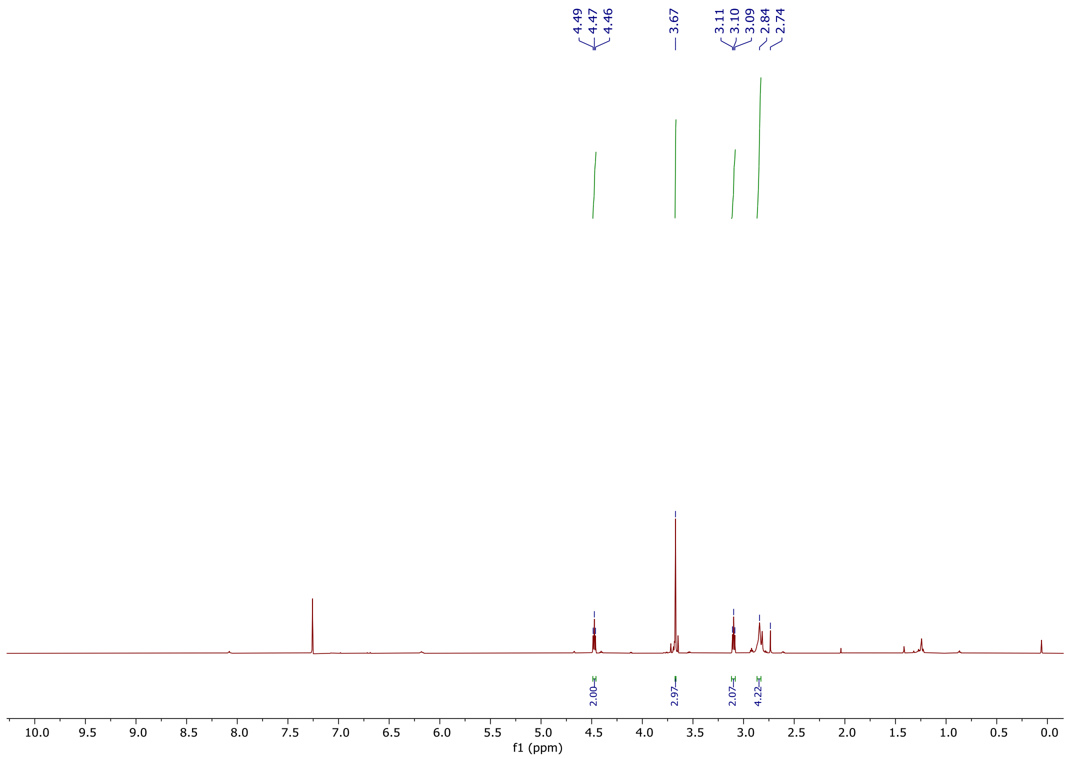
4.49
4.47
4.46
3.67
3.11
3.10
3.09
2.84
2.74
2.00
2.97
2.07
4.22
10.0 9.5 9.0 8.5 8.0 7.5 7.0 6.5 6.0 5.5 5.0 4.5 4.0 3.5 3.0 2.5 2.0 1.5 1.0 0.5 0.0
f1 (ppm)
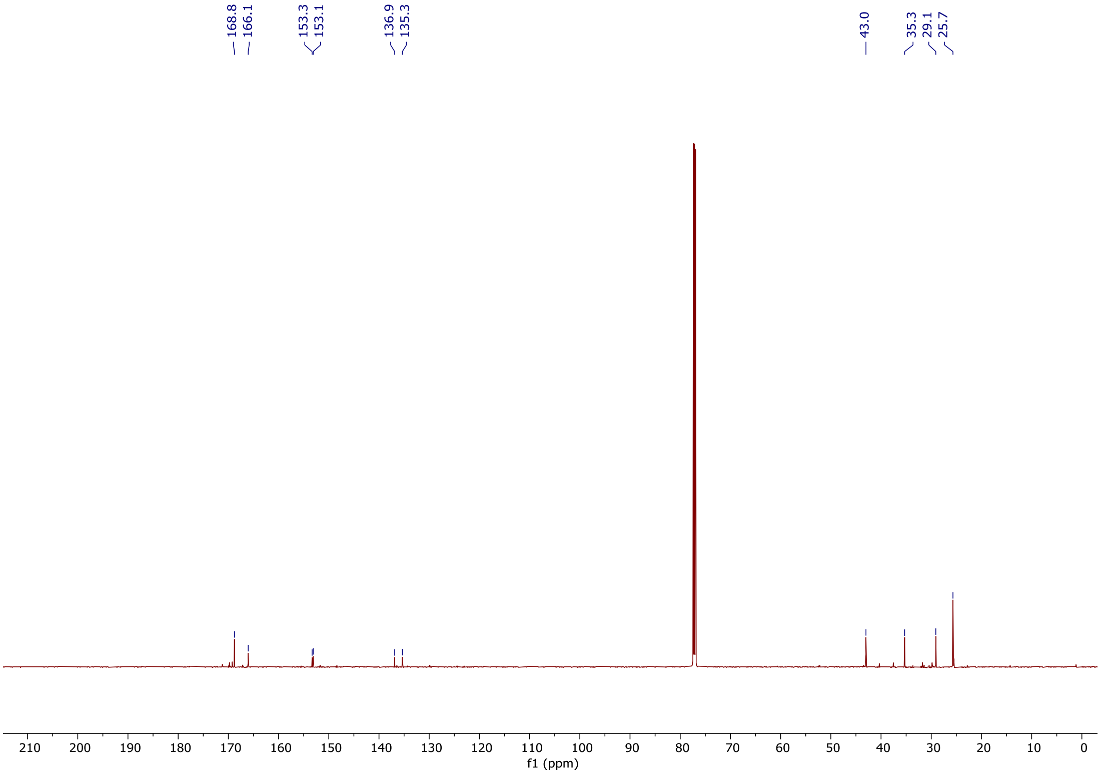
168.8
166.1
153.3
153.1
136.9
135.3
43.0
35.3
29.1
25.7
210 200 190 180 170 160 150 140 130 120 110 100 90 80 70 60 50 40 30 20 10 0
f1 (ppm)

((1*R*,8*S*,9*s*)-Bicyclo[6.1.0]non-4-yn-9-yl)methyl (2-(2-(2-(3-(4,5-dibromo-2-methyl-3,6- dioxo-3,6-dihydropyridazin-1(2*H*)-yl)propanamido)ethoxy)ethoxy)ethyl)carbamate (BCN PD) 1[2]

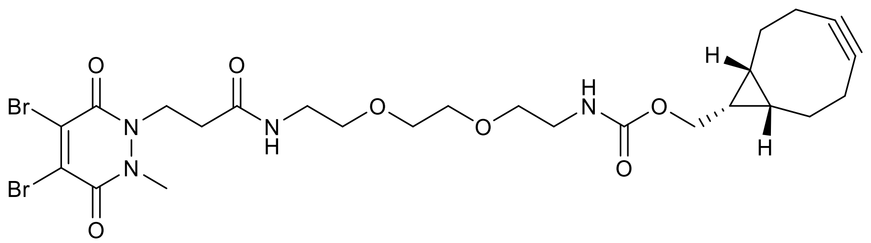

1

To a solution of 2,5-dioxopyrrolidin-1-yl 3-(4,5-dibromo-2-methyl-3,6-dioxo-3,6-dihydropyridazin-1(2*H*)-yl) propanoate **S4** (70 mg, 0.15 mmol) in dry MeCN (10 mL) was added *N*-[(1*R*,8*S*,9*s*)-bicyclo[6.1.0]non-4-yn-9-ylmethyloxycarbonyl]-1,8-diamino-3,6-dioxaoctane (50 mg, 0.15 mmol), and the reaction stirred at 21 °C for 18 h. After this time, the solvent was removed *in vacuo* and the crude residue dissolved in $CHCl_3$ (50 mL). The organic solution was then washed with water (3 × 30 mL), followed by saturated aq. K_2CO_3 (30 mL). The organic layer was then dried ($MgSO_4$) and concentrated *in vacuo.* Purification of the crude residue by flash column chromatography (0% to 10% MeOH/EtOAc) afforded ((1*R*,8*S*,9*s*)-bicyclo[6.1.0]non-4-yn-9-yl)methyl (2-(2-(2-(3-(4,5-dibromo-2-methyl-3,6-dioxo-3,6-dihydropyridazin-1(2*H*)-yl)propanamido)ethoxy)ethoxy)ethyl) carbamate **1** (51.2 mg, 0.08 mmol, 51%) as a yellow gum.

^{1}H NMR (700 MHz, $CDCl_3$, rotamers) δ 7.78 (s, 0.2H), 6.30 (s, 0.6H), 5.74 (s, 0.2H), 5.21 (s, 0.6H), 4.44 (t, *J* = 7.0 Hz, 2H), 4.15-4.14 (m, 2H), 3.72 (s, 3H), 3.64–3.36 (m, 12H), 2.62 (t, *J* = 6.9 Hz, 2H), 2.33–2.19 (m, 6H), 1.61–1.57* (m, 2H), 1.43–1.26 (m, 3H), 0.95 (d, *J* = 9.0 Hz, 2H).

^{13}C NMR (176 MHz, $CDCl_3$, rotamers) δ 169.0 (C), 157.0 (C), 153.1 (C), 153.0 (C), 136.4 (C), 135.5 (C), 98.9 (C), 70.4 (CH_2), 70.3 (CH_2), 69.7 (CH_2), 63.0 (CH_2), 44.6 (CH_2), 40.9 (CH_2), 39.5 (CH_2), 35.1 (CH_3), 34.1 (CH_2), 29.2 (CH_2), 21.6 (CH_2), 20.3 (CH_2), 17.9 (CH), 14.2 (CH).

IR (thin film) 3329, 2920, 2858, 1708, 1630, 1572, 1534 cm^{-1}.

*Behind water peak

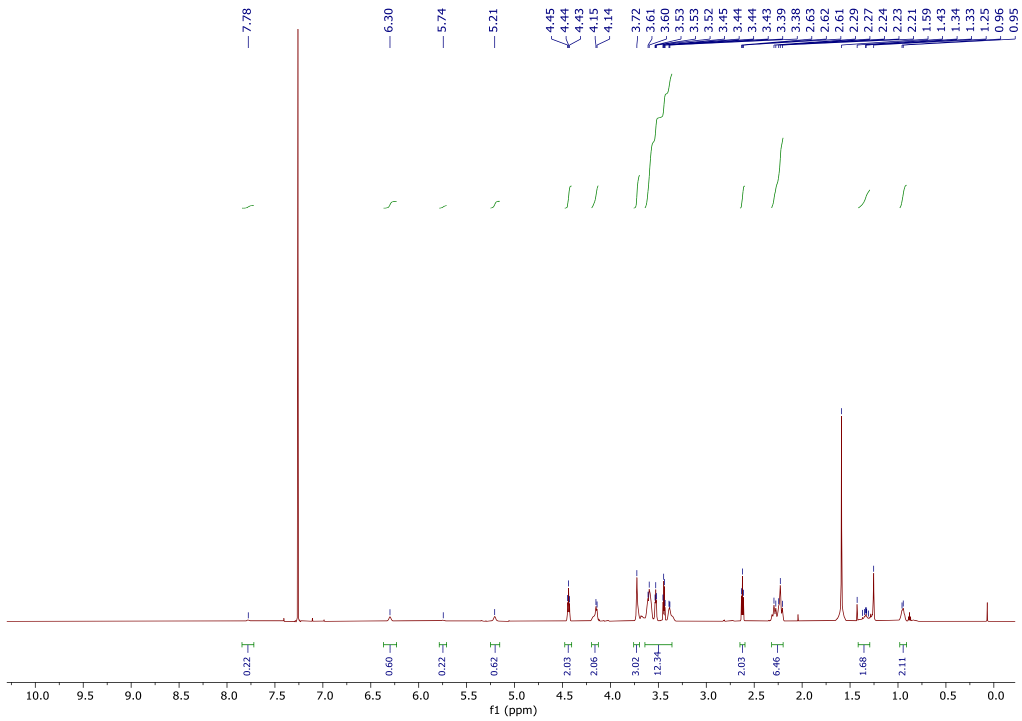
7.78
6.30
5.74
5.21
4.45
4.44
4.43
4.15
4.14
3.72
3.61
3.60
3.53
3.53
3.52
3.45
3.44
3.44
3.43
3.39
3.38
2.63
2.62
2.61
2.29
2.27
2.24
2.23
2.21
1.59
1.43
1.34
1.33
1.25
0.96
0.95
0.22
0.60
0.22
0.62
2.03
2.06
3.02
12.34
2.03
6.46
1.68
2.11
f1 (ppm)

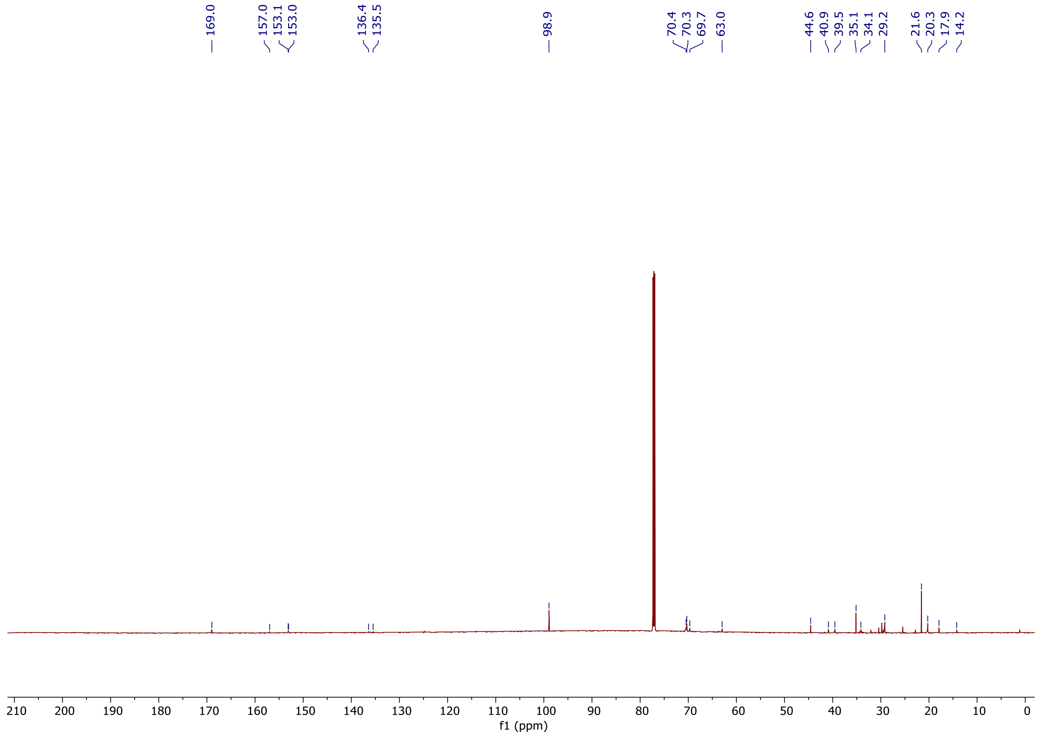
169.0
157.0
153.1
153.0
136.4
135.5
98.9
70.4
70.3
69.7
63.0
44.6
40.9
39.5
35.1
34.1
29.2
21.6
20.3
17.9
14.2
f1 (ppm)

Synthesis of ArN_3 bisPD 2

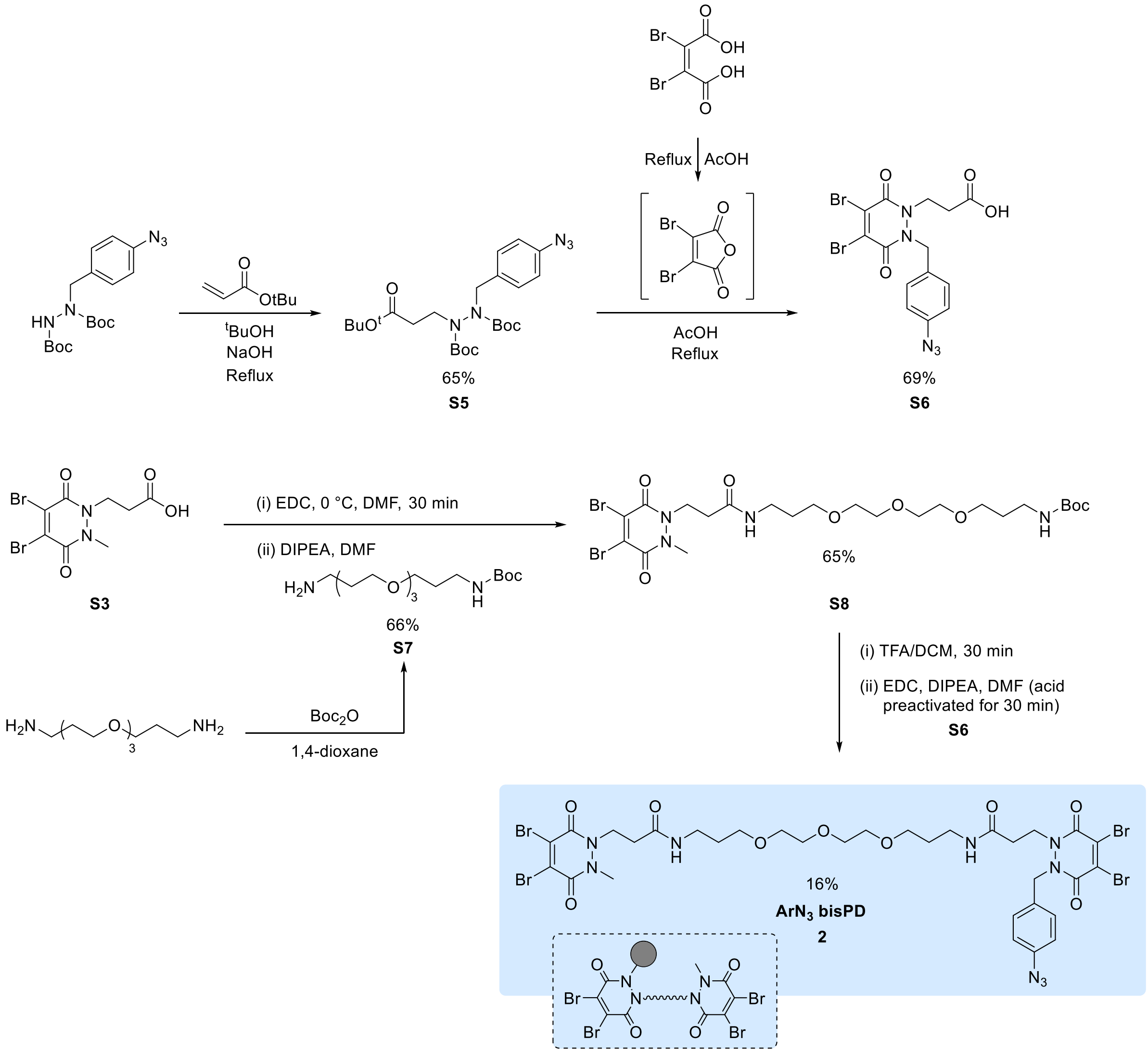

Figure S2. Synthetic Route to ArN_3 bisPD, **2**. Firstly, diBoc ArN_3 hydrazine (synthesised according to the literature)[4] is reacted with *tert*-butyl acrylate to form acid/ArN_3 hydrazine **S5**, which is refluxed with dibromomaleic anhydride to form the ArN_3/Acid PD **S6**. 4,7,10-trioxa-1,13-tridecanediamine is then Boc-protected to form **S7**, which is coupled to **S3** to form MetPAc PEG_3 Boc amine **S8**. The Boc group is then removed and **S8** coupled to **S6** to form ArN_3 bisPD **2**.

Di-*tert*-butyl 1-(4-azidobenzyl)-2-(3-(*tert*-butoxy)-3-oxopropyl)hydrazine-1,2-dicarboxylate S5[4]

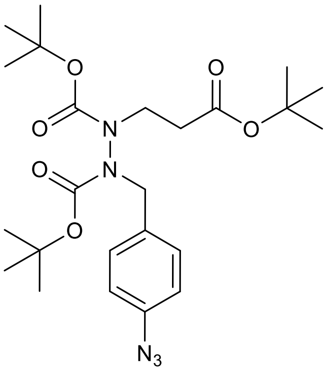

S5

To a solution of di-*tert*-butyl 1-(4-azidobenzyl)hydrazine-1,2-dicarboxylate (2 g, 5.51 mmol) in *t*BuOH (20 mL) was added 10% w/v NaOH (0.17 mL) and the solution stirred at 25 °C for 10 min. After this time, to the reaction mixture was added *tert*-butyl acrylate (1.60 mL, 11.02 mmol) and the solution stirred at 60 °C for 21.5 h. After this time, the solvent was removed *in vacuo* and the crude product dissolved in EtOAc (30 mL). The organic solution was then washed with water (5 × 100 mL) followed by brine (60 mL). The organic layer was then dried ($MgSO_4$) and concentrated *in vacuo.* The crude product was purified *via* flash column chromatography (0-40% EtOAc/cyclohexane) to afford di-*tert*-butyl 1-(4-azidobenzyl)-2-(3-(*tert*-butoxy)-3-oxopropyl)hydrazine-1,2-dicarboxylate **S5** (1.76 g, 3.58 mmol, 65%) as an orange oil.

^{1}H NMR (500 MHz, $CDCl_3$, rotamers) δ 7.31 (d, *J* = 8.5 Hz, 2H), 6.97 (d, *J* = 8.4 Hz, 2H), 4.90–4.19 (m, 2H), 3.60–3.29 (m, 2H), 2.52–1.94 (m, 2H), 1.48–1.31 (m, 27H).

^{13}C NMR (126 MHz, $CDCl_3$, rotamers) δ 171.0 (C), 155.0 (C), 154.6 (C), 139.6 (C), 133.7 (CH), 131.3 (CH), 131.0 (CH), 130.7 (CH), 119.1 (C), 81.6 (C), 81.2 (C), 80.8 (C), 54.1 (CH_2), 52.6 (CH_2), 52.1 (CH_2), 48.0 (CH_2), 46.2 (CH_2), 46.1 (CH_2), 34.5 (CH_3), 34.3 (CH_3), 34.0 (CH_3), 28.5 (CH_3), 28.4 (CH_3), 28.4 (CH_3), 28.3 (CH_3), 28.2 (CH_3), 28.2 (CH_3).

IR (thin film) 2977, 2934, 1705, 1518, 1453 cm^{-1}.

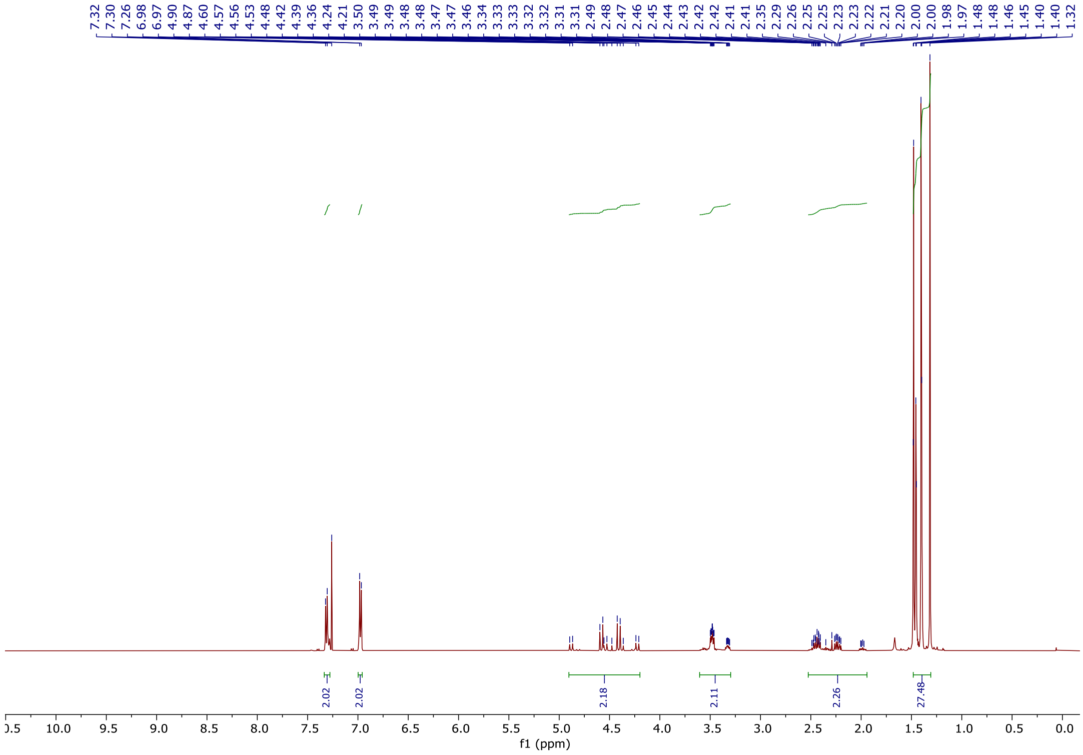
7.32
7.30
7.26
6.98
6.97
4.90
4.87
4.60
4.57
4.56
4.53
4.48
4.42
4.39
4.36
4.24
4.21
3.50
3.49
3.49
3.48
3.48
3.47
3.47
3.46
3.34
3.33
3.33
3.32
3.32
3.31
3.31
2.49
2.48
2.47
2.46
2.45
2.44
2.43
2.42
2.42
2.41
2.41
2.35
2.29
2.26
2.25
2.25
2.23
2.23
2.22
2.21
2.20
2.00
2.00
1.98
1.97
1.48
1.46
1.45
1.40
1.40
1.32
2.02
2.02
2.18
2.11
2.26
27.48
f1 (ppm)

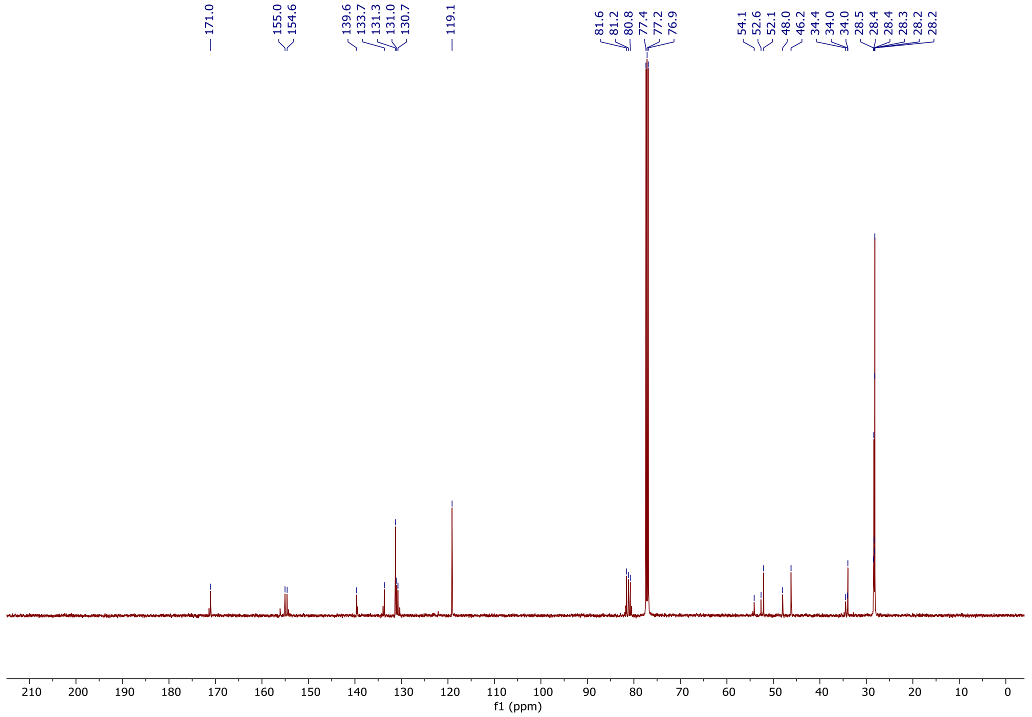
171.0
155.0
154.6
139.6
133.7
131.3
131.0
130.7
119.1
81.6
81.2
80.8
77.4
77.2
76.9
54.1
52.6
52.1
48.0
46.2
34.4
34.0
34.0
28.5
28.4
28.3
28.2
28.2
f1 (ppm)

3-(2-(4-Azidobenzyl)-4,5-dibromo-3,6-dioxo-3,6-dihydropyridazin-1(2*H*)-yl)propanoic acid S6[4]

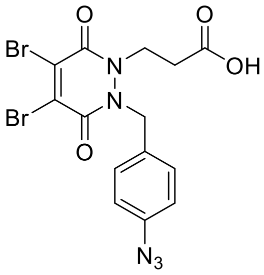

S6

Dibromomaleic acid (1.18 g, 4.30 mmol) in AcOH (35 mL) was heated under reflux for 30 min. After this time, to the reaction mixture, was added di-*tert*-butyl 1-(4-azidobenzyl)-2-(3-(*tert*-butoxy)-3-oxopropyl)hydrazine-1,2-dicarboxylate **S5** (1.76 g, 3.58 mmol), and the reaction stirred under reflux for 6 h. After this time, the solvent was removed *in vacuo* with toluene co-evaporation (3 × 30 mL, as an azeotrope) and then chloroform co-evaporation (3 × 30 mL). The crude residue was purified by flash column chromatography (20-80% EtOAc/cyclohexane, 1% AcOH) to give 3-(2-(4-azidobenzyl)-4,5-dibromo-3,6-dioxo-3,6-dihydropyridazin-1(2*H*)-yl)propanoic acid **S6** (1.17 g, 2.47 mmol, 69%) as a yellow solid.

^{1}H NMR (500 MHz, $CDCl_3$) δ 7.23 (d, *J* = 8.5 Hz, 2H), 7.03 (d, *J* = 8.5 Hz, 2H), 5.34 (s, 2H), 4.28 (t, *J* = 7.1 Hz, 2H), 2.62 (t, *J* = 7.1 Hz, 2H).

^{13}C NMR (126 MHz, $CDCl_3$) δ 173.7 (C), 154.2 (C), 153.5 (C), 140.9 (C), 136.3 (C), 136.3 (C), 130.7 (CH), 128.9 (CH), 120.1 (C), 50.8 (CH_2), 43.3 (CH_2), 31.2 (CH_2).

IR (solid) 3117, 2962, 2913, 2115, 1713, 1628, 1572, 1507 cm^{-1}.

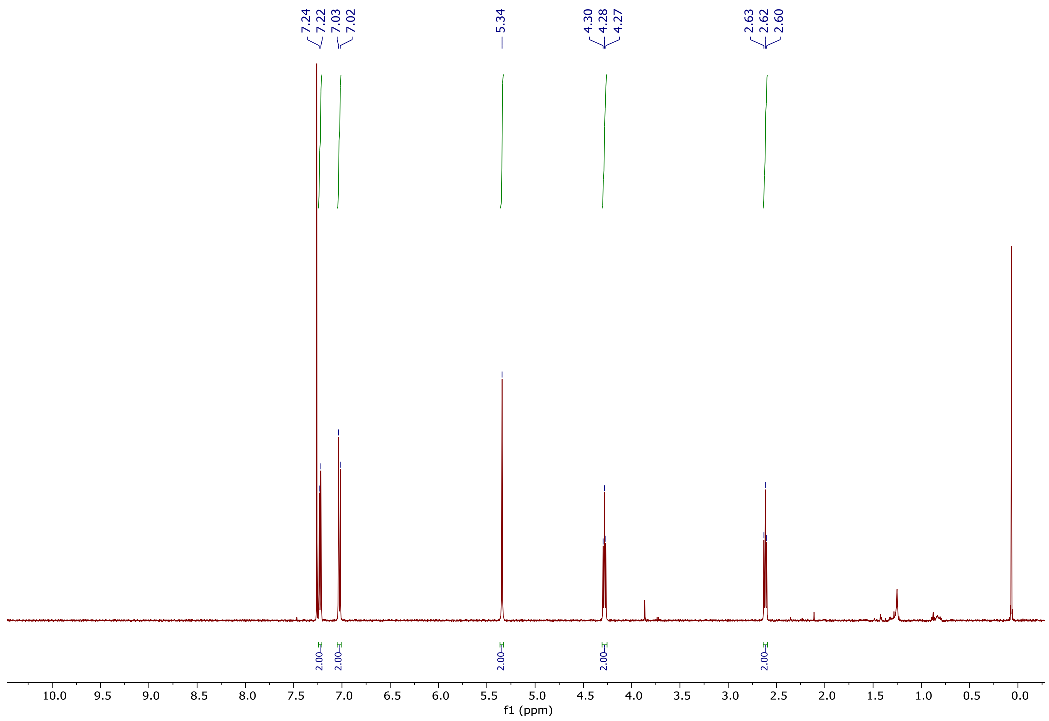
7.24
7.22
7.03
7.02
5.34
4.30
4.28
4.27
2.63
2.62
2.60
2.00
2.00
2.00
2.00
2.00
10.0 9.5 9.0 8.5 8.0 7.5 7.0 6.5 6.0 5.5 5.0 4.5 4.0 3.5 3.0 2.5 2.0 1.5 1.0 0.5 0.0
f1 (ppm)

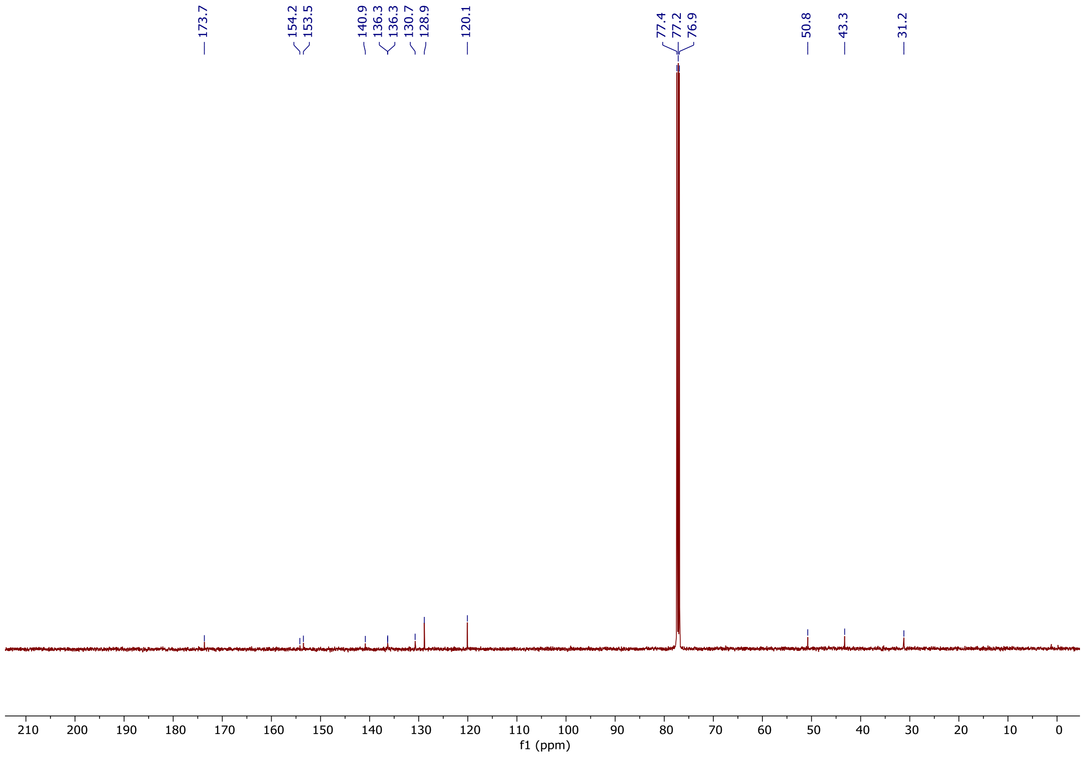
173.7
154.2
153.5
140.9
136.3
136.3
130.7
128.9
120.1
77.4
77.2
76.9
50.8
43.3
31.2
210 200 190 180 170 160 150 140 130 120 110 100 90 80 70 60 50 40 30 20 10 0
f1 (ppm)

Tert-Butyl (3-(2-(2-(3-aminopropoxy)ethoxy)ethoxy)propyl)carbamate S7

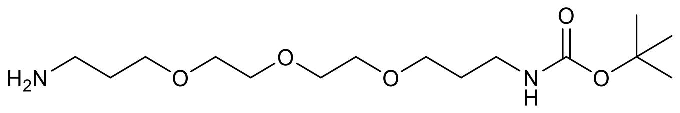

S7

To a stirring solution of 3,3'-((oxybis(ethane-2,1-diyl))bis(oxy))bis(propan-1-amine) (4.05 g, 18.4 mmol) in 1,4-dioxane (30 mL) was added dropwise di-*tert*-butyl dicarbonate (0.5 g, 2.3 mmol, pre-dissolved in 1,4-dioxane (12.5 mL) over 2 h. After this time, the reaction mixture was stirred at 25 °C for a further 45 mins. Following this, the reaction mixture was concentrated *in vacuo*, the crude residue dissolved in water (30 mL), and the organics extracted into EtOAc (5 × 15 mL). The organics were combined, dried ($MgSO_4$) and concentrated *in vacuo* to give *tert*-butyl (3-(2-(2-(3-aminopropoxy)ethoxy)ethoxy)propyl)carbamate **S7** (502 mg, 2.36 mmol, 68%) as a clear yellow oil.

^{1}H NMR (500 MHz, $CDCl_3$) δ 5.10 (s, 0.5H*), 3.63 (dt, *J* = 5.2, 2.8 Hz, 4H), 3.60–3.48 (m, 8H), 3.21 (t, *J* = 6.3 Hz, 2H), 2.79 (t, *J* = 6.7 Hz, 1H), 1.73 (dt, *J* = 11.3, 6.4 Hz, 4H), 1.42 (s, 9H).

^{13}C NMR (151 MHz, $CDCl_3$) δ 156.2 (C), 79.0 (C), 70.7 (CH_2), 70.7 (CH_2), 70.3 (CH_2), 70.3 (CH_2), 69.6 (CH_2), 39.7 (CH_2), 38.6 (CH_2), 33.1 (CH_2), 29.7 (CH_2), 28.6 (CH_3).

IR (thin film) 3353, 2923, 2865, 1694, 1563, 1516 cm^{-1}.

* Less than 1H potentially due to partial D-H exchange of the acidic NH amide proton in the NMR sample.

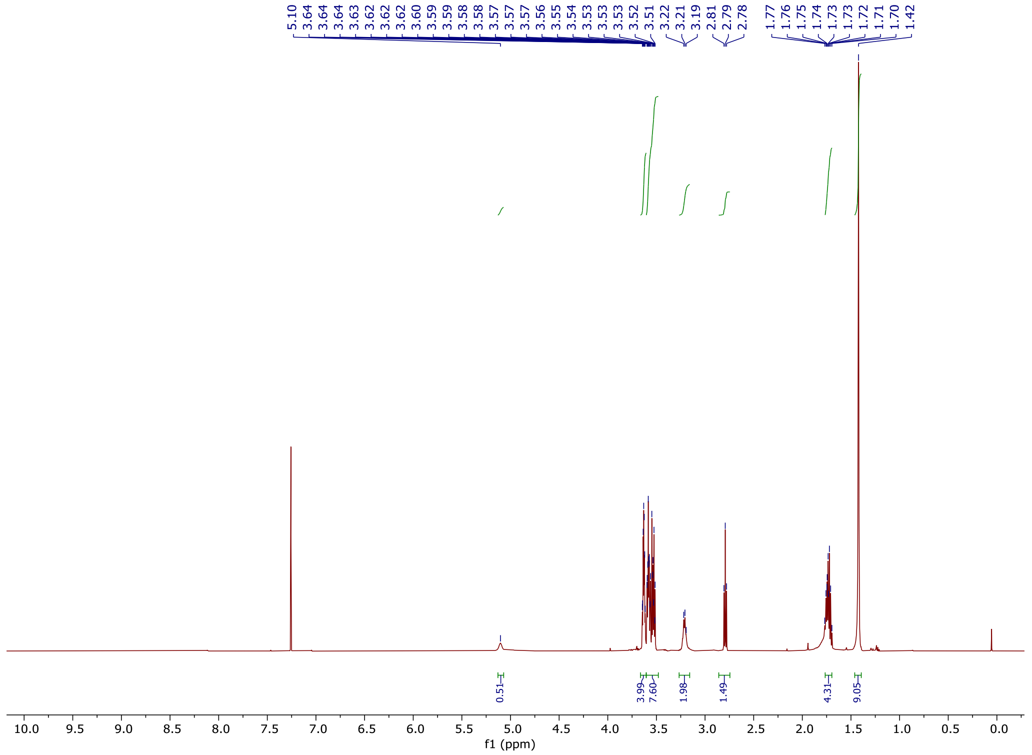
5.10
3.64
3.64
3.64
3.63
3.62
3.62
3.62
3.60
3.59
3.59
3.58
3.58
3.57
3.57
3.57
3.56
3.55
3.54
3.53
3.53
3.53
3.52
3.51
3.22
3.21
3.19
2.81
2.79
2.78
1.77
1.76
1.75
1.74
1.73
1.73
1.72
1.71
1.70
1.42
0.51
3.99
7.60
1.98
1.49
4.31
9.05
f1 (ppm)

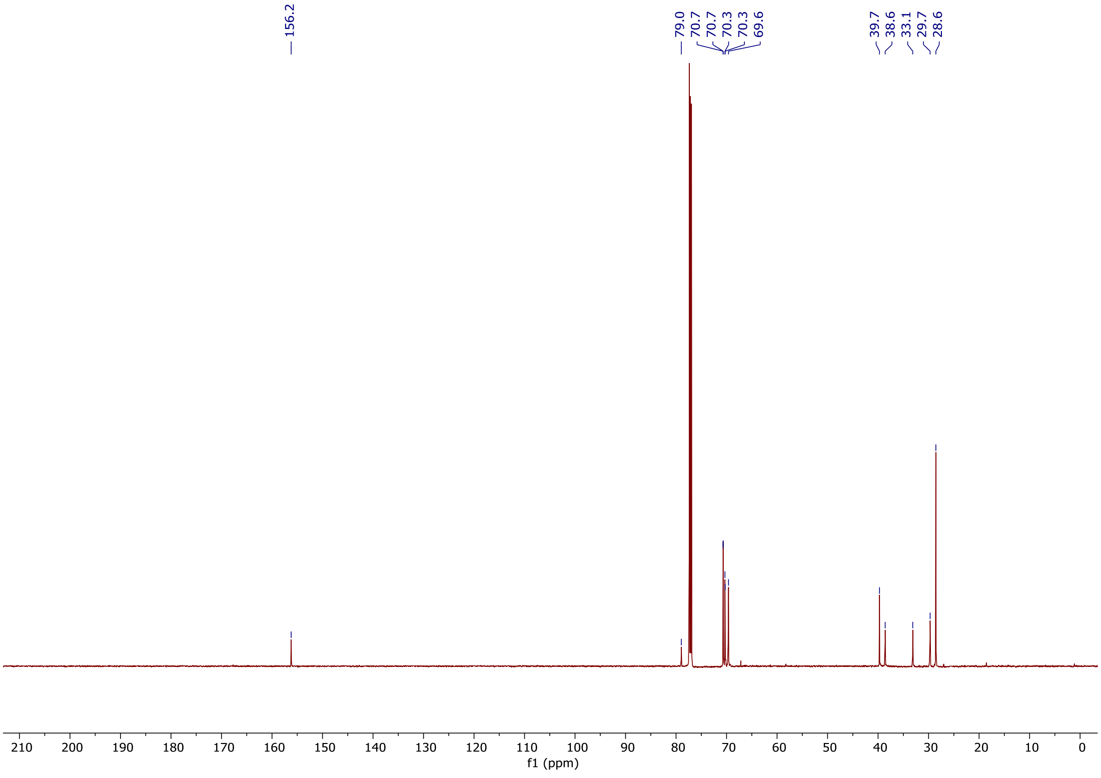
156.2
79.0
70.7
70.7
70.3
70.3
69.6
39.7
38.6
33.1
29.7
28.6
f1 (ppm)

Tert-Butyl (17-(4,5-dibromo-2-methyl-3,6-dioxo-3,6-dihydropyridazin-1(2*H*)-yl)-15-oxo-4,7,10-trioxa-14-azaheptadecyl)carbamate S8 [2]

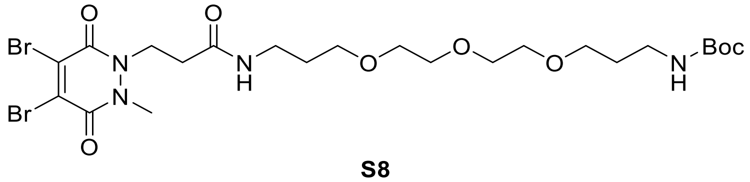

S8

To a pre-cooled to 0 °C solution of 3-(4,5-dibromo-2-methyl-3,6-dioxo-3,6-dihydropyridazin-1(2*H*)-yl) propanoic acid **S3** (850 mg, 2.39 mmol) in anhydrous DMF (20 mL), was added EDC.HCl (458 mg, 2.39 mmol), and the reaction stirred under an inert atmosphere for 30 min. After this time, to the reaction mixture was added (3-(2-(2-(3- aminopropoxy)ethoxy)ethoxy)propyl)carbamate (696 mg, 2.17 mmol) **S7**, and the reaction allowed to warm to 25 °C and then stirred for 17 h. After this time, the solvent was removed in *vacuo* and the crude product diluted with water (100 mL). The organics were then extracted into EtOAc (4 × 50 mL). The organics were combined and washed with sat. aq. LiCl (100 mL), brine (70 mL), dried ($MgSO_4$) and then concentrated *in vacuo*. The crude product was purified *via* flash column chromatography (0-10% MeOH/EtOAc) to give *tert*-butyl (17-(4,5-dibromo-2-methyl-3,6-dioxo-3,6-dihydropyridazin-1(2*H*)-yl)-15-oxo-4,7,10-trioxa-14-azaheptadecyl)carbamate **S8** (926 mg, 1.41 mmol, 65%) as a yellow oil.

^{1}H NMR (600 MHz, $CDCl_3$) δ 6.80 (s, 0.8H*), 4.99 (s, 0.9H*), 4.43 (t, *J* = 6.8 Hz, 2H), 3.72 (s, 3H), 3.64–3.60 (m, 4H), 3.58–3.50 (m, 8H), 3.35 (q, *J* = 5.8 Hz, 2H), 3.19 (s, 2H), 2.55 (t, *J* = 6.9 Hz, 2H), 1.76–1.69 (m, 4H), 1.42 (s, 9H).

^{13}C NMR (126 MHz, $CDCl_3$) δ 169.1 (C), 156.3 (C), 153.2 (C), 152.9 (C), 136.4 (C), 135.5 (C), 79.3 (C) 70.6 (CH_2), 70.4 (CH_2), 70.3 (CH_2), 70.1 (CH_2), 69.5 (CH_2), 44.5 (CH_2), 38.7 (CH_2), 35.2 (CH_2), 34.2 (CH_2), 29.8 (CH_3), 28.6 (CH_3), 28.6 (CH_3).

IR (thin film) 3507, 3497, 3334, 2921, 2869, 1692, 1627, 1571, 1529 cm^{-1}.

* Less than 1H potentially due to partial D-H exchange of the acidic NH amide proton in the NMR sample.

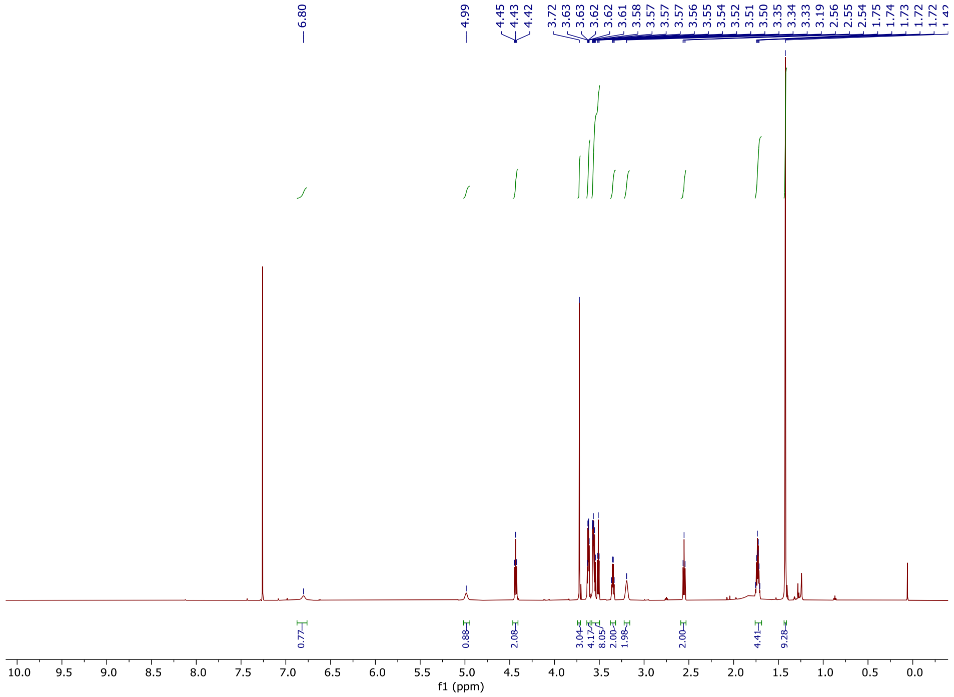
6.80
4.99
4.45
4.43
4.42
3.72
3.63
3.63
3.62
3.62
3.61
3.58
3.57
3.57
3.57
3.56
3.55
3.54
3.52
3.51
3.50
3.35
3.34
3.33
3.19
2.56
2.55
2.54
1.75
1.74
1.73
1.72
1.72
0.77
0.88
2.08
3.04
4.17
8.05
2.00
1.98
2.00
4.41
9.28
10.0 9.5 9.0 8.5 8.0 7.5 7.0 6.5 6.0 5.5 5.0 4.5 4.0 3.5 3.0 2.5 2.0 1.5 1.0 0.5 0.0
f1 (ppm)

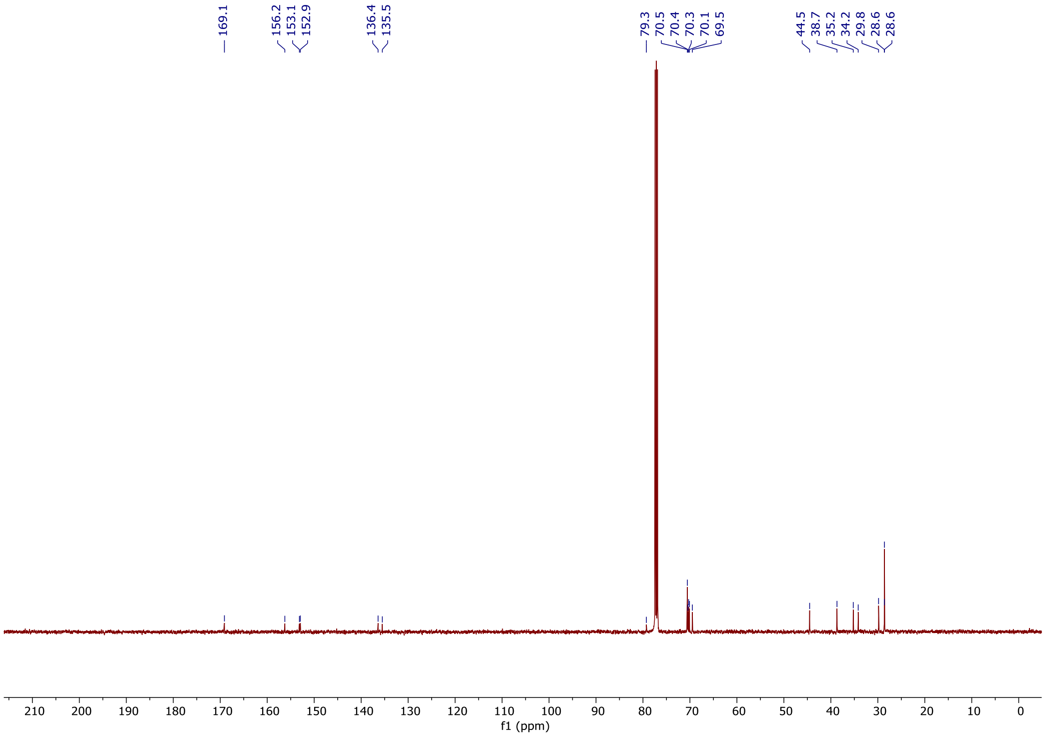
169.1
156.2
153.1
152.9
136.4
135.5
79.3
70.5
70.4
70.3
70.1
69.5
44.5
38.7
35.2
34.2
29.8
28.6
28.6
210 200 190 180 170 160 150 140 130 120 110 100 90 80 70 60 50 40 30 20 10 0
f1 (ppm)

3-(2-(4-Azidobenzyl)-4,5-dibromo-3,6-dioxo-3,6-dihydropyridazin-1(2*H*)-yl)-*N*-(17-(4,5-dibromo-2-methyl-3,6-dioxo-3,6-dihydropyridazin-1(2*H*)-yl)-15-oxo-4,7,10-trioxa-14-azaheptadecyl)propanamide (ArN$_3$ bisPD) 2

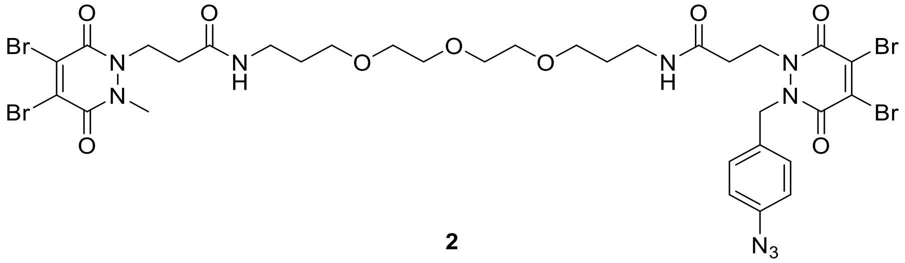

2

To a solution of *tert*-butyl (17-(4,5-dibromo-2-methyl-3,6-dioxo-3,6-dihydropyridazin-1(2*H*)-yl)-15-oxo-4,7,10-trioxa-14-azaheptadecyl)carbamate (305 mg, 0.46 mmol) in DCM (5 mL) was added TFA (5 mL) and the reaction stirred at 25 °C for 30 min. After this time, the solvent was removed *in vacuo* with DCM co-evaporation (5 × 5 mL). The deprotected amine was dissolved in DMF (3 mL) and stirred with DIPEA (200 µL). This was added dropwise to a solution of 3-(2-(4-azidobenzyl)-4,5-dibromo-3,6-dioxo-3,6-dihydropyridazin-1(2*H*)-yl)propanoic acid (240 mg, 0.51 mmol), pre-cooled to 0 °C, which had been stirred at 0 °C with EDC.HCl (98 mg, 0.51 mmol) under an inert atmosphere in dry DMF (6 mL) for 30 min. The reaction was allowed to warm to room temperature and stirred for 30 h. After this time, the reaction mix was diluted with water (100 mL) and the product extracted with DCM (4 × 50 mL). The organic layers were combined, washed with sat. aq. LiCl (100 mL) and dried ($MgSO_4$). The solvent was removed *in vacuo*. The crude product was purified by flash column chromatography (0-30% MeOH/EtOAc, 1% AcOH) to give 3-(2-(4-azidobenzyl)-4,5-dibromo-3,6-dioxo-3,6-dihydropyridazin-1(2*H*)-yl)-*N*-(17-(4,5-dibromo-2-methyl-3,6-dioxo-3,6-dihydropyridazin-1(2*H*)-yl)-15-oxo-4,7,10-trioxa-14-azaheptadecyl)propanamide **2** (76 mg, 0.07 mmol, 16%) as a yellow solid.

^{1}H NMR (500 MHz, $CDCl_3$) δ 7.27 (d, *J* = 8.2 Hz, 2H), 7.01 (d, *J* = 8.2 Hz, 2H), 5.48 (s, 2H), 4.42 (t, *J* = 6.9 Hz, 2H), 4.30 (t, *J* = 6.8 Hz, 2H), 3.72 (s, 3H), 3.60 (dd, *J* = 6.2, 3.4 Hz, 4H), 3.56–3.49 (m, 8H), 3.37–3.26 (m, 4H), 2.54 (dt, *J* = 33.4, 6.9 Hz, 4H), 1.74 (m, 4H).

^{13}C NMR (126 MHz, $CDCl_3$) δ 169.3 (C), 169.2 (C), 153.8 (C), 153.2 (C), 153.1 (C), 153.0 (C), 140.5 (C), 136.5 (C), 136.3 (C), 136.1 (C), 131.1 (CH), 129.0 (CH), 119.9 (C), 70.4 (CH_2), 70.1 (CH_2), 70.0 (CH_2), 70.0 (CH_2), 49.9 (CH_2), 44.6 (CH_2), 44.3 (CH_2), 38.3 (CH_2), 38.2 (CH_2), 35.2 (CH_2), 34.2 (CH_2), 34.1 (CH_2), 28.8 (CH_3).

IR (solid) 3326, 2923, 2856, 2110, 1626, 1573, 1548, 1506 cm^{-1}.

LRMS (ESI) 1018 (20, [$M^{81}Br_4$+H]$^+$), 1016 (70, [$M^{81}Br_3{}^{79}Br$+H]$^+$) 1014 (100, [$M^{81}Br_2{}^{79}Br_2$+H]$^+$), 1012 (70, [$M^{81}Br^{79}Br_3$+H]$^+$) 1010 (20, [$M^{79}Br_3$+H]$^+$); **HRMS (ESI)** calcd for $C_{32}H_{40}O_9N_9Br_4$ [$M^{81}Br_2{}^{79}Br_2$+H]$^+$ 1014.3309; observed 1013.9654.

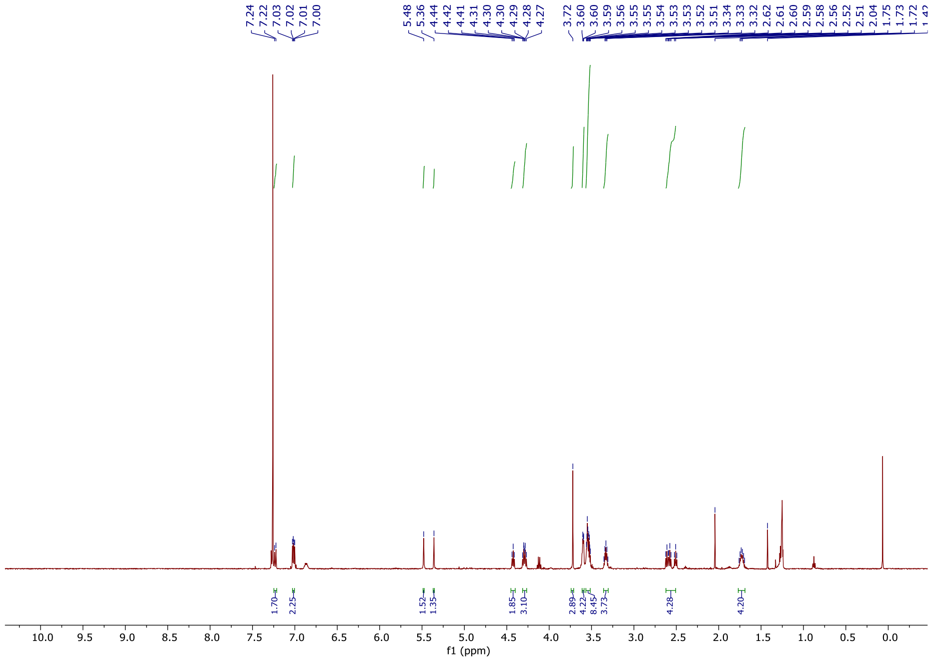
7.24
7.22
7.03
7.02
7.01
7.00
5.48
5.36
4.44
4.42
4.41
4.31
4.30
4.29
4.28
4.27
3.72
3.60
3.60
3.59
3.56
3.55
3.55
3.54
3.53
3.53
3.52
3.51
3.34
3.33
3.32
2.62
2.61
2.60
2.59
2.58
2.56
2.52
2.51
2.04
1.75
1.73
1.72
1.70
2.25
1.52
1.35
1.85
3.10
2.89
4.22
8.45
3.73
4.28
4.20
f1 (ppm)

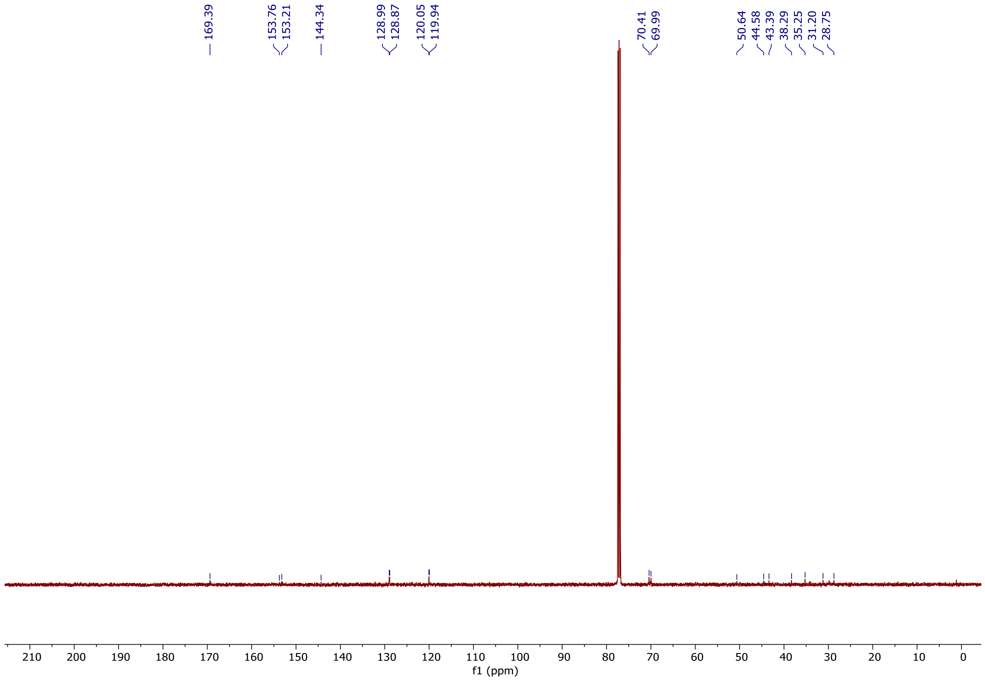
169.39
153.76
153.21
144.34
128.99
128.87
120.05
119.94
70.41
69.99
50.64
44.58
43.39
38.29
35.25
31.20
28.75
f1 (ppm)

Synthesis of N_3 Aniline 3

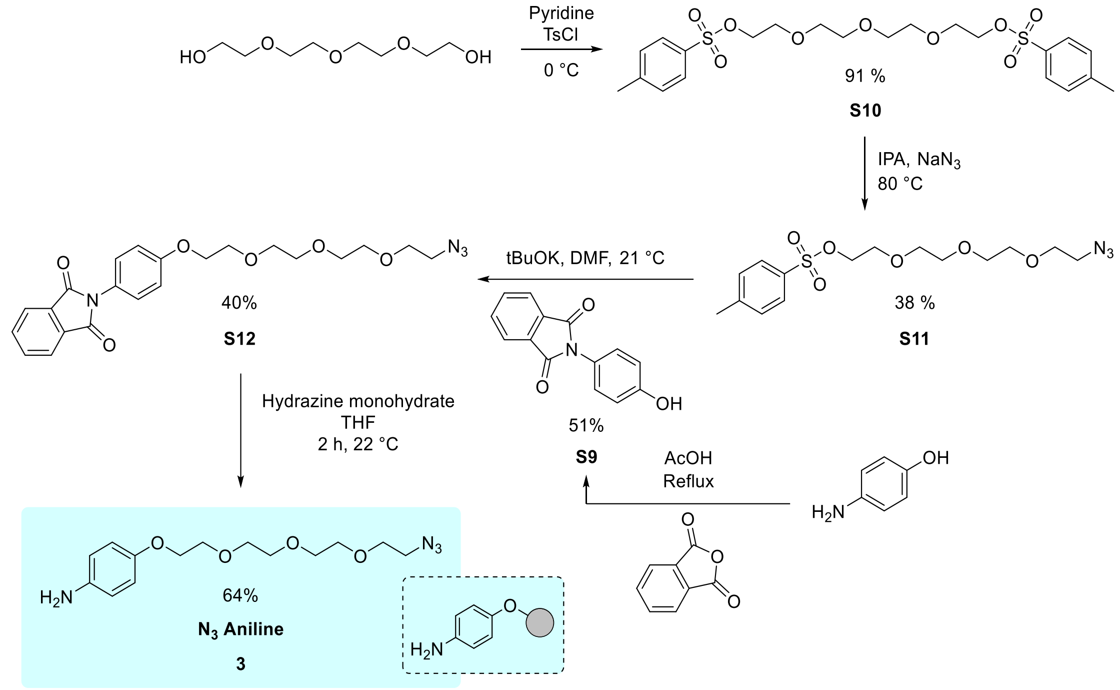

Figure S3. Synthetic route to N_3 aniline **3**. *para*-Aminophenol is protected with phthalic anhydride to form protected *p*-aminophenol **S9.** Tetraethylene glycol is tosylated to form bistosyl tetraethylene glycol **S10**. One of the tosyl groups of **S10** is then displaced with NaN_3 to form tosyl-PEG_3-N_3 **S11**. **S11** is then reacted with **S9** to form phthalimide-protected PEG_3 N_3 **S12**, which is deprotected to form N_3 aniline **3**.

2-(4-Hydroxyphenyl)isoindoline-1,3-dione S9 [3]

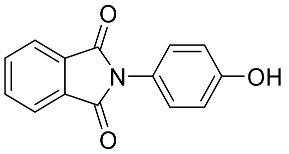

S9

To a solution of phthalic anhydride (1 g, 6.75 mmol), pre-dissolved in AcOH (40 mL) was added 4-aminophenol (737 mg, 6.75 mmol). The mixture was heated under reflux for 16 h. After cooling to room temperature, the precipitate was filtered and washed with MeOH (50 mL). The solvent was removed *in vacuo* with toluene (3 × 30 mL, as an azeotrope) then chloroform (3 × 30 mL) co-evaporation to afford 2-(4-hydroxyphenyl)isoindoline-1,3-dione **S9** (826 mg, 3.45 mmol, 51%) as a white solid.

^{1}H NMR (600 MHz, DMSO) δ 9.80 (br s, 1H), 7.94 (dd, *J* = 5.4, 3.0 Hz, 2H), 7.89 (dd, *J* = 5.4, 3.1 Hz, 2H), 7.20 (d, *J* = 8.7 Hz, 2H), 6.87 (d, *J* = 8.7 Hz, 2H).

^{13}C NMR (151 MHz, DMSO) δ 167.5 (C), 157.3 (C), 134.7 (CH), 131.6 (C), 128.9 (CH), 123.4 (CH), 122.9 (C), 115.3 (C).

IR (solid) 3410, 3035, 2923, 2852, 1707, 1610, 1595, 1580, 1514 cm^{-1}.

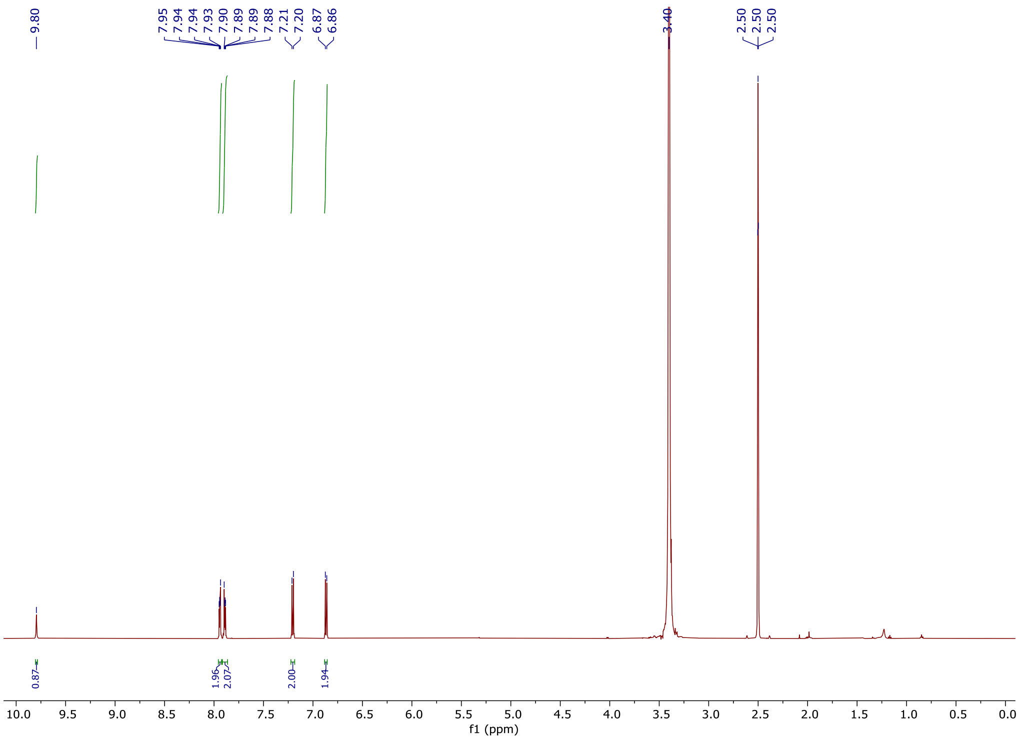

9.80
7.95
7.94
7.94
7.93
7.90
7.89
7.89
7.88
7.21
7.20
6.87
6.86
3.40
2.50
2.50
2.50
0.87
1.96
2.07
2.00
1.94
f1 (ppm)

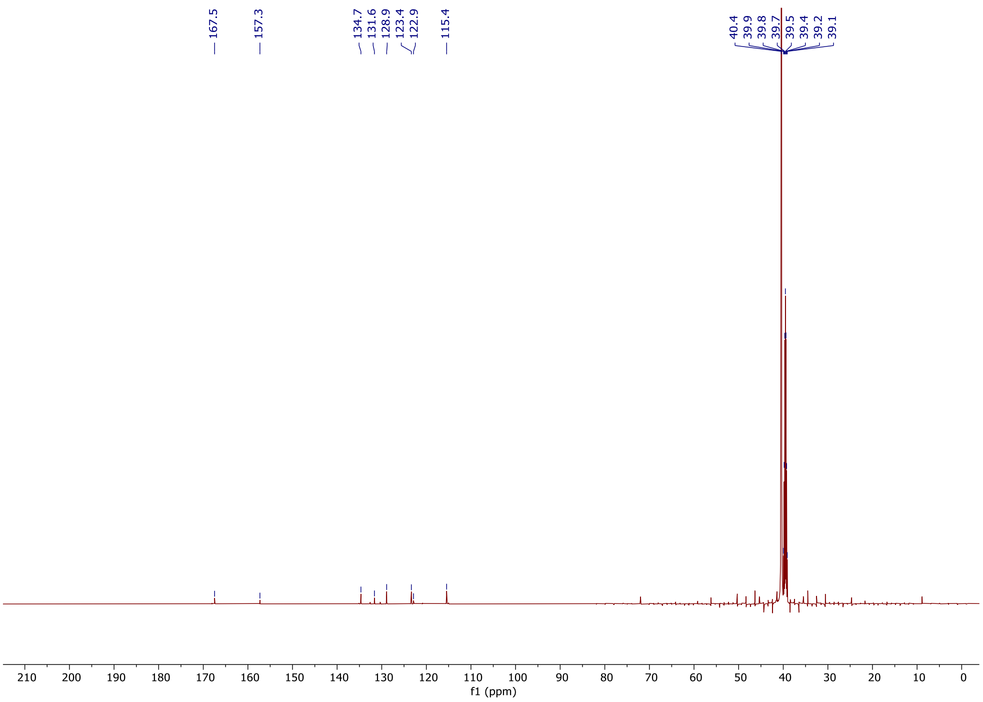

167.5
157.3
134.7
131.6
128.9
123.4
122.9
115.4
40.4
39.9
39.8
39.7
39.5
39.4
39.2
39.1
f1 (ppm)

((Oxybis(ethane-2,1-diyl))bis(oxy))bis(ethane-2,1-diyl) bis(4-methylbenzenesulfonate) S10[3]

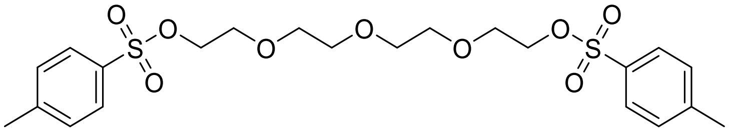

S10

To a stirred solution of tetraethylene glycol (1.08 g, 5.56 mmol) in pyridine (3.6 mL) at 0 °C was added dropwise over 30 min tosyl chloride (2.33 g, 12.23 mmol) pre-dissolved in DCM (5 mL). The mixture was stirred at 0 °C for 4 h. After this time, the reaction was incomplete by TLC so more tosyl chloride (1.17 g, 6.12 mmol), pre-dissolved in DCM (2 mL) was added and the reaction stirred at 0 °C for a further 2 h. Ice water (30 mL) was added to the reaction mixture and the crude product extracted with EtOAc (3 × 30 mL). The organic layer was washed with 2 M HCl (2 × 13 mL) and dried ($MgSO_4$). The solvent was removed *in vacuo* to afford ((oxybis(ethane-2,1-diyl))bis(oxy))bis(ethane-2,1-diyl) bis(4-methylbenzenesulfonate) **S10** (2.54 g, 5.05 mmol, 91%) as a clear pink oil.

^{1}H NMR (600 MHz, $CDCl_3$) δ 7.72 (d, *J* = 8.3 Hz, 4H), 7.27 (d, *J* = 8.1 Hz, 4H), 4.08 (t, *J* = 4.7 Hz, 4H), 3.60 (t, *J* = 5.0 Hz, 4H), 3.51–3.47 (m, 8H), 2.37 (s, 6H).

^{13}C NMR (151 MHz, $CDCl_3$) δ 145.0 (C), 133.0 (C), 129.9 (CH), 128.1 (CH), 70.8 (CH_2), 70.6 (CH_2), 69.4 (CH_2), 68.8 (CH_2), 21.8 (CH_3).

IR (thin film) 2947, 2872, 1597 cm^{-1}.

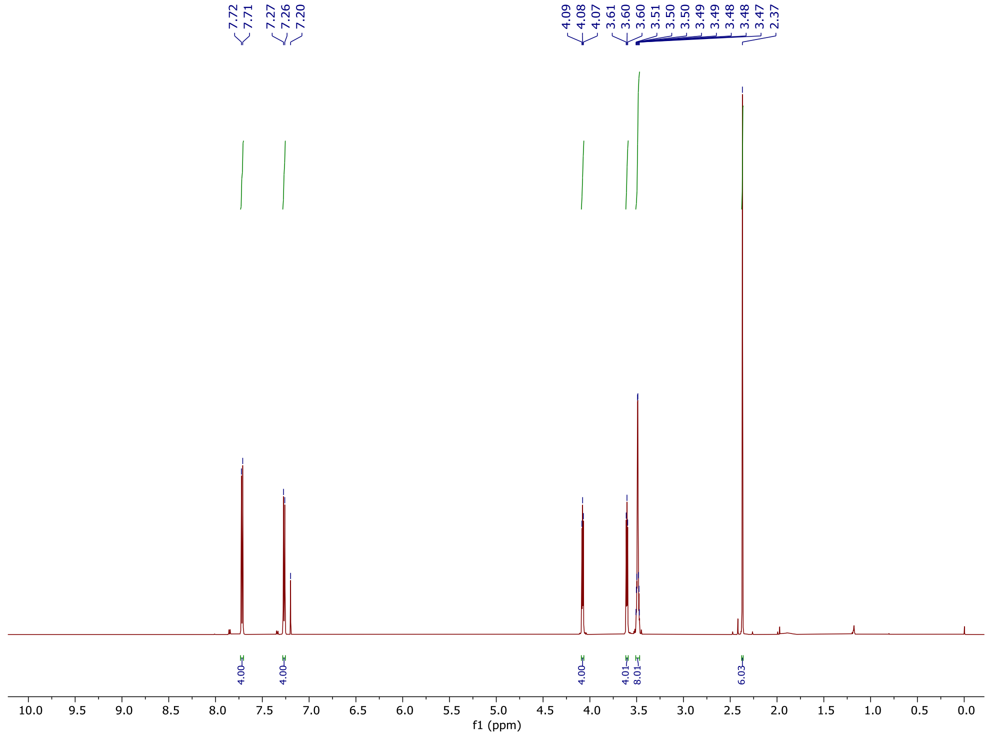
7.72
7.71
7.27
7.26
7.20
4.09
4.08
4.07
3.61
3.60
3.60
3.51
3.50
3.50
3.49
3.49
3.48
3.48
3.47
2.37
4.00
4.00
4.00
4.01
8.01
6.03
10.0 9.5 9.0 8.5 8.0 7.5 7.0 6.5 6.0 5.5 5.0 4.5 4.0 3.5 3.0 2.5 2.0 1.5 1.0 0.5 0.0
f1 (ppm)

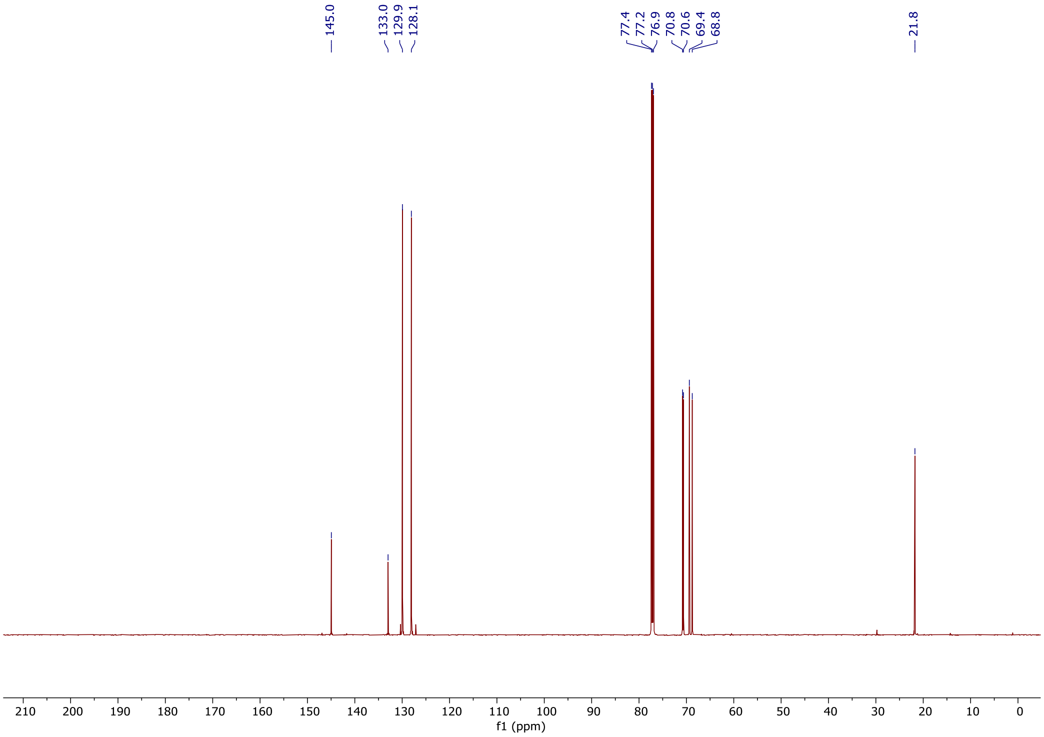
145.0
133.0
129.9
128.1
77.4
77.2
76.9
70.8
70.6
69.4
68.8
21.8
210 200 190 180 170 160 150 140 130 120 110 100 90 80 70 60 50 40 30 20 10 0
f1 (ppm)

2-(2-(2-(2-Azidoethoxy)ethoxy)ethoxy)ethyl 4-methylbenzenesulfonate S11[3]

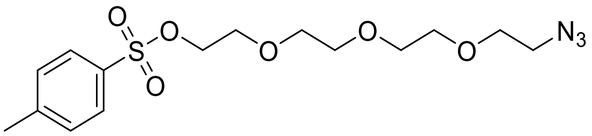

S11

To a solution of ((oxybis(ethane-2,1-diyl))bis(oxy))bis(ethane-2,1-diyl) bis(4-methylbenzenesulfonate) **S10** (1 g, 1.99 mmol) in IPA (20 mL) was added sodium azide (142 mg, 2.19 mmol). The resulting solution was heated to 60 °C for 20 h and then to 80 °C for a further 10 h. After this time, the reaction was poured into ice water (50 mL) and the product extracted with EtOAc (3 × 50 mL). The organic extracts were combined and washed with water (50 mL), then brine (50 mL), dried ($MgSO_4$) and the solvent removed *in vacuo*. The crude product was purified *via* flash column chromatography (30-80% EtOAc/cyclohexane) to give 2-(2-(2-(2-azidoethoxy)ethoxy)ethoxy)ethyl 4-methylbenzenesulfonate **S11** (282 mg, 0.76 mmol, 38%) as a clear oil.

^{1}H NMR (500 MHz, $CDCl_3$) δ 7.79 (d, *J* = 8.3 Hz, 2H), 7.34 (d, *J* = 8.0 Hz, 2H), 4.17–4.14 (m, 2H), 3.70–3.59 (m, 12H), 3.38 (t, *J* = 5.0 Hz, 2H), 2.44 (s, 3H).

^{13}C NMR (126 MHz, $CDCl_3$) δ 144.9, 133.1, 130.0, 128.1, 77.4, 77.4, 77.2, 76.9, 70.9, 70.8, 70.7, 70.2, 69.4, 68.8, 50.8, 21.8.

IR (thin film) 2915, 2869, 2099, 1598 cm^{-1}.

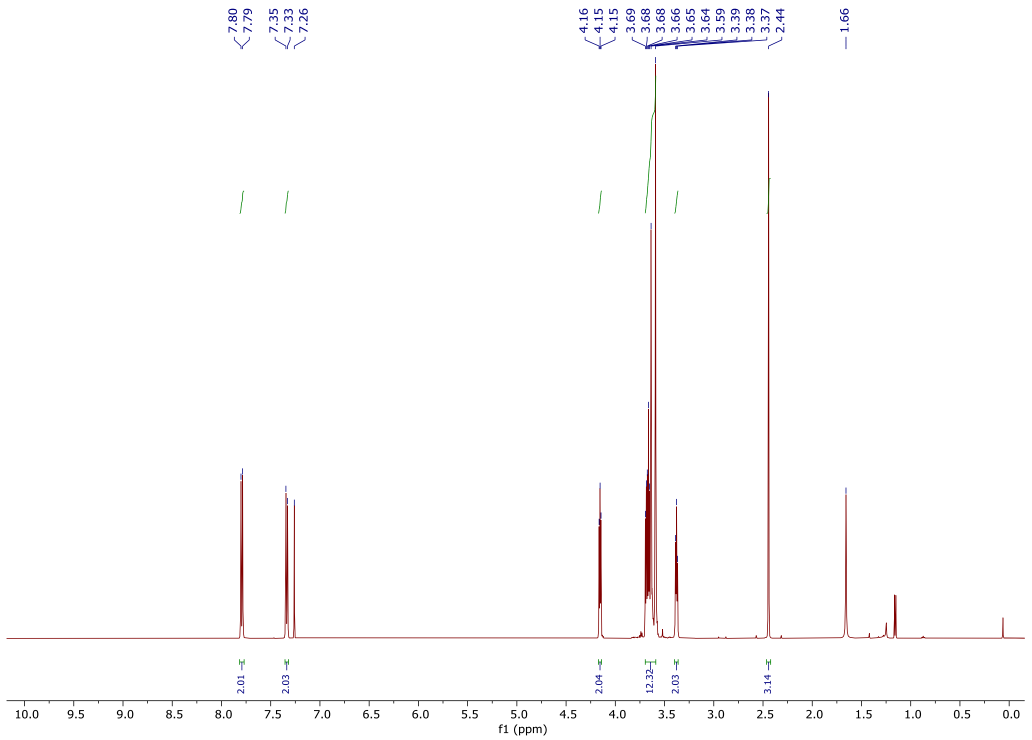
7.80
7.79
7.35
7.33
7.26
4.16
4.15
4.15
3.69
3.68
3.68
3.66
3.65
3.64
3.59
3.39
3.38
3.37
2.44
1.66
2.01
2.03
2.04
12.32
2.03
3.14
10.0 9.5 9.0 8.5 8.0 7.5 7.0 6.5 6.0 5.5 5.0 4.5 4.0 3.5 3.0 2.5 2.0 1.5 1.0 0.5 0.0
f1 (ppm)

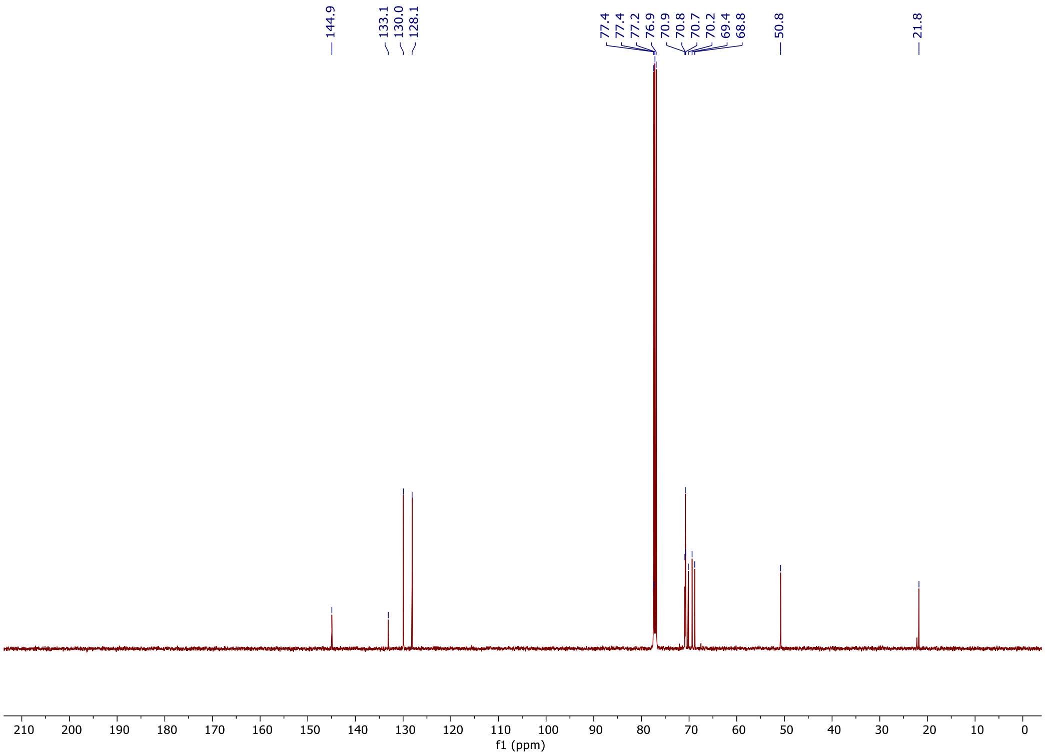
144.9
133.1
130.0
128.1
77.4
77.4
77.2
76.9
70.9
70.8
70.7
70.2
69.4
68.8
50.8
21.8
210 200 190 180 170 160 150 140 130 120 110 100 90 80 70 60 50 40 30 20 10 0
f1 (ppm)

2-(4-(2-(2-(2-(2-Azidoethoxy)ethoxy)ethoxy)ethoxy)phenyl)isoindoline-1,3-dione S12[3]

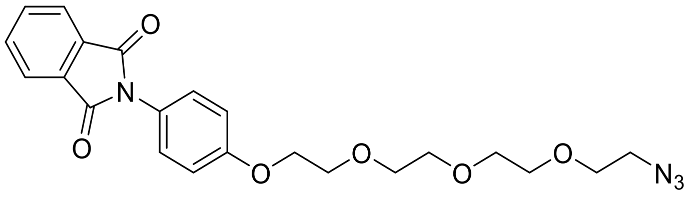

S12

To a solution of 2-(4-hydroxyphenyl)isoindoline-1,3-dione **S9** (271 mg, 1.14 mmol) in anhydrous DMF (20 mL) was added *t*BuOK (128 mg, 1.14 mmol) and the solution stirred at 21 °C for 10 min. After this time was added 2-(2-(2-(2-azidoethoxy)ethoxy)ethoxy)ethyl 4-methylbenzenesulfonate **S11** (282 mg, 0.76 mmol) and the reaction mix stirred at 90 °C for 20 h. After this, the reaction was diluted with water (100 mL) and the product extracted with EtOAc (4 × 50 mL). The organic extracts were washed with sat. aq. LiCl (2 × 30 mL), dried ($MgSO_4$) and the solvent removed *in vacuo*. The crude product was purified *via* flash column chromatography (20-80% EtOAc/cyclohexane) to give the product 2-(4-(2-(2-(2-(2-azidoethoxy)ethoxy)ethoxy)ethoxy)phenyl)isoindoline-1,3-dione **S12** (135 mg, 0.31 mmol, 40%) as a yellow oil.

^{1}H NMR (500 MHz, $CDCl_3$) δ 7.93 (dd, *J* = 5.5, 3.0 Hz, 2H), 7.77 (dd, *J* = 5.5, 3.0 Hz, 2H), 7.31 (d, *J* = 8.9 Hz, 2H), 7.03 (d, *J* = 8.9 Hz, 2H), 4.17 (t, *J* = 4.8 Hz, 2H), 3.87 (dd, *J* = 5.7, 4.1 Hz, 2H), 3.73 (dd, *J* = 6.2, 3.5 Hz, 2H), 3.71–3.63 (m, 8H), 3.37 (t, *J* = 5.0 Hz, 2H).

^{13}C NMR (151 MHz, $CDCl_3$) δ 167.7 (C), 158.6 (C), 134.5 (CH), 131.9 (C), 128.0 (CH), 123.8 (CH), 115.3 (CH), 77.4 (CH_2), 77.2 (CH_2), 76.9 (CH_2), 71.0 (CH_2), 70.8 (CH_2), 70.2 (CH_2), 69.7 (CH_2), 67.8 (CH_2), 50.8 (CH_2).

IR (thin film) 2921, 2858, 2114, 1784, 1705, 1612, 1588, 1516 cm^{-1}.

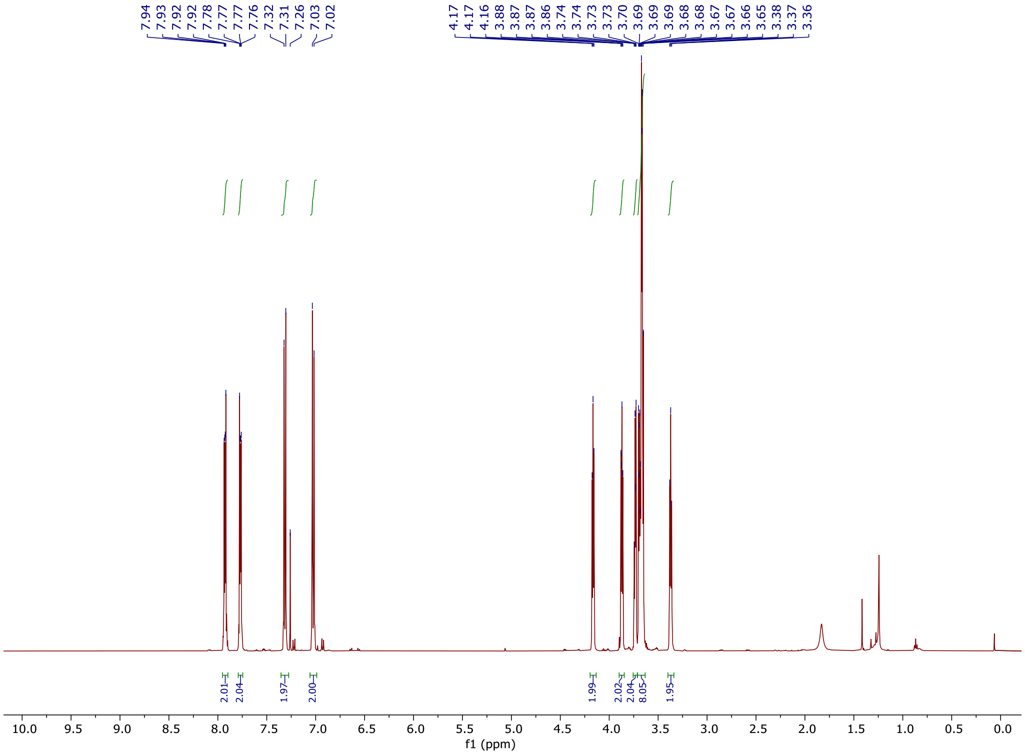
7.94
7.93
7.92
7.92
7.78
7.77
7.77
7.76
7.32
7.31
7.26
7.03
7.02
4.17
4.17
4.16
3.88
3.87
3.86
3.74
3.74
3.73
3.73
3.70
3.69
3.69
3.69
3.68
3.68
3.67
3.67
3.66
3.65
3.38
3.37
3.36
2.01
2.04
1.97
2.00
1.99
2.02
2.04
8.05
1.95
f1 (ppm)

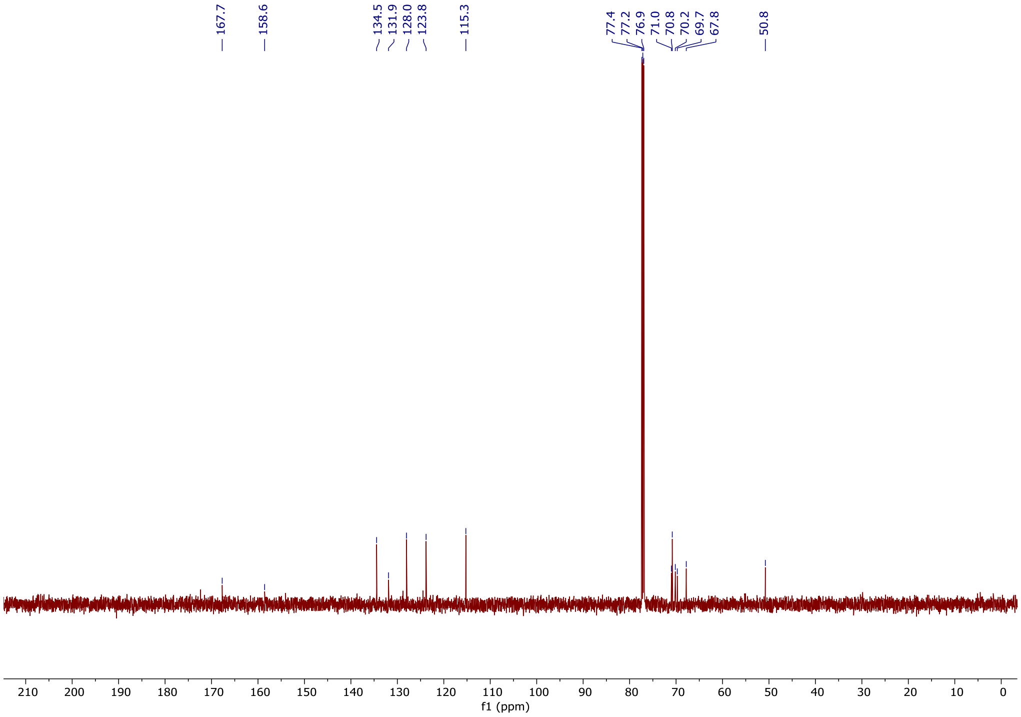
167.7
158.6
134.5
131.9
128.0
123.8
115.3
77.4
77.2
76.9
71.0
70.8
70.2
69.7
67.8
50.8
f1 (ppm)

4-(2-(2-(2-(2-Azidoethoxy)ethoxy)ethoxy)ethoxy)aniline (N_3 Aniline) 3

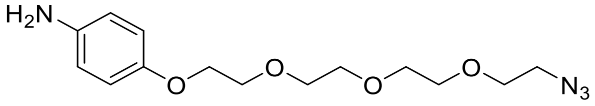

3

To a solution of 2-(4-(2-(2-(2-(2-azidoethoxy)ethoxy)ethoxy)ethoxy)phenyl)isoindoline-1,3-dione **S12** (135 mg, 0.31 mmol) in THF (20 mL) was added hydrazine monohydrate (307 µL, 3.72 mmol, 60% solution). The reaction was then stirred at 22 °C for 2 h. After this time the reaction mixture was diluted with water (100 mL) and the product extracted with EtOAc (3 × 30 mL). The organic layers were combined, washed with brine (30 mL) and dried ($MgSO_4$). The solvent was removed *in vacuo* and the crude product purified *via* flash column chromatography (20-100% EtOAc/cyclohexane) to give 4-(2-(2-(2-(2-azidoethoxy)ethoxy)ethoxy)ethoxy)aniline **3** (61.8 mg, 0.2 mmol, 64%) as a brown oil.

^{1}H NMR (500 MHz, $CDCl_3$) δ 6.76 (d, *J* = 8.7 Hz, 2H), 6.63 (d, *J* = 8.7 Hz, 2H), 4.05 (t, *J* = 4.9 Hz, 2H), 3.81 (dd, *J* = 5.6, 4.2 Hz, 2H), 3.73–3.70 (m, 2H), 3.70–3.65 (m, 8H), 3.37 (t, *J* = 5.1 Hz, 2H).

^{13}C NMR (126 MHz, $CDCl_3$) δ 152.1 (C), 140.2 (C), 116.5 (CH), 116.0 (CH), 70.9 (CH_2), 70.9 (CH_2), 70.8 (CH_2), 70.8 (CH_2), 70.2 (CH_2), 70.0 (CH_2), 68.3 (CH_2), 50.8 (CH_2), 29.8 (CH_2).

IR (thin film) 3384 3358, 2920, 2874, 2104, 1631, 1511 cm^{-1}.

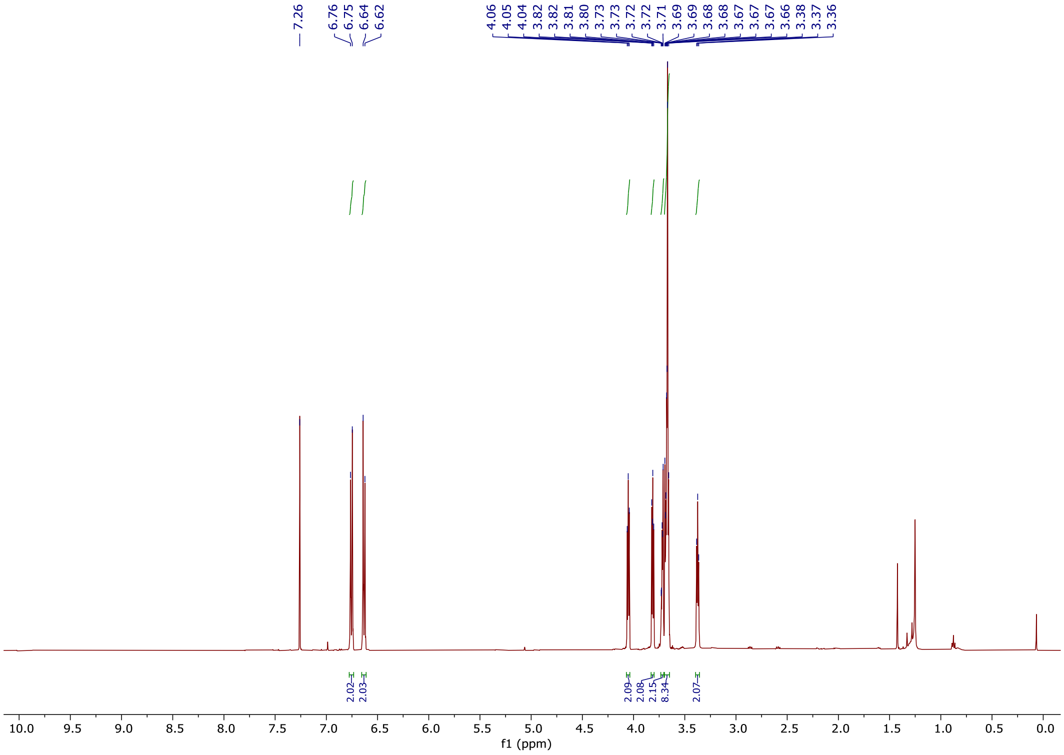
7.26
6.76
6.75
6.64
6.62
4.06
4.05
4.04
3.82
3.82
3.81
3.80
3.73
3.73
3.72
3.72
3.71
3.69
3.69
3.68
3.68
3.67
3.67
3.67
3.66
3.38
3.37
3.36
2.02
2.03
2.09
2.08
2.15
8.34
2.07
f1 (ppm)

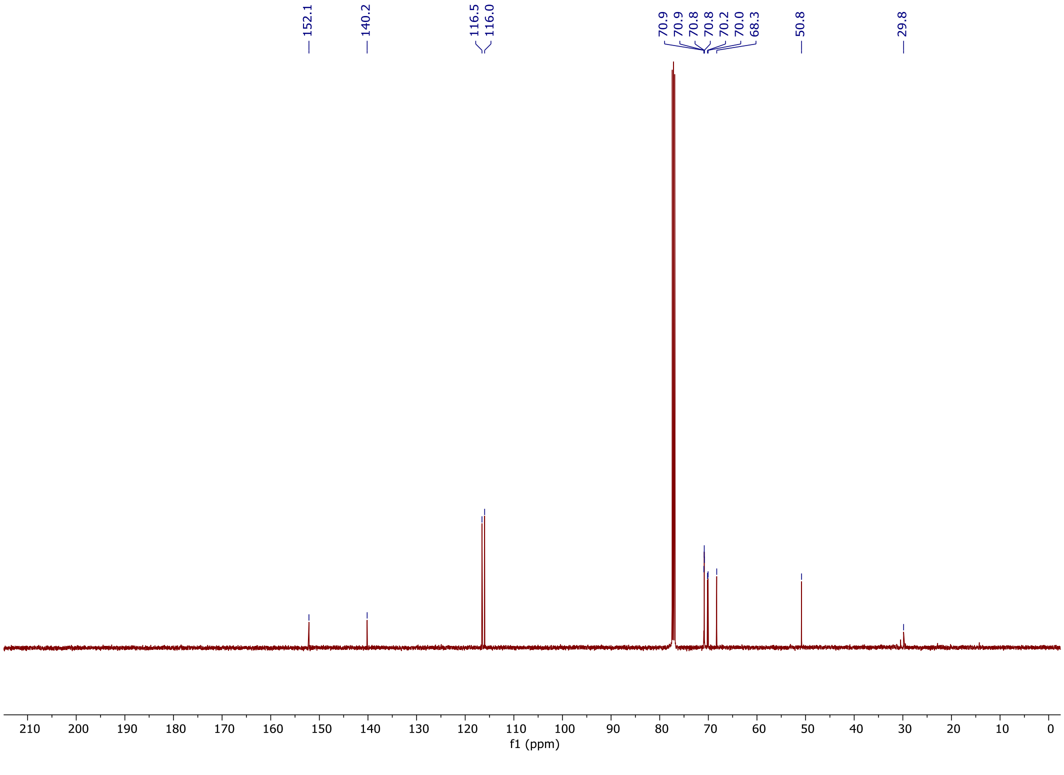
152.1
140.2
116.5
116.0
70.9
70.9
70.8
70.8
70.2
70.0
68.3
50.8
29.8
f1 (ppm)

Synthesis of diEt PD 4

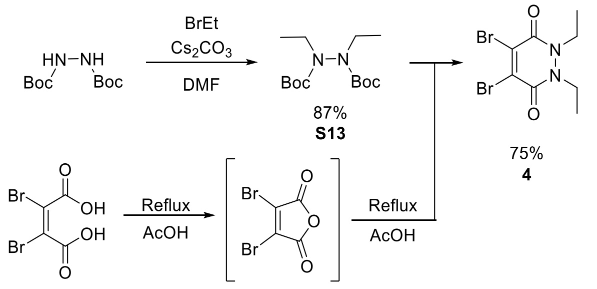

Figure S4. Synthetic Route to diEt PD, **4.** Diboc hydrazine is ethylated to form **S13**, which is subsequently refluxed with dibromomaleic anhydride to form the product.

Di-*tert*-butyl 1,2-diethylhydrazine-1,2-dicarboxylate S13[3]

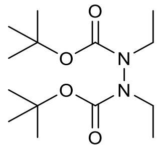

S13

To a solution of di-*tert*-butyl hydrazine-1,2-dicarboxylate (1.16 g, 5.0 mmol) in DMF (20 mL) was added caesium carbonate (3.26 g, 10.0 mmol) and bromoethane (0.7 mL, 14.7 mmol). The mixture was then stirred at 21 °C for 24 h, after this time was added another 0.5 eq. of caesium carbonate (0.81 g, 2.5 mmol). The reaction was stirred for a further 4 h. After this time the solvent was removed *in vacuo* with toluene co-evaporation (3 × 50 mL as an azeotrope). The crude reaction mixture was then dissolved in diethyl ether (100 mL) and washed with water (2 × 30 mL), followed by saturated aq. LiCl solution (2 × 30 mL). The organic layer was dried ($MgSO_4$) and concentrated *in vacuo* to give di-*tert*-butyl 1,2-diethylhydrazine-1,2-dicarboxylate **S13** (1.25 g, 4.3 mmol, 87%).

^{1}H NMR (600 MHz, $CDCl_3$, rotamers) δ 3.53-3.37 (m, 4H), 1.47-1.42 (m, 18H), 1.15 (t, *J* = 7.2 Hz, 6H).

^{13}C NMR (151 MHz, $CDCl_3$, rotamers*) δ 155.2 (C), 80.7 (C), 44.4 (CH_2), 28.4 (CH_3), 13.0 (CH_3).

IR (thin film) 2976, 2935, 1703 cm^{-1}.

*Only one peak per carbon is assigned

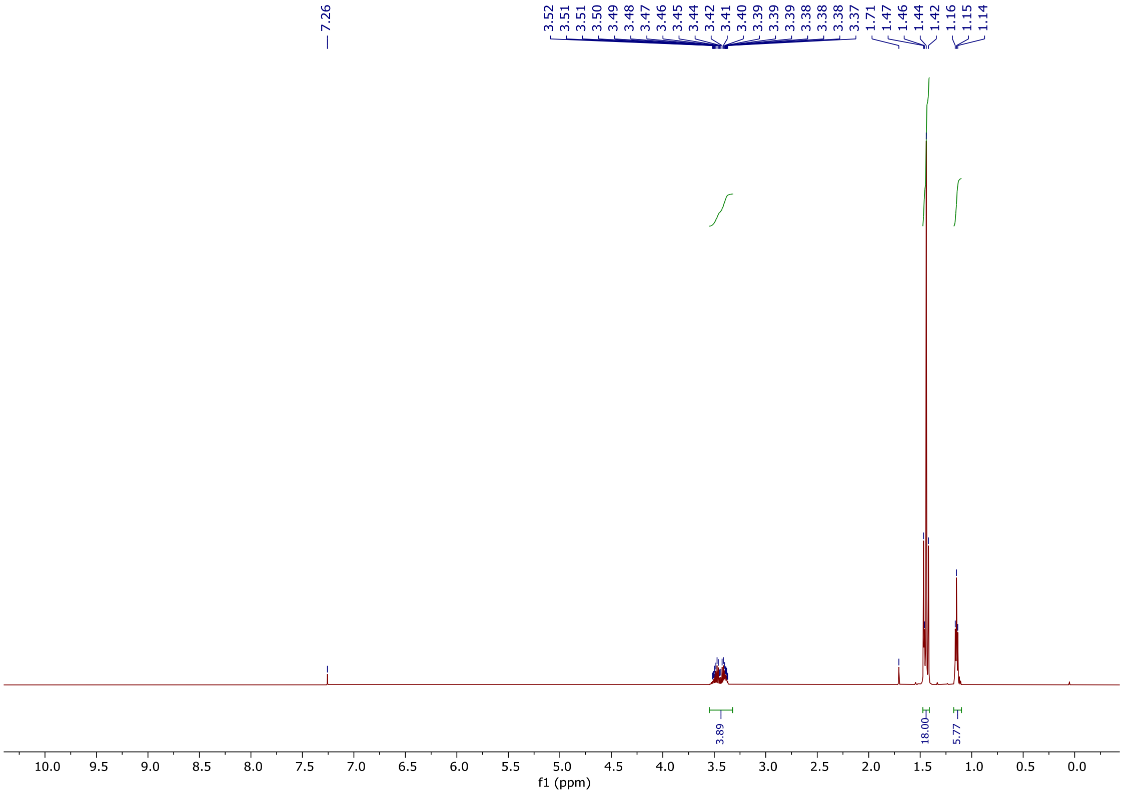
7.26
3.52 3.51 3.51 3.50 3.49 3.48 3.47 3.46 3.45 3.44 3.42 3.41 3.40 3.39 3.39 3.39 3.38 3.38 3.38 3.37 1.71 1.47 1.46 1.44 1.42 1.16 1.15 1.14
3.89
18.00
5.77
10.0 9.5 9.0 8.5 8.0 7.5 7.0 6.5 6.0 5.5 5.0 4.5 4.0 3.5 3.0 2.5 2.0 1.5 1.0 0.5 0.0
f1 (ppm)

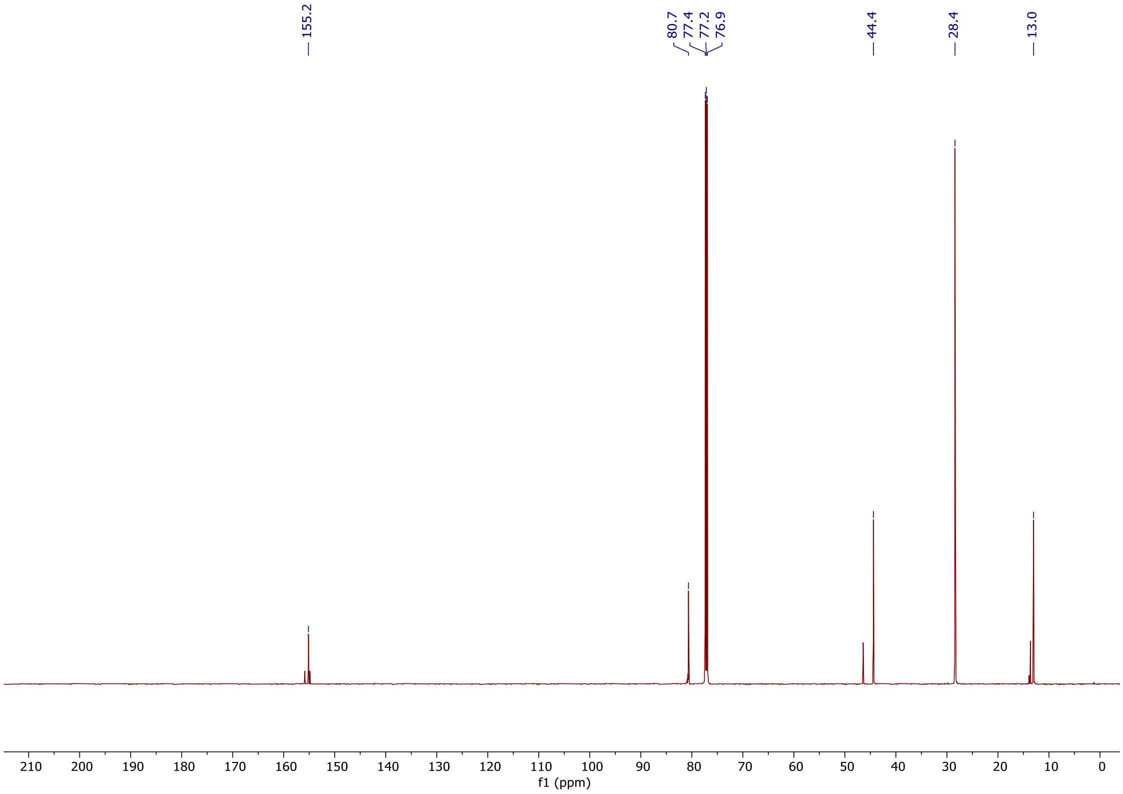
155.2
80.7 77.4 77.2 76.9
44.4
28.4
13.0
210 200 190 180 170 160 150 140 130 120 110 100 90 80 70 60 50 40 30 20 10 0
f1 (ppm)

4,5-Dibromo-1,2-diethyl-1,2-dihydropyridazine-3,6-dione (diEt PD) 4[3]

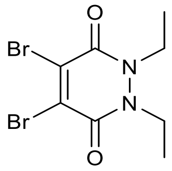

4

Dibromomaleic acid (219 mg, 0.8 mmol) was dissolved in AcOH (10 mL) and heated under reflux for 2 h. After this time, di-*tert*-butyl 1,2-diethylhydrazine-1,2-dicarbonate **S13** (288 mg, 1.0 mmol) was added and the mixture heated under reflux for a further 24 h. After this time, the solvent was removed *in vacuo* with toluene co-evaporation (3 × 30 mL, as an azeotrope). The crude product was then purified *via* flash column chromatography (20-80% EtOAc/cyclohexane) to give 4,5-dibromo-1,2-diethyl-1,2-dihydropyridazine-3,6-dione **4** (196 mg, 0.6 mmol, 75%) as a yellow solid.

^{1}H NMR (600 MHz, $CDCl_3$) δ 4.17 (q, *J* = 7.1 Hz, 4H), 1.29 (t, *J* = 7.1 Hz, 6H).

^{13}C NMR (151 MHz, $CDCl_3$) δ 153.3 (C), 136.1 (C), 42.5 (CH_2), 13.2 (CH_3).

IR (solid) 2979, 2937, 2873, 1629, 1574 cm^{-1}

.

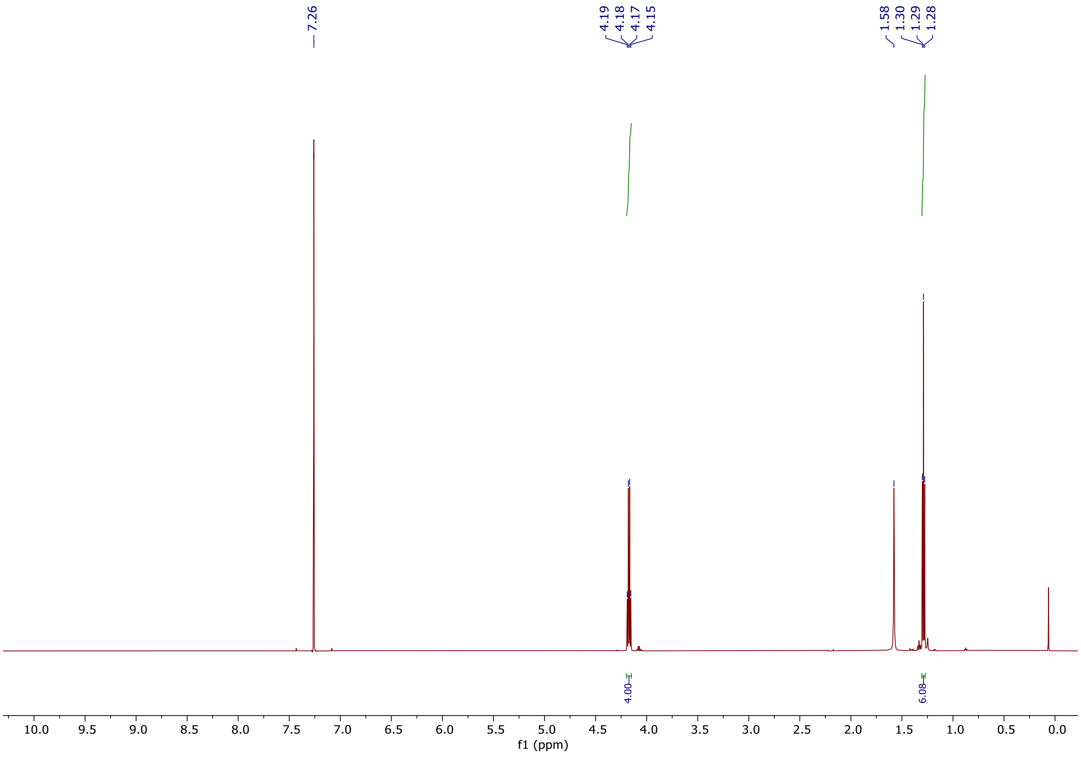
7.26
4.19
4.18
4.17
4.15
1.58
1.30
1.29
1.28
4.00
6.08
10.0
9.5
9.0
8.5
8.0
7.5
7.0
6.5
6.0
5.5
5.0
4.5
4.0
3.5
3.0
2.5
2.0
1.5
1.0
0.5
0.0
f1 (ppm)

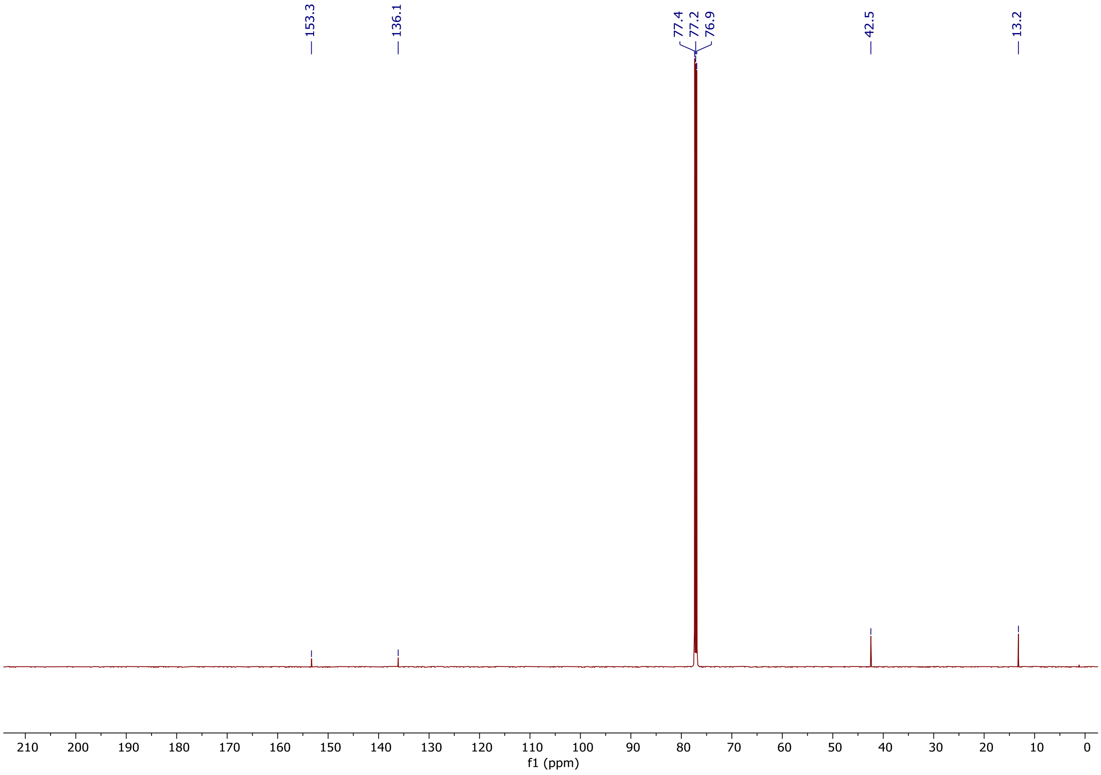
153.3
136.1
77.4
77.2
76.9
42.5
13.2
210
200
190
180
170
160
150
140
130
120
110
100
90
80
70
60
50
40
30
20
10
0
f1 (ppm)

Synthesis of diMe bisPD 5

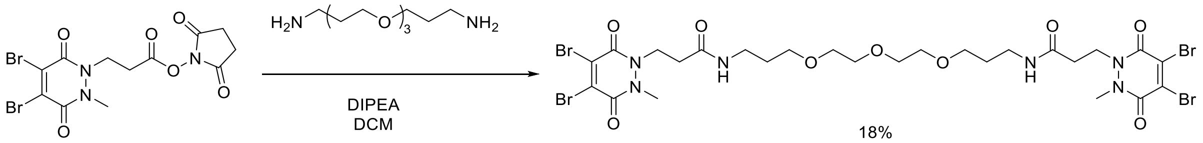

S4 **5**

Figure S5. Synthetic Route to diMe bisPD, **5. S4** is reacted with a $(PEG)_3$ amine to form the product.

N,*N'*-(((Oxybis(ethane-2,1-diyl))bis(oxy))bis(propane-3,1-diyl))bis(3-(4,5-dibromo-2-methyl-3,6-dioxo-3,6-diydropyridazin-1(2*H*)-yl)propanamide) (diMe bisPD) 5

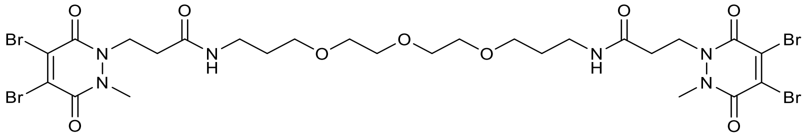

5

To a solution of 4,7,10-trioxa-1,13-tridecanediamine (24.3 mg, 0.11 mmol) in DCM (10 mL) was added DIPEA (38.3 µL, 0.22 mmol) and the solution stirred at 21 °C for 30 min. After this time, was added dropwise 2,5-dioxopyrrolidin-1-yl 3-(4,5-dibromo-2-methyl-3,6-dioxo-3,6-dihydropyridazin-1(2*H*)-yl) propanoate **S4** (150 mg, 0.33 mmol) pre-dissolved in DCM (5 mL), and the solution stirred at 21 °C for 24 h. After this time the reaction mixture was washed with water (2 × 30 mL) then brine (30 mL). The aqueous phases were combined and the product extracted with DCM (60 mL). The organic phases were combined, dried ($MgSO_4$) and the solvent removed *in vacuo*. The crude product was purified *via* flash column chromatography (0-30% MeOH/EtOAc) to give *N*,*N'*-(((oxybis(ethane-2,1-diyl))bis(oxy))bis(propane-3,1-diyl))bis(3-(4,5-dibromo-2-methyl-3,6-dioxo-3,6-diydropyridazin-1(2*H*)-yl)propanamide) **5** (18 mg, 0.02 mmol, 18%) as a yellow oil.

^{1}H NMR (600 MHz, $CDCl_3$, rotamers) δ 6.99–6.87 (m, 2H), 4.46–4.31 (m, 4H), 3.69 (d, 6H), 3.60–3.47 (m, 12H), 3.30 (m, 4H), 2.60–2.45 (m, 4H), 1.70 (m, 4H).

^{13}C NMR (151 MHz, $CDCl_3$, rotamers) δ 169.1 (C), 152.9 (C), 150.6 (C), 136.4 (C), 135.4 (C), 70.0 (CH_2), 70.0 (CH_2), 60.9 (CH_2), 44.5 (CH_2), 38.3 (CH_2), 35.2 (CH_2), 34.2 (CH_2), 28.7 (CH_3).

*Some impurities are present in this sample

LRMS (ESI) 893 (20, $[M^{79}Br_4+H]^+$), 895 (70, $[M^{79}Br_3{}^{81}Br+H]^+$) 897 (100, $[M^{79}Br_2{}^{81}Br_2+H]^+$), 899 (70, $[M^{79}Br^{81}Br_3+H]^+$), 901 (20, $[M^{81}Br_4+H]^+$); **HRMS (ESI)** calcd for $C_{26}H_{37}O_9N_6Br_4$ $[M^{81}Br_2{}^{79}Br_2+H]^+$ 896.9309; observed 896.9241.

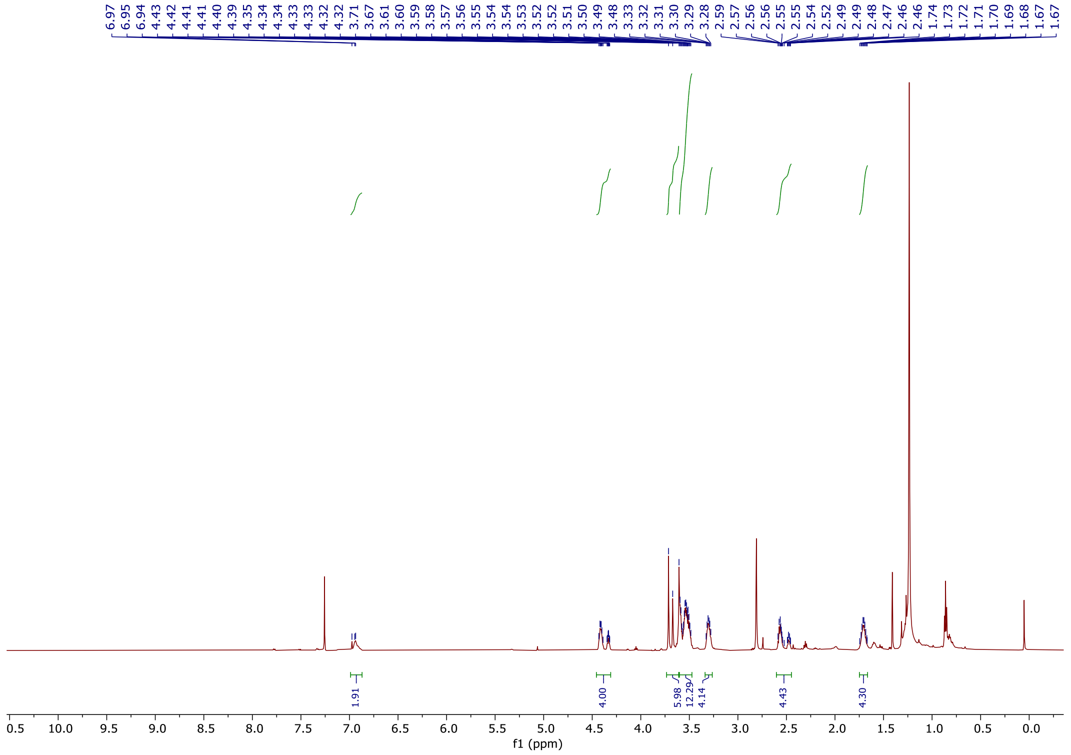
6.97
6.95
6.94
4.43
4.42
4.41
4.40
4.39
4.35
4.34
4.34
4.33
4.33
4.32
4.32
3.71
3.67
3.61
3.60
3.59
3.58
3.57
3.56
3.55
3.54
3.54
3.53
3.52
3.52
3.51
3.50
3.49
3.48
3.33
3.32
3.31
3.30
3.29
3.28
2.59
2.57
2.56
2.56
2.55
2.55
2.54
2.52
2.49
2.49
2.48
2.47
2.46
2.46
1.74
1.73
1.72
1.71
1.70
1.69
1.68
1.67
1.67
1.91
4.00
5.98
12.29
4.14
4.43
4.30
f1 (ppm)
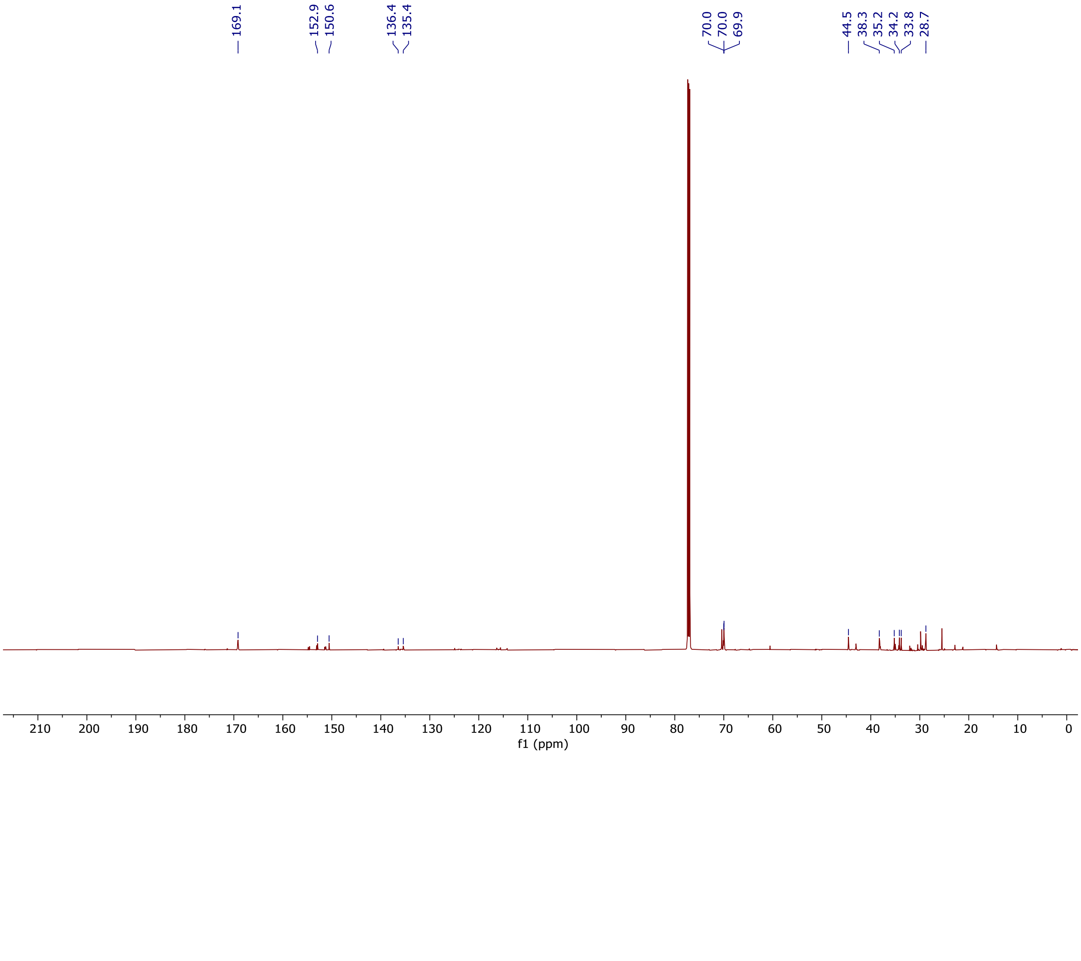
169.1
152.9
150.6
136.4
135.4
70.0
70.0
69.9
44.5
38.3
35.2
34.2
33.8
28.7
f1 (ppm)

Chemical Biology

Capped LC S168C Thio-Trastuzumab Mutant 6

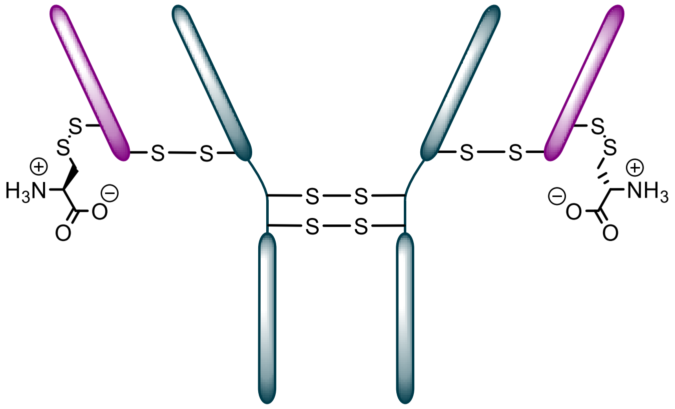

6

LC S168C thio-trastuzumab was obtained capped and analysed unmodified by LC-MS (method 1a).

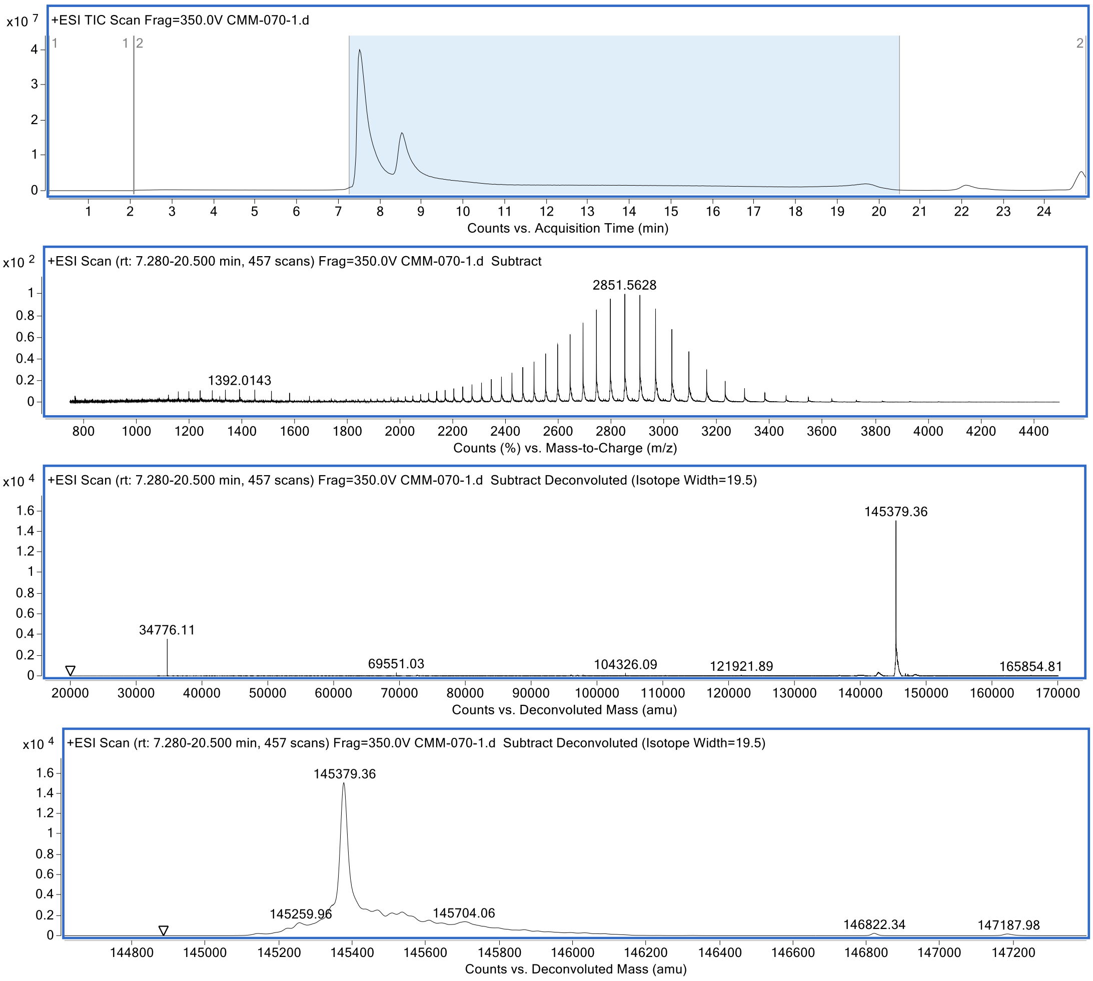

Figure S6: (i) TIC LC-MS trace (top), (ii) non-deconvoluted LC-MS trace (upper middle), (iii) deconvoluted MS data (lower middle, wide range), (iv) deconvoluted MS data (bottom, zoom in range).

Uncapped HC S378C Thio-Trastuzumab Mutant 7

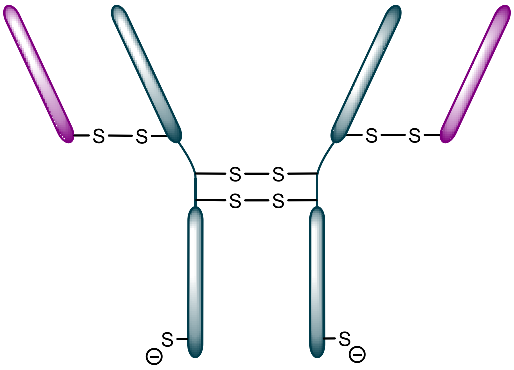

7

HC S378C thio-trastuzumab was obtained uncapped and analysed unmodified by LC-MS (method 1a).

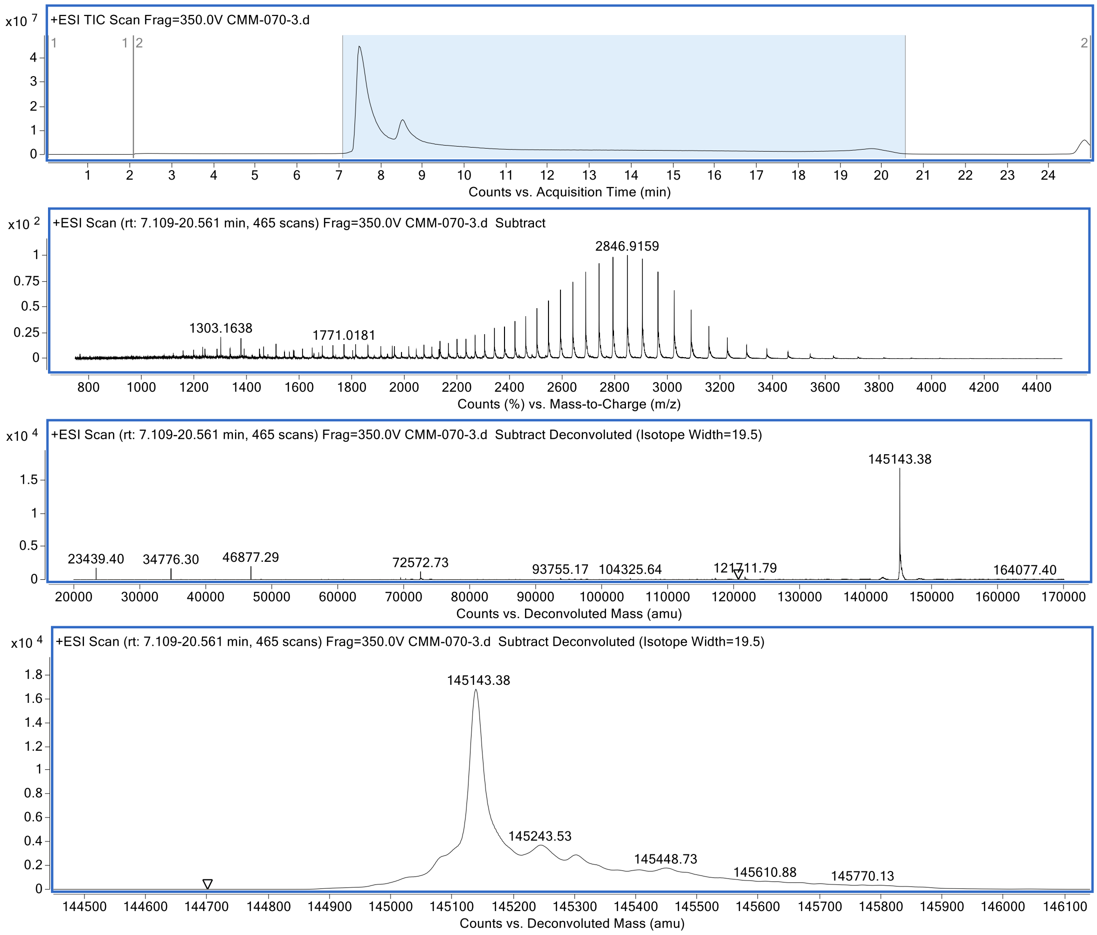

Figure S7: (i) TIC LC-MS trace (top), (ii) non-deconvoluted LC-MS trace (upper middle), (iii) deconvoluted MS data (lower middle, wide range), (iv) deconvoluted MS data (bottom, zoom in range).

Uncapped LC S168C Thio-Trastuzumab Mutant 8

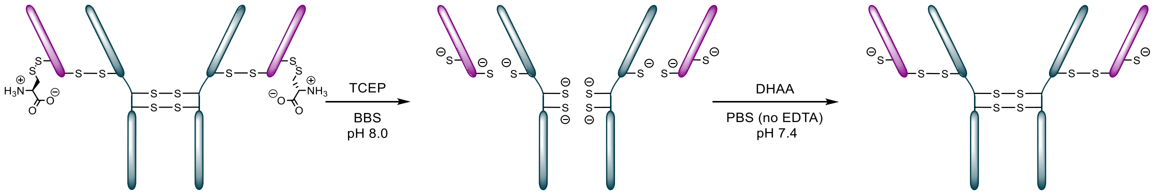

6 25 8

LC S168C thio-trastuzumab was obtained with the mutant thiols capped (see **6**), therefore an uncapping procedure was required.

To a solution of LC S168C thio-trastuzumab **6** (100 µL, 20 µM) in BBS (25 mM borate, 25 mM NaCl, 2 mM EDTA, 2% DMSO, pH 8.0) was added TCEP.HCl (20 mM in dH_2O, 10 eq.) and the resulting solution incubated for 3 h at 37 °C. After this time, the TCEP was removed *via* ultrafiltration into PBS (10 mM phosphates, 2.7 mM KCl, 137 mM NaCl, pH 7.4) and (in the case of conjugates **8a-e**) DHAA (20 mM in PBS, A eq.) was added. In the case of conjugates **8f-k**, no further reagents were added. The reaction was then incubated at B °C for C h/min. After this time, the DHAA was removed *via* ultrafiltration into BBS and the product analysed by LC-MS (method 1b).

As LC-MS analysis at this point does not show whether over oxidation has occurred, to assess the extent of conjugation, the uncapped conjugates were immediately reacted with *N*-methylmaleimide (NMM) or BCN PD (see conjugates **S14** and **9**, respectively).

	Oxidant + Eq. (A)	Re-oxidation Temperature (°C) (B)	Re-oxidation Time (C)
**8a**	DHAA, 20	22	1.5 h
**8b**	DHAA, 10	22	1.5 h
**8c**	DHAA, 10	22	30 min
**8d**	DHAA, 10	22	20 min
**8e**	DHAA, 10	22	10 min
**8f**	Air	22	16 h
**8g**	Air	22	2.5 h
**8h**	Air	22	2 h
**8i**	Air	22	1.5 h
**8j**	Air	37	1 h
**8k**	Air	37	30 min

Table S6. Conditions used to form **8**.

8a

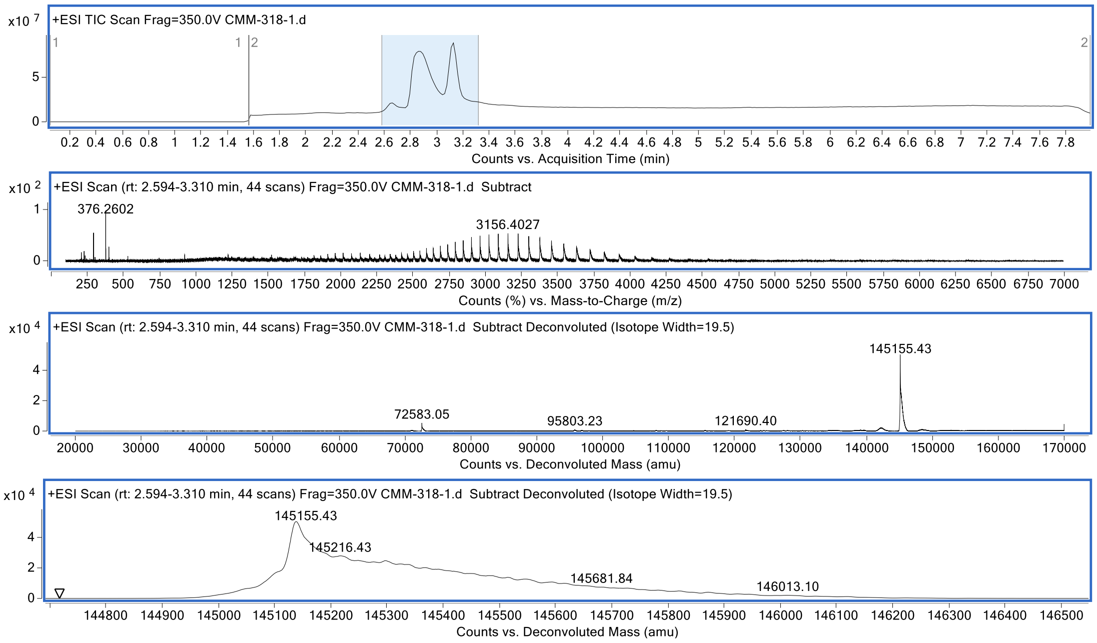

Figure S8: (i) TIC LC-MS trace (top), (ii) non-deconvoluted LC-MS trace (upper middle), (iii) deconvoluted MS data (lower middle, wide range), (iv) deconvoluted MS data (bottom, zoom in range).

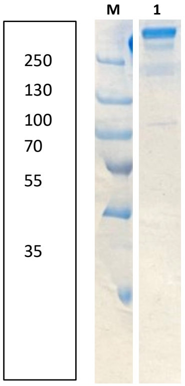

Figure S9: SDS-PAGE gel: M. Ladder, 1. Conjugate **8**

No LC-MS is available for **8b - 8k** as they were immediately reacted with NMM or Mepstra PD **1** to assess uncapping, for post-conjugation MS see conjugates **S14** and **9** (respectively).

LC S168C NMM conjugate S14 (LC S168C Thio-Trastuzumab Conjugated to *N*-Methylmaleimide)

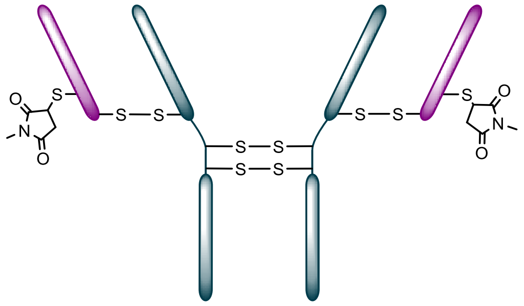

S14

To a solution of uncapped LC S168C thio-trastuzumab **8c** (30 µL, 20 µM) in BBS (25 mM borate, 25 mM NaCl, 2 mM EDTA, 2% DMSO, pH 8.0) was added NMM (0.12 µL, 20 mM in DMSO, 4 eq.) and the reaction incubated at 22 °C for 3 h under constant agitation (300 rpm). After this time, excess reagents were removed to give conjugate **S14** (expected mass 145,365 Da, observed mass 145,366 Da) which was analysed by LC-MS (method 1a), UV-Vis spectroscopy and SDS-PAGE.

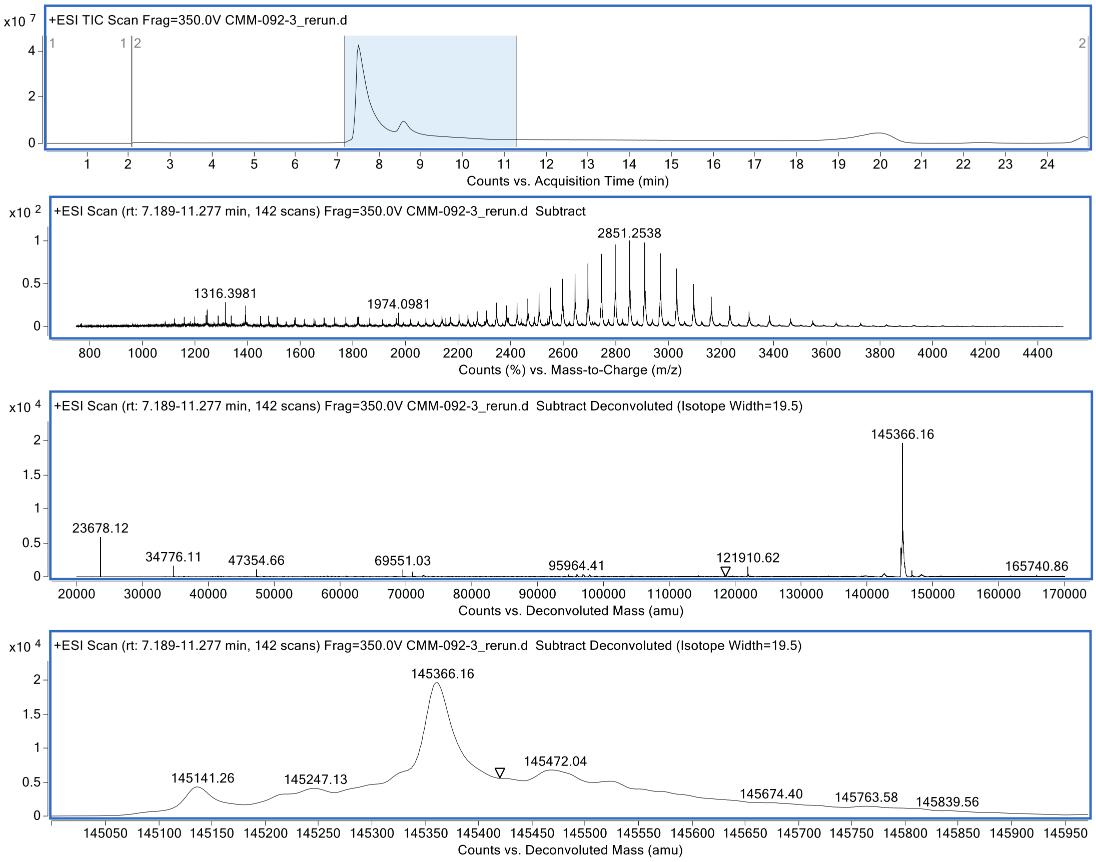

Figure S10: (i) TIC LC-MS trace (top), (ii) non-deconvoluted LC-MS trace (upper middle), (iii) deconvoluted MS data (lower middle, wide range), (iv) deconvoluted MS data (bottom, zoom in range).

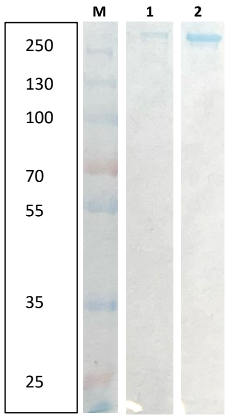

Figure S11: SDS-PAGE gel: M. Ladder, 1. Conjugate **8c**, 2. Conjugate **S14**

LC S168C PD conjugate 9 (LC S168C Thio-Trastuzumab Conjugated to BCN PD)

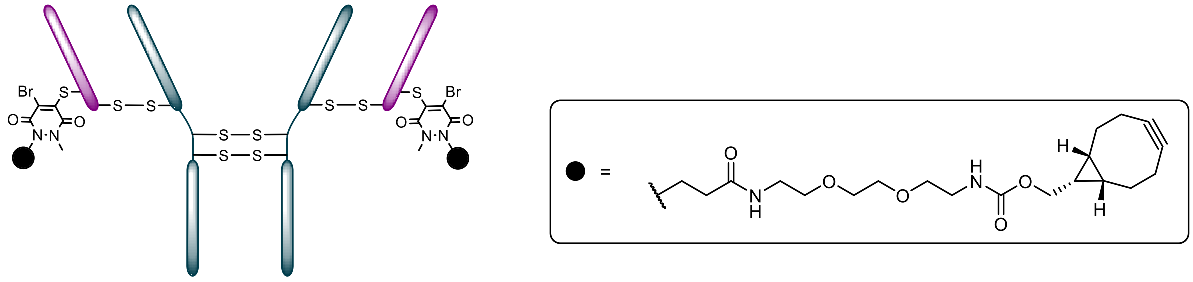

9

Uncapping Tests

To a solution of uncapped LC S168C thio-trastuzumab **8A** (100 µL, 20 µM) in BBS (25 mM borate, 25 mM NaCl, 2 mM EDTA, 2% DMSO, pH C) was added BCN PD **1** (20 mM in DMSO, 4 eq.) and the reaction incubated at 37 °C for 3 h under constant agitation (300 rpm). After this time, excess reagents were removed to give conjugate **9** (expected mass 146,307 Da, observed mass 146,307 Da) which was analysed by LC-MS (method 1b), UV-Vis spectroscopy and SDS-PAGE.

	Uncapping Protocol (A)
**9a**	**8a**
**9b**	**8b**
**9c**	**8c**
**9d**	**8d**
**9e**	**8e**
**9f**	**8f**
**9g**	**8g**
**9h**	**8h**
**9i**	**8i**
**9j**	**8j**
**9k**	**8k**

Table S7. Conditions used to form **9**.

Optimisation on Uncapped Ab

To a solution of uncapped LC S168C thio-trastuzumab **8** (100 µL, B µM) in BBS (25 mM borate, 25 mM NaCl, 2 mM EDTA, 2% DMSO, pH C) was added BCN PD **1** (20 mM in DMSO, D eq.) and the reaction incubated at 37 °C for E h under constant agitation (300 rpm). After this time, excess reagents were removed to give conjugate **9** (expected mass 146,309 Da, observed mass 146,308 Da) which was analysed by LC-MS (method 1b), UV-Vis spectroscopy and SDS-PAGE.

	Antibody Concentration (B)	Buffer pH (C)	BCN PD Eq. (D)	Reaction Time (E)
**9l**	20	8.0	8	3 h
**9m**	20	8.0	5	16 h
**9n**	20	8.0	4	1 h
**9o**	20	8.0	4	2 h
**9p**	20	8.0	4	3 h
**9q**	20	8.0	4	4 h
**9r**	20	8.5	4	3 h
**9s**	10	8.0	4	3 h

Table S8. Conditions used to form **9**.

9a

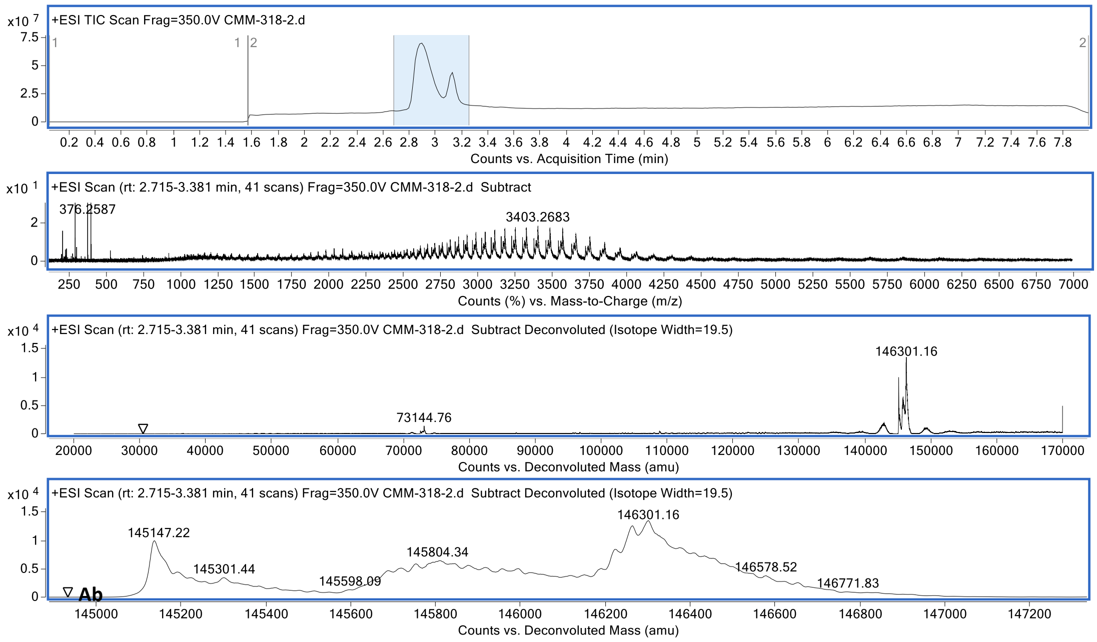

Figure S12: (i) TIC LC-MS trace (top), (ii) non-deconvoluted LC-MS trace (upper middle), (iii) deconvoluted MS data (lower middle, wide range), (iv) deconvoluted MS data (bottom, zoom in range).

9b

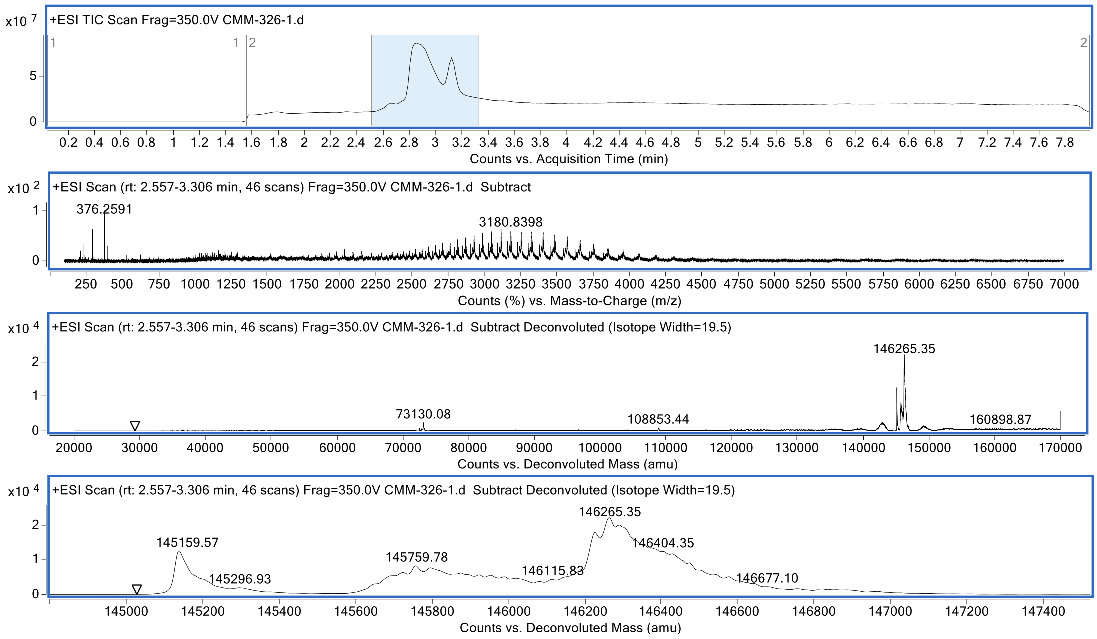

Figure S13: (i) TIC LC-MS trace (top), (ii) non-deconvoluted LC-MS trace (upper middle), (iii) deconvoluted MS data (lower middle, wide range), (iv) deconvoluted MS data (bottom, zoom in range).

9c

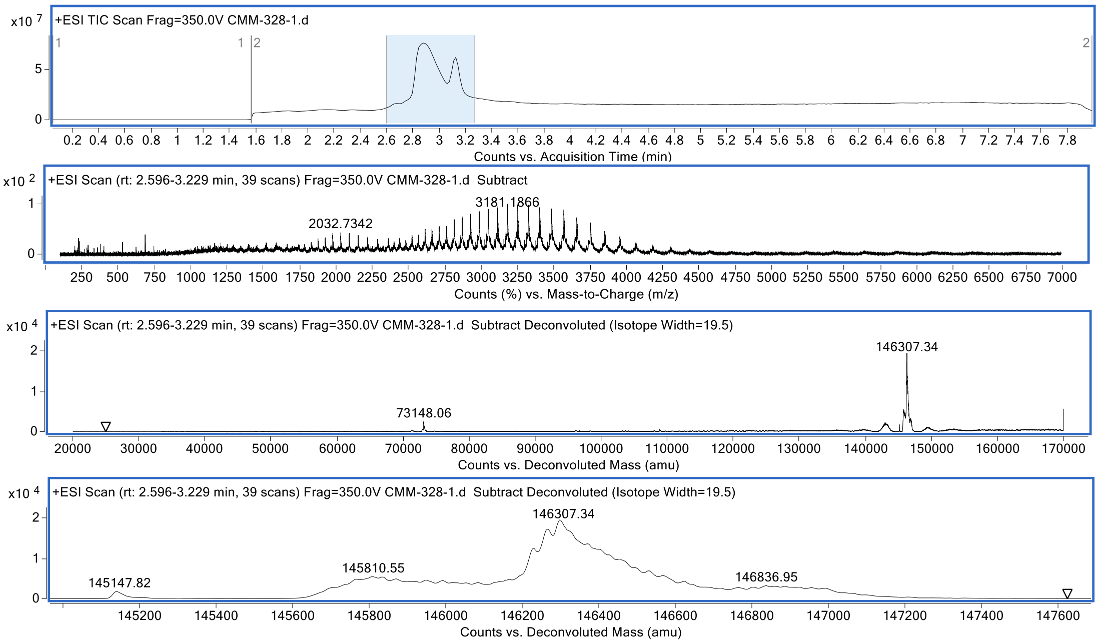

Figure S14: (i) TIC LC-MS trace (top), (ii) non-deconvoluted LC-MS trace (upper middle), (iii) deconvoluted MS data (lower middle, wide range), (iv) deconvoluted MS data (bottom, zoom in range).

9d

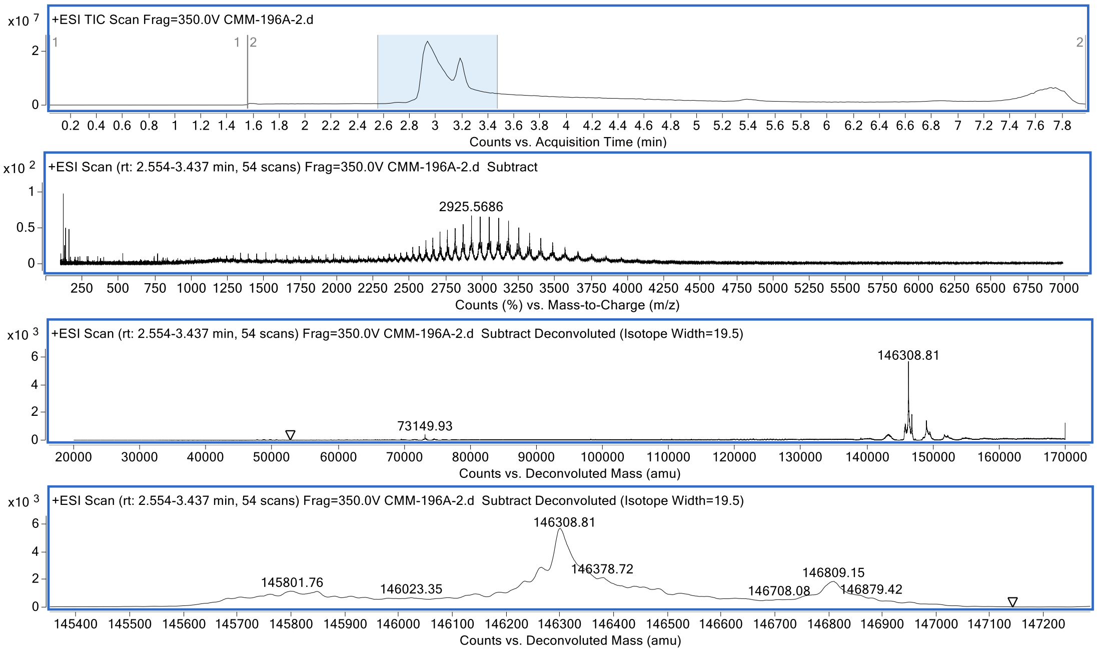

Figure S15: (i) TIC LC-MS trace (top), (ii) non-deconvoluted LC-MS trace (upper middle), (iii) deconvoluted MS data (lower middle, wide range), (iv) deconvoluted MS data (bottom, zoom in range).

9e

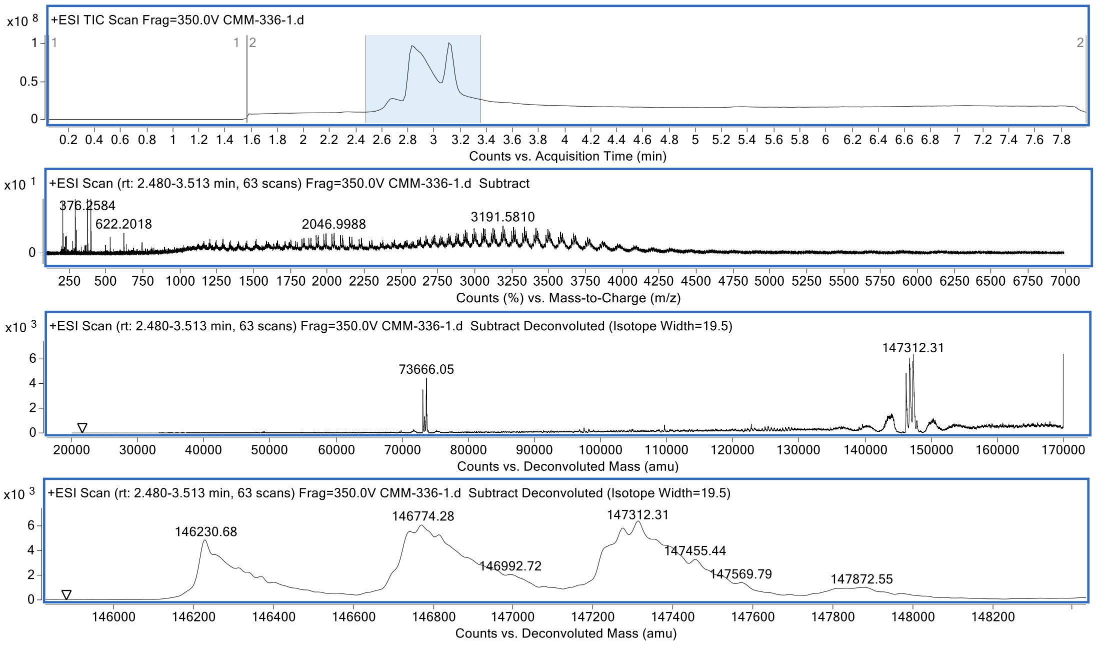

Figure S16: (i) TIC LC-MS trace (top), (ii) non-deconvoluted LC-MS trace (upper middle), (iii) deconvoluted MS data (lower middle, wide range), (iv) deconvoluted MS data (bottom, zoom in range).

9f

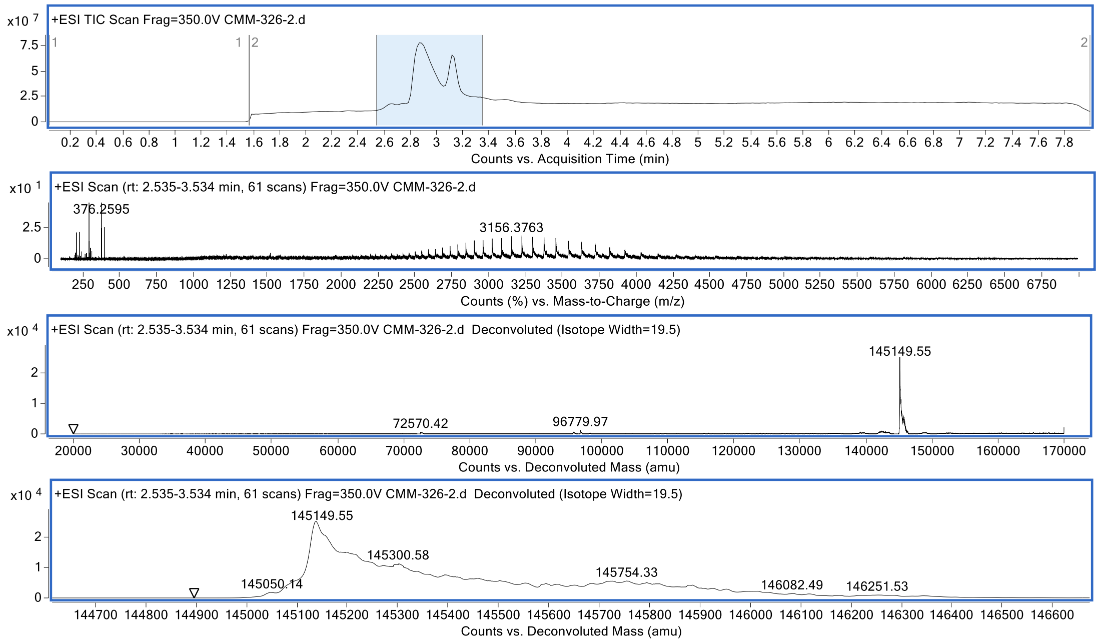

Figure S17: (i) TIC LC-MS trace (top), (ii) non-deconvoluted LC-MS trace (upper middle), (iii) deconvoluted MS data (lower middle, wide range), (iv) deconvoluted MS data (bottom, zoom in range).

9g

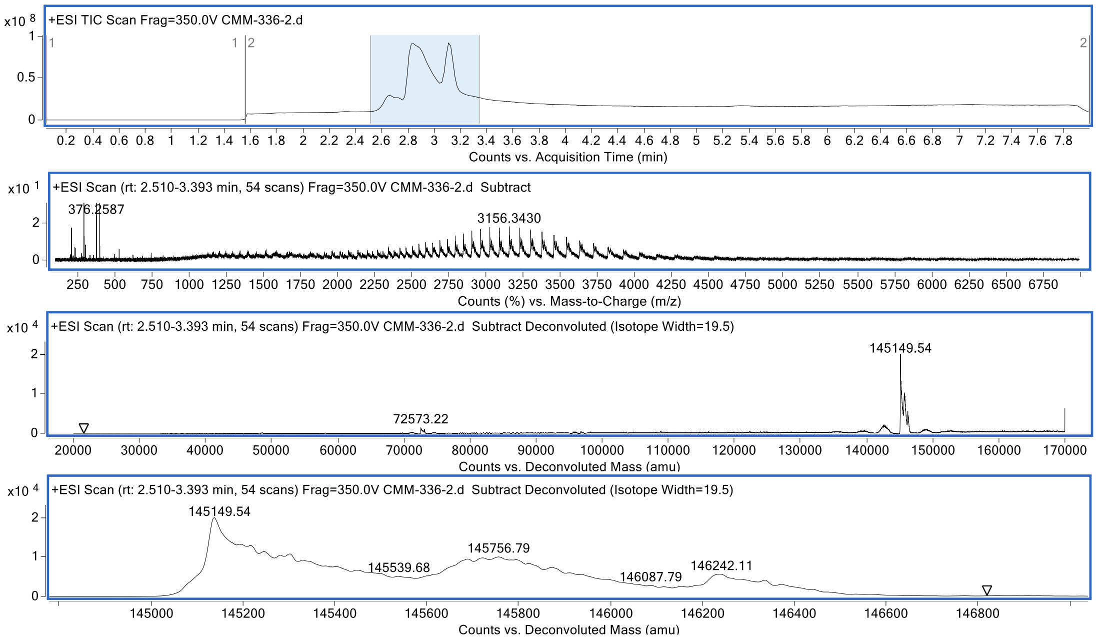

Figure S18: (i) TIC LC-MS trace (top), (ii) non-deconvoluted LC-MS trace (upper middle), (iii) deconvoluted MS data (lower middle, wide range), (iv) deconvoluted MS data (bottom, zoom in range).

9h

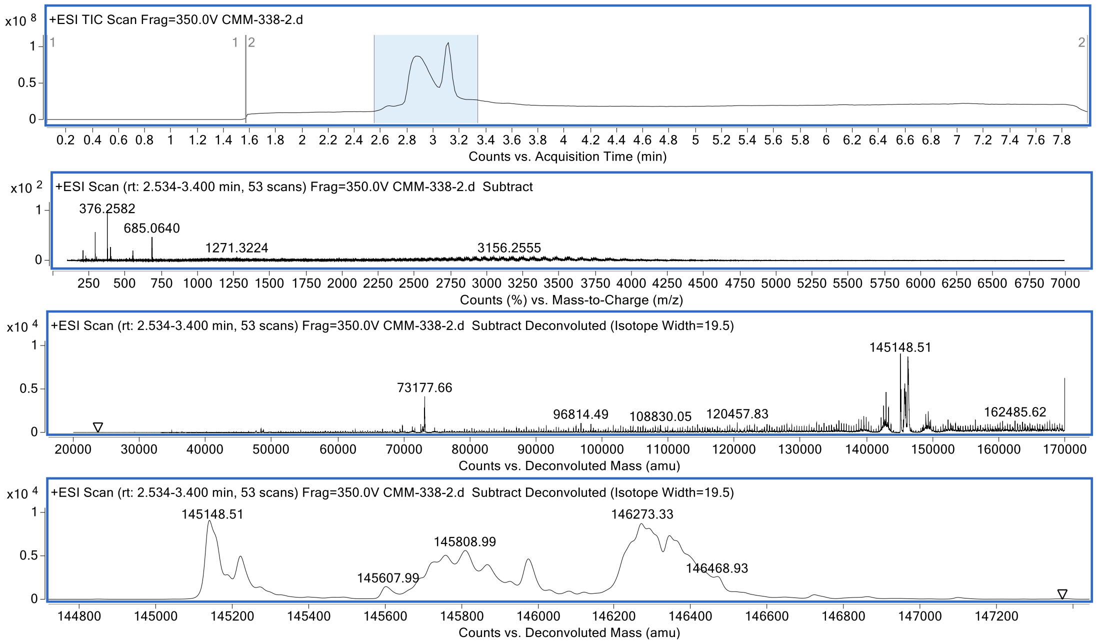

Figure S19: (i) TIC LC-MS trace (top), (ii) non-deconvoluted LC-MS trace (upper middle), (iii) deconvoluted MS data (lower middle, wide range), (iv) deconvoluted MS data (bottom, zoom in range).

9i

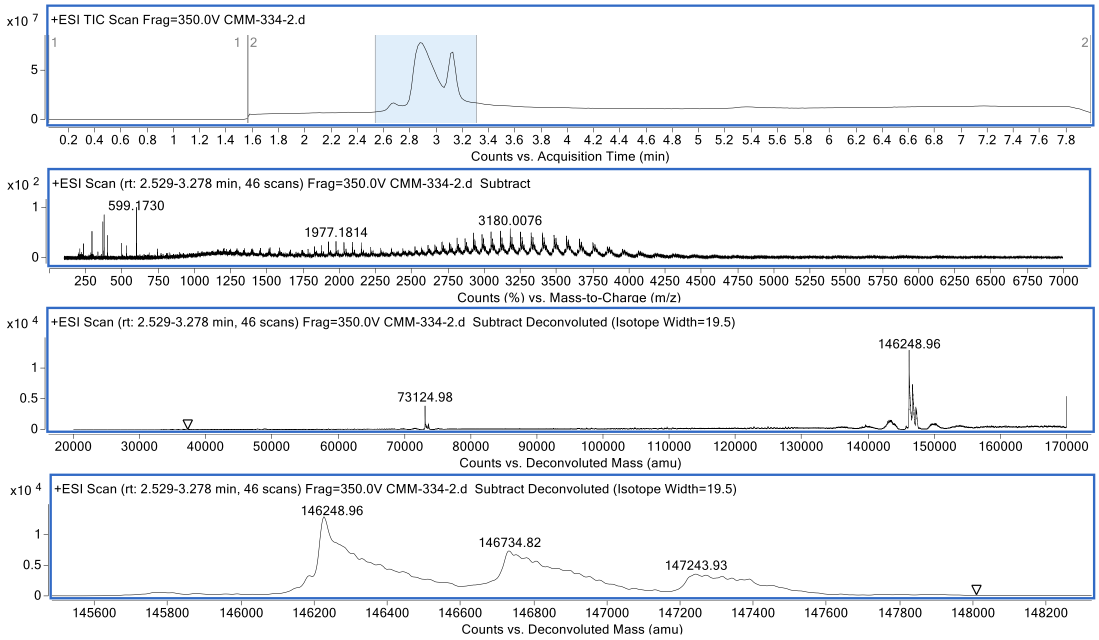

Figure S20: (i) TIC LC-MS trace (top), (ii) non-deconvoluted LC-MS trace (upper middle), (iii) deconvoluted MS data (lower middle, wide range), (iv) deconvoluted MS data (bottom, zoom in range).

9j

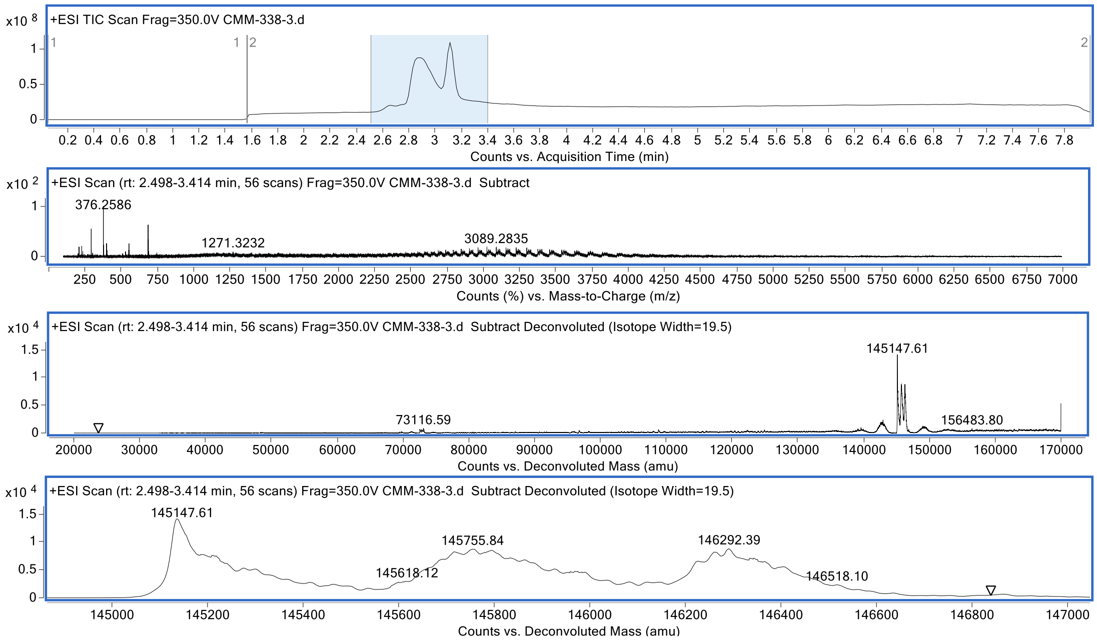

Figure S21: (i) TIC LC-MS trace (top), (ii) non-deconvoluted LC-MS trace (upper middle), (iii) deconvoluted MS data (lower middle, wide range), (iv) deconvoluted MS data (bottom, zoom in range).

9k

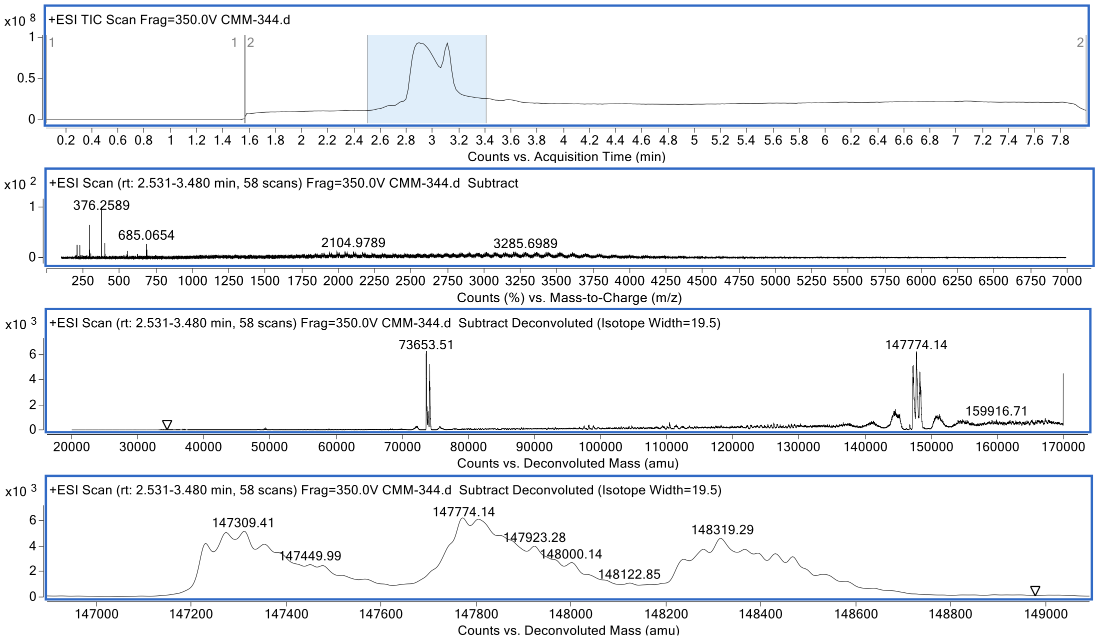

Figure S22: (i) TIC LC-MS trace (top), (ii) non-deconvoluted LC-MS trace (upper middle), (iii) deconvoluted MS data (lower middle, wide range), (iv) deconvoluted MS data (bottom, zoom in range).

9l

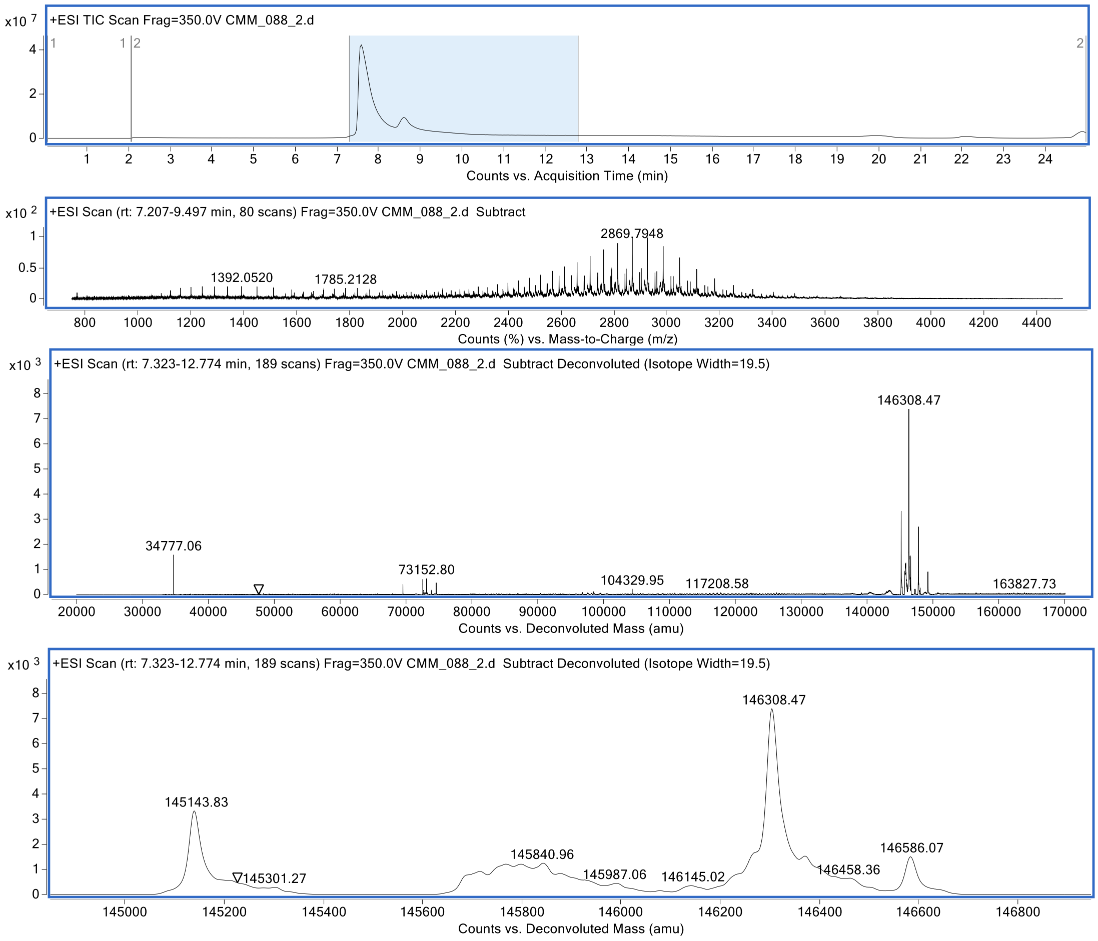

Figure S23: (i) TIC LC-MS trace (top), (ii) non-deconvoluted LC-MS trace (upper middle), (iii) deconvoluted MS data (lower middle, wide range), (iv) deconvoluted MS data (bottom, zoom in range).

9m

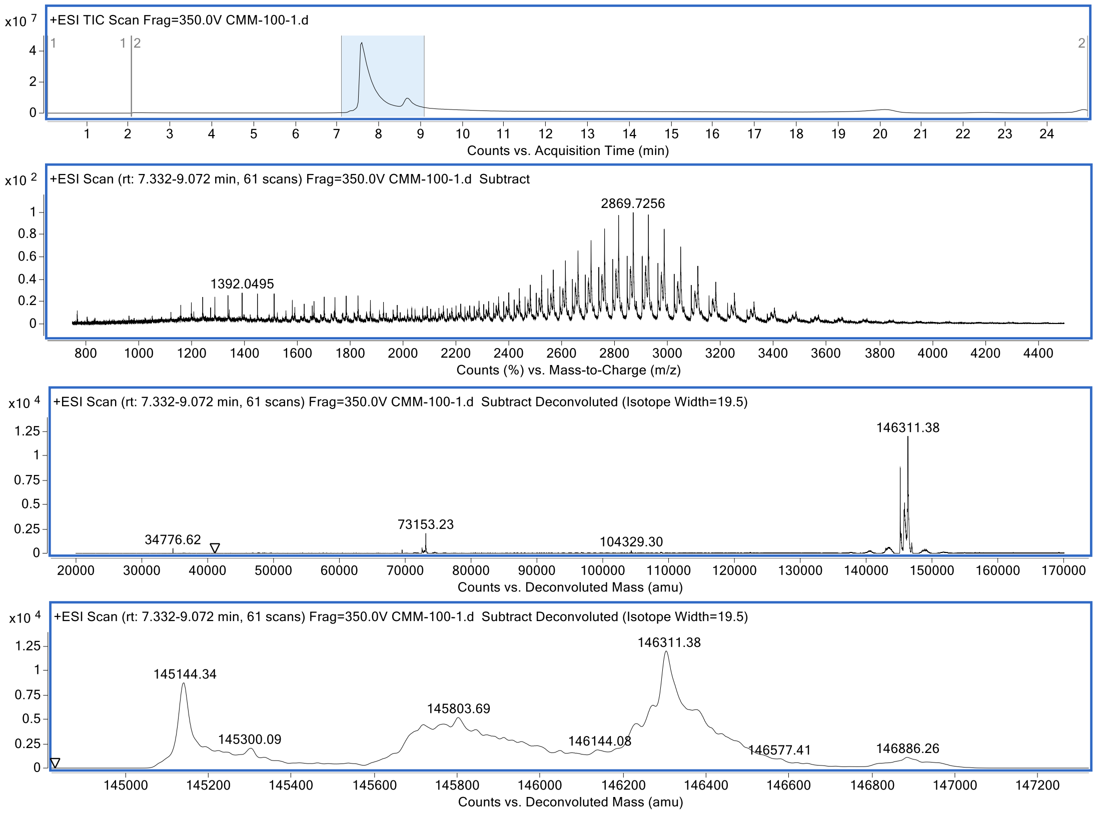

Figure S24: (i) TIC LC-MS trace (top), (ii) non-deconvoluted LC-MS trace (upper middle), (iii) deconvoluted MS data (lower middle, wide range), (iv) deconvoluted MS data (bottom, zoom in range).

9n

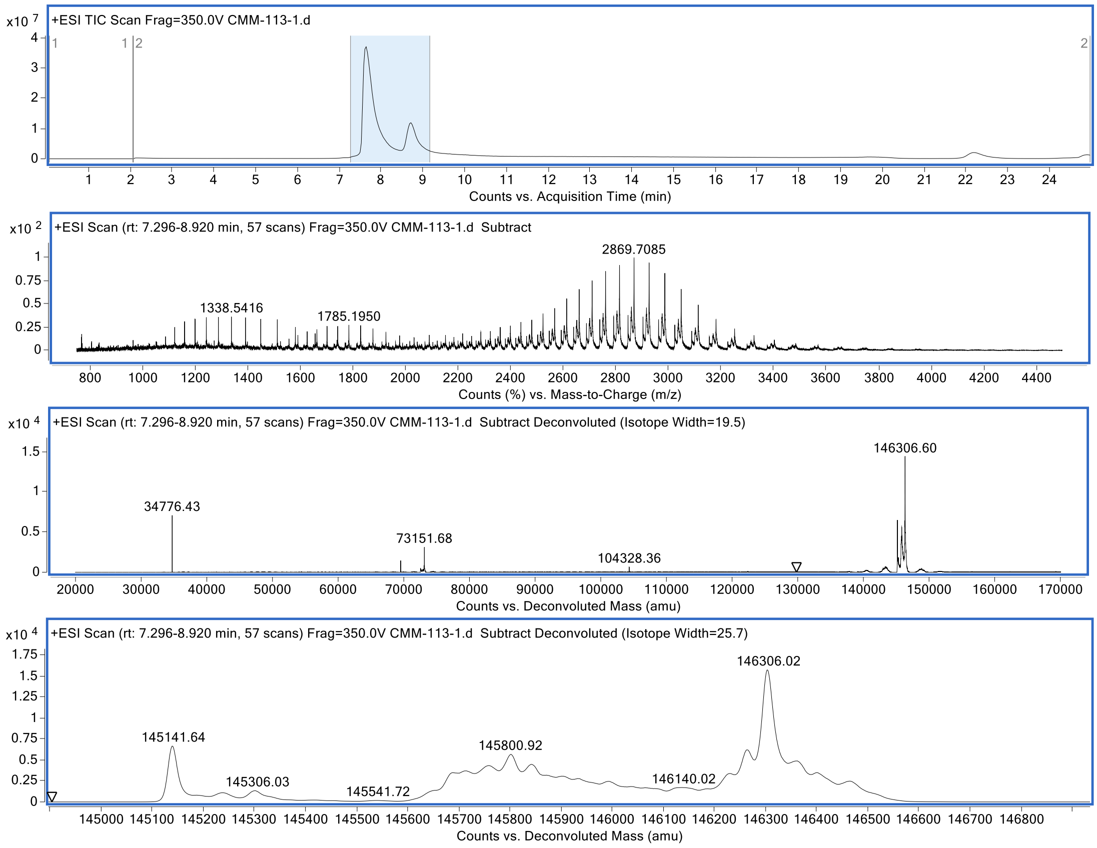

Figure S25: (i) TIC LC-MS trace (top), (ii) non-deconvoluted LC-MS trace (upper middle), (iii) deconvoluted MS data (lower middle, wide range), (iv) deconvoluted MS data (bottom, zoom in range).

9o

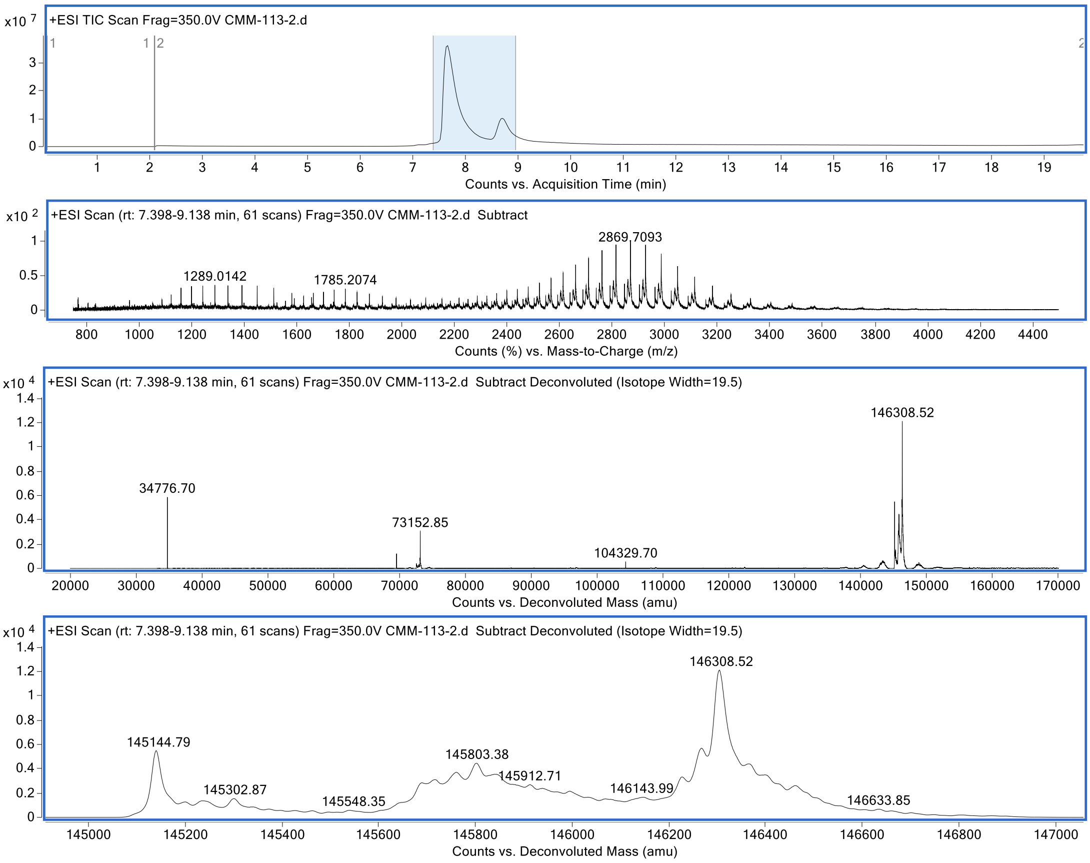

Figure S26: (i) TIC LC-MS trace (top), (ii) non-deconvoluted LC-MS trace (upper middle), (iii) deconvoluted MS data (lower middle, wide range), (iv) deconvoluted MS data (bottom, zoom in range).

9p

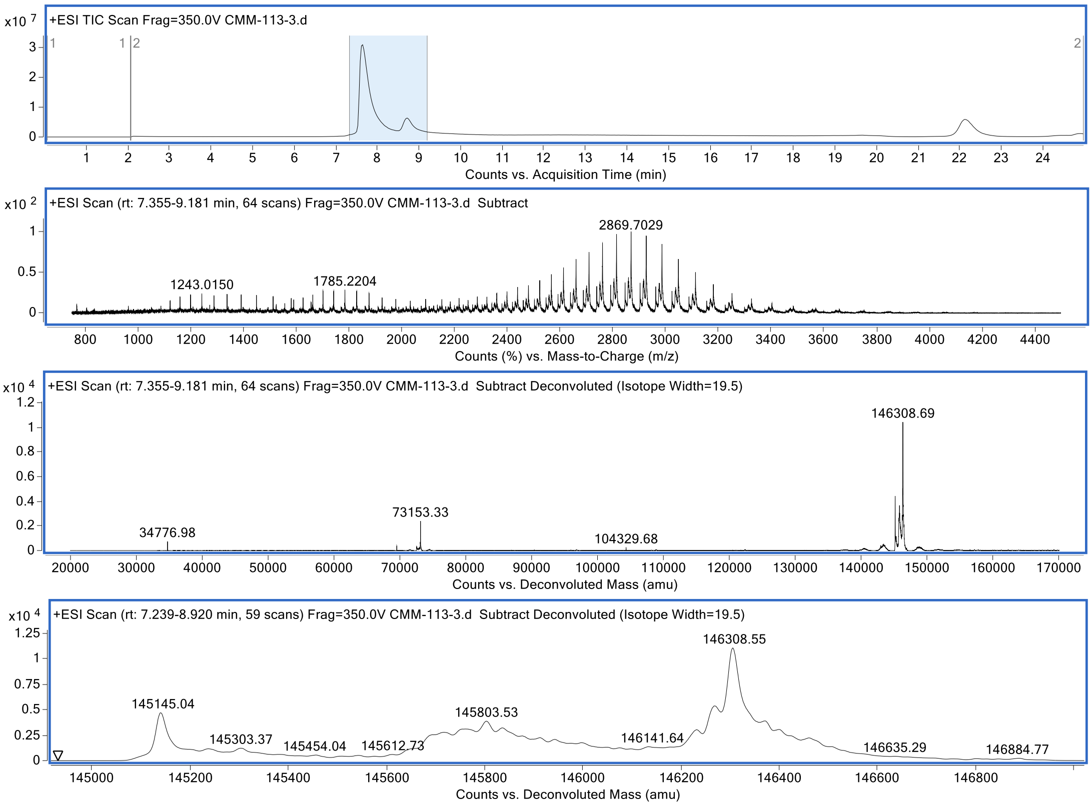

Figure S27: (i) TIC LC-MS trace (top), (ii) non-deconvoluted LC-MS trace (upper middle), (iii) deconvoluted MS data (lower middle, wide range), (iv) deconvoluted MS data (bottom, zoom in range).

9q

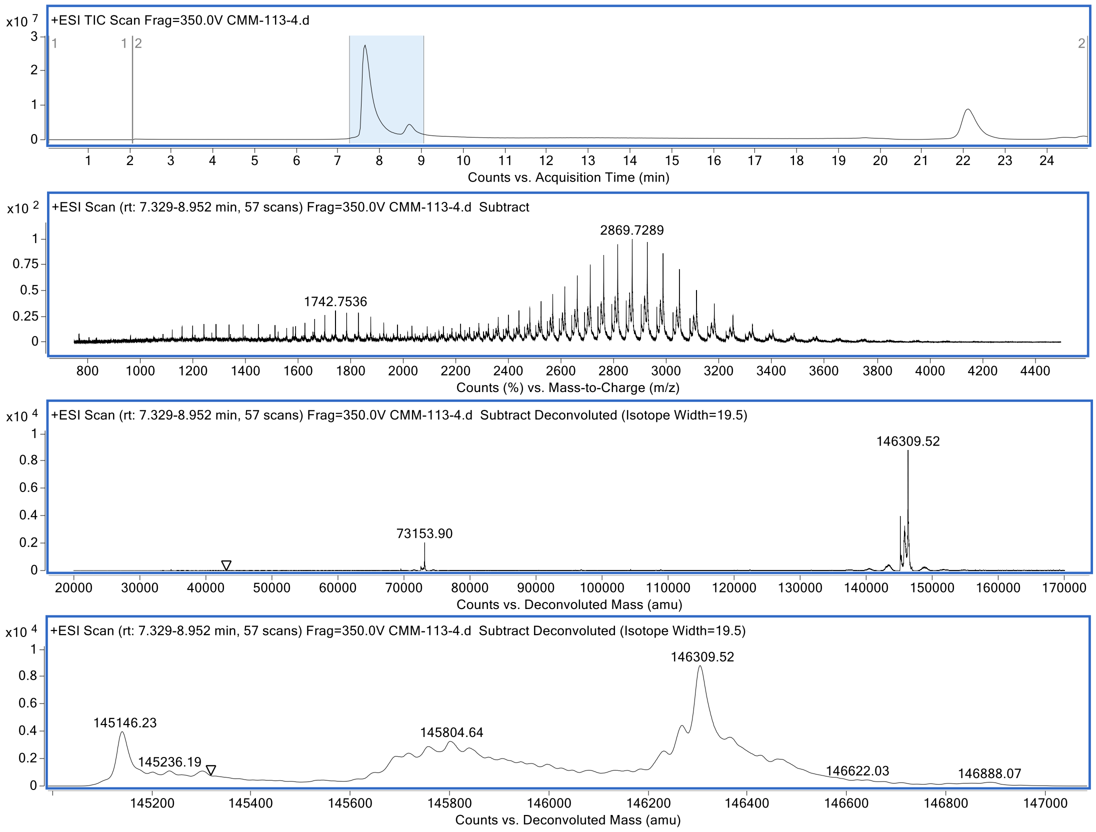

Figure S28: (i) TIC LC-MS trace (top), (ii) non-deconvoluted LC-MS trace (upper middle), (iii) deconvoluted MS data (lower middle, wide range), (iv) deconvoluted MS data (bottom, zoom in range).

9r

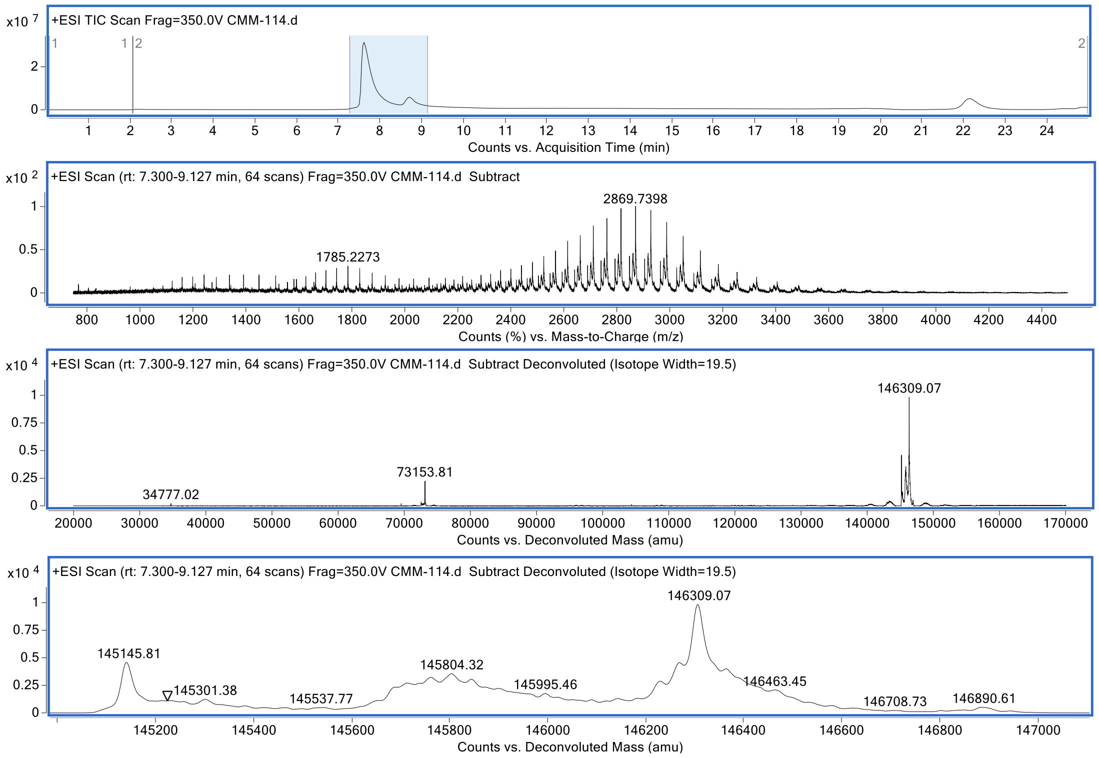

Figure S29: (i) TIC LC-MS trace (top), (ii) non-deconvoluted LC-MS trace (upper middle), (iii) deconvoluted MS data (lower middle, wide range), (iv) deconvoluted MS data (bottom, zoom in range).

9s

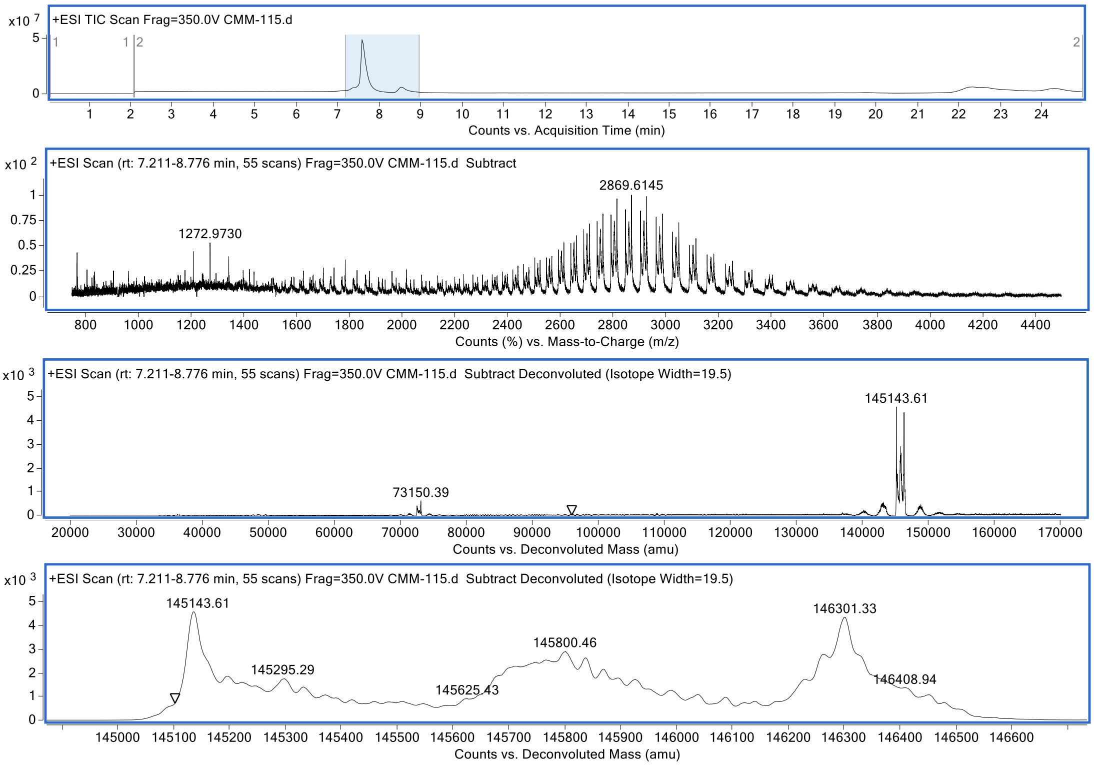

Figure S30: (i) TIC LC-MS trace (top), (ii) non-deconvoluted LC-MS trace (upper middle), (iii) deconvoluted MS data (lower middle, wide range), (iv) deconvoluted MS data (bottom, zoom in range).

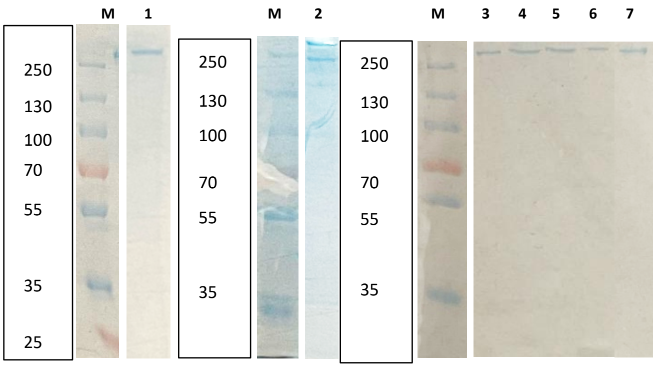

Figure S31: SDS-PAGE gel: M. Ladder, 1. Conjugate **9l**, 2. Conjugate **9m**, 3. Conjugate **9n**, 4. Conjugate **9o**, 5. Conjugate **9p**, 6. Conjugate **9q**, 7. Conjugate **9s**.

HC S378C NMM conjugate S15 (HC S378C Thio-Trastuzumab Conjugated to *N*-methylmaleimide)

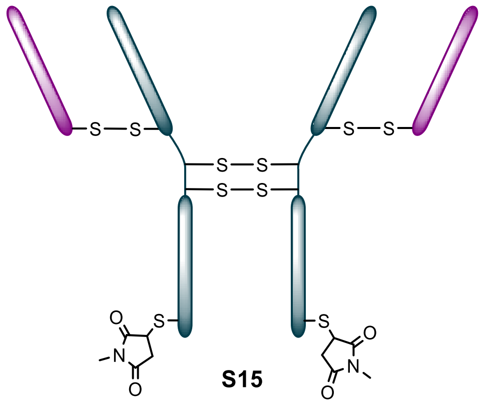

S15

To a solution of uncapped HC S378C thio-trastuzumab **7** (85 µL, 13.9 µM) in BBS (25 mM borate, 25 mM NaCl, 2 mM EDTA, 2% DMSO, pH 8.0) was added NMM (0.236 µL, 20 mM in DMSO, 4 eq.) and the reaction incubated at 22 °C for 3 h under constant agitation (300 rpm). After this time, excess reagents were removed to give conjugate **S15** (expected mass 145,365 Da, observed mass 145,367 Da) which was analysed by LC-MS (method 1b), UV-Vis spectroscopy and SDS-PAGE.

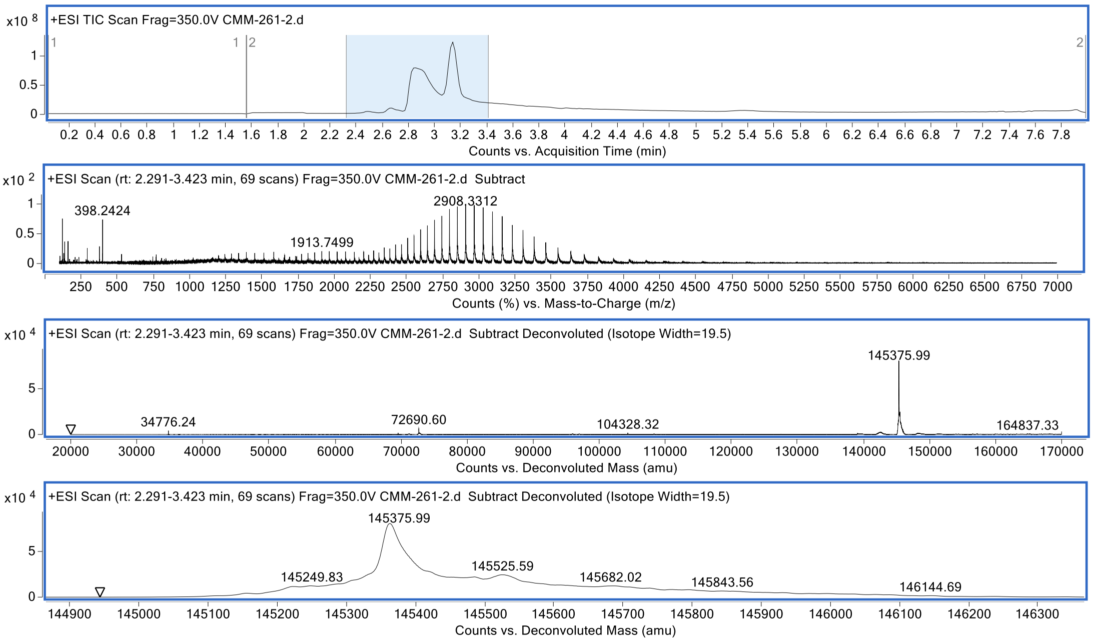

Figure S32: (i) TIC LC-MS trace (top), (ii) non-deconvoluted LC-MS trace (upper middle), (iii) deconvoluted MS data (lower middle, wide range), (iv) deconvoluted MS data (bottom, zoom in range).

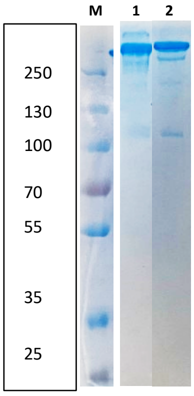

Figure S33: SDS-PAGE gel: M. Ladder, 1. Conjugate **7**, 2. Conjugate **S15**.

HC S378C PD conjugate 10 (HC S378C Thio-trastuzumab conjugated to BCN PD 1)

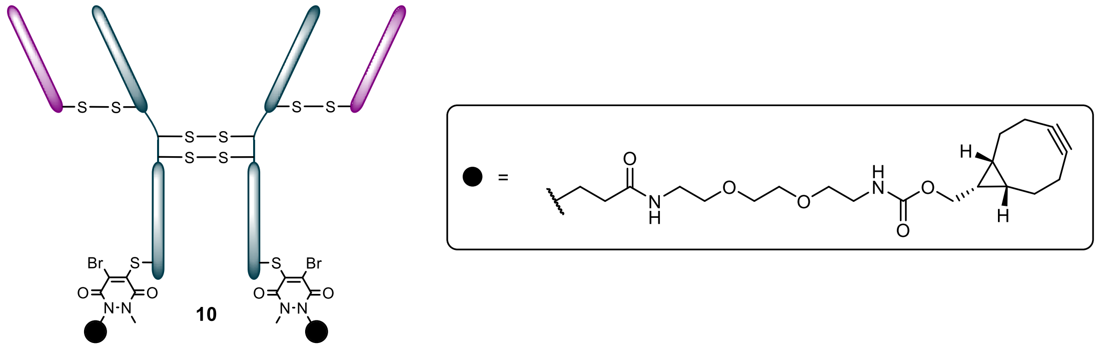

To a solution of uncapped HC S378C thio-trastuzumab **7** (100 µL, 20 µM) in A was added BCN PD **1** (20 mM in DMSO, B eq.) and the reaction incubated at C °C for D h under constant agitation (300 rpm). After this time, excess reagents were removed to give conjugate **10** (expected mass 146,309 Da, observed mass 146,302 Da) which was analysed by LC-MS (method 1a), UV-Vis spectroscopy and SDS-PAGE.

	Buffer (A)	BCN PD Eq. (B)	Incubation Temperature (C)	Reaction Time
**10a**	BBS, pH 8	4	22	3 h
**10b**	BBS, pH 8	5	37	3 h
**10c**	BBS, pH 8	5	37	16 h
**10d**	BBS, pH 8.5	5	37	3 h

Table S9. Conditions used to form **10**.

10a

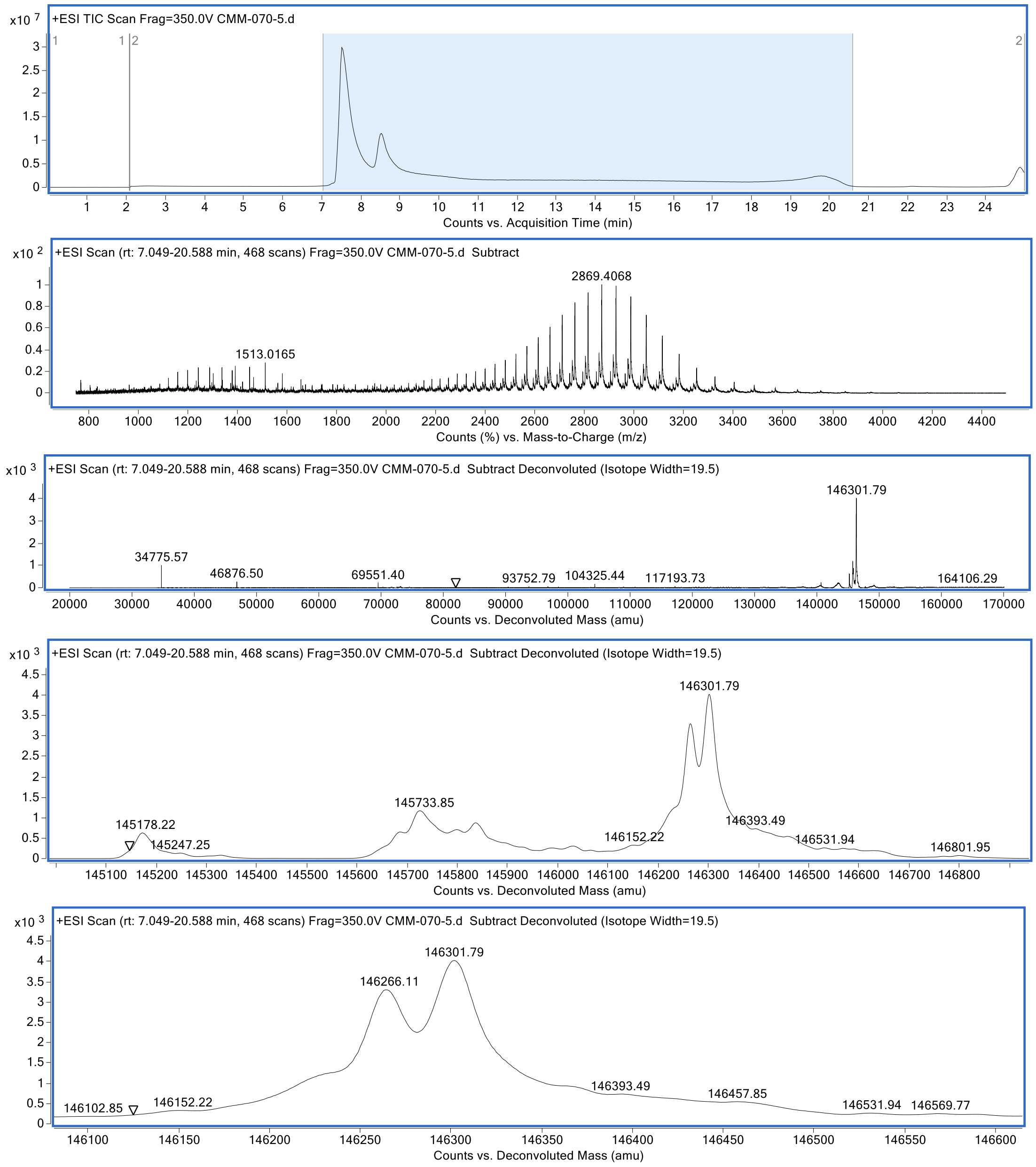

Figure S34: (i) TIC LC-MS trace (top), (ii) non-deconvoluted LC-MS trace (upper middle), (iii) deconvoluted MS data (lower middle, wide range), (iv) and (v) deconvoluted MS data (bottom, zoom in range).

10b

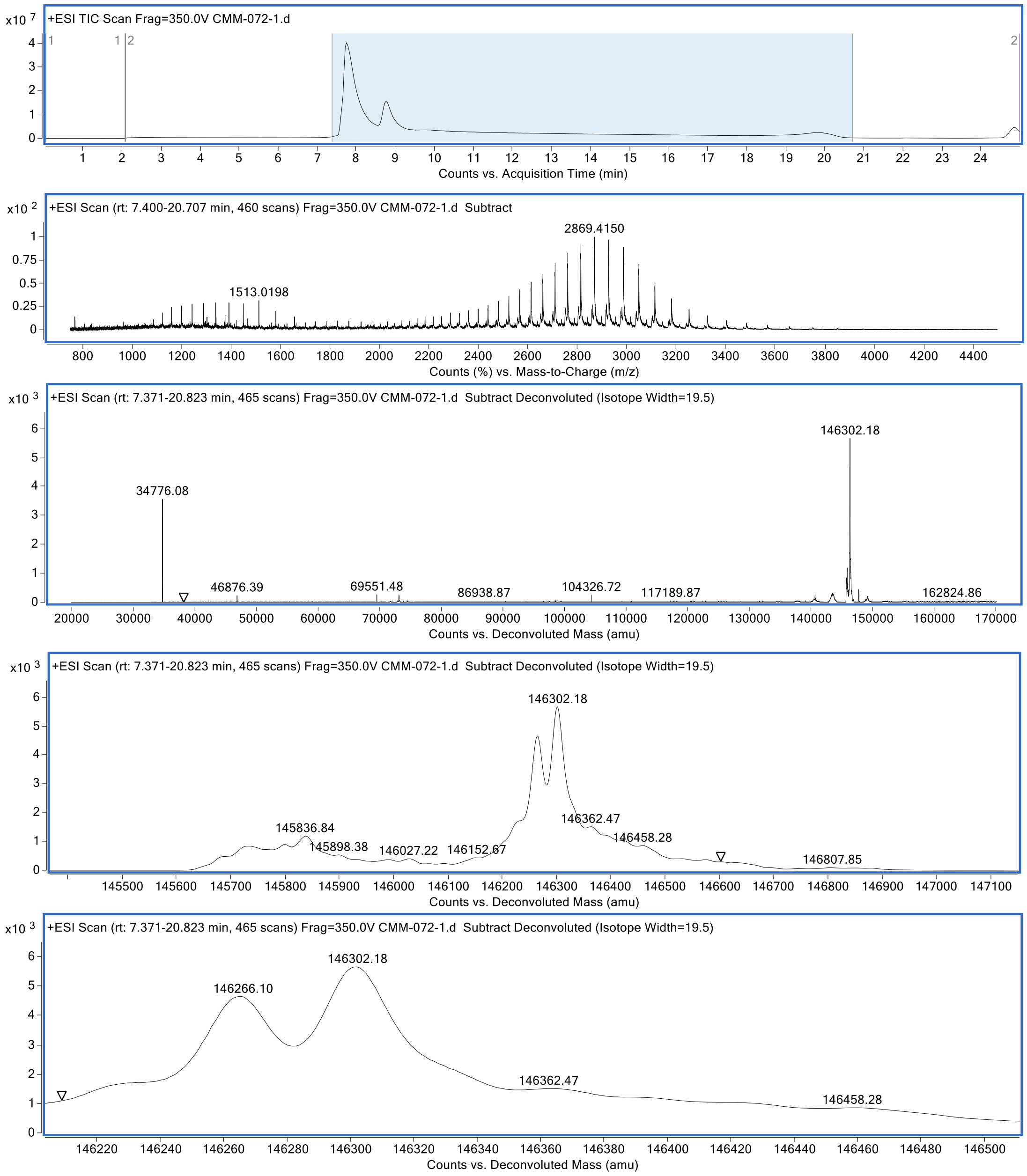

Figure S35: (i) TIC LC-MS trace (top), (ii) non-deconvoluted LC-MS trace (upper middle), (iii) deconvoluted MS data (lower middle, wide range), (iv) and (v) deconvoluted MS data (bottom, zoom in range).

10c

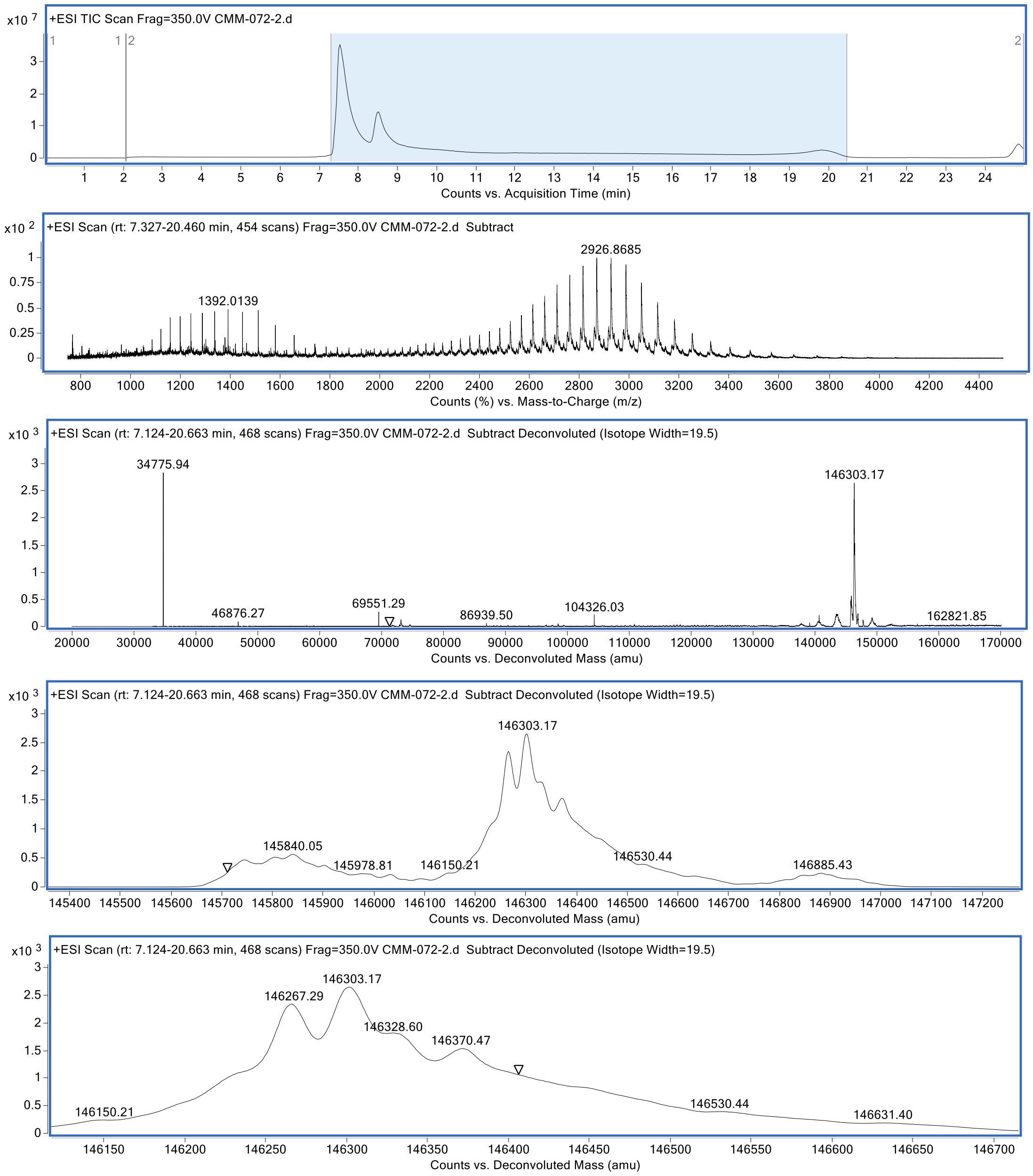

Figure S36: (i) TIC LC-MS trace (top), (ii) non-deconvoluted LC-MS trace (upper middle), (iii) deconvoluted MS data (lower middle, wide range), (iv) and (v) deconvoluted MS data (bottom, zoom in range).

10d

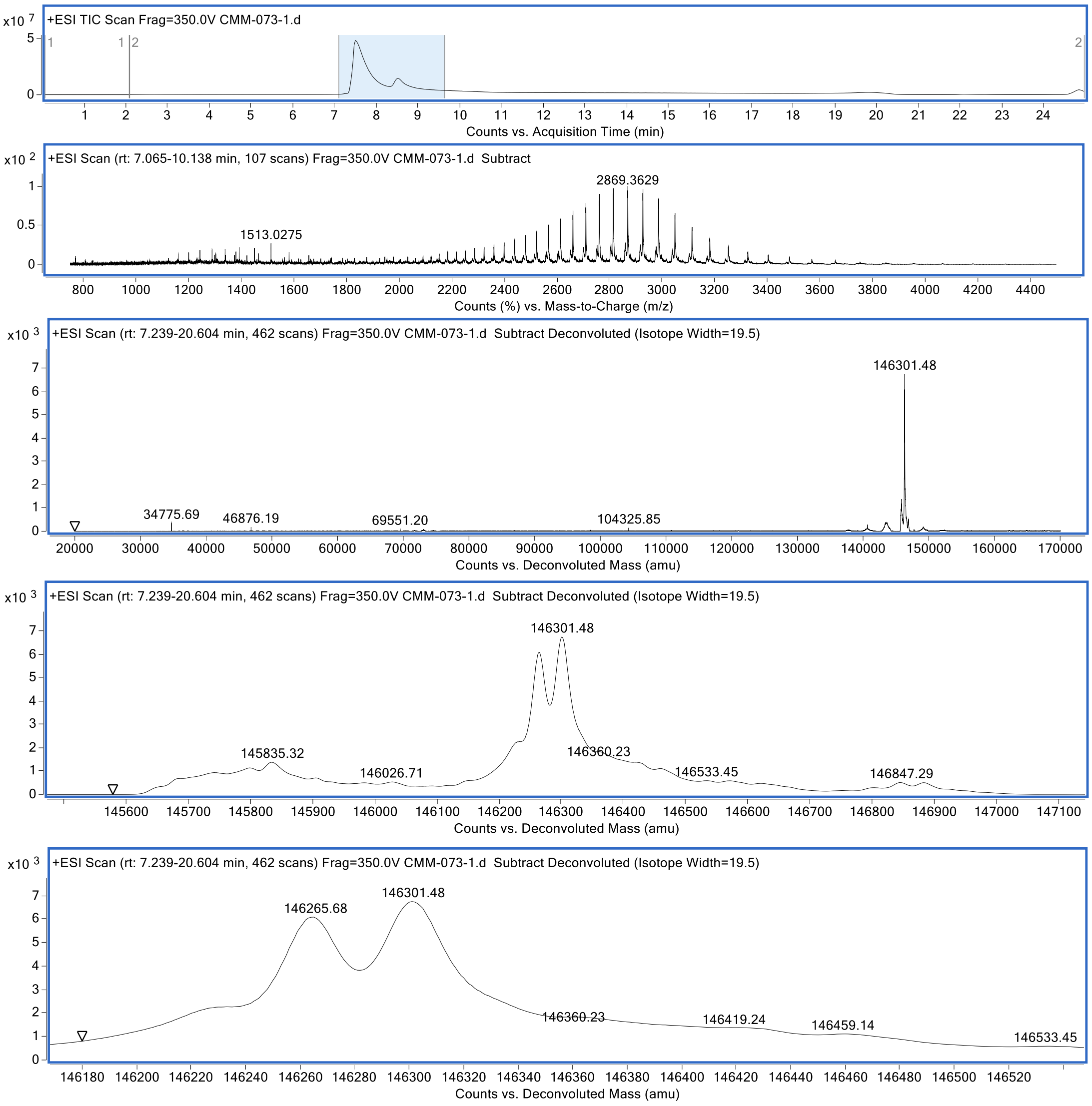

Figure S37: (i) TIC LC-MS trace (top), (ii) non-deconvoluted LC-MS trace (upper middle), (iii) deconvoluted MS data (lower middle, wide range), (iv) and (v) deconvoluted MS data (bottom, zoom in range).

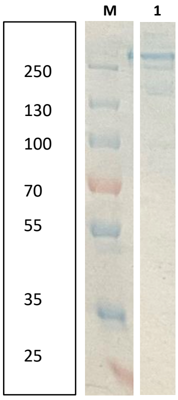

Figure S38: SDS-PAGE gel: M. Ladder, 1. Conjugate **10**

HC S378C PD fluorophore conjugate 11 (HC S378C Thio-trastuzumab conjugated to BCN PD 1, Clicked with Azide-fluor 488)

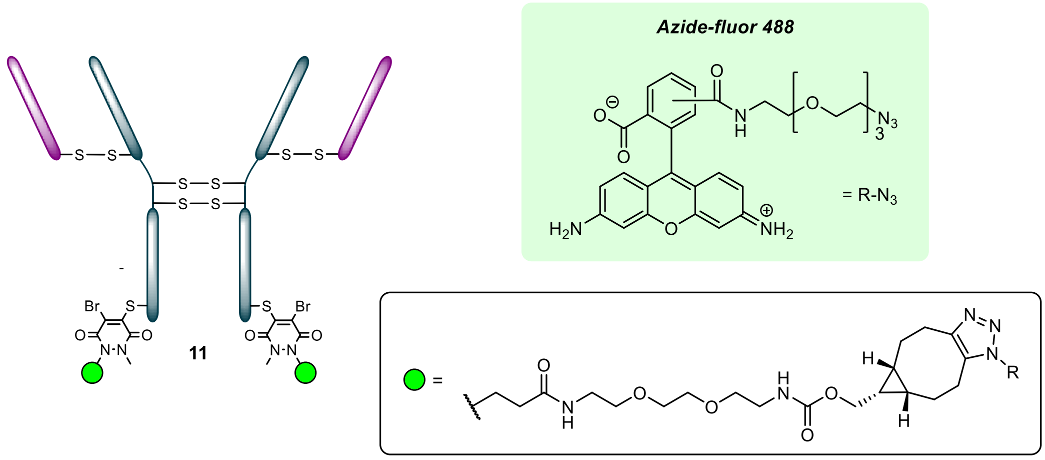

Method 1: Post Click

To a solution of uncapped HC S378C thio-trastuzumab conjugated to BCN PD **10** (50 µL, 17 µM) in BBS (25 mM borate, 25 mM NaCl, 2 mM EDTA, 2% DMSO, pH 8.0) was added Azide-fluor 488 (4.25 µL, 10 mM in DMSO, 5 eq.) and the reaction incubated at 37 °C for 16 h under constant agitation (300 rpm). After this time, excess reagents were removed to give conjugate **11** (expected mass 147,459 Da, observed mass 147,450 Da) which was analysed by LC-MS (method 1b), UV-Vis spectroscopy and SDS-PAGE.

Method 2: Pre Click

To BBS (18 µL, 25 mM borate, 25 mM NaCl, 2 mM EDTA, 2% DMSO, pH 8.0) was added BCN PD (2 µL, 10 mM DMSO) and Azide-fluor 488 (20 µL, 10 mM DMSO, 10x excess) and the reaction incubated at 37 °C for 20 h under constant agitation (300 rpm) under argon.

To a solution of uncapped HC S378C thio-trastuzumab **7** (160 µL, 25 µM) in BBS was added the BCN PD pre-clicked with Azide-fluor 488 (40 µL, 5 eq.) and the reaction incubated at 37 °C for 5 h under constant agitation (300 rpm). After this time, excess reagents were removed to give conjugate **11** (expected mass 147,459 Da, observed mass 147,459 Da) which was analysed by LC-MS (method 1b), UV-Vis spectroscopy and SDS-PAGE.

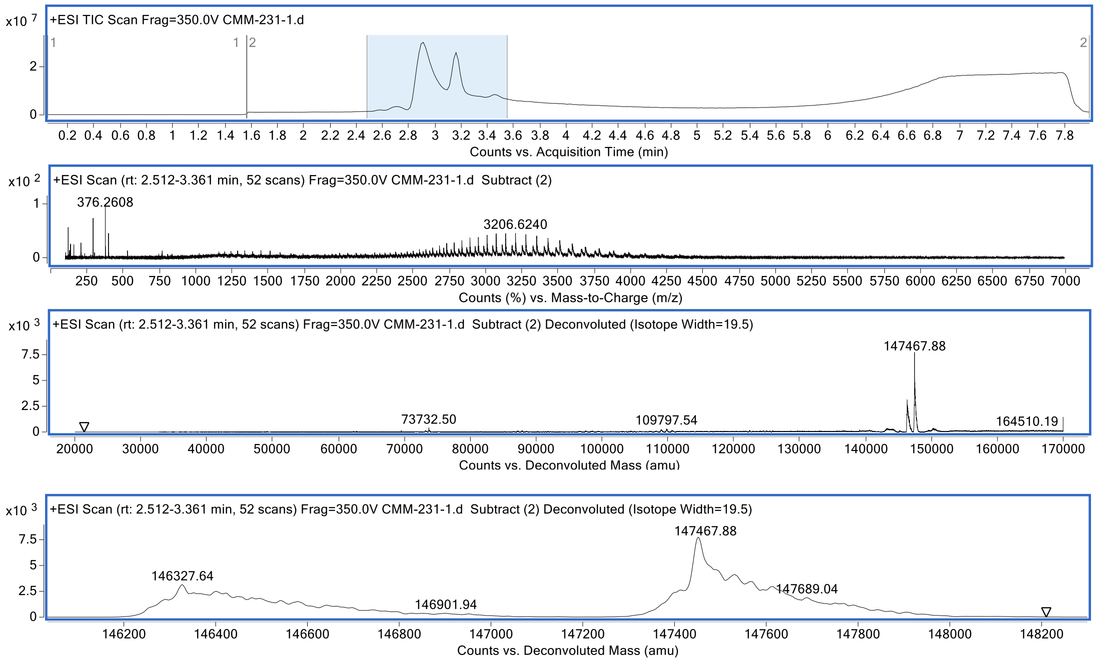

Figure S39: (i) TIC LC-MS trace (top), (ii) non-deconvoluted LC-MS trace (upper middle), (iii) deconvoluted MS data (lower middle, wide range), (iv) deconvoluted MS data (bottom, zoom in range).

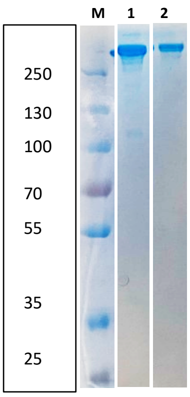

Figure S40: SDS-PAGE gel: M. Ladder, 1. Conjugate **10**, 2. Conjugate **11**

LC S168C PD fluorophore conjugate 12 (LC S168C thio-trastuzumab conjugated to BCN PD 1, Clicked with Azide-fluor 488)

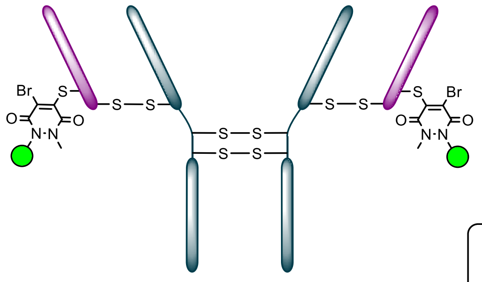

12

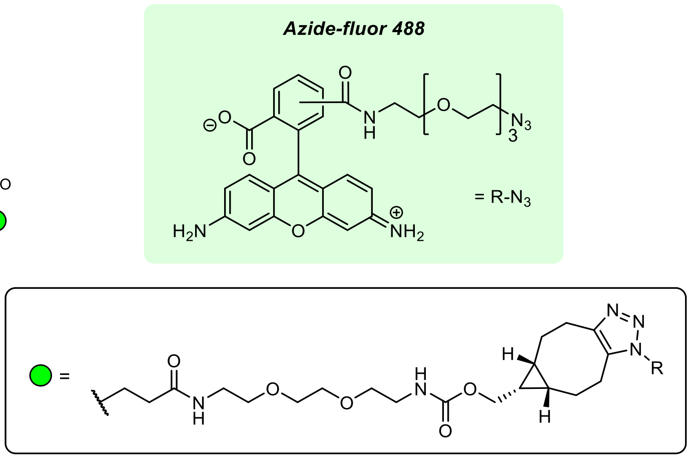

Method 1: Post Click

To a solution of uncapped LC S168C thio-trastuzumab conjugated to BCN PD **9a** (50 µL, 13.2 µM) in BBS (25 mM borate, 25 mM NaCl, 2 mM EDTA, 2% DMSO, pH 8.0) was added Azide-fluor 488 (0.66 µL, 10 mM in DMSO, 10 eq.) and the reaction incubated at 37 °C for 16 h under constant agitation (300 rpm). After this time, excess reagents were removed to give conjugate **12** (expected mass 147,459 Da, observed mass 147,459 Da) which was analysed by LC-MS (method 1b), UV-Vis spectroscopy and SDS-PAGE.

Method 2: Pre Click

To BBS (22.4 µL, 25 mM borate, 25 mM NaCl, 2 mM EDTA, 2% DMSO, pH 8.0) was added BCN PD (1.6 µL, 10 mM DMSO) and Azide-fluor 488 (16 µL, 10 mM DMSO, 10x excess) and the reaction incubated at 37 °C for 20 h under constant agitation (300 rpm) under argon.

To a solution of uncapped LC S168C thio-trastuzumab **8** (160 µL, 25 µM) in BBS was added the BCN PD pre-clicked with Azide-fluor 488 (40 µL, 4 eq.) and the reaction incubated at 37 °C for 5 h under constant agitation (300 rpm). After this time, excess reagents were removed to give conjugate **12** (expected mass 147,459 Da, observed mass 147,459 Da) which was analysed by LC-MS (method 1b), UV-Vis spectroscopy and SDS-PAGE.

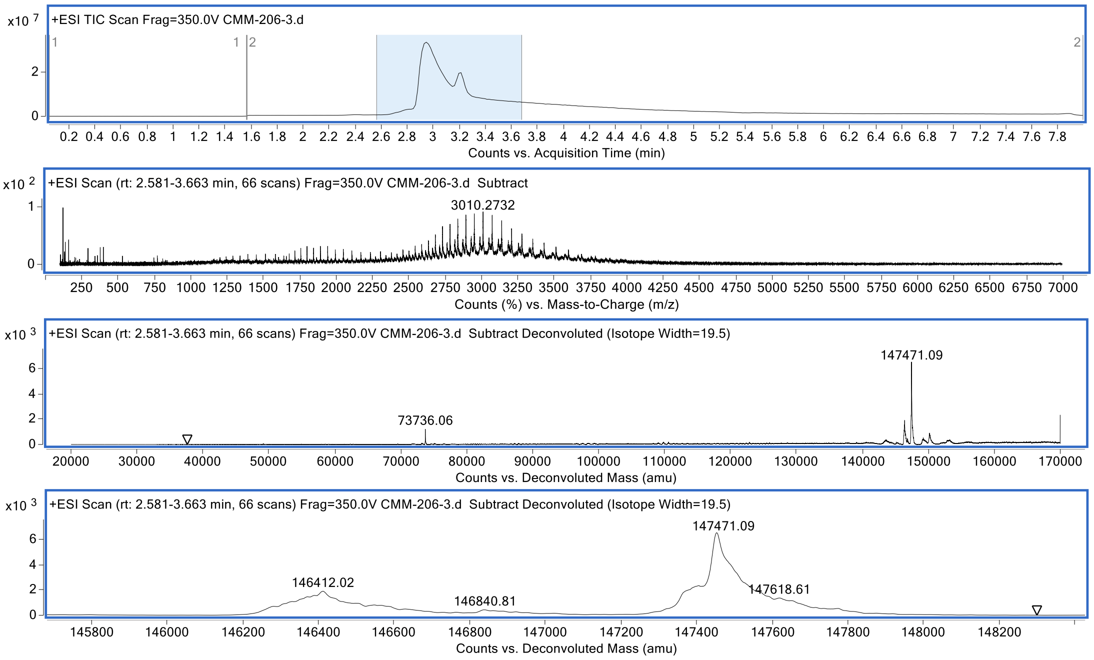

Figure S41: (i) TIC LC-MS trace (top), (ii) non-deconvoluted LC-MS trace (upper middle), (iii) deconvoluted MS data (lower middle, wide range), (iv) deconvoluted MS data (bottom, zoom in range).

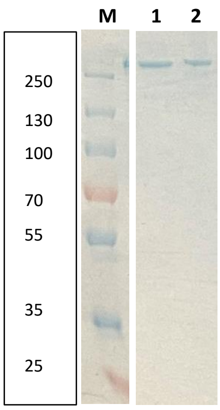

Figure S42: SDS-PAGE gel: M. Ladder, 1. Conjugate **9**, 2. Conjugate **12**.

HC S378C PD aniline fluorophore conjugate 13 (HC S378C Thio-trastuzumab conjugated to BCN PD 1, Azide-fluor 488 and N_3 Aniline 3)

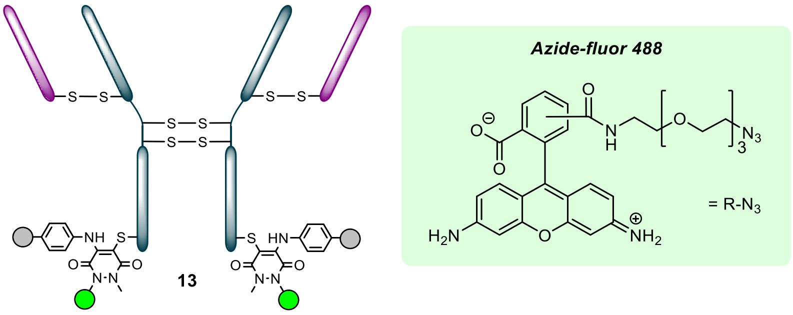

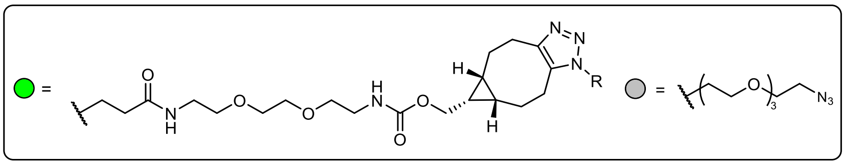

To a solution of conjugated HC S378C thio-trastuzumab **11** (40 µL, 20 µM) in BBS (25 mM borate, 25 mM NaCl, 2 mM EDTA, 2% DMSO, pH 8.0) was added N_3 Aniline **3** (1.6 µL, 0.5 M in DMSO, 1000 eq.) and the reaction incubated at 37 °C for 16 h under constant agitation (300 rpm). After this time, excess reagents were removed to give conjugate **13** (expected mass 147,917 Da, observed mass 147,915 Da) which was analysed by LC-MS (method 1b), UV-Vis spectroscopy and SDS-PAGE.

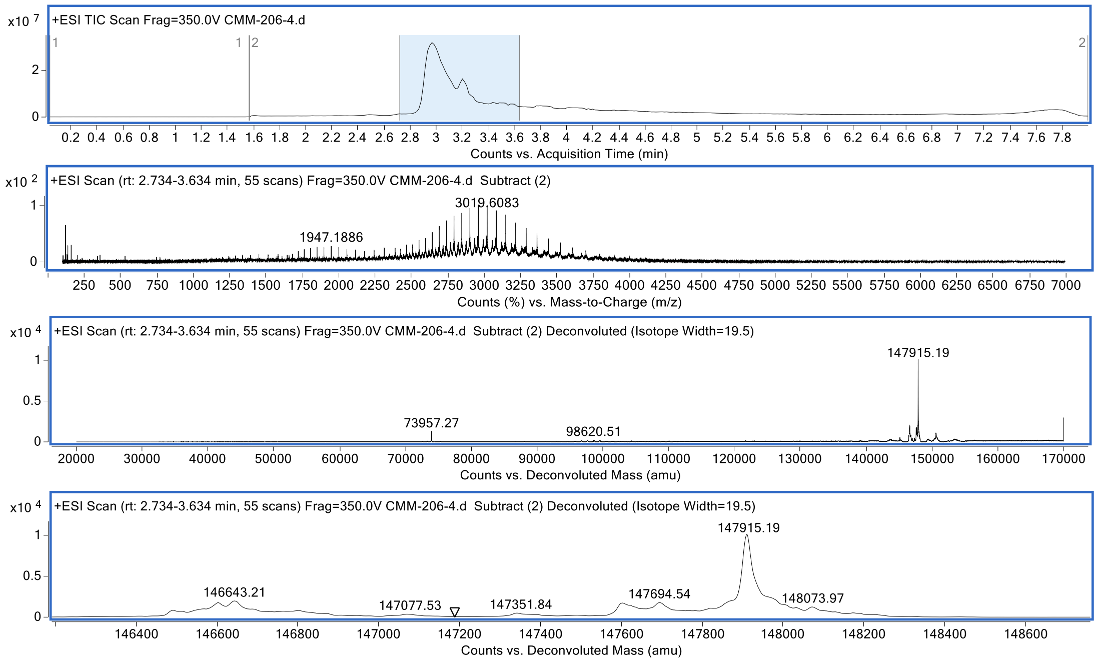

Figure S43: (i) TIC LC-MS trace (top), (ii) non-deconvoluted LC-MS trace (upper middle), (iii) deconvoluted MS data (lower middle, wide range), (iv) deconvoluted MS data (bottom, zoom in range).

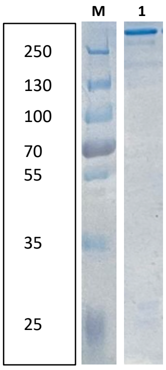

Figure S44: SDS-PAGE gel: M. Ladder, 1. Conjugate **13.**

LC S168C PD aniline fluorophore conjugate 14 (LC S168C thio-trastuzumab conjugated to BCN PD 1, Azide-fluor 488 and N_3 Aniline)

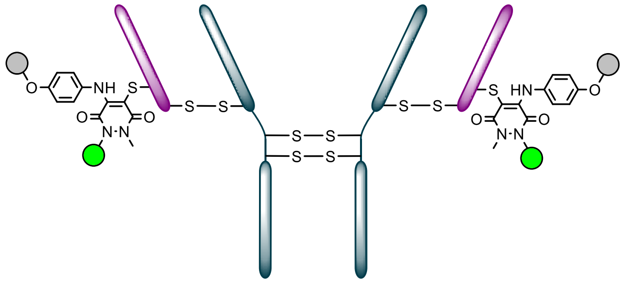

14

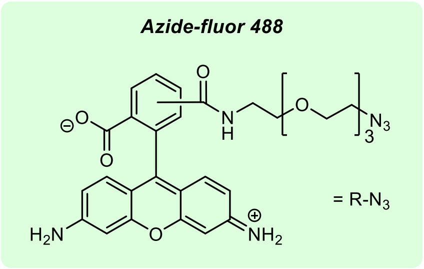

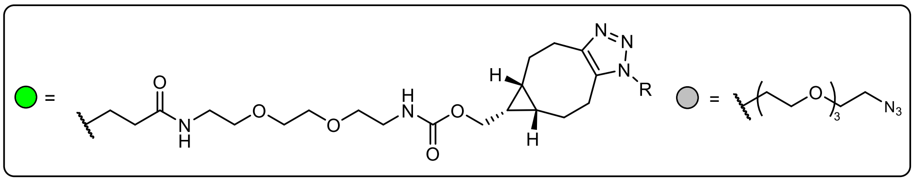

To a solution of conjugated LC S168C thio-trastuzumab **12** (190 µL, 11.9 µM) in BBS (25 mM borate, 25 mM NaCl, 2 mM EDTA, 2% DMSO, pH 8.0) was added N_3 Aniline (6.8 µL, 0.5 M in DMSO, 1500 eq.) and the reaction incubated at 37 °C for 16 h under constant agitation (300 rpm). After this time, excess reagents were removed to give conjugate **14** (expected mass 147,917 Da, observed mass 147,920 Da) which was analysed by LC-MS (method 1b), UV-Vis spectroscopy and SDS-PAGE.

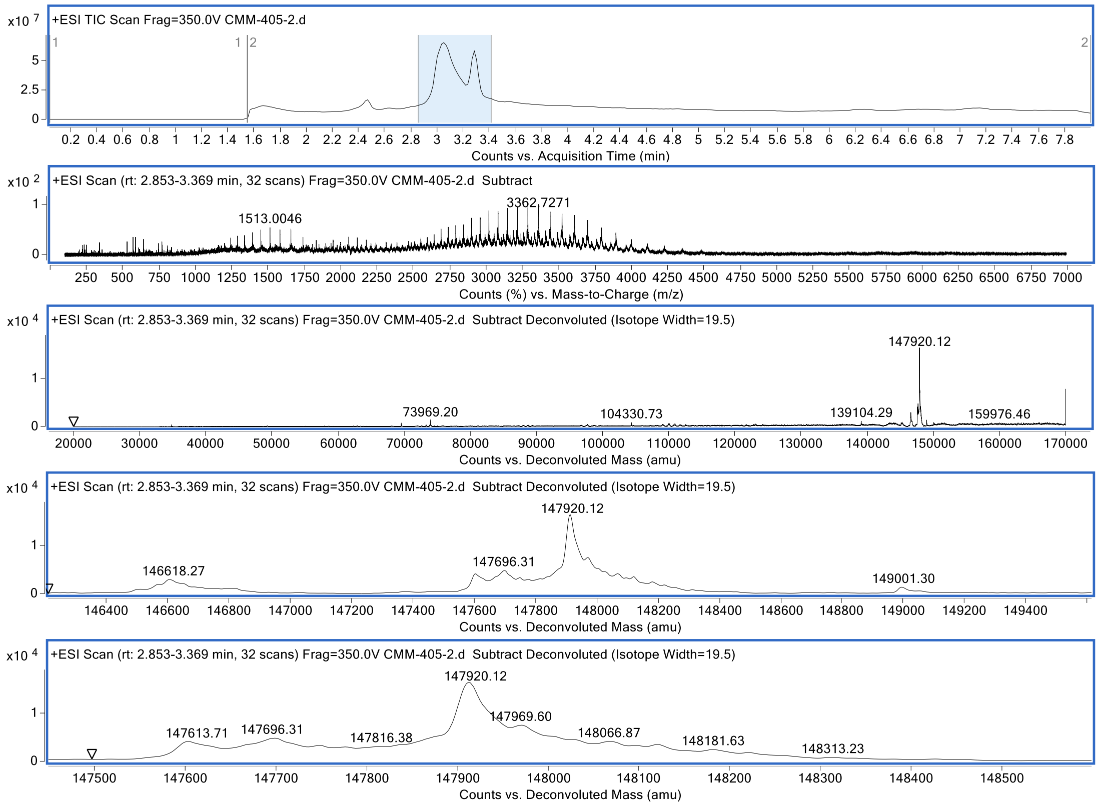

Figure S45: (i) TIC LC-MS trace (top), (ii) non-deconvoluted LC-MS trace (upper middle), (iii) deconvoluted MS data (lower middle, wide range), (iv) deconvoluted MS data (bottom, zoom in range).

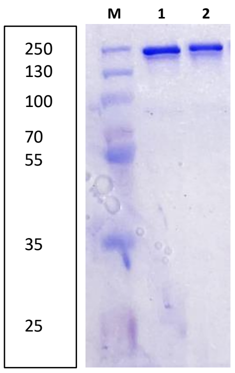

Figure S46: SDS-PAGE gel: M. Ladder, 1. Conjugate **12**, 2. Conjugate **14**.

LC S168C PD aniline fluorophore conjugate 15 (LC S168C thio-trastuzumab conjugated to BCN PD 1, Azide-fluor 488, N_3 Aniline and BP Fluor 568)

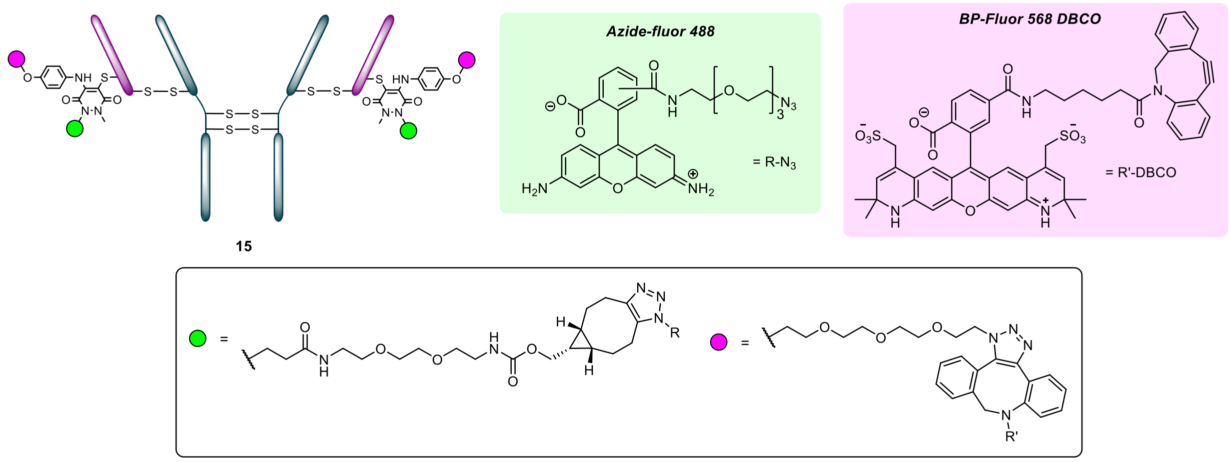

To a solution of conjugated LC S168C thio-trastuzumab **14** (110 µL, 14 µM) in BBS (25 mM borate, 25 mM NaCl, 2 mM EDTA, 2% DMSO, pH 8.0) was added BP Fluor 568 DBCO (3.1 µL, 20 mM in DMSO, 40 eq.) and the reaction incubated at 37 °C for 16 h under constant agitation (300 rpm). After this time, excess reagents were removed to give conjugate **15** (expected mass 149,824 Da, observed mass 149,825 Da) which was analysed by LC-MS (method 1b), UV-Vis spectroscopy and SDS-PAGE.

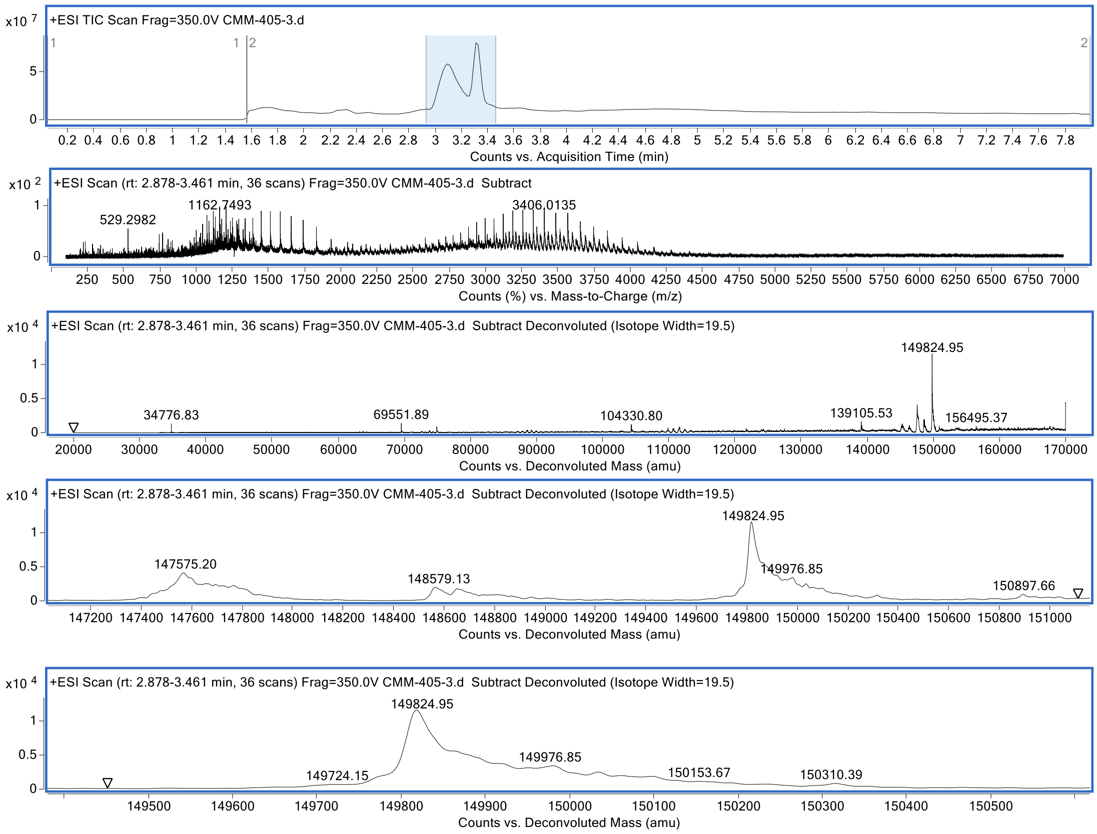

Figure S47: (i) TIC LC-MS trace (top), (ii) non-deconvoluted LC-MS trace (upper middle), (iii) deconvoluted MS data (lower middle, wide range), (iv) deconvoluted MS data (bottom, zoom in range).

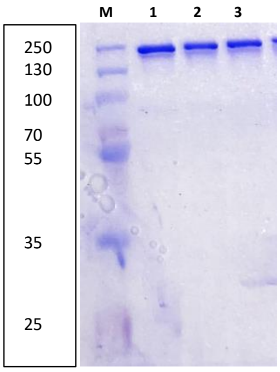

Figure S48: SDS-PAGE gel: M. Ladder, 1. Conjugate **12**, 2. Conjugate **14**, 3. Conjugate **15**.

HC S378C bisPD conjugate S16 (HC S378C thio-trastuzumab conjugated to diMe bisPD 5)

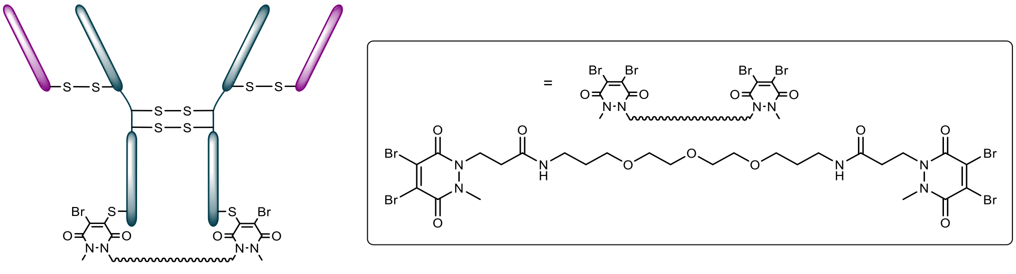

S16

To a solution of HC S378C thio-trastuzumab **7** (30 µL, 20 µM) in BBS (25 mM borate, 25 mM NaCl, 2 mM EDTA, 2% DMSO, pH 8.0) was added diMe bisPD **5** (0.37 µL, 8.2 mM in MeCN, 5 eq.) and the reaction incubated at 37 °C for 3 h under constant agitation (300 rpm). After this, excess reagents were removed to give conjugate **S16** (expected mass 145,879 Da, observed mass 145,880 Da) which was analysed by LC-MS (method 1a), UV-Vis spectroscopy and SDS-PAGE.

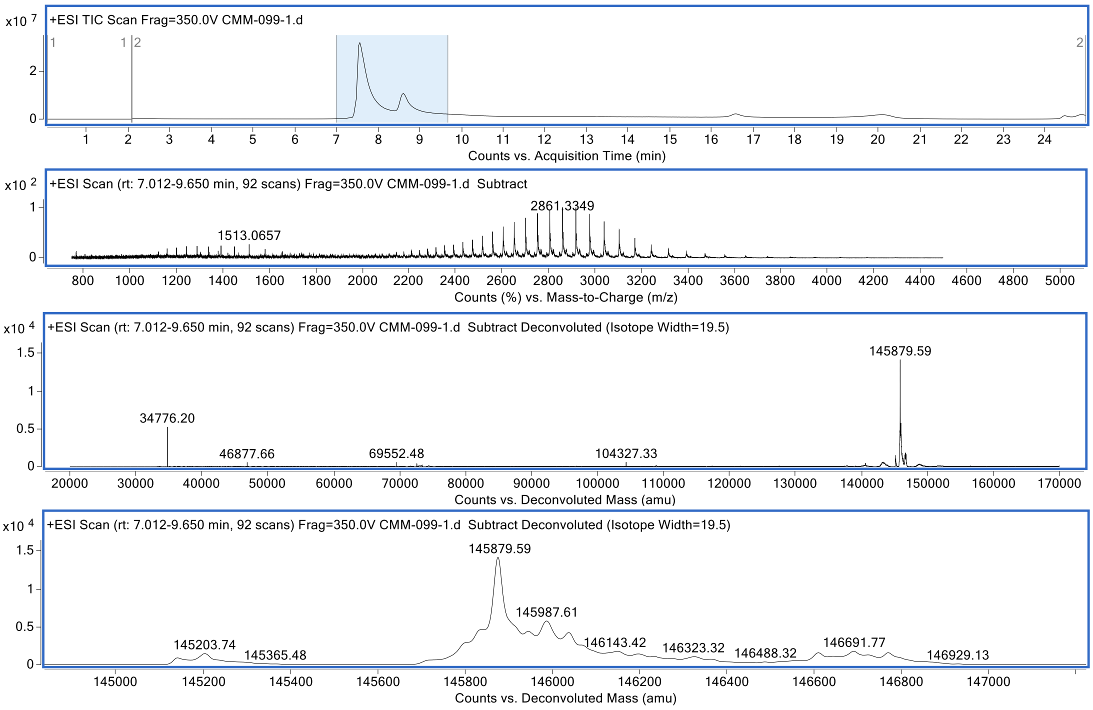

Figure S49: (i) TIC LC-MS trace (top), (ii) non-deconvoluted LC-MS trace (upper middle), (iii) deconvoluted MS data (lower middle, wide range), (iv) deconvoluted MS data (bottom, zoom in range).

Residue Distance Analysis

(a)

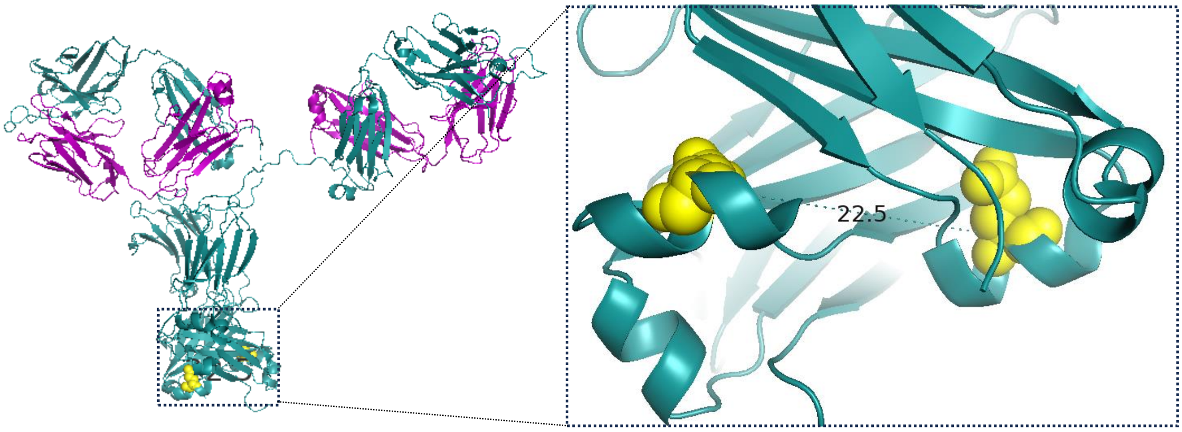

(b)

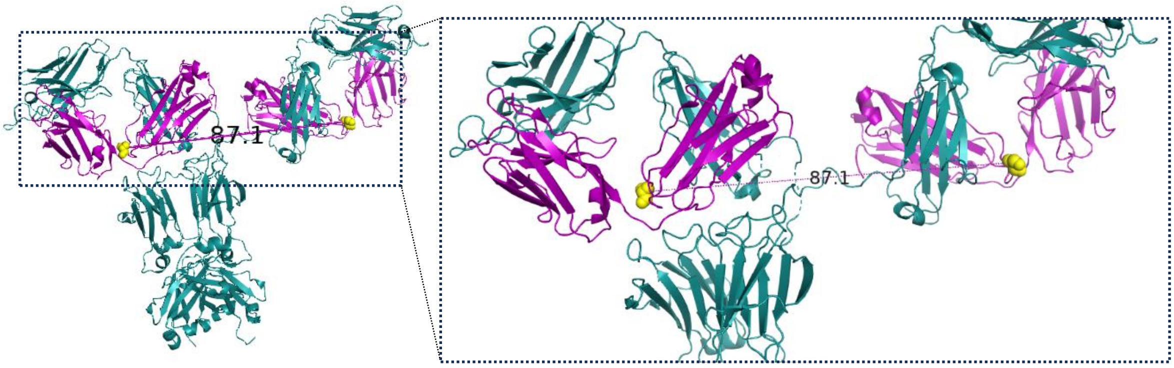

(c)

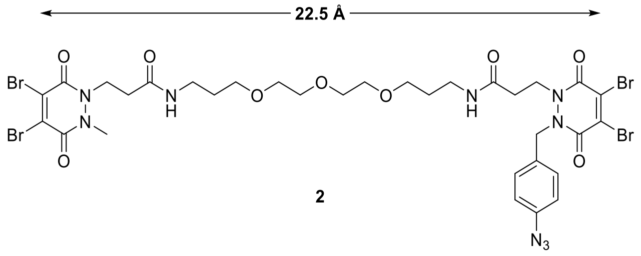

Figure S50. (a) The distance between the two HC residue 378s. Measurement was done on PyMol using the 1HZH file (human IgG1 b12) as there is no available crystal structure for full trastuzumab. (b) The distance between the two LC residue 168s. Measurement was done on PyMol using the 1HZH file (human IgG1 b12) as there is no available crystal structure for full trastuzumab. (c) PhN_3 bisPD **2** length, measured from carbon to carbon using MolView.

HC S378C bisPD aniline conjugate S17 (HC S378C thio-trastuzumab conjugated to diMe bisPD 5, reacted with *p*-Anisidine)

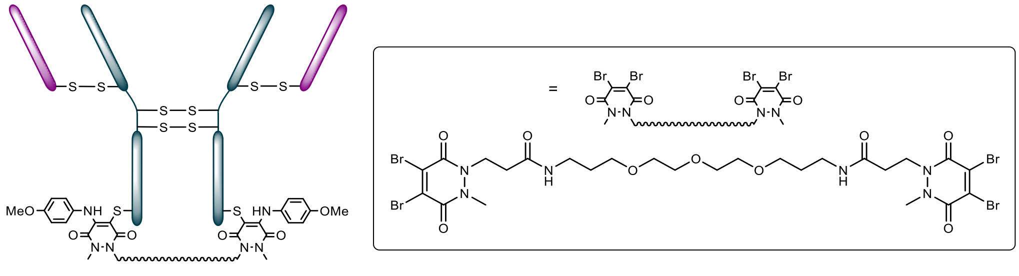

S17

To a solution of conjugated HC S378C thio-trastuzumab **S16** (21.4 µL, 20 µM) in BBS (25 mM borate, 25 mM NaCl, 2 mM EDTA, 2% DMSO, pH 8.0) was added *p*-anisidine (0.43 µL, 1 M in DMSO, 1000 eq.) and the reaction incubated at 37 °C for 16 h under constant agitation (300 rpm). After this, excess reagents were removed to give conjugate **S17** (expected mass 145,964 Da, observed mass 145,968 Da) which was analysed by LC-MS (method 1a), UV-Vis spectroscopy and SDS-PAGE.

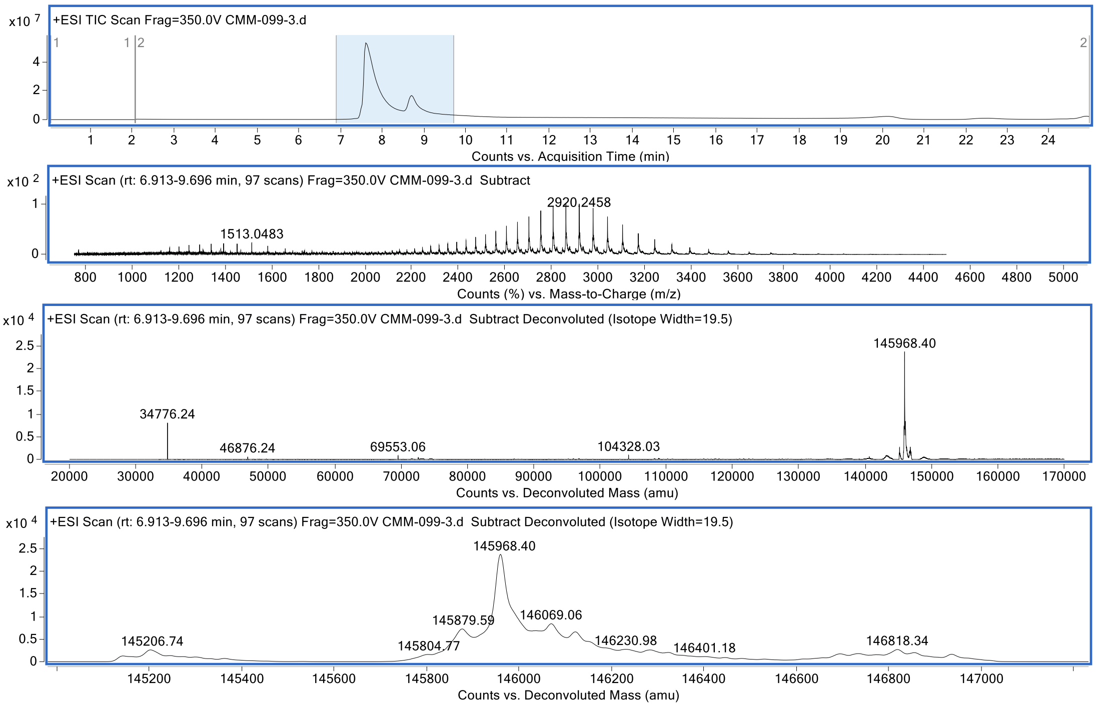

Figure S51: (i) TIC LC-MS trace (top), (ii) non-deconvoluted LC-MS trace (upper middle), (iii) deconvoluted MS data (lower middle, wide range), (iv) deconvoluted MS data (bottom, zoom in range).

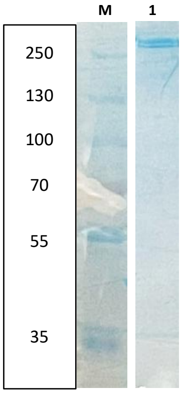

Figure S52: SDS-PAGE gel: M. Ladder, 1. Conjugate **S17**.

HC S378C bisPD conjugate 16 (HC S378C thio-trastuzumab conjugated to ArN_3 bisPD 2)

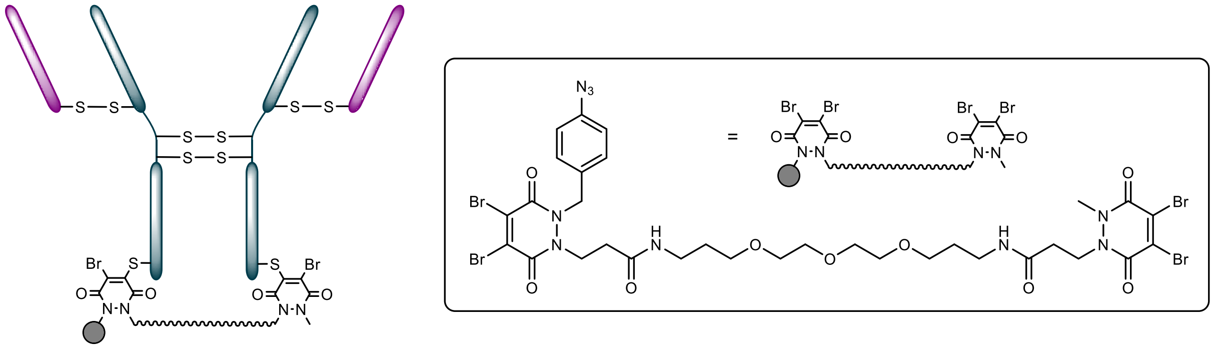

16

To a solution of HC S378C thio-trastuzumab **7** (200 µL, 20 µM) in BBS (25 mM borate, 25 mM NaCl, 2 mM EDTA, 2% DMSO, pH 8.0) was added ArN_3 bisPD **2** (1 µL, 20 mM in DMSO, 5 eq.) and the reaction incubated at 37 °C for 3 h under constant agitation (300 rpm). After this time, excess reagents were removed to give conjugate **16** (expected mass 145,997 Da, observed mass 146,007 Da) which was analysed by LC-MS (method 1b), UV-Vis spectroscopy and SDS-PAGE.

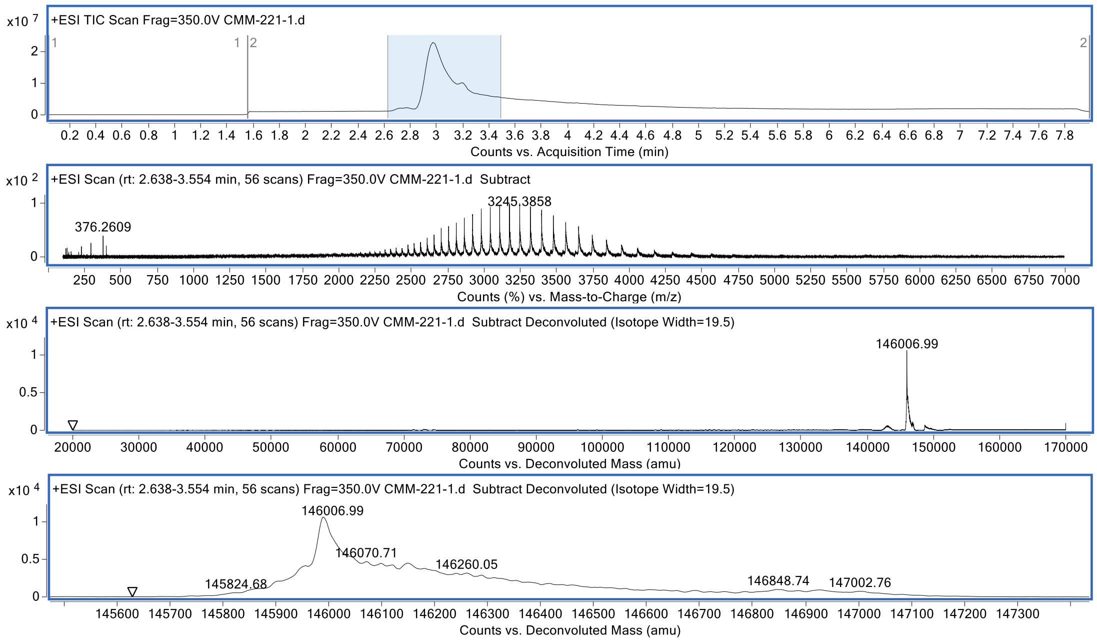

Figure S53: (i) TIC LC-MS trace (top), (ii) non-deconvoluted LC-MS trace (upper middle), (iii) deconvoluted MS data (lower middle, wide range), (iv) deconvoluted MS data (bottom, zoom in range).

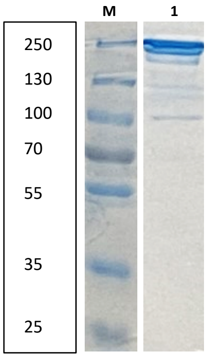

Figure S54: SDS-PAGE gel: M. Ladder, 1. Conjugate **16**.

HC S378C bisPD fluorophore conjugate 17 (HC S378C thio-trastuzumab conjugated to ArN_3 bisPD 2 and BP Fluor 568)

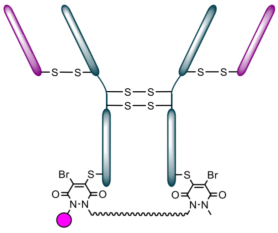

17

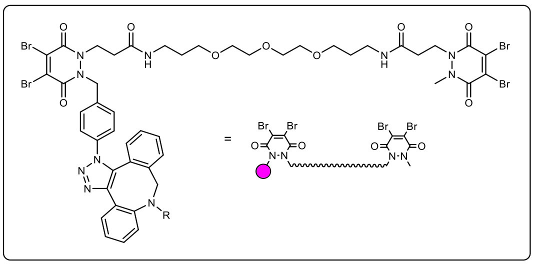

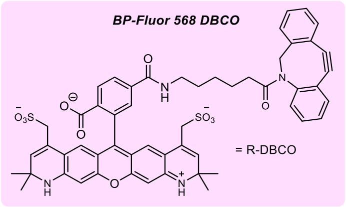

Pre-Click Method

To BBS (9 µL, 25 mM borate, 25 mM NaCl, 2 mM EDTA, 2% DMSO, pH 8.0) was added ArN3 bisPD **2** (1 µL, 20 mM DMSO) and BP Fluor 568 DBCO (10 µL, 20 mM DMSO, 10x excess) and the reaction incubated at 37 °C for 16 h under constant agitation (300 rpm) under argon.

To a solution of uncapped HC S378C thio-trastuzumab **7** (180 µL, 22.2 µM) in BBS was added the ArN_3 bisPD clicked with BP Fluor 568 (20 µL, 5 eq.) and the reaction incubated at 37 °C for 5 h under constant agitation (300 rpm). After this time, excess reagents were removed to give conjugate **17** (expected mass 146,950 Da, observed mass 146,962 Da) which was analysed by LC-MS (method 1b), UV-Vis spectroscopy and SDS-PAGE.

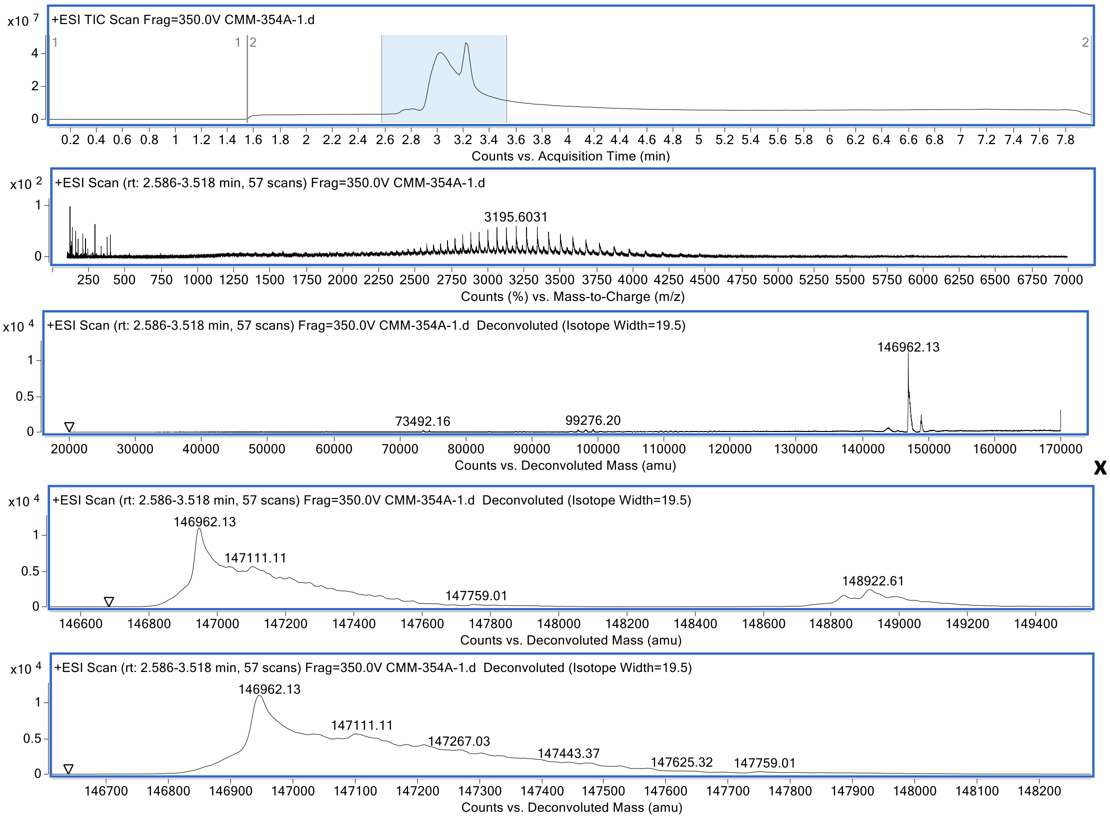

Figure S55: (i) TIC LC-MS trace (top), (ii) non-deconvoluted LC-MS trace (upper middle), (iii) deconvoluted MS data (lower middle, wide range), (iv) deconvoluted MS data (bottom, zoom in range).

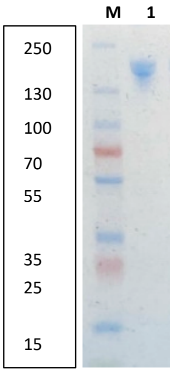

Figure S56: SDS-PAGE gel: M. Ladder, 1. Conjugate **17**.

HC S378C bisPD fluorophore conjugate S18 (HC S378C Thio-trastuzumab conjugated to ArN$_3$ bisPD 2 and DBCO biotin)

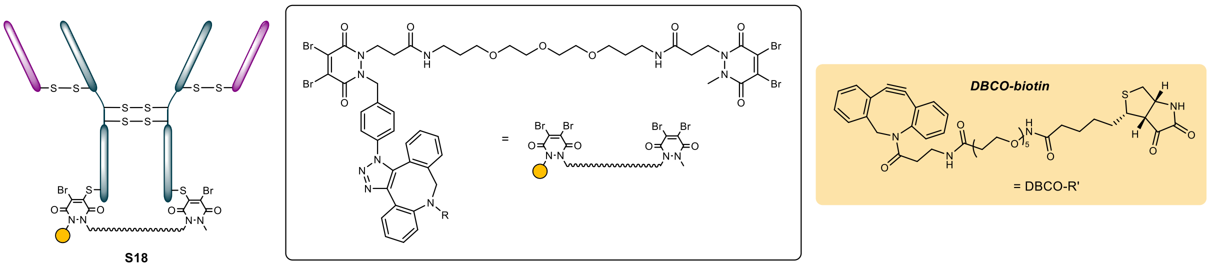

Pre-Click Method

To BBS (9 µL, 25 mM borate, 25 mM NaCl, 2 mM EDTA, 2% DMSO, pH 8.0) was added ArN3 bisPD **2** (1 µL, 20 mM DMSO) and DBCO biotin (10 µL, 20 mM DMSO, 10x excess) and the reaction incubated at 37 °C for 16 h under constant agitation (300 rpm) under argon.

To a solution of uncapped HC S378C thio-trastuzumab **7** (180 µL, 22.2 µM) in BBS was added the ArN$_3$ bisPD clicked with DBCO biotin (20 µL, 5 eq.) and the reaction incubated at 37 °C for 5 h under constant agitation (300 rpm). After this time, excess reagents were removed to give conjugate **S18** (expected mass 146,746 Da, observed mass 146,760 Da) which was analysed by LC-MS (method 1b), UV-Vis spectroscopy and SDS-PAGE.

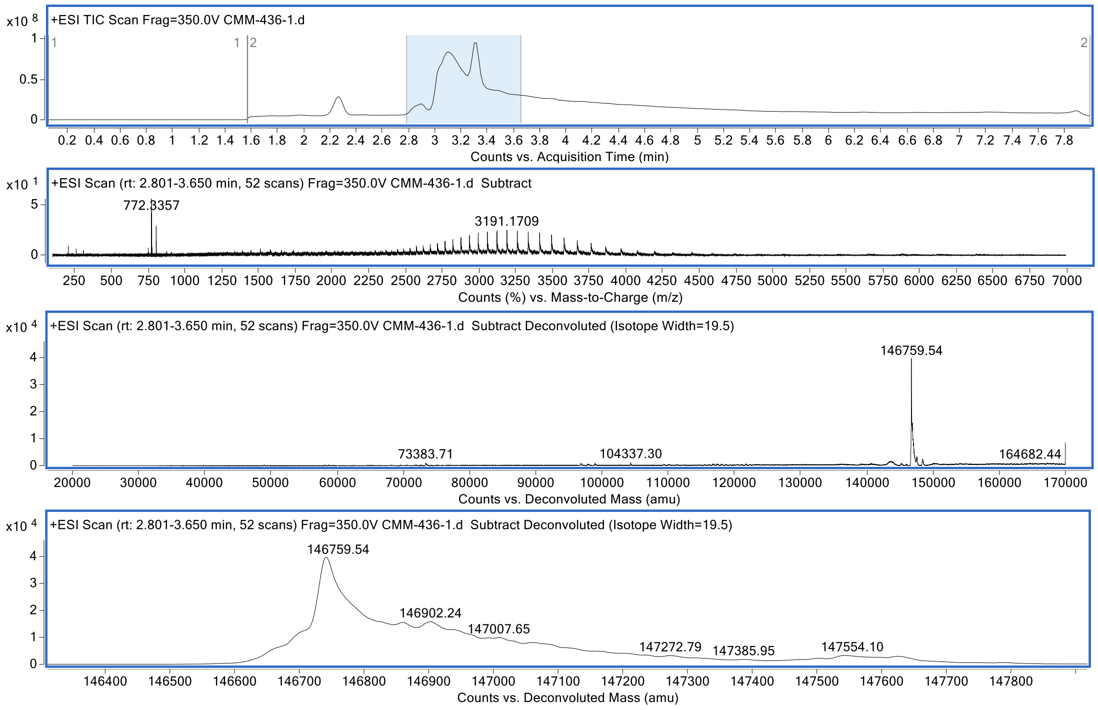

Figure S57: (i) TIC LC-MS trace (top), (ii) non-deconvoluted LC-MS trace (upper middle), (iii) deconvoluted MS data (lower middle, wide range), (iv) deconvoluted MS data (bottom, zoom in range).

HC S378C bisPD aniline fluorophore conjugate 18 (HC S378C thio-trastuzumab conjugated to ArN$_3$ bisPD 2, BP Fluor 568 and N$_3$ Aniline 3)

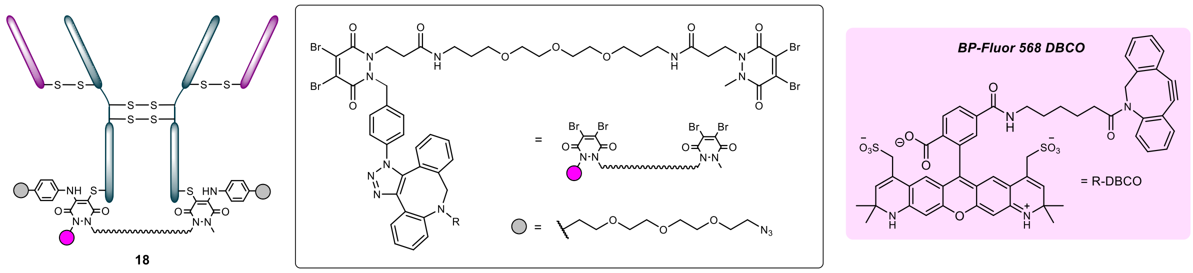

To a solution of conjugated HC S378C thio-trastuzumab **17** (180 µL, 20 µM) in BBS (25 mM borate, 25 mM NaCl, 2 mM EDTA, 2% DMSO, pH 8.0) was added N$_3$ aniline **3** (7.2 µL, 0.5 M in DMSO, 1000 eq.) and the reaction incubated at 37 °C for 16 h under constant agitation (300 rpm). After this, excess reagents were removed to give conjugate **18** (expected mass 147,411 Da, observed mass 147,433 Da) which was analysed by LC-MS (method 1b), UV-Vis spectroscopy and SDS-PAGE.

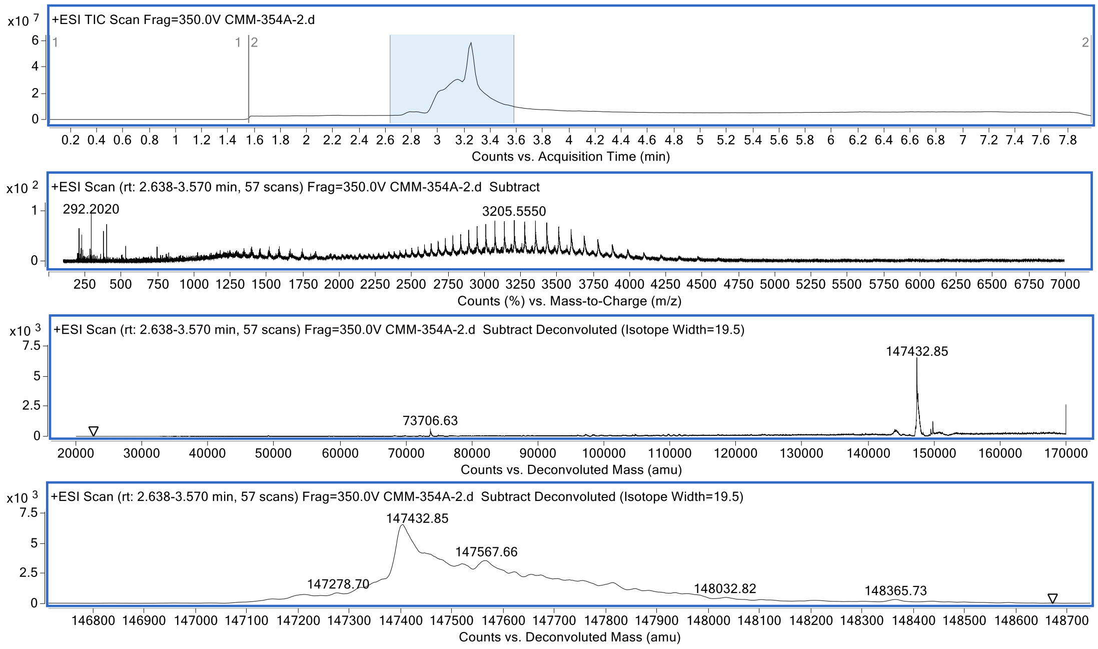

Figure S58: (i) TIC LC-MS trace (top), (ii) non-deconvoluted LC-MS trace (upper middle), (iii) deconvoluted MS data (lower middle, wide range), (iv) deconvoluted MS data (bottom, zoom in range).

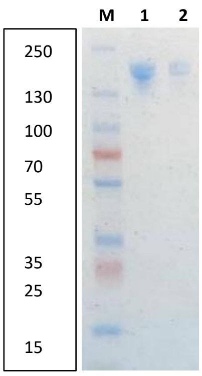

Figure S59: SDS-PAGE gel: M. Ladder, 1. Conjugate **17**, 2. Conjugate **18**.

HC S378C bisPD aniline fluorophore conjugate S19 (HC S378C thio-trastuzumab conjugated to ArN_3 bisPD 2, DBCO biotin and N_3 Aniline 3)

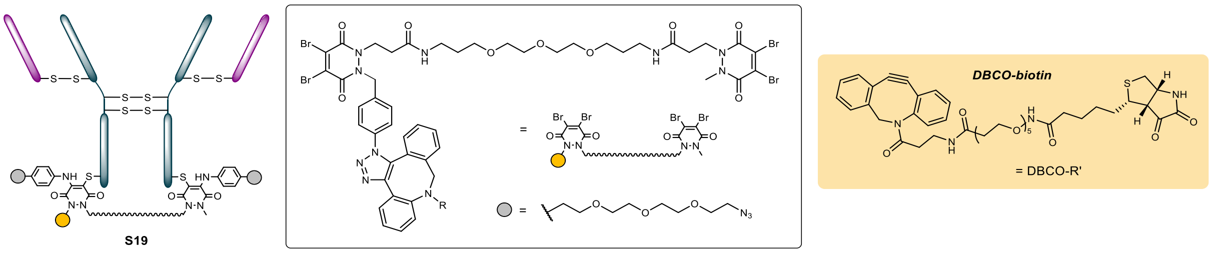

To a solution of conjugated HC S378C thio-trastuzumab **S18** (180 µL, 20 µM) in BBS (25 mM borate, 25 mM NaCl, 2 mM EDTA, 2% DMSO, pH 8.0) was added N_3 aniline **3** (7.2 µL, 0.5 M in DMSO, 1000 eq.) and the reaction incubated at 37 °C for 16 h under constant agitation (300 rpm). After this, excess reagents were removed to give conjugate **S19** (expected mass 147,207 Da, observed mass 147,212 Da) which was analysed by LC-MS (method 1b), UV-Vis spectroscopy and SDS-PAGE.

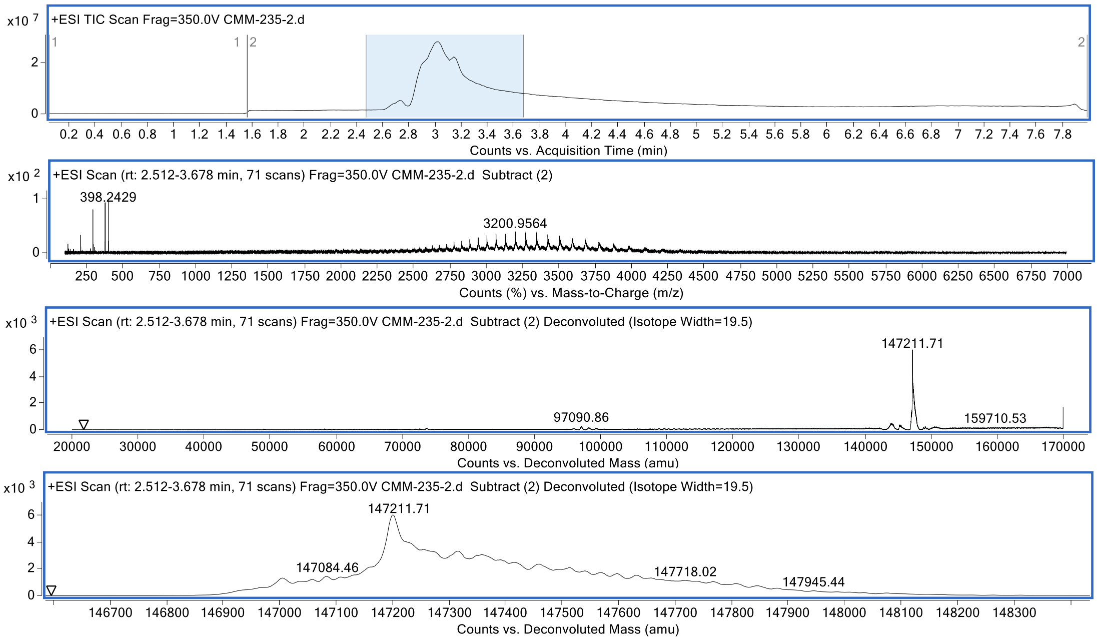

Figure S60: (i) TIC LC-MS trace (top), (ii) non-deconvoluted LC-MS trace (upper middle), (iii) deconvoluted MS data (lower middle, wide range), (iv) and (v) deconvoluted MS data (bottom, zoom in range).

HC S378C bisPD aniline fluorophore conjugate 19 (HC S378C thio-trastuzumab conjugated to ArN$_3$ bisPD 2, BP Fluor 568, N$_3$ Aniline and BP Fluor 647)

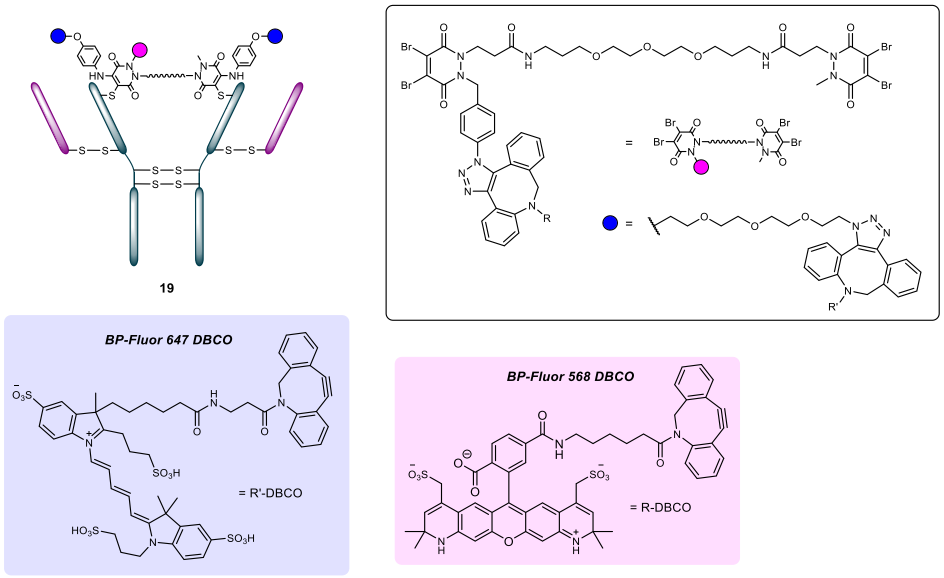

To a solution of conjugated HC S378C thio-trastuzumab **18** (175 µL, 18.8 µM) in BBS (25 mM borate, 25 mM NaCl, 2 mM EDTA, 2% DMSO, pH 8.0) was added BP Fluor 647 (9.9 µL, 20 mM in DMSO, 60 eq.) and the reaction incubated at 37 °C for 16 h under constant agitation (300 rpm). After this, excess reagents were removed to give conjugate **19** (expected mass 149,673 Da, observed mass 149,655 Da) which was analysed by LC-MS (method 1b), UV-Vis spectroscopy and SDS-PAGE.

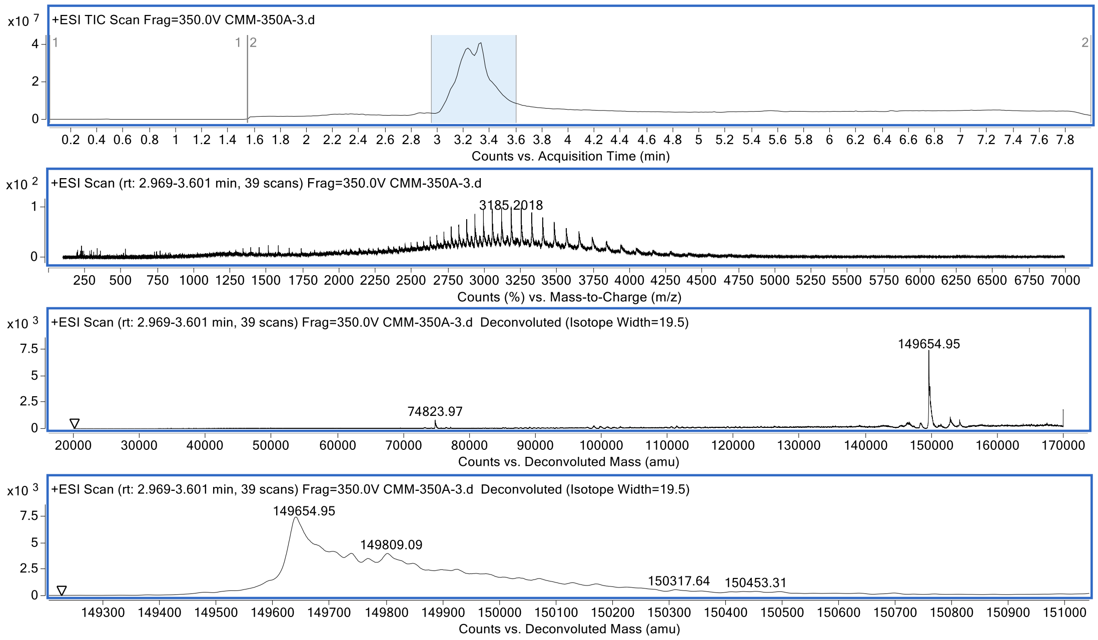

Figure S61: (i) TIC LC-MS trace (top), (ii) non-deconvoluted LC-MS trace (upper middle), (iii) deconvoluted MS data (lower middle, wide range), (iv) deconvoluted MS data (bottom, zoom in range)

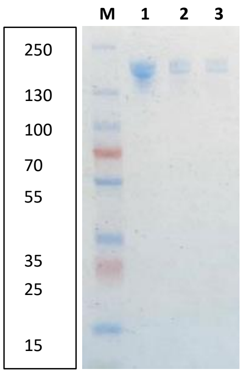

Figure S62: SDS-PAGE gel: M. Ladder, 1. Conjugate **17**, 2. Conjugate **18**, 3. Conjugate **19**.

HC S378C bisPD aniline fluorophore conjugate 20 (HC S378C thio-trastuzumab conjugated to ArN₃ bisPD 2, DBCO biotin, N₃ Aniline 3 and BP Fluor 568)

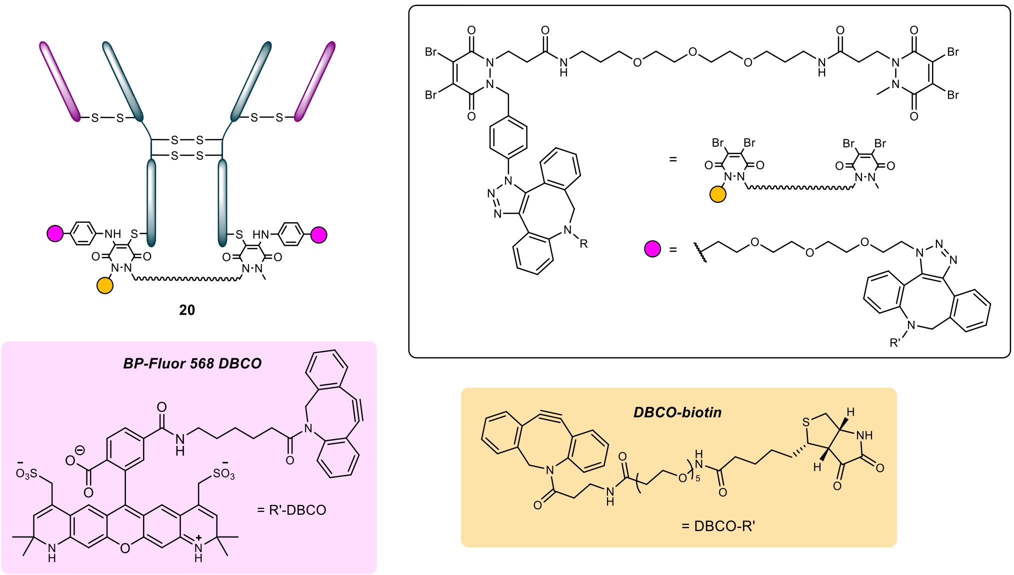

To a solution of conjugated HC S378C thio-trastuzumab **S19** (100 µL, 18.7 µM) in BBS (25 mM borate, 25 mM NaCl, 2 mM EDTA, 2% DMSO, pH 8.0) was added BP Fluor 568 (1.9 µL, 20 mM in DMSO, 20 eq.) and the reaction incubated at 37 °C for 16 h under constant agitation (300 rpm). After this, excess reagents were removed to give conjugate **20** (expected mass 149,114 Da, observed mass 149,121 Da) which was analysed by LC-MS (method 1b), UV-Vis spectroscopy and SDS-PAGE.

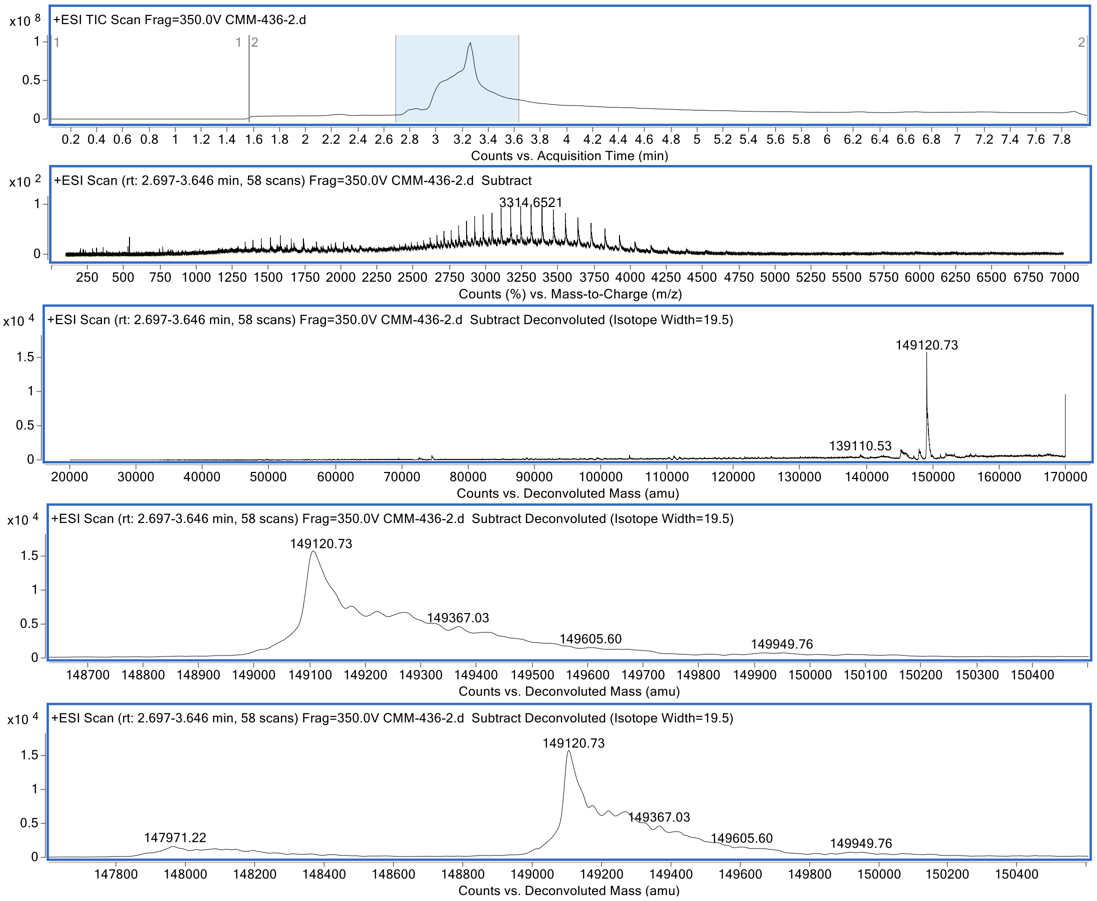

Figure S63: (i) TIC LC-MS trace (top), (ii) non-deconvoluted LC-MS trace (upper middle), (iii) deconvoluted MS data (lower middle, wide range), (iv) and (v) deconvoluted MS data (bottom, zoom in range).

LC S168C bisPD conjugates S20 and S21 (Reaction of HC S378C thio-trastuzumab with diMe bisPD 5)

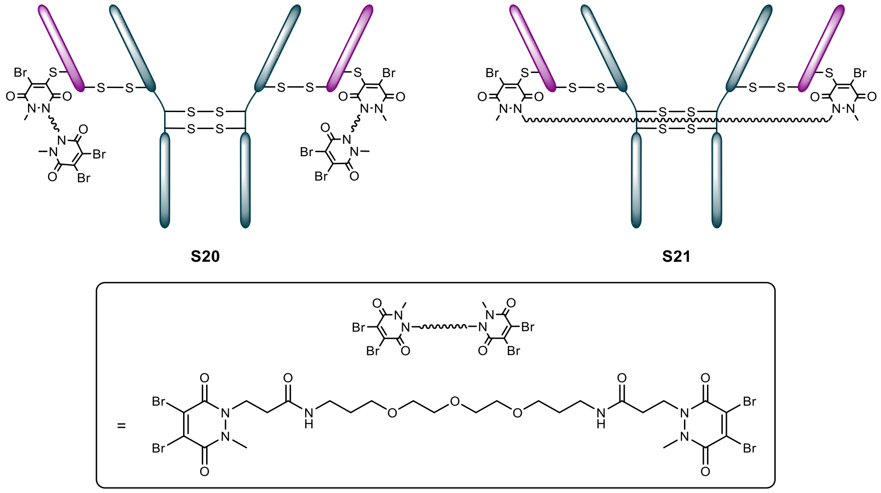

To a solution of HC S378C thio-trastuzumab **7** (25 µL, 20 µM) in BBS (25 mM borate, 25 mM NaCl, 2 mM EDTA, 2% DMSO, pH 8.0) was added diMe bisPD **5** (0.24 µL, 8.2 mM in MeCN, 4 eq.) and the reaction incubated at 37 °C for 3 h under constant agitation (300 rpm). After this, excess reagents were removed to give conjugates **S20** and **S21** and the sample analysed by LC-MS (method 1a), UV-Vis spectroscopy and SDS-PAGE.

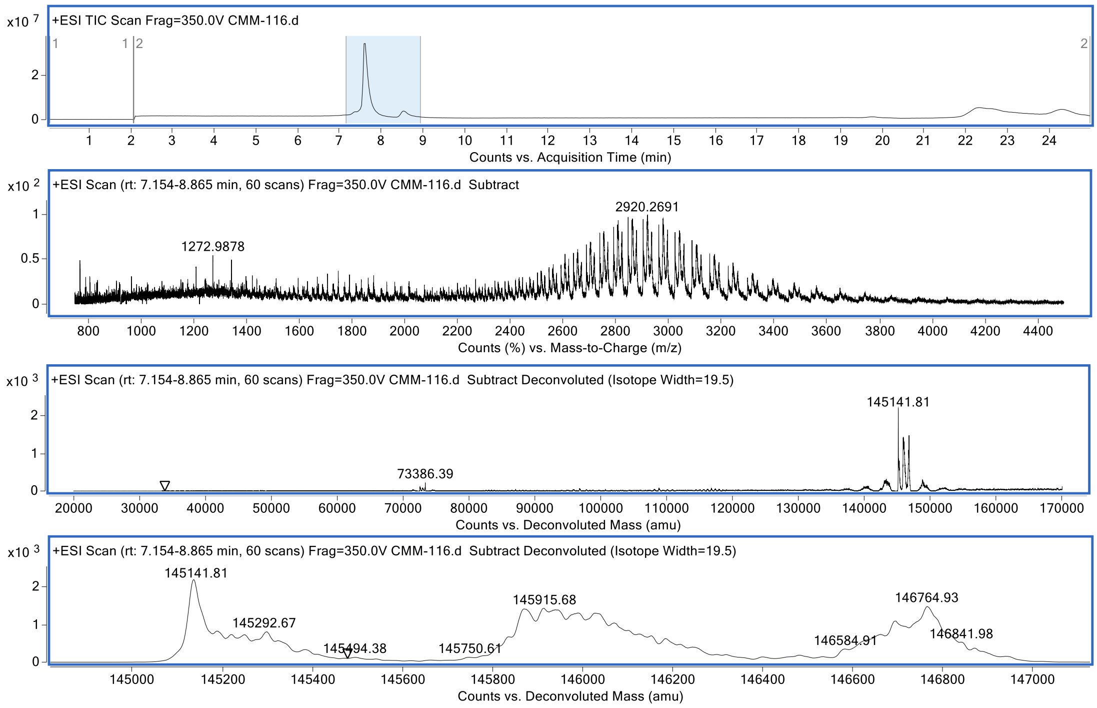

Figure S64: (i) TIC LC-MS trace (top), (ii) non-deconvoluted LC-MS trace (upper middle), (iii) deconvoluted MS data (lower middle, wide range), (iv) deconvoluted MS data (bottom, zoom in range).

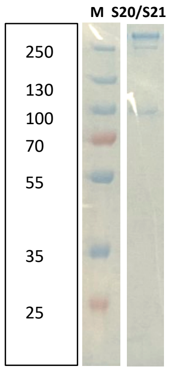

Figure S65: SDS-PAGE gel: M. Ladder, 1. Conjugates **S20** and **S21**.

Trastuzumab PD conjugate S22 (Trastuzumab re-bridged with BCN PD 1)

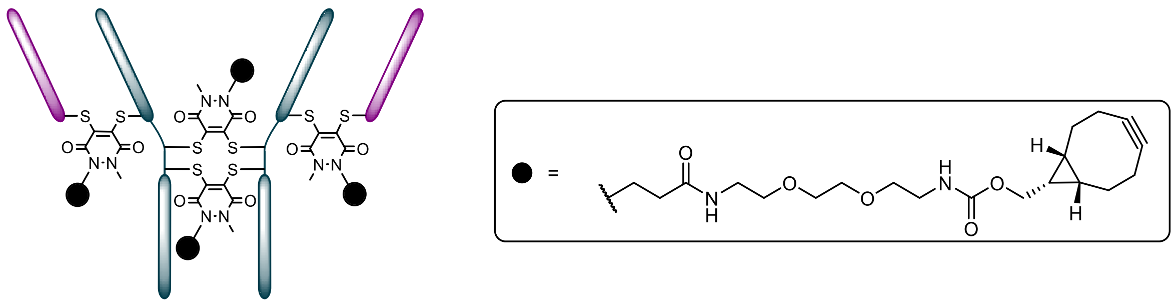

S22

To a solution of trastuzumab (Ontruzant®, A µM, 100 µL) in BBS (25 mM borate, 25 mM NaCl, pH 8.0, 2 mM EDTA, pH 8.0, 2% DMSO (unless otherwise specified below), was added TCEP.HCl (20 mM in dH_2O, B eq.). The mixture was incubated for C h at D °C under constant agitation (300 rpm). After this time, excess TCEP was removed *via* ultrafiltration into BBS and BCN PD **1** (20 mM in DMSO, E eq.) was added. The reaction was incubated at F °C for D h under constant agitation (300 rpm). After this time, excess reagents were removed *via* ultrafiltration into BBS and the concentration of the sample and PDAR were assessed by UV-Vis spectroscopy and LC-MS (method 1a).

PDAR 4: Expected mass = 147,189 Da; PDAR 3: Expected mass = 146686 Da; PDAR 2: Expected mass = 146,183 Da; Native Ontruzant®: Expected mass = 145,176 Da.

	Antibody Concentration (μM) (A)	TCEP eq. (B)	Reduction Time (C)	Reduction temperature (°C) (D)	PD eq. (E)	Reaction Time and Temperature (°C) (F, G)	pH
**S22a**	40	10	1.5 h	4	20	16 h, 4	8.0
**S22b**	40	10	1.5 h	4	40	16 h, 4	8.0
**S22c**	40	10	1.5 h	4	200	16 h, 4	8.0
**S22d**	40	10	1.5 h	4	20	16 h, 4	7.4
**S22e**	40	20	1.5 h	4	20	16 h, 4	8.0
**S22f**	40	80	1.5 h	4	20	16 h, 4	8.0
**S22g**	40	10	1.5 h	22	20	16 h, 4	8.0
**S22h**	40	10	1.5 h	37	20	16 h, 4	8.0
**S22i**	40	10	8 h	4	20	16 h, 4	8.0
**S22j**	40	10	4 h	4	20	16 h, 4	8.0
**S22k**	40	10	1.5 h	37	15	16 h, 4	8.0
**S22l**	40	10	1.5 h	37	10	16 h, 4	8.0
**S22m**	40	10	1.5 h	37	8	16 h, 4	8.0
**S22n**	40	10	1.5 h	37	5	16 h, 4	8.0
**S22o**	10	10	1.5 h	37	8	16 h, 4	8.0
**S22p**	20	10	1.5 h	37	8	16 h, 4	8.0
**S22q**	60	10	1.5 h	37	8	16 h, 4	8.0
**S22r**	20	10	1.5 h	37	10	3 h, 37	8.0
**S22s**	20	10	1.5 h	37	10	16 h, 4	8.0
**S22t**	20	10	1 h	37	10	3 h, 37	8.0

Table S10. Stepwise trials at forming conjugate **S22**.
