# Supplementary material for: Enabling the synthesis of multi-payload thio-antibody conjugates through the use of pyridazinediones, p-anisidine derivatives and various click chemistries
Source: RSC Chem Biol. 2026 Feb 3;7(4):656–68. doi: 10.1039/d6cb00018e (PMC12917607; doi:10.1039/d6cb00018e)
Supplement: CB-007-D6CB00018E-s002 [file CB-007-D6CB00018E-s002.pdf]

Supplementary Information (SI) for RSC Chemical Biology.
This journal is © The Royal Society of Chemistry 2026

S22a

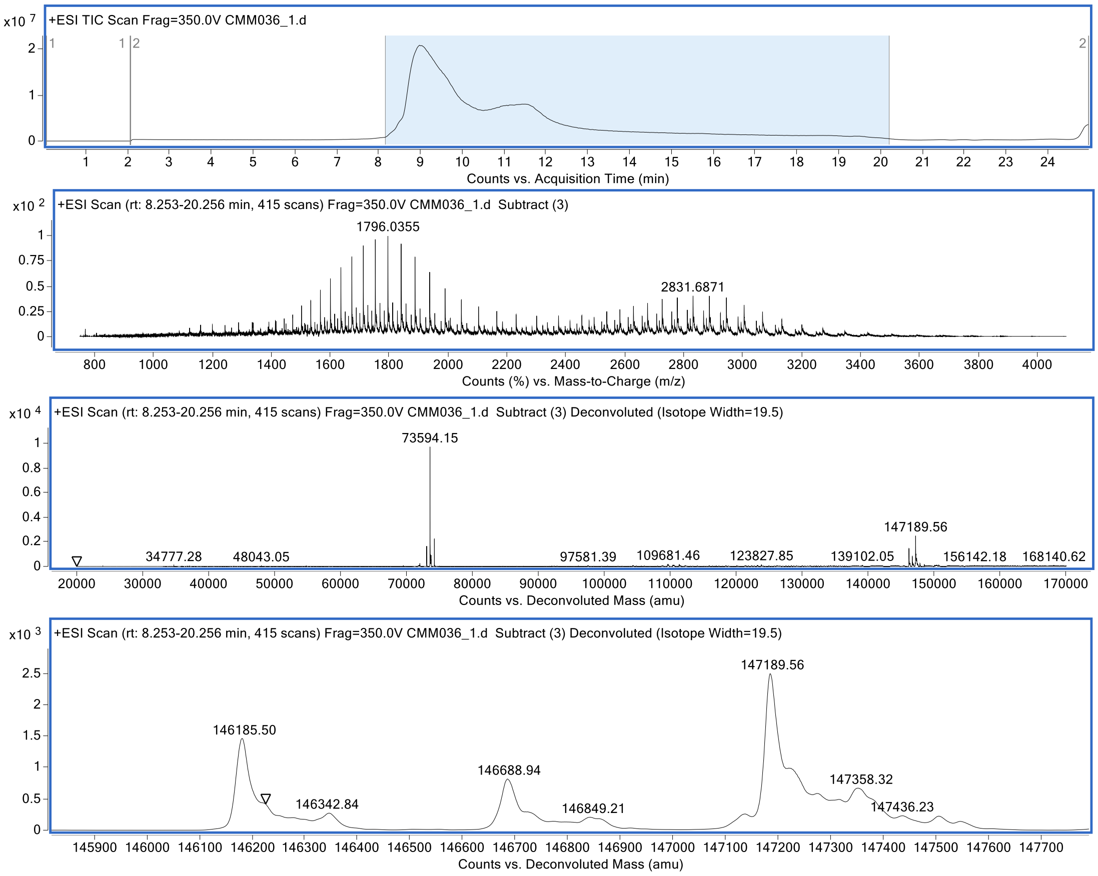

Figure S66: (i) TIC LC-MS trace (top), (ii) non-deconvoluted LC-MS trace (upper middle), (iii) deconvoluted MS data (lower middle, wide range), (iv) deconvoluted MS data (bottom, zoom in range).

S22b

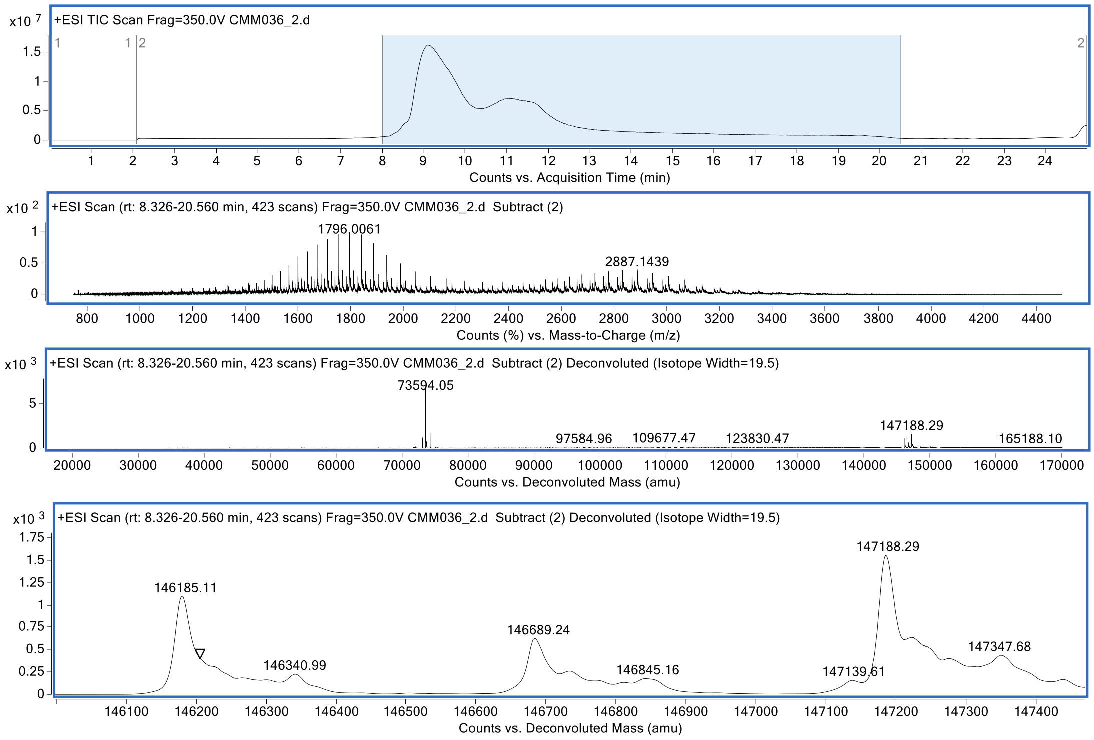

Figure S67: (i) TIC LC-MS trace (top), (ii) non-deconvoluted LC-MS trace (upper middle), (iii) deconvoluted MS data (lower middle, wide range), (iv) deconvoluted MS data (bottom, zoom in range).

S22c

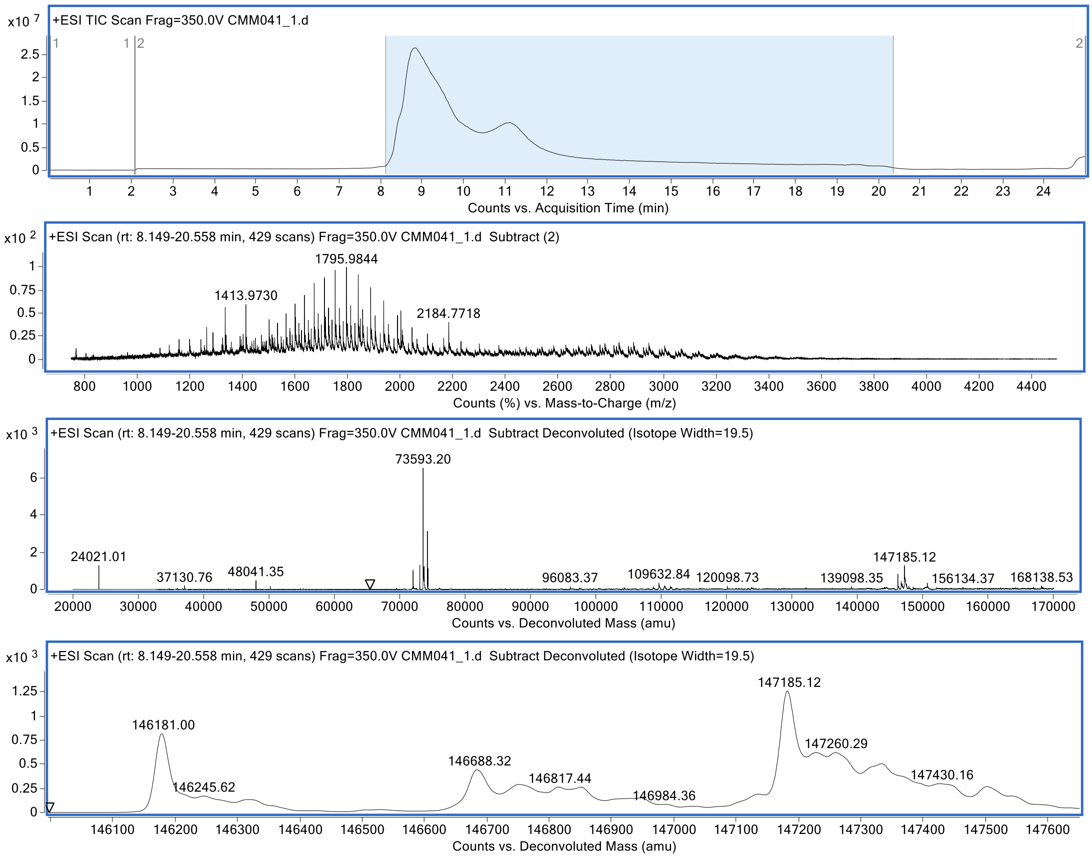

Figure S68: (i) TIC LC-MS trace (top), (ii) non-deconvoluted LC-MS trace (upper middle), (iii) deconvoluted MS data (lower middle, wide range), (iv) deconvoluted MS data (bottom, zoom in range).

S22d

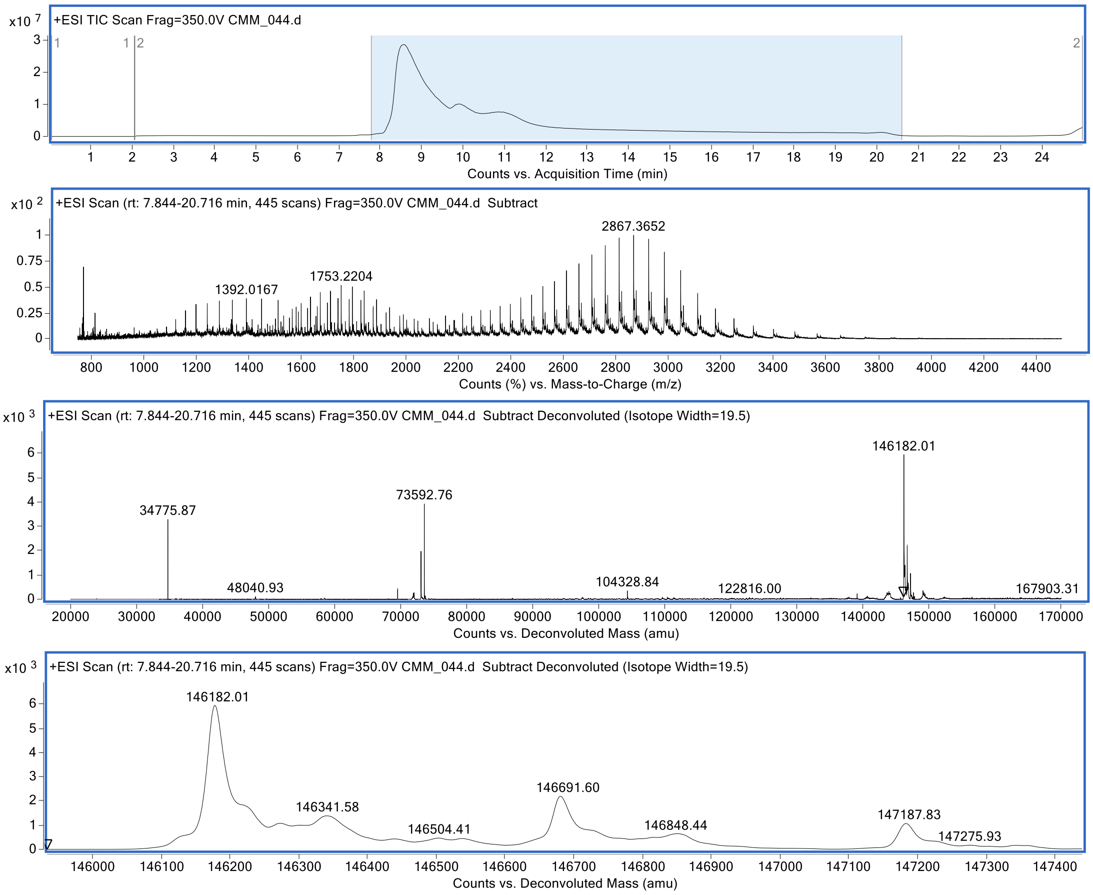

Figure S69: (i) TIC LC-MS trace (top), (ii) non-deconvoluted LC-MS trace (upper middle), (iii) deconvoluted MS data (lower middle, wide range), (iv) deconvoluted MS data (bottom, zoom in range).

S22e

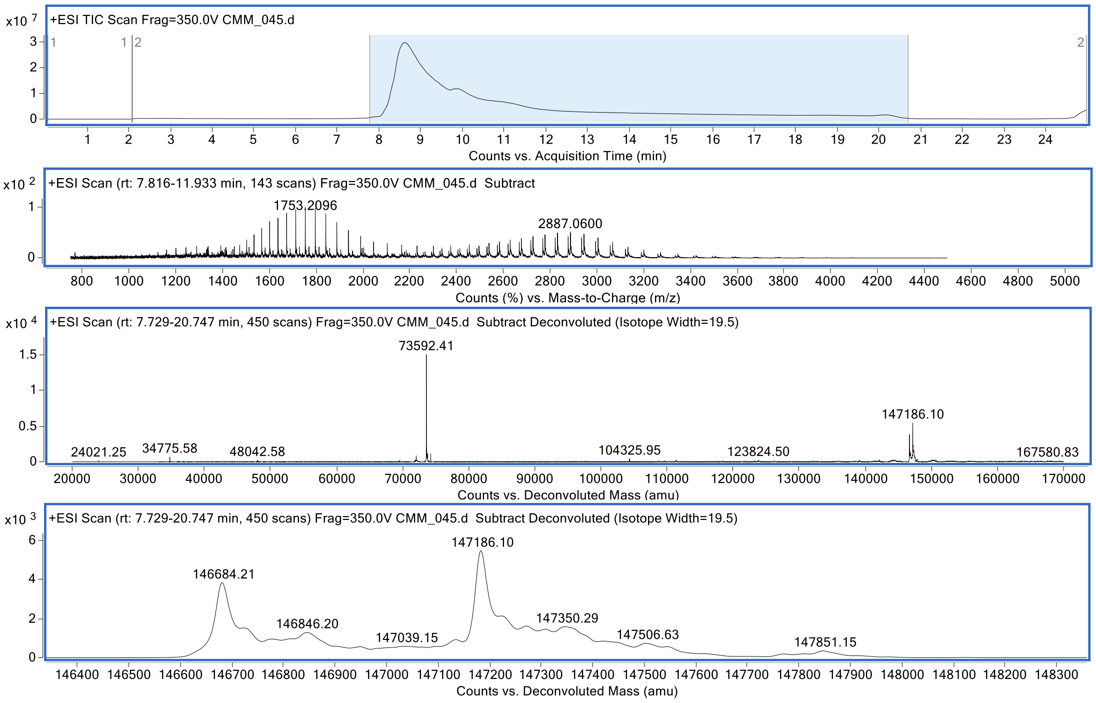

Figure S70: (i) TIC LC-MS trace (top), (ii) non-deconvoluted LC-MS trace (upper middle), (iii) deconvoluted MS data (lower middle, wide range), (iv) deconvoluted MS data (bottom, zoom in range).

S22f

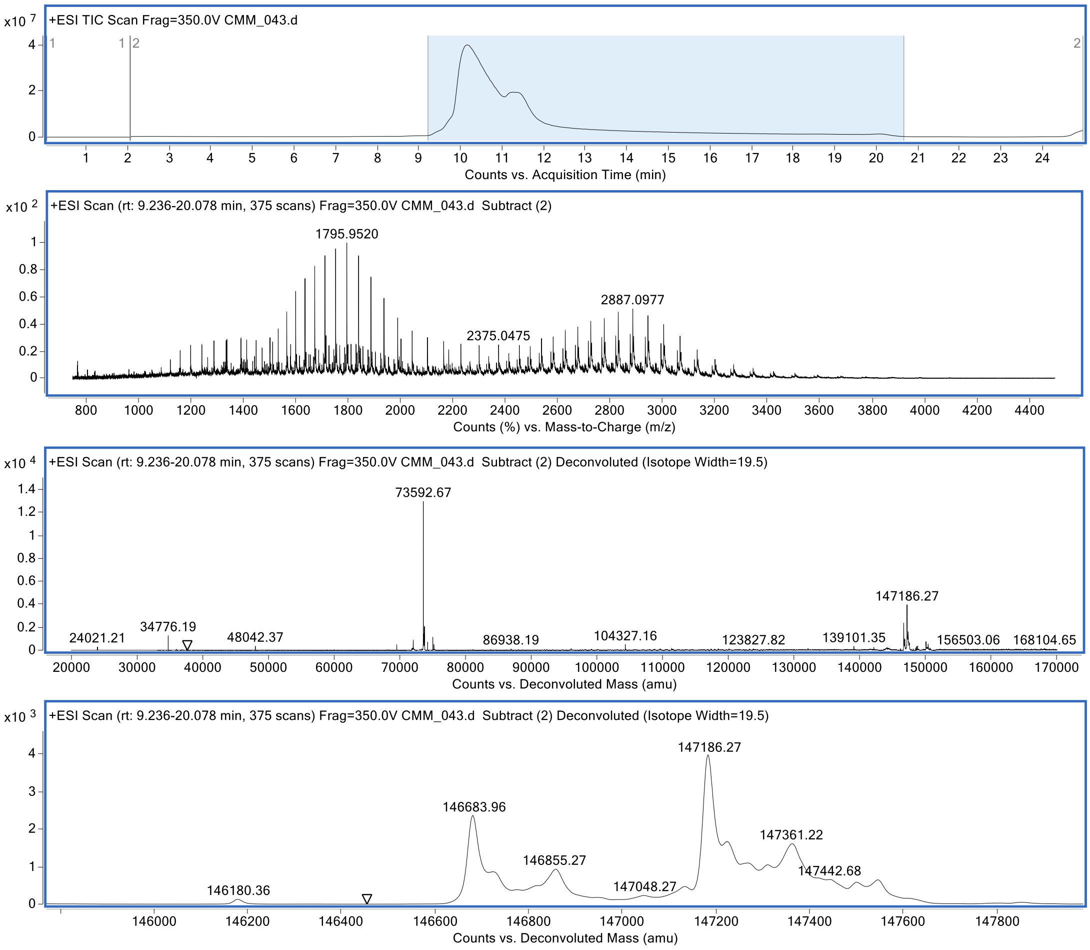

Figure S71: (i) TIC LC-MS trace (top), (ii) non-deconvoluted LC-MS trace (upper middle), (iii) deconvoluted MS data (lower middle, wide range), (iv) deconvoluted MS data (bottom, zoom in range).

S22g

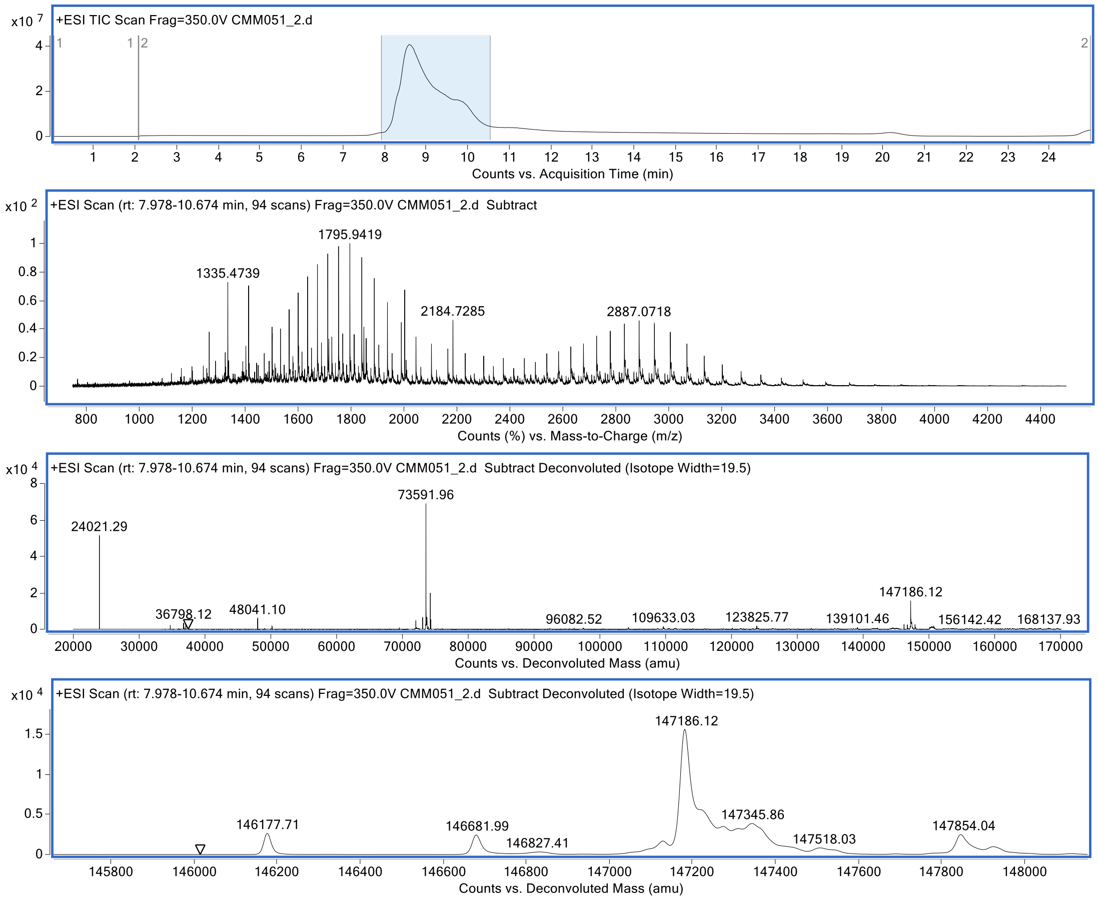

Figure S72: (i) TIC LC-MS trace (top), (ii) non-deconvoluted LC-MS trace (upper middle), (iii) deconvoluted MS data (lower middle, wide range), (iv) deconvoluted MS data (bottom, zoom in range).

S22h

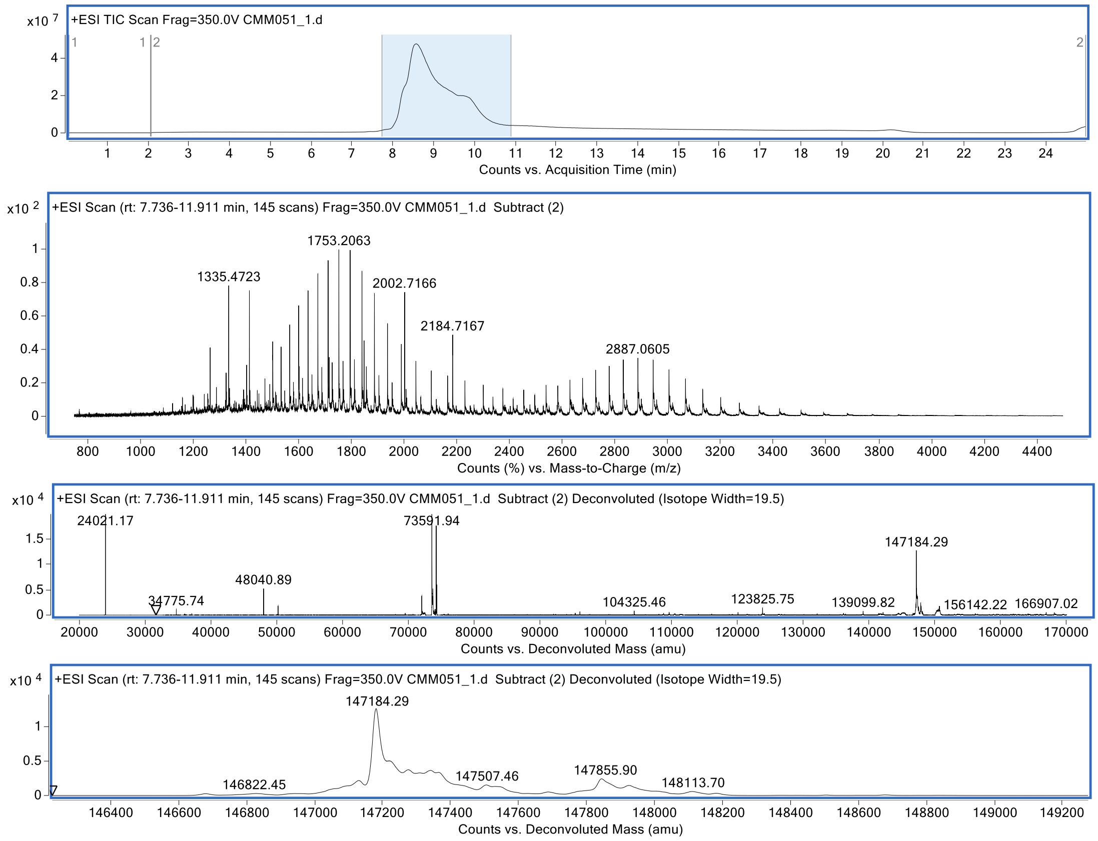

Figure S73: (i) TIC LC-MS trace (top), (ii) non-deconvoluted LC-MS trace (upper middle), (iii) deconvoluted MS data (lower middle, wide range), (iv) deconvoluted MS data (bottom, zoom in range).

S22i

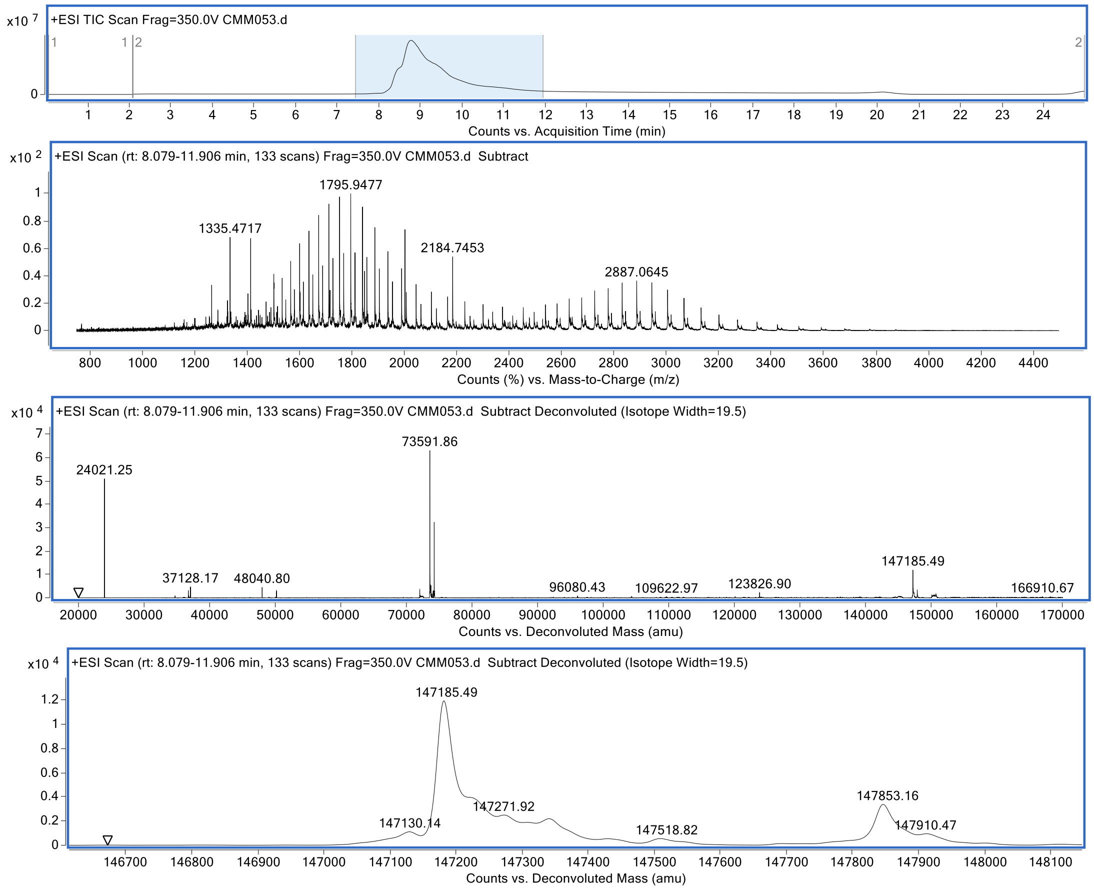

Figure S74: (i) TIC LC-MS trace (top), (ii) non-deconvoluted LC-MS trace (upper middle), (iii) deconvoluted MS data (lower middle, wide range), (iv) deconvoluted MS data (bottom, zoom in range).

S22j

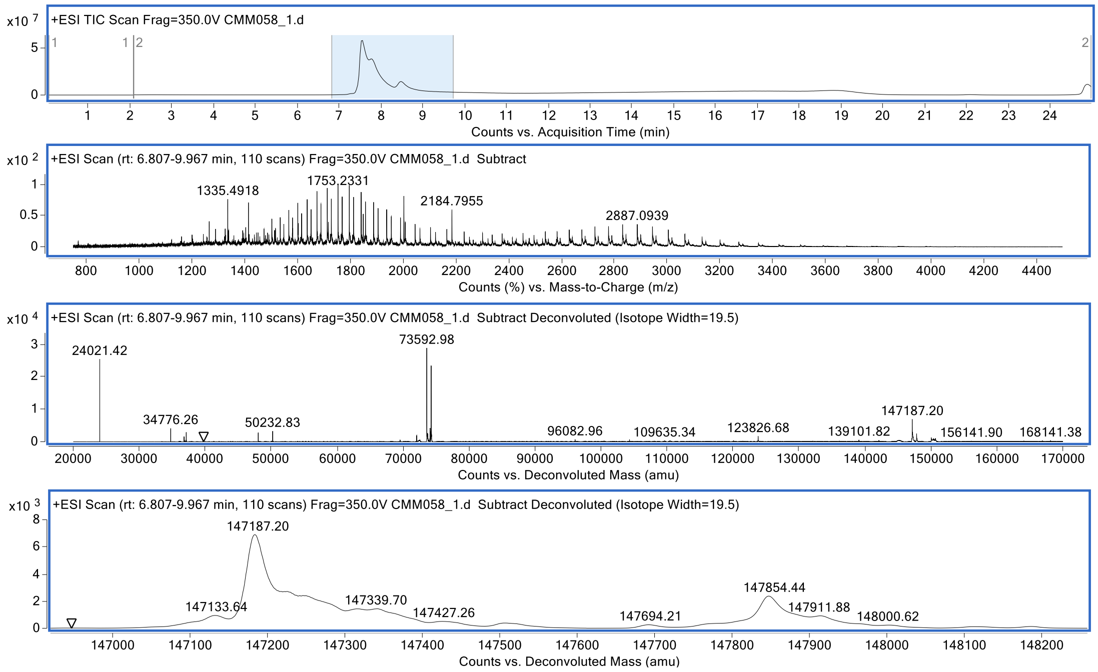

Figure S75: (i) TIC LC-MS trace (top), (ii) non-deconvoluted LC-MS trace (upper middle), (iii) deconvoluted MS data (lower middle, wide range), (iv) deconvoluted MS data (bottom, zoom in range).

S22k

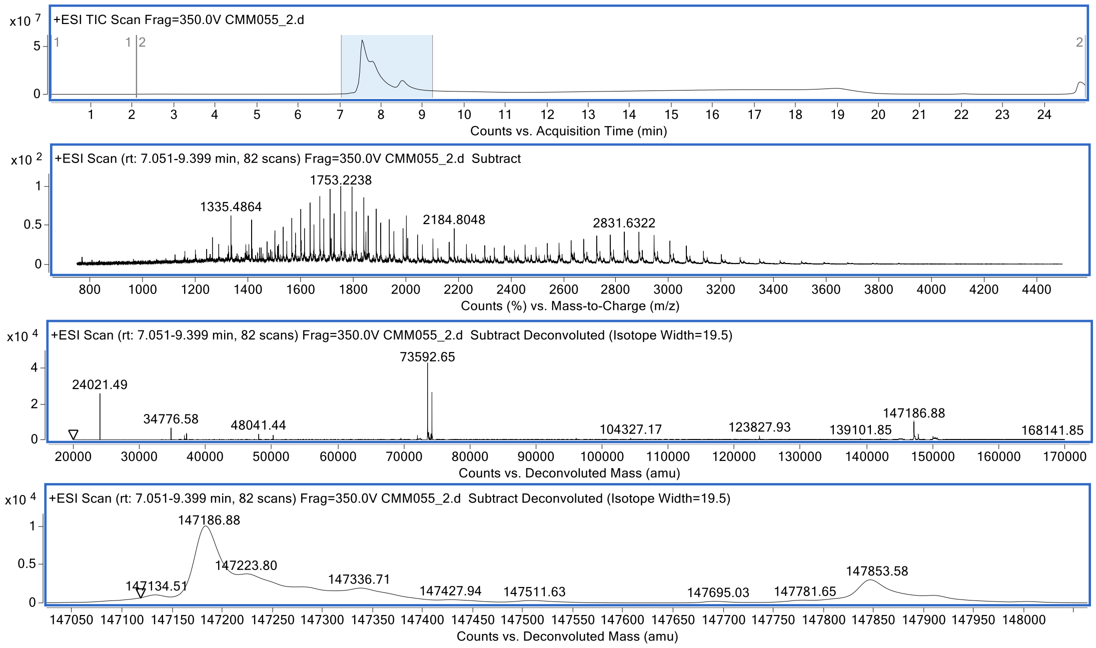

Figure S76: (i) TIC LC-MS trace (top), (ii) non-deconvoluted LC-MS trace (upper middle), (iii) deconvoluted MS data (lower middle, wide range), (iv) deconvoluted MS data (bottom, zoom in range).

S22l

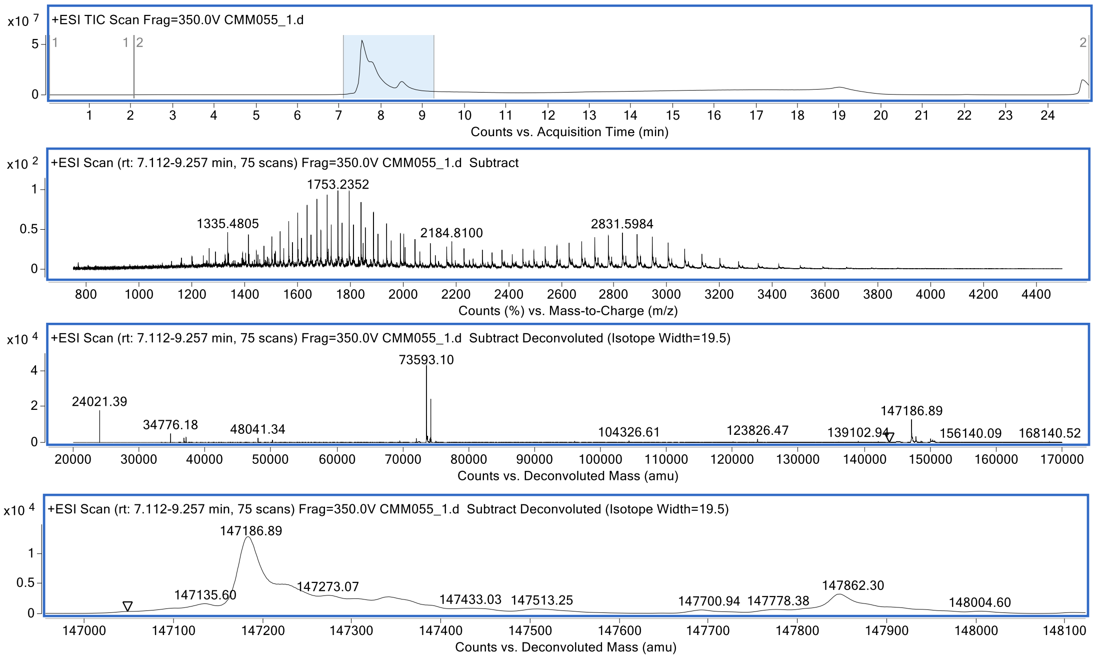

Figure S77: (i) TIC LC-MS trace (top), (ii) non-deconvoluted LC-MS trace (upper middle), (iii) deconvoluted MS data (lower middle, wide range), (iv) deconvoluted MS data (bottom, zoom in range).

S22m

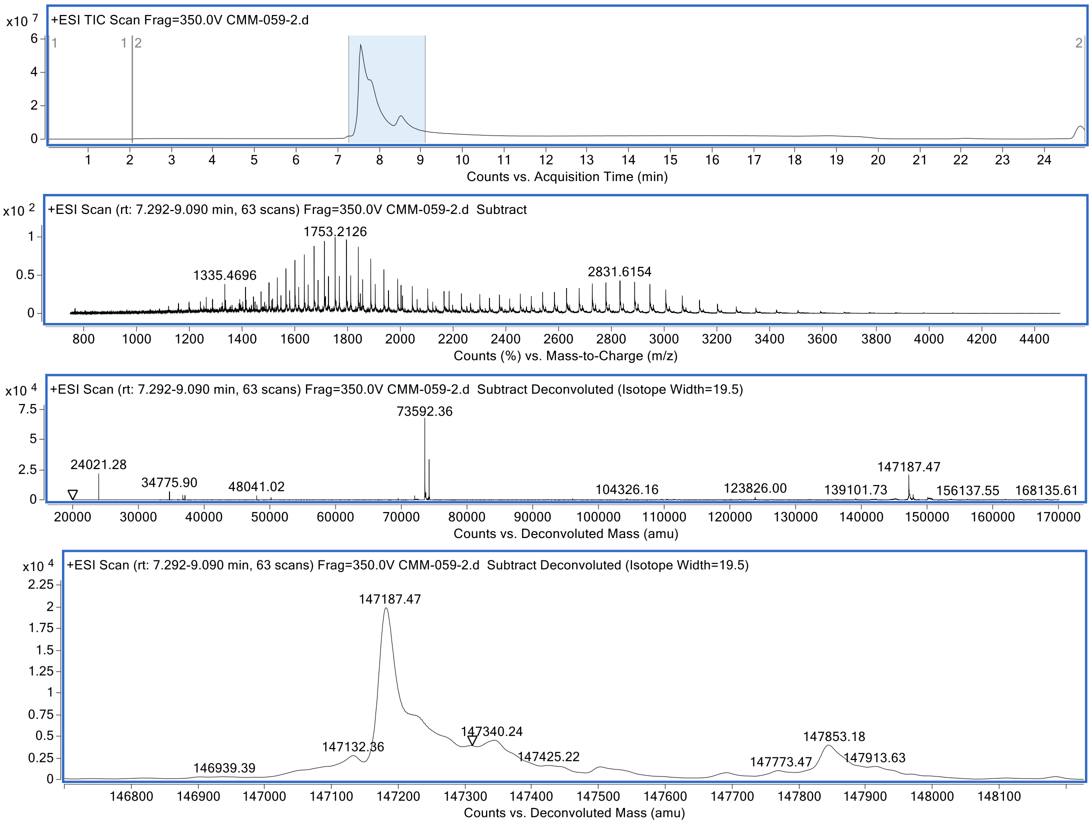

Figure S78: (i) TIC LC-MS trace (top), (ii) non-deconvoluted LC-MS trace (upper middle), (iii) deconvoluted MS data (lower middle, wide range), (iv) deconvoluted MS data (bottom, zoom in range).

S22n

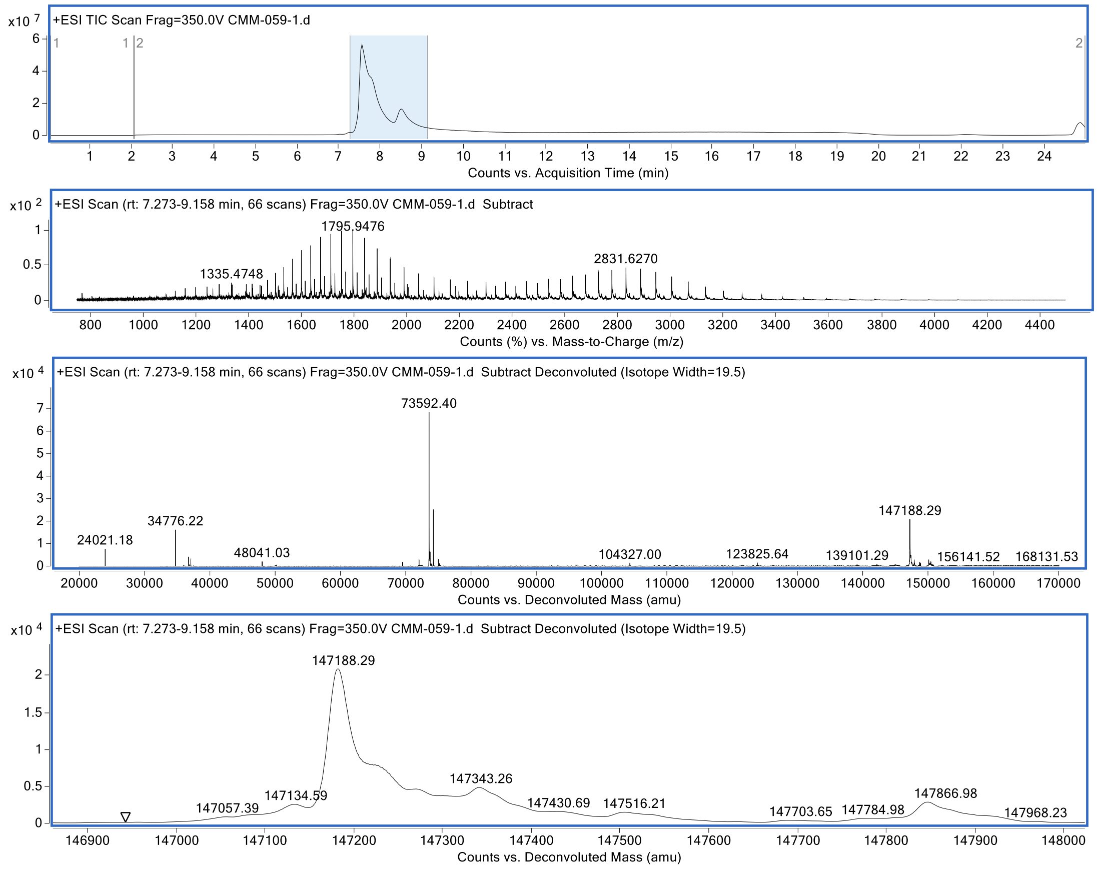

Figure S79: (i) TIC LC-MS trace (top), (ii) non-deconvoluted LC-MS trace (upper middle), (iii) deconvoluted MS data (lower middle, wide range), (iv) deconvoluted MS data (bottom, zoom in range).

S22o

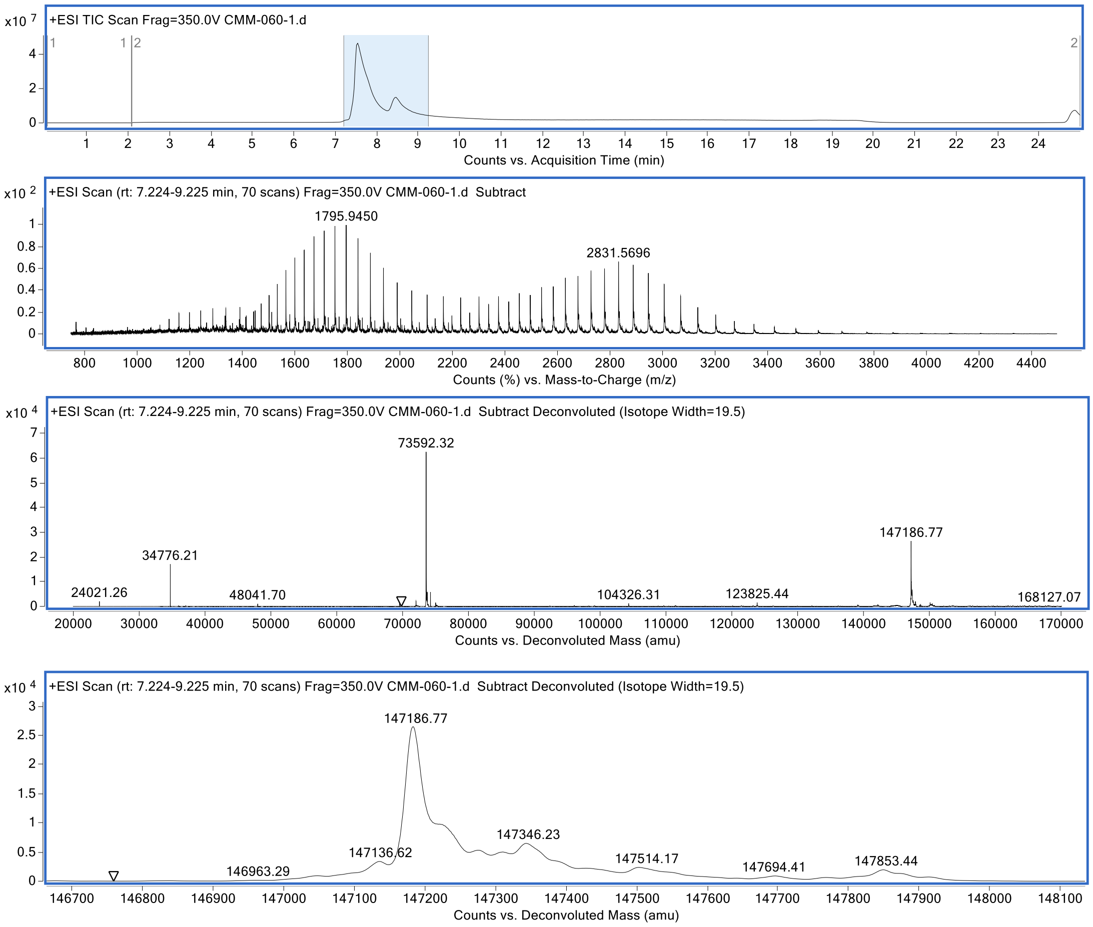

Figure S80: (i) TIC LC-MS trace (top), (ii) non-deconvoluted LC-MS trace (upper middle), (iii) deconvoluted MS data (lower middle, wide range), (iv) deconvoluted MS data (bottom, zoom in range).

S22p

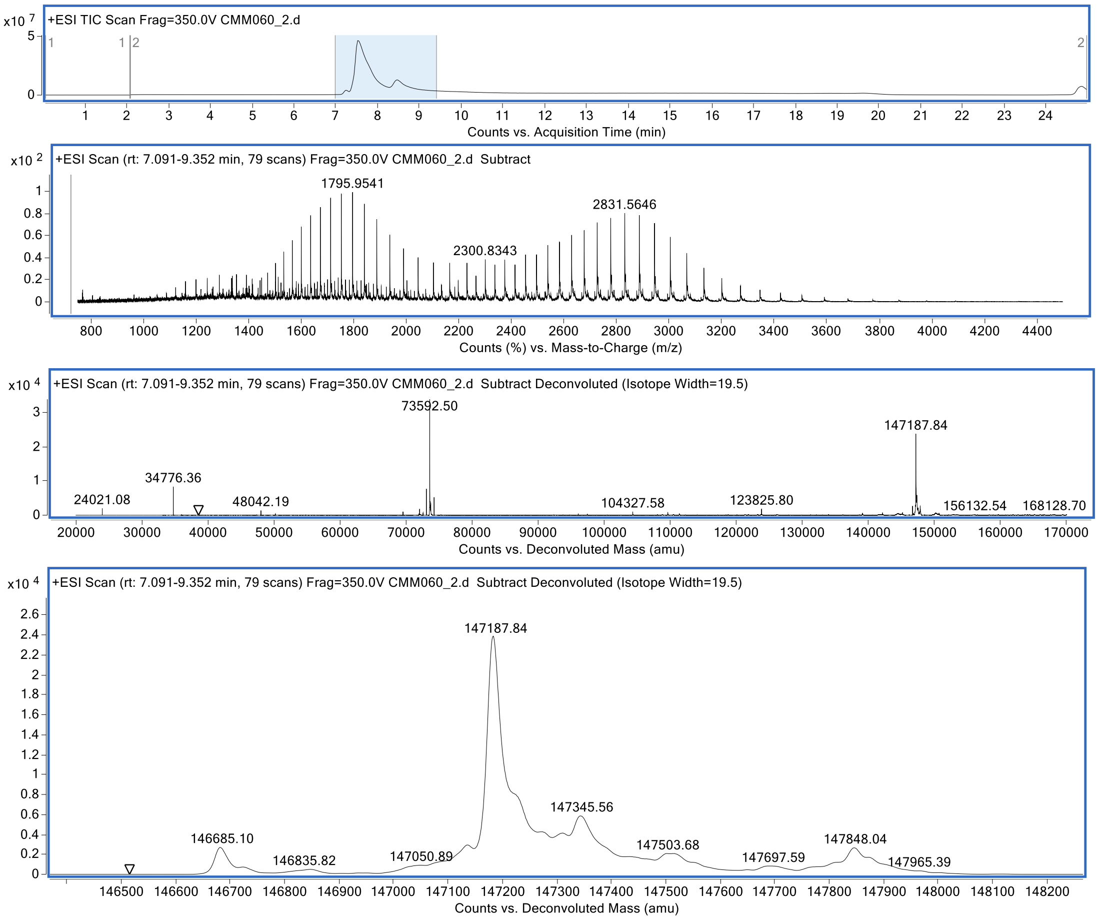

Figure S81: (i) TIC LC-MS trace (top), (ii) non-deconvoluted LC-MS trace (upper middle), (iii) deconvoluted MS data (lower middle, wide range), (iv) deconvoluted MS data (bottom, zoom in range).

S22q

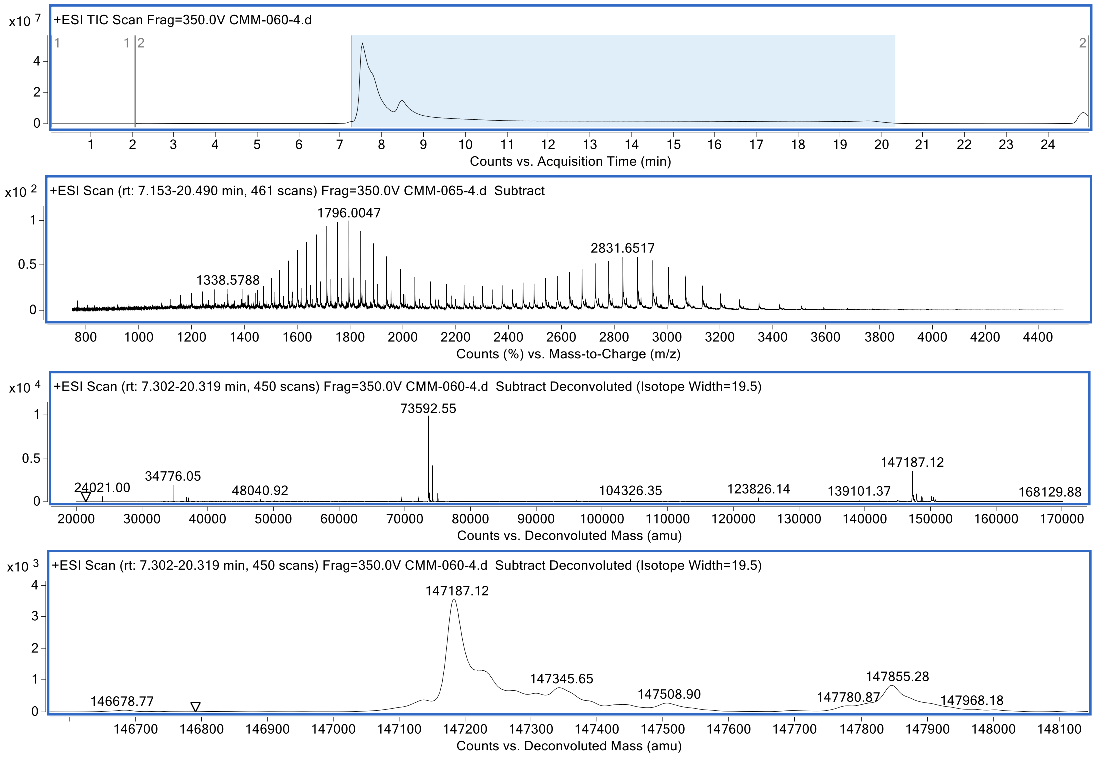

Figure S82: (i) TIC LC-MS trace (top), (ii) non-deconvoluted LC-MS trace (upper middle), (iii) deconvoluted MS data (lower middle, wide range), (iv) deconvoluted MS data (bottom, zoom in range).

S22r

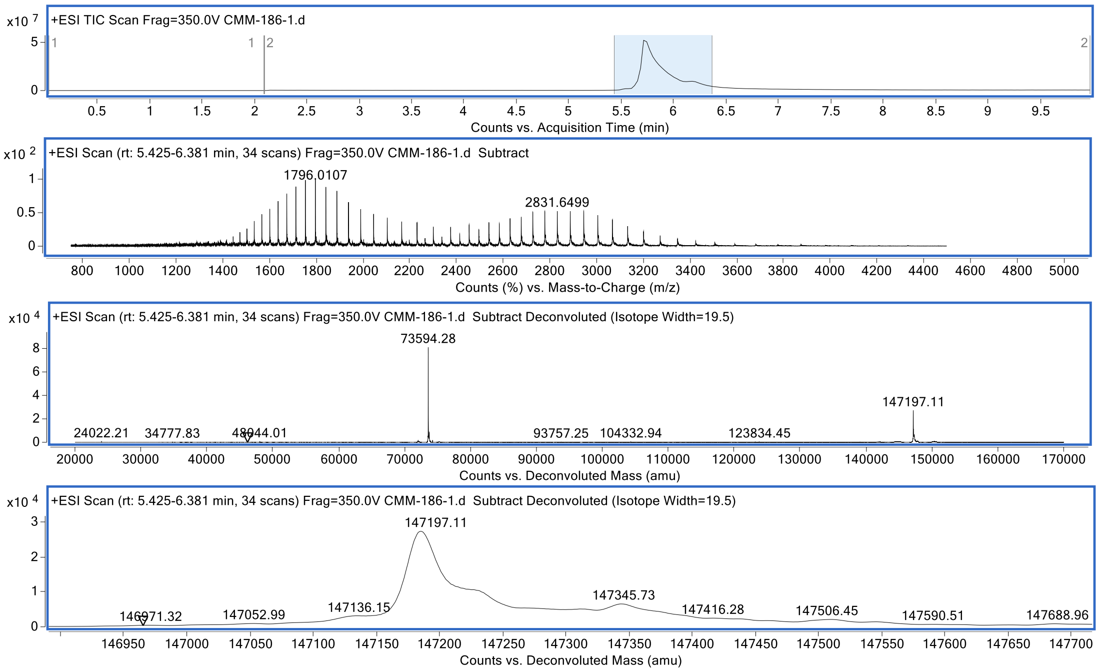

Figure S83: (i) TIC LC-MS trace (top), (ii) non-deconvoluted LC-MS trace (upper middle), (iii) deconvoluted MS data (lower middle, wide range), (iv) deconvoluted MS data (bottom, zoom in range).

S22s

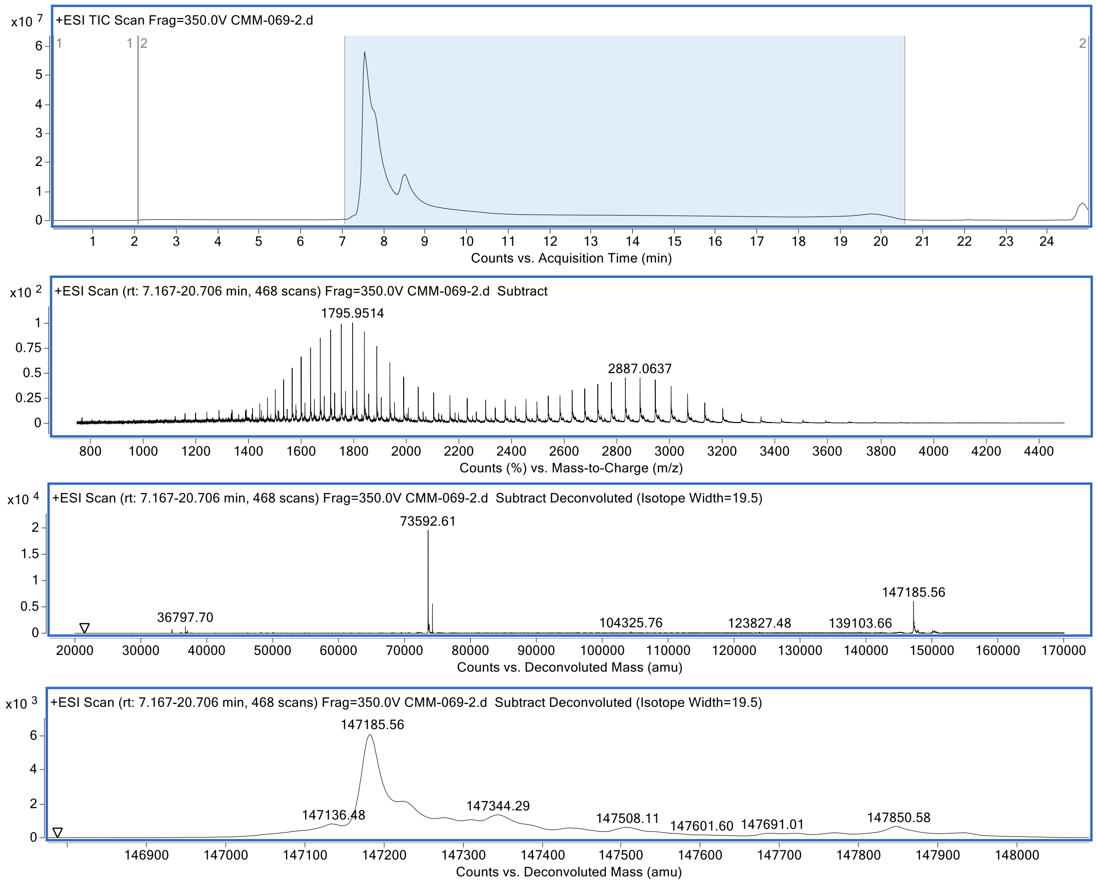

Figure S84: (i) TIC LC-MS trace (top), (ii) non-deconvoluted LC-MS trace (upper middle), (iii) deconvoluted MS data (lower middle, wide range), (iv) deconvoluted MS data (bottom, zoom in range).

S22t

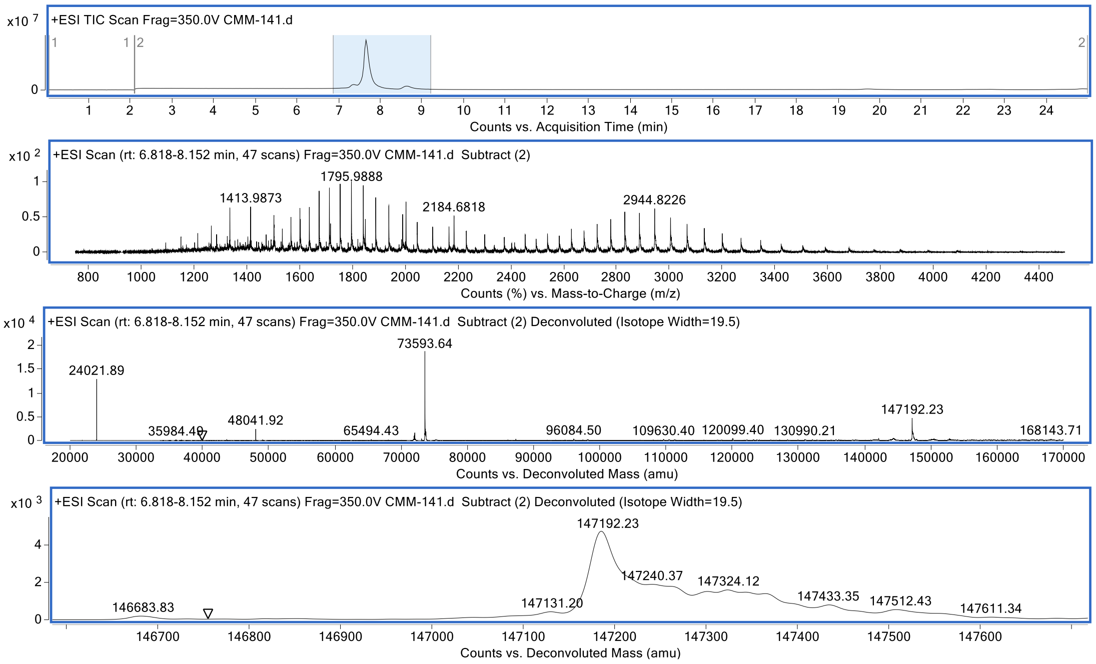

Figure S85: (i) TIC LC-MS trace (top), (ii) non-deconvoluted LC-MS trace (upper middle), (iii) deconvoluted MS data (lower middle, wide range), (iv) deconvoluted MS data (bottom, zoom in range).

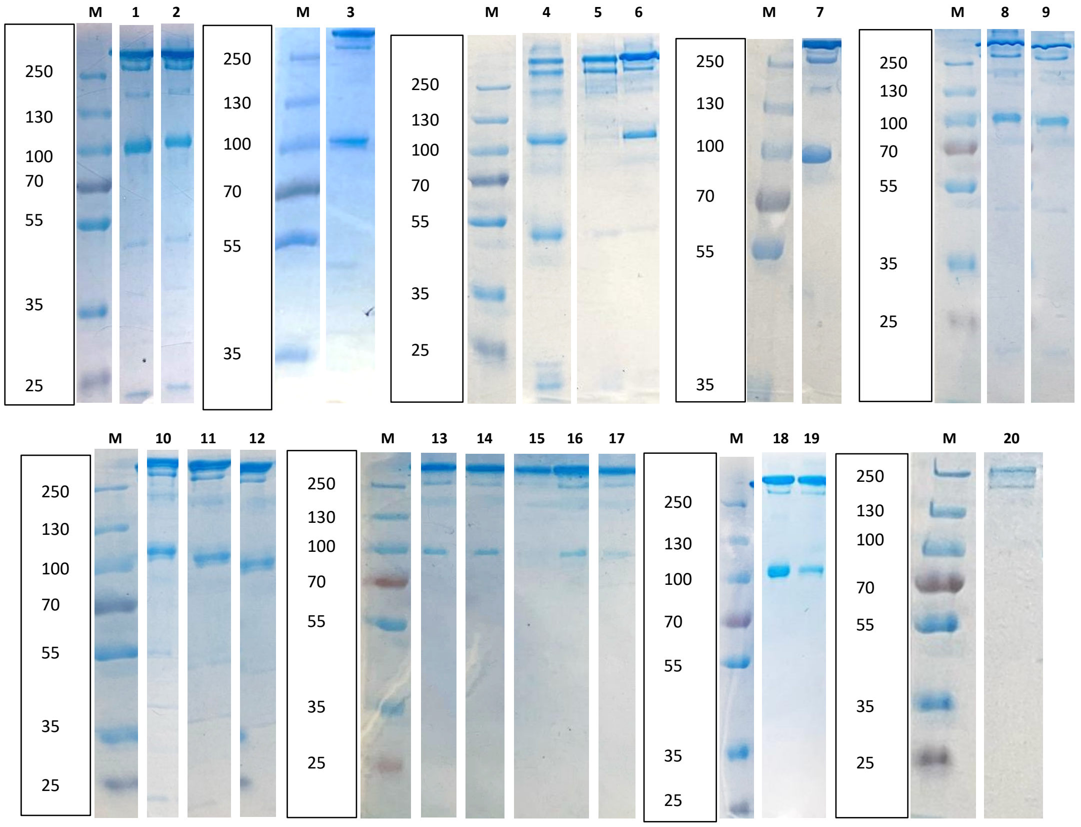

Figure S86: SDS-PAGE gel: M. Ladder, 1. Conjugate **S22a**, 2. Conjugate **S22b**, 3.Conjugate **S22c**, 4. Conjugate **S22d**, 5. Conjugate **S22f**, 6. Conjugate **S22i**, 7. Conjugate **S22e**, 8. Conjugate **S22g**, 9. Conjugate **S22h**, 10. Conjugate **S22j**, 11. Conjugate **S22k**, 12. Conjugate **S22l**, 13. Conjugate **S22m**, 14. Conjugate **S22n**, 15. Conjugate **S22o**, 16. Conjugate **S22p**, 17. Conjugate **S22q**, 18. Conjugate **S22r**, 19. Conjugate **S22s**, 20. Conjugate **S22t**.

Trastuzumab PD fluorophore conjugate S23 (trastuzumab re-bridged with BCN PD 1, clicked with Azide-Fluor 488)

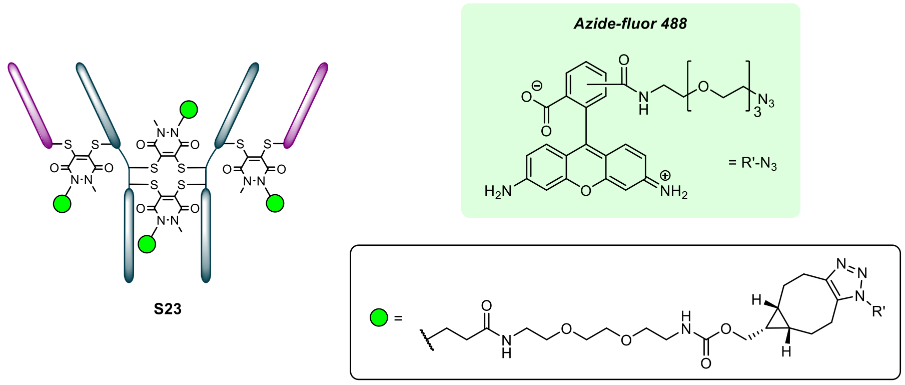

To a solution of re-bridged trastuzumab **S22** (100 µL, 20 µM) in BBS (25 mM borate, 25 mM NaCl, pH 8.0, 2 mM EDTA) was added Azide-fluor 488 (2 µL, 10 mM in DMSO, 10 eq.) and the mixture incubated at 37 °C with constant agitation (300 rpm) for 16 h to give the product **S23** (expected mass 149,487 Da, observed mass 149,483 Da). The concentration of the sample and FAR were assessed by UV-Vis spectroscopy and LC-MS (method 1a).

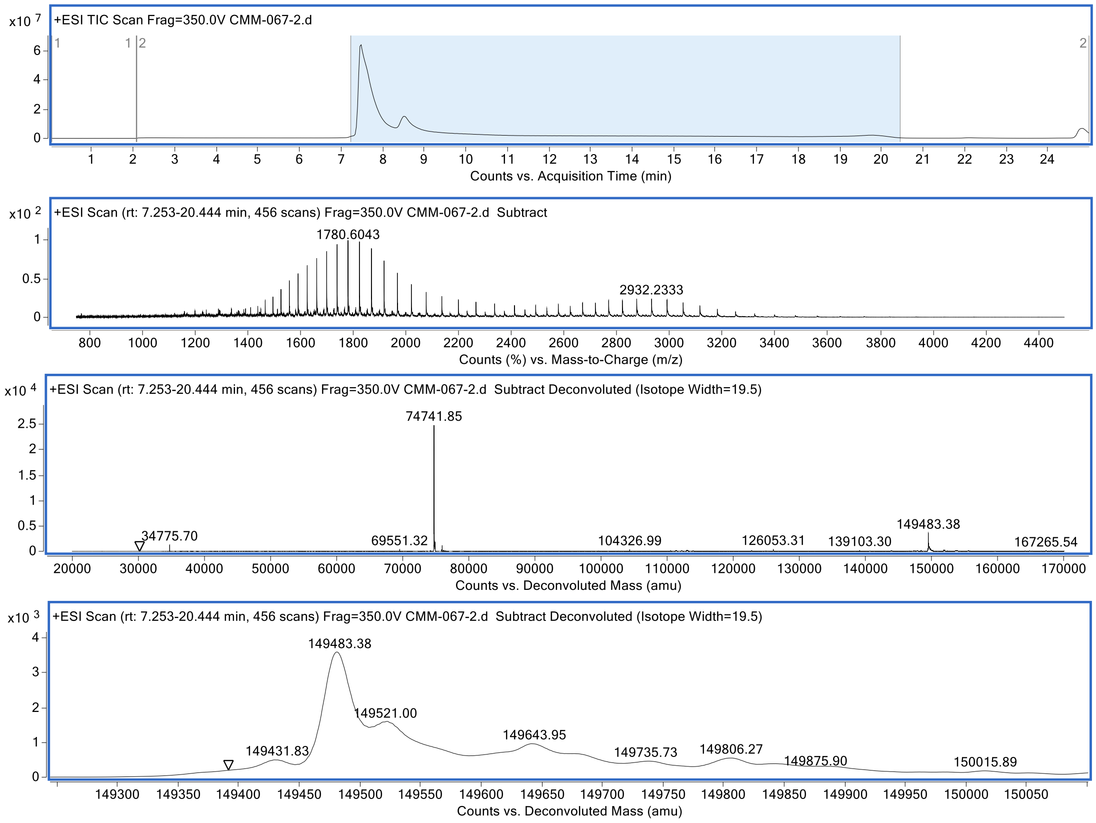

Figure S87: (i) TIC LC-MS trace (top), (ii) non-deconvoluted LC-MS trace (upper middle), (iii) deconvoluted MS data (lower middle, wide range), (iv) deconvoluted MS data (bottom, zoom in range).

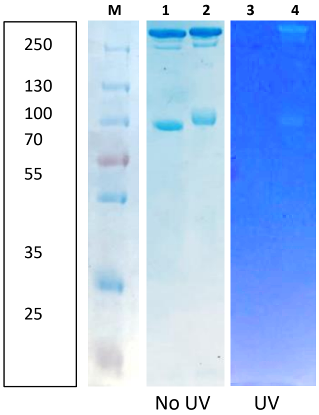

Figure S88: SDS-PAGE gel: M. Ladder, 1. Conjugate **S22**, no UV, 2. Conjugate **S23** , no UV,3. Conjugate **S22**, UV, 4. Conjugate **S23**, UV.

Trastuzumab bisPD conjugate S24 (Trastuzumab re-bridged with ArN_3 bisPD 2)

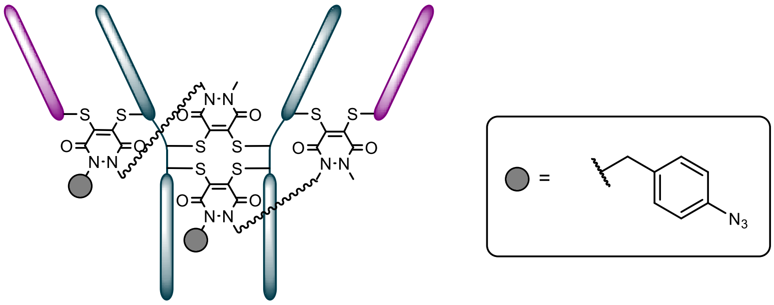

S24

To a solution of trastuzumab (100 µL, 20 µM) in BBS (25 mM borate, 25 mM NaCl, pH 8.0, 2 mM EDTA, 2% DMSO, pH 8.0) was added TCEP.HCl (1 µL, 20 mM in dH_2O, 10 eq.) and the mixture incubated for 1.5 h at 37 °C under constant agitation (300 rpm). After this time, the TCEP was removed and ArN_3 bisPD **2** (1 µL, 20 mM in DMSO, 10 eq.) added and the reaction incubated for 3 h at 37 °C under constant agitation (300 rpm) to give the product, **S24** (expected mass: 146,563 Da, observed mass: 146,572 Da). The concentration of the sample and PDAR were assessed by UV-Vis spectroscopy and LC-MS (method 1b).

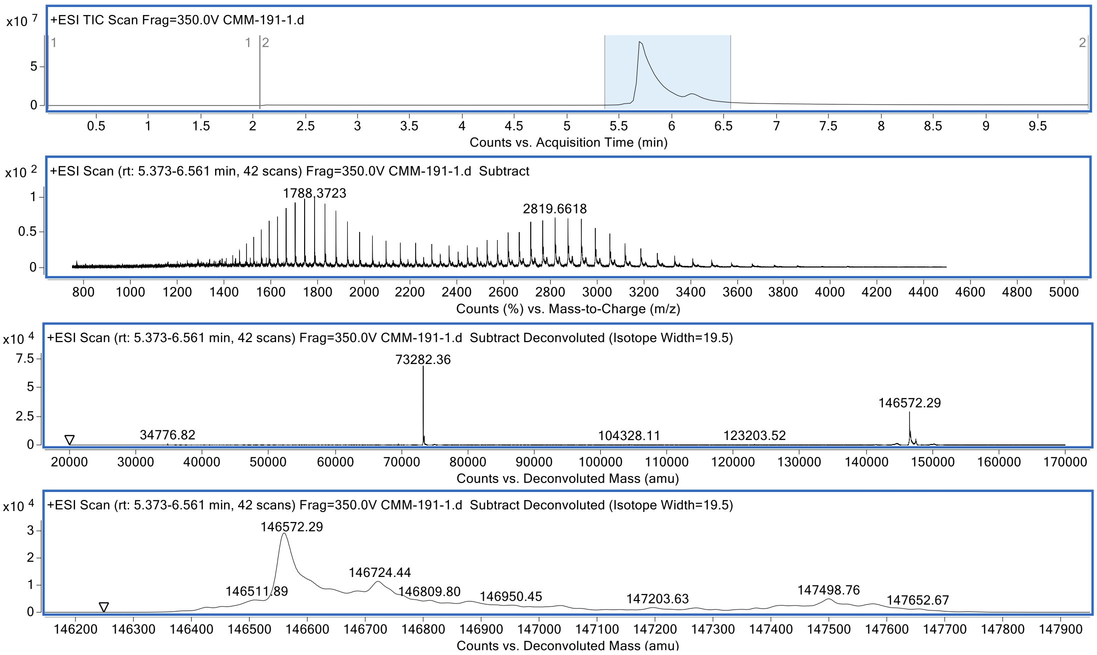

Figure S89: (i) TIC LC-MS trace (top), (ii) non-deconvoluted LC-MS trace (upper middle), (iii) deconvoluted MS data (lower middle, wide range), (iv) deconvoluted MS data (bottom, zoom in range).

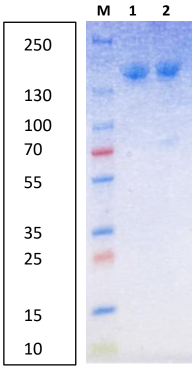

Figure S90: SDS-PAGE gel: M. Ladder, 1. Native trastuzumab, 2. Conjugate **S24**.

Trastuzumab bisPD conjugate S25 (trastuzumab re-bridged with ArN$_3$ bisPD 2 clicked with DBCO Biotin)

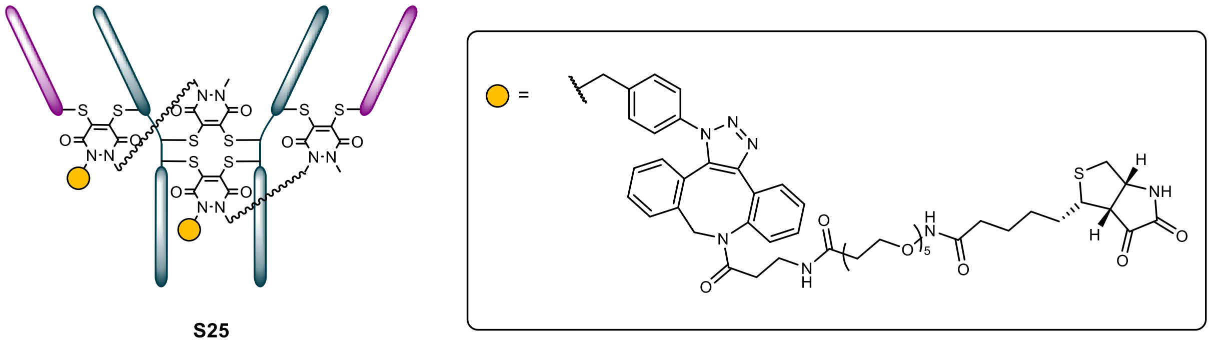

S25

To a solution of conjugate **S24** (100 µL, 20 µM) in BBS (25 mM borate, 25 mM NaCl, pH 8.0, 2 mM EDTA, 2% DMSO, pH 8.0) was added DBCO-biotin (1 µL, 20 mM in DMSO, 10 eq.) and the reaction incubated for 24 h at 37 °C under constant agitation (300 rpm) to give the product **S25** (expected mass: 145,997 Da, observed mass: 146,007 Da). The concentration of the sample and PDAR were assessed by UV-Vis spectroscopy and LC-MS (method 1b).

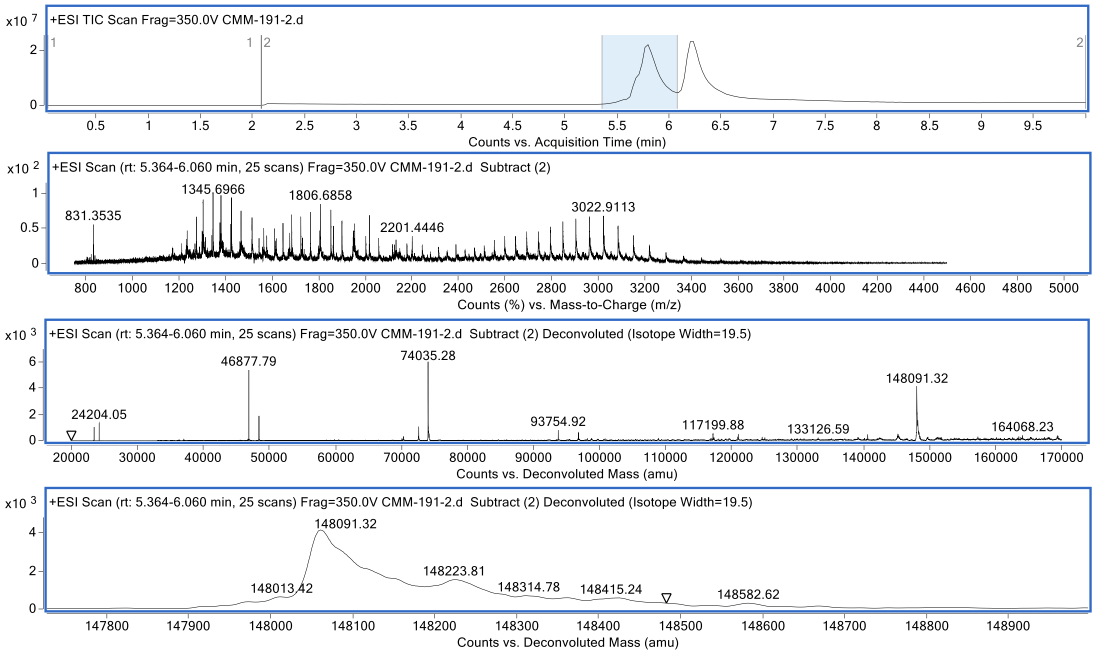

Figure S91: (i) TIC LC-MS trace (top), (ii) non-deconvoluted LC-MS trace (upper middle), (iii) deconvoluted MS data (lower middle, wide range), (iv) deconvoluted MS data (bottom, zoom in range).

HC S378C PD conjugate 22 (HC S378C Thio-trastuzumab one-pot reaction with BCN PD 1)

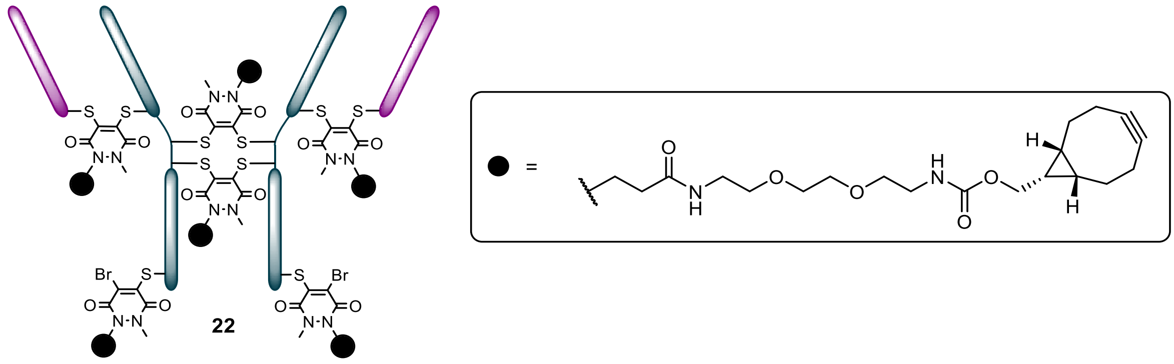

To a solution of HC S378C thio-trastuzumab **7** (70 µL, 13.2 µM) in BBS (25 mM borate, 25 mM NaCl, 2 mM EDTA, 2% DMSO, pH 8.0) was added TCEP.HCl (20 mM in dH_2O, A eq.) and the reaction incubated at 37 °C for B h under constant agitation (300 rpm). After this time, the TCEP was removed *via* ultrafiltration and BCN PD (20 mM in DMSO, C eq.) added to reduced antibody **21**. The reaction was incubated at 37 °C for 3 h under constant agitation (300 rpm). After this, excess reagents were removed to give conjugate **22** (expected mass 148,316 Da, observed mass 148,315 Da) which was analysed by LC-MS (method 1a), UV-Vis spectroscopy and SDS-PAGE.

	TCEP Eq. (A)	Reduction Time (B)	BCN PD Eq. (C)
**22a**	10	8.0	14
**22b**	10	8.0	25

Table S11. Conditions used to form **22**.

22a

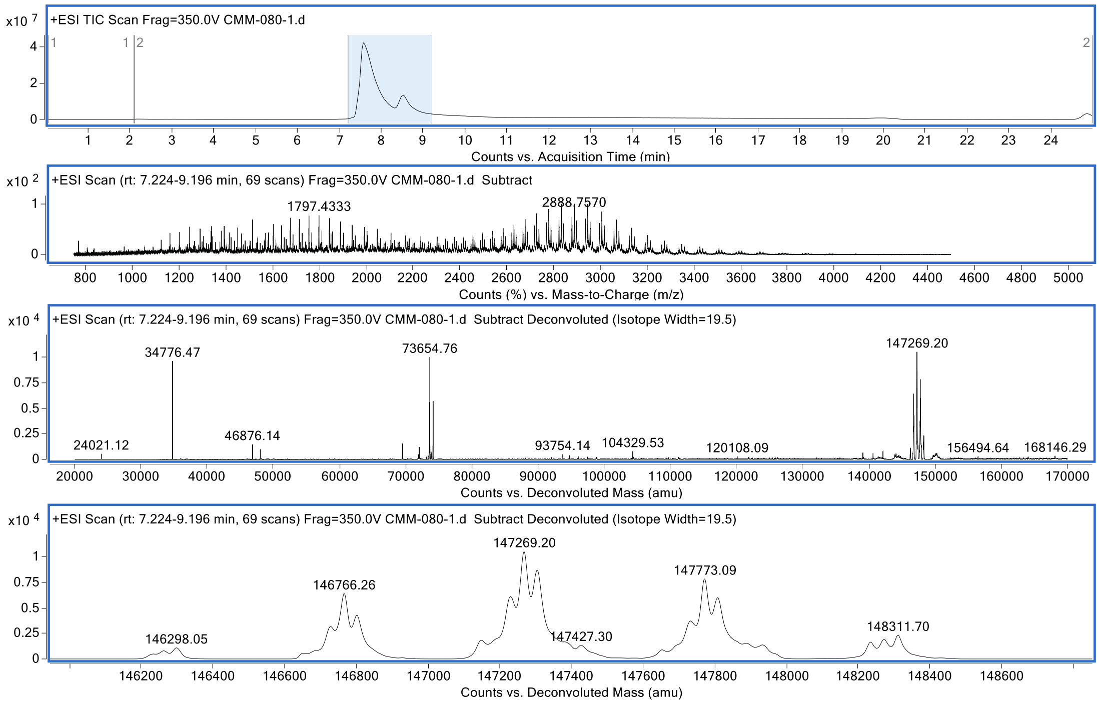

Figure S92: (i) TIC LC-MS trace (top), (ii) non-deconvoluted LC-MS trace (upper middle), (iii) deconvoluted MS data (lower middle, wide range), (iv) deconvoluted MS data (bottom, zoom in range).

22b

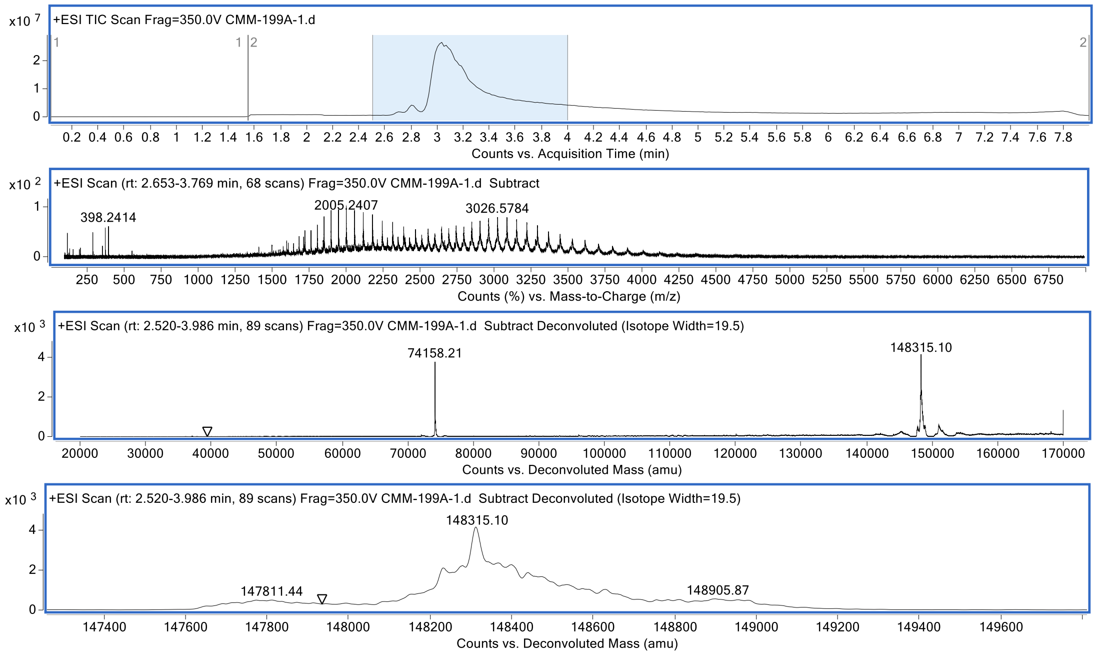

Figure S93: (i) TIC LC-MS trace (top), (ii) non-deconvoluted LC-MS trace (upper middle), (iii) deconvoluted MS data (lower middle, wide range), (iv) deconvoluted MS data (bottom, zoom in range).

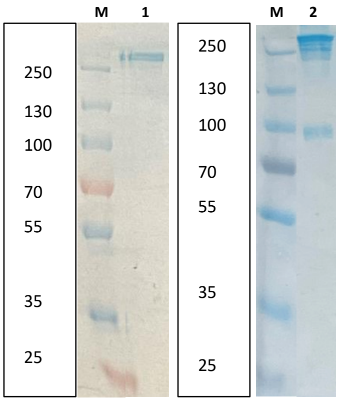

Figure S94: SDS-PAGE gel: M. Ladder, 1. Conjugate **22a**, 2. Conjugate **22b**.

HC S378C PD fluorophore conjugate 23 (HC S378C thio-trastuzumab one-pot reaction with BCN PD 1 + Azide-Fluor 488)

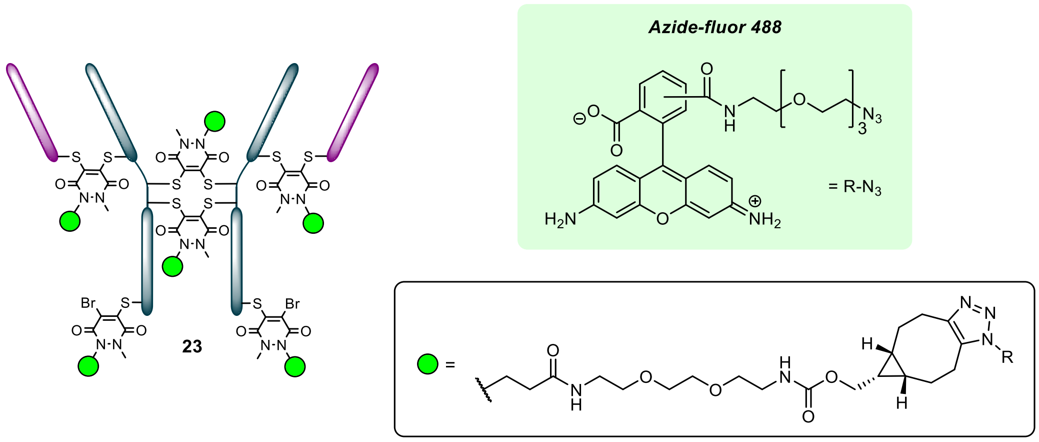

To a solution of conjugated HC S378C thio-trastuzumab **22** (100 µL, 16 µM) in BBS (25 mM borate, 25 mM NaCl, 2 mM EDTA, 2% DMSO, pH 8.0) was added Azide-fluor 488 (3.2 µL, 10 M in DMSO, 20 eq.). The reaction was incubated at 37 °C for 16 h under constant agitation (300 rpm). After this, excess reagents were removed to give conjugate **23** (expected mass 151,764 Da, observed mass 151,759 Da) which was analysed by LC-MS (method 1b), UV-Vis spectroscopy and SDS-PAGE.

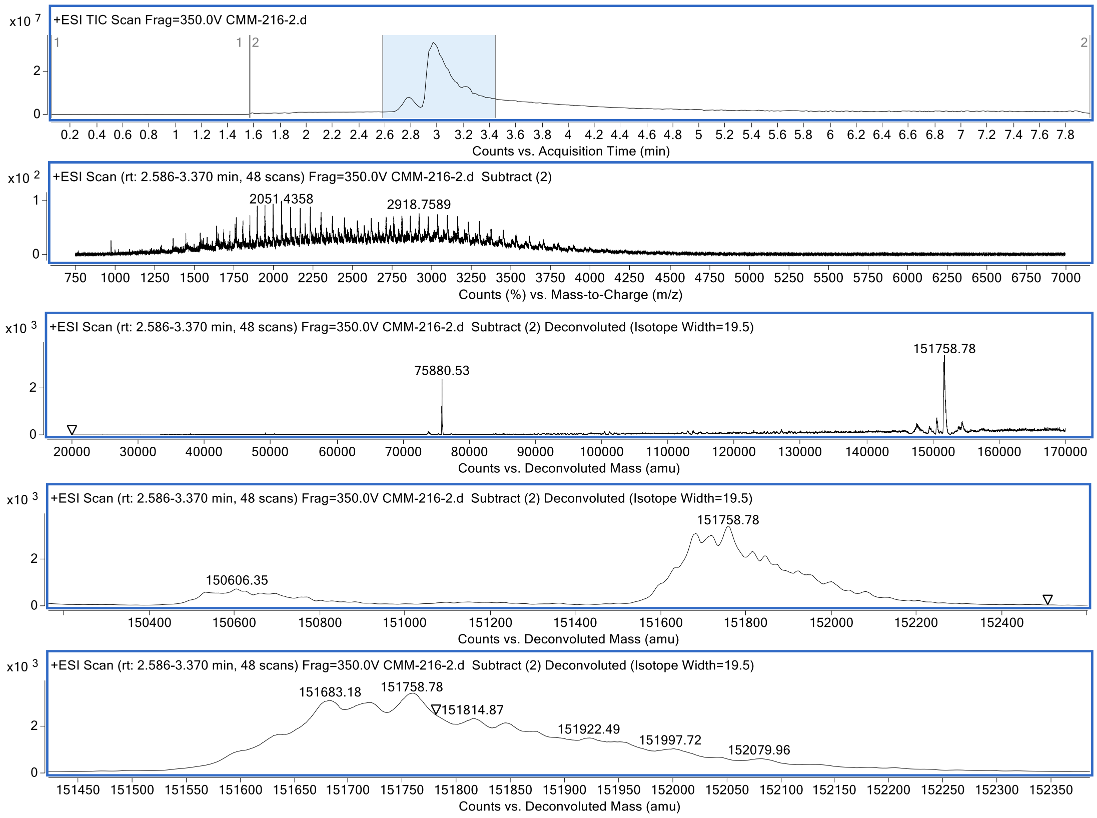

Figure S95: (i) TIC LC-MS trace (top), (ii) non-deconvoluted LC-MS trace (upper middle), (iii) deconvoluted MS data (lower middle, wide range), (iv) and (v) deconvoluted MS data (bottom, zoom in range)

HC S378C PD aniline fluorophore conjugate 24 (HC S378C Thio-Trastuzumab One-Pot Reduction/Re-bridging with BCN PD 1 + *p*-Anisidine + Azide-Fluor 488)

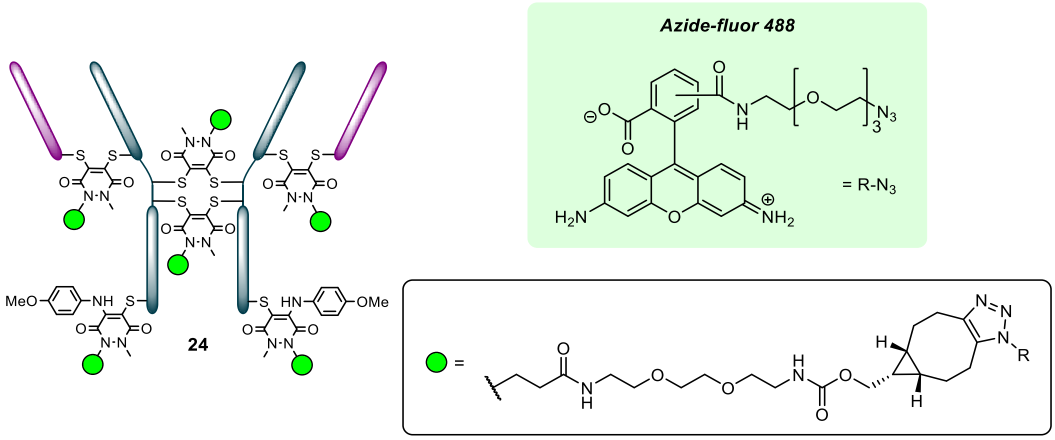

To a solution of conjugated HC S378C thio-trastuzumab **23** (30 µL, 20 µM) in BBS (25 mM borate, 25 mM NaCl, 2 mM EDTA, 2% DMSO, pH 8.0) was added *p*-anisidine (0.3 µL, 1 M in DMSO, 1000 eq.). The reaction was incubated at 37 °C for 16 h under constant agitation (300 rpm). After this, excess reagents were removed to give conjugate **24** (expected mass 151,858 Da, observed mass 151,849 Da) which was analysed by LC-MS (method 1a), UV-Vis spectroscopy and SDS-PAGE.

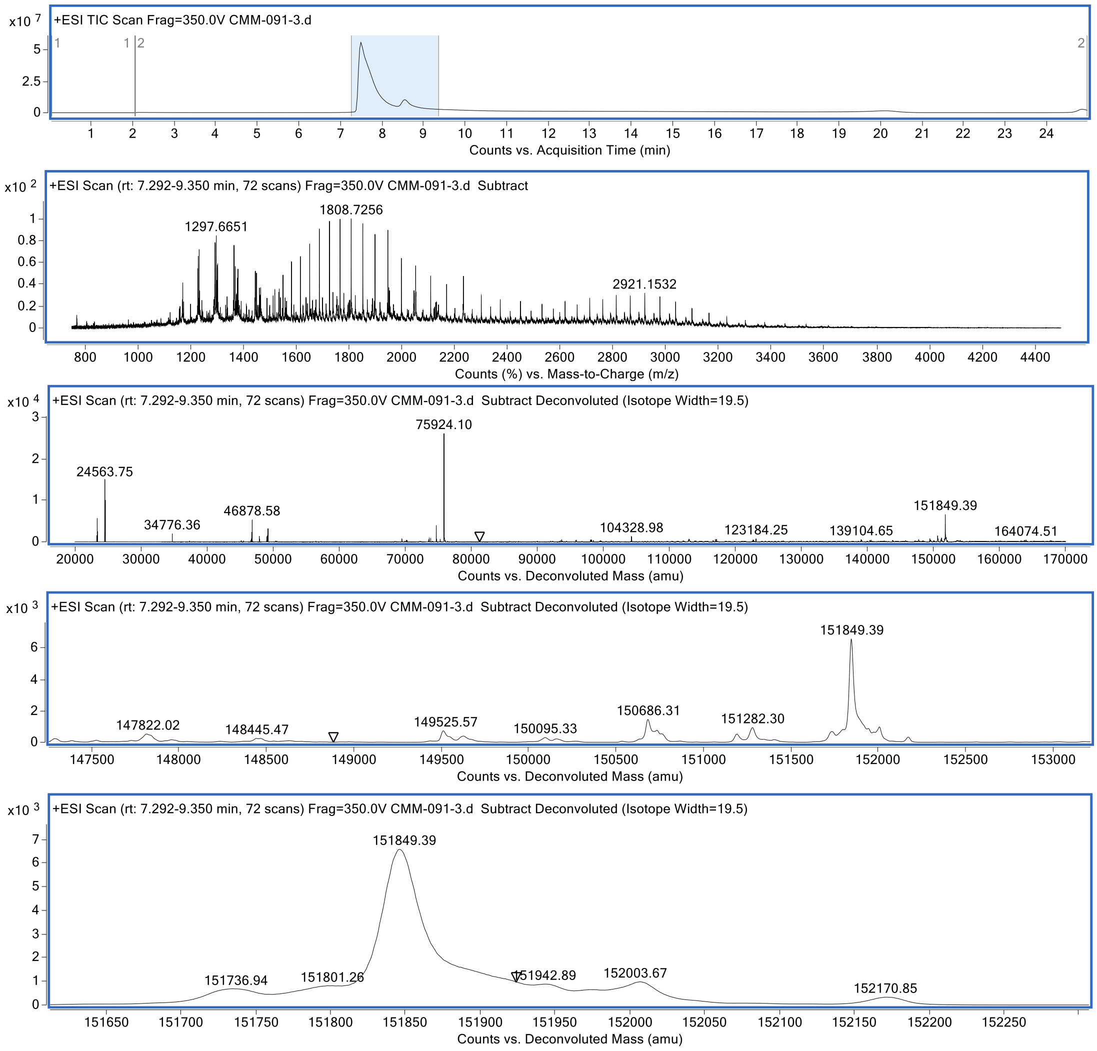

Figure S96: (i) TIC LC-MS trace (top), (ii) non-deconvoluted LC-MS trace (upper middle), (iii) deconvoluted MS data (lower middle, wide range), (iv) deconvoluted MS data (bottom, zoom in range).

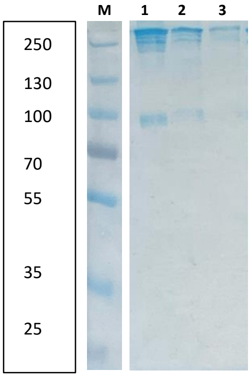

Figure S97: SDS-PAGE gel: M. Ladder, 1. Conjugate **22b**, 2. Conjugate **23**, 3. Conjugate **24**.

LC S168C PD conjugate 26 (LC S168C Thio-trastuzumab one-pot reaction with BCN PD 1)

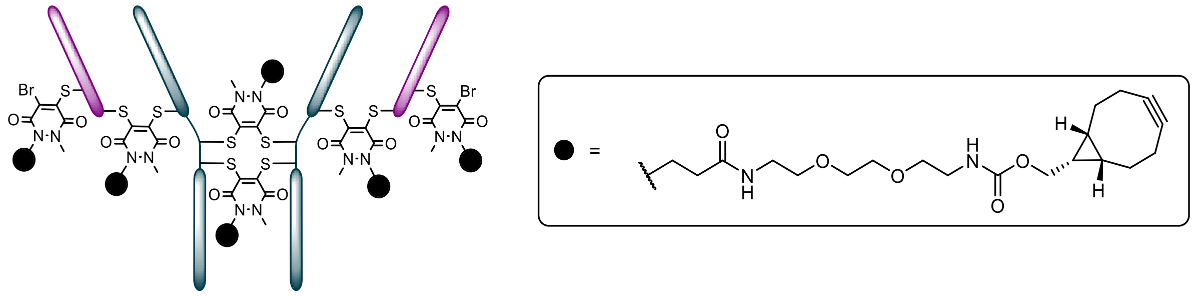

26

To a solution of LC S168C thio-trastuzumab **6** (70 µL, 20 µM) in BBS (25 mM borate, 25 mM NaCl, 2 mM EDTA, 2% DMSO, pH 8.0) was added TCEP.HCl (2.8 µL, 20 mM in dH_2O, 40 eq.) and the reaction incubated at 37 °C for 3 h under constant agitation (300 rpm). After this time, the TCEP was removed *via* ultrafiltration and BCN PD (1.4 µL, 20 mM in DMSO, 20 eq.) added to reduced antibody **25**. The reaction was incubated at 37 °C for 3 h under constant agitation (300 rpm). After this, excess reagents were removed to give conjugate **26** (expected mass 148,316 Da, observed mass 148,316 Da) which was analysed by LC-MS (method 1b), UV-Vis spectroscopy and SDS-PAGE.

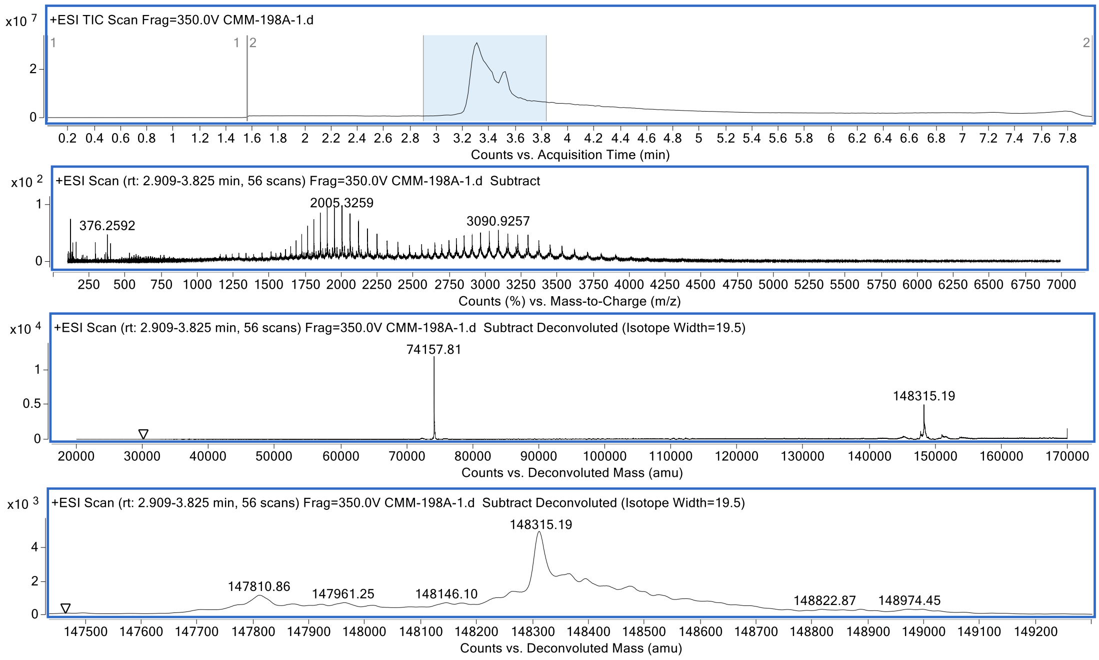

Figure S98: (i) TIC LC-MS trace (top), (ii) non-deconvoluted LC-MS trace (upper middle), (iii) deconvoluted MS data (lower middle, wide range), (iv) deconvoluted MS data (bottom, zoom in range).

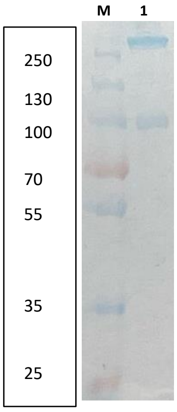

Figure S99: SDS-PAGE gel: M. Ladder, 1. Conjugate **26**.

LC S168C PD fluorophore conjugate 27 (LC S168C thio-trastuzumab one-pot reaction with BCN PD 1, clicked with Azide-Fluor 488)

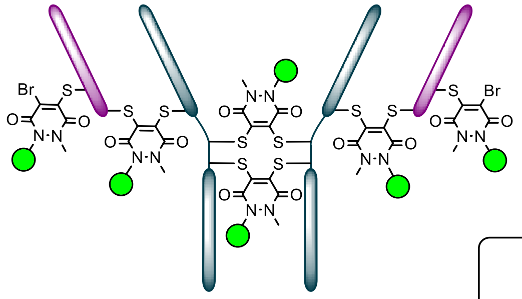

27

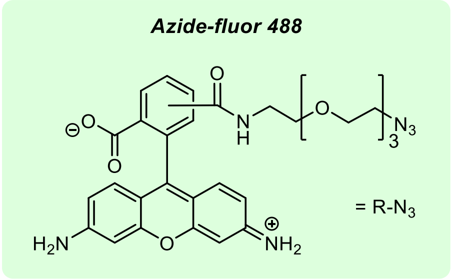

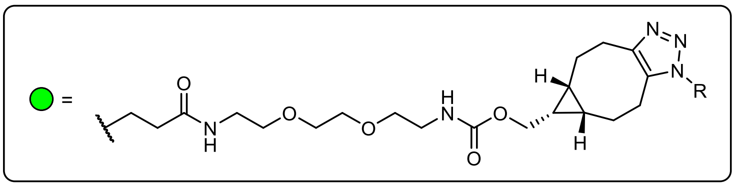

To a solution of conjugated LC S168C thio-trastuzumab **26** (50 µL, 16 µM) in BBS (25 mM borate, 25 mM NaCl, 2 mM EDTA, 2% DMSO, pH 8.0) was added Azide-fluor 488 (1.6 µL, 10 mM in dH_2O, 20 eq.) and the reaction incubated at 37 °C for 16 h under constant agitation (300 rpm). After this, excess reagents were removed to give conjugate **27** (expected mass 148,322 Da, observed mass 148,320 Da) which was analysed by LC-MS (method 1b), UV-Vis spectroscopy and SDS-PAGE.

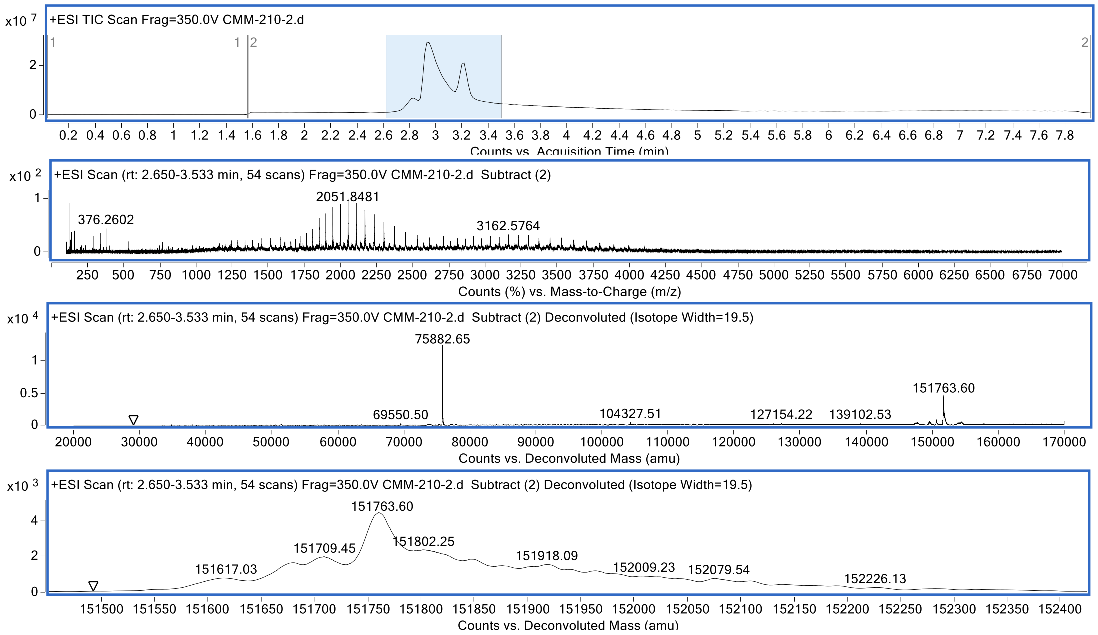

Figure S100: (i) TIC LC-MS trace (top), (ii) non-deconvoluted LC-MS trace (upper middle), (iii) deconvoluted MS data (lower middle, wide range), (iv) deconvoluted MS data (bottom, zoom in range).

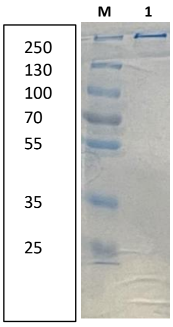

Figure S101: SDS-PAGE gel: M. Ladder, 1. Conjugate **27**.

LC S168C PD aniline fluorophore conjugate 28 (LC S168C thio-trastuzumab one-pot reaction with BCN PD 1, reacted with Azide-Fluor 488 and N_3 Aniline)

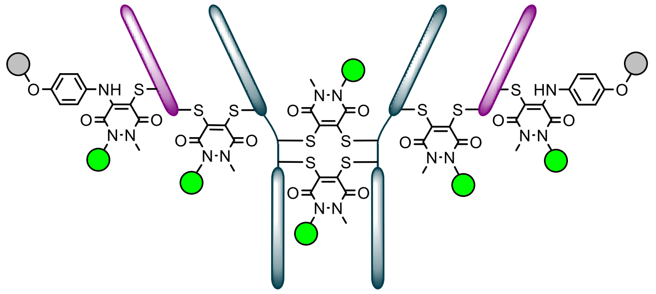

28

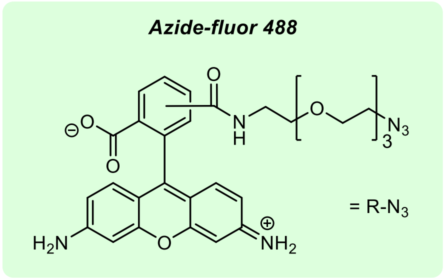

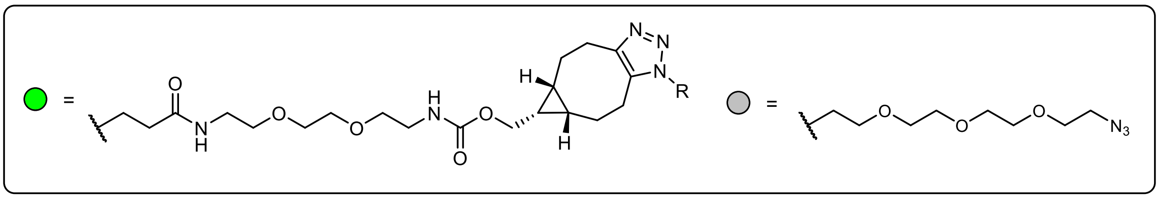

To a solution of conjugated LC S168C thio-trastuzumab **27** (100 µL, 20 µM) in BBS (25 mM borate, 25 mM NaCl, 2 mM EDTA, 2% DMSO, pH 8.0) was added N_3 aniline **3** (1 µL, 1 M in DMSO, 1000 eq.) and the reaction incubated at 37 °C for 16 h under constant agitation (300 rpm). After this, excess reagents were removed to give conjugate **28** (expected mass 152,225 Da, observed mass 152,221 Da) which was analysed by LC-MS (method 1b), UV-Vis spectroscopy and SDS-PAGE.

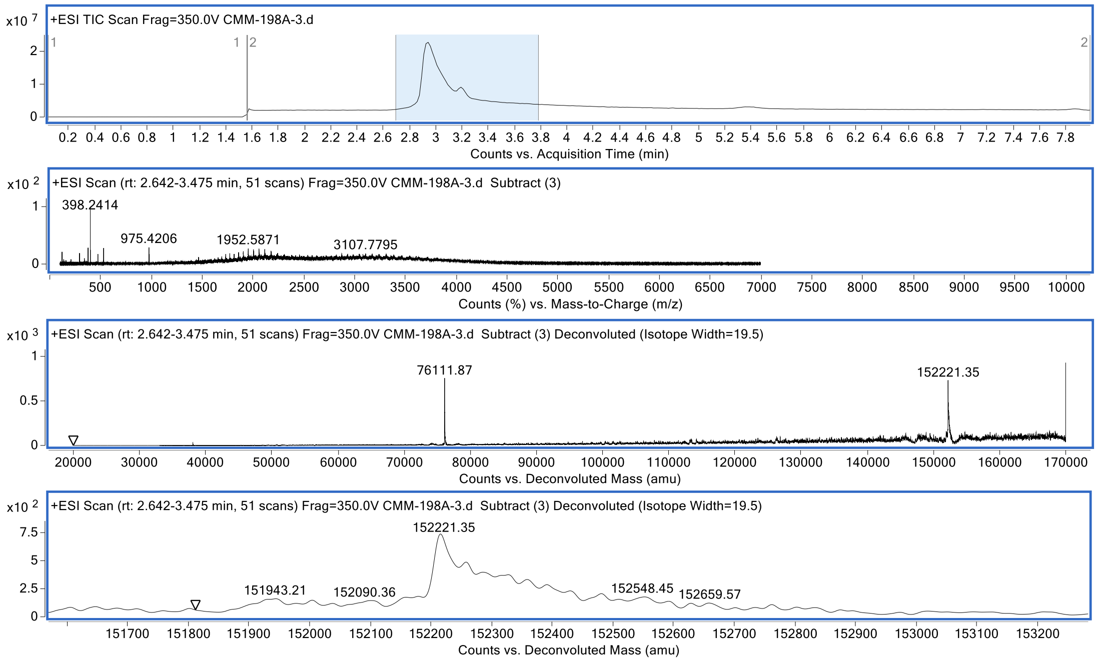

Figure S102: (i) TIC LC-MS trace (top), (ii) non-deconvoluted LC-MS trace (upper middle), (iii) deconvoluted MS data (lower middle, wide range), (iv) deconvoluted MS data (bottom, zoom in range).

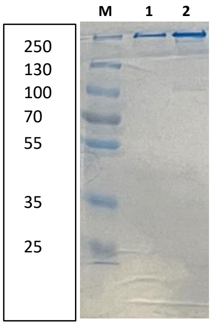

Figure S103: SDS-PAGE gel: M. Ladder, 1. Conjugate **27**, 2. Conjugate **28**.

LC S168C PD aniline fluorophore conjugate 29 (LC S168C thio-trastuzumab one-pot reaction with Mepstra PD 1, reacted with Azide-fluor 488, N_3 Aniline 3 and DBCO-biotin)

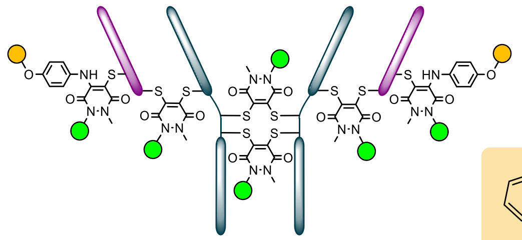

29

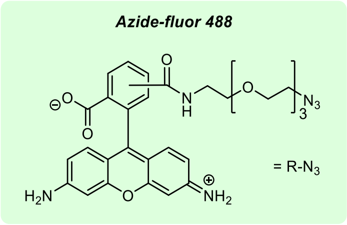

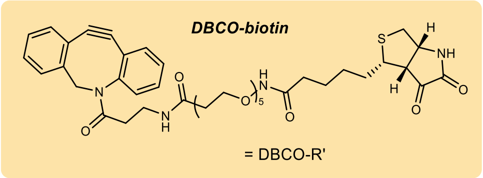

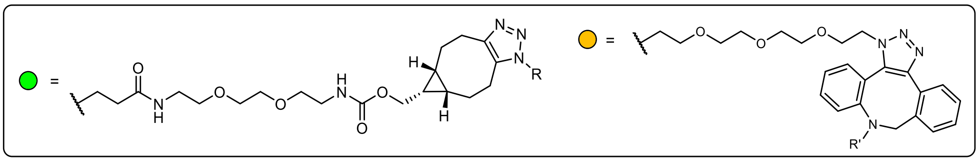

To a solution of conjugated LC S168C thio-trastuzumab **28** (65 µL, 13.9 µM) in BBS (25 mM borate, 25 mM NaCl, 2 mM EDTA, 2% DMSO, pH 8.0) was added DBCO-biotin (0.9 µL, 10 mM in dH_2O, 20 eq.) and the reaction incubated at 22 °C for 72 h under constant agitation (300 rpm). After this, excess reagents were removed to give conjugate **29** (expected mass 153,725 Da, observed mass 153,719 Da) which was analysed by LC-MS (method 1b), UV-Vis spectroscopy and SDS-PAGE.

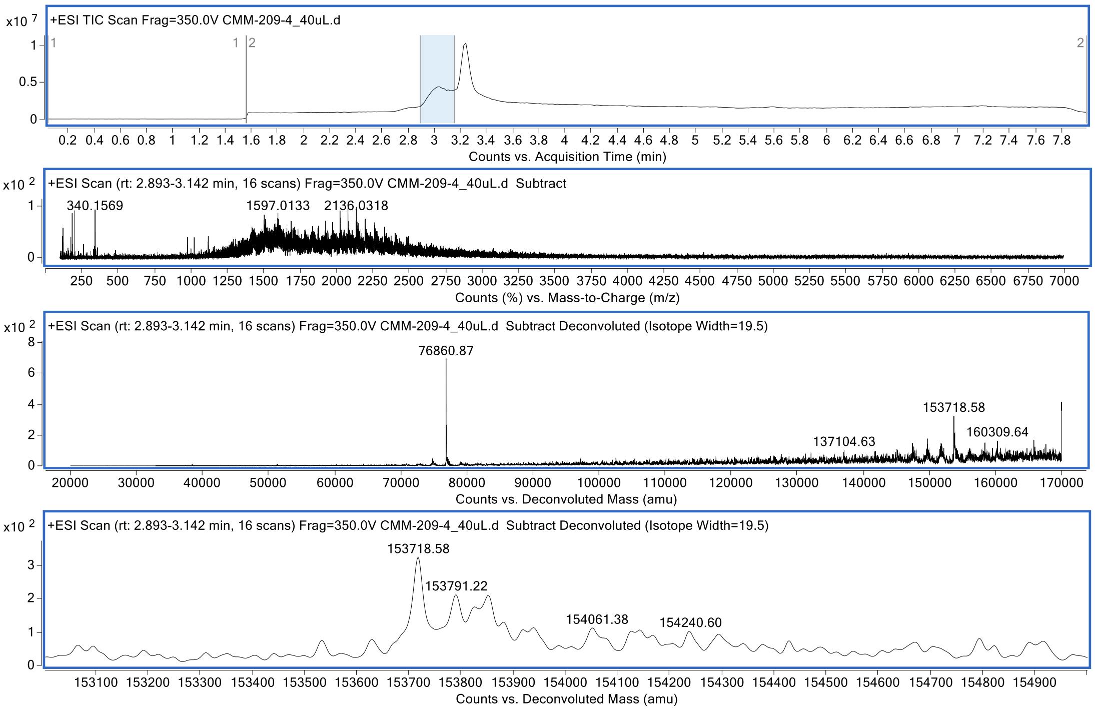

Figure S104: (i) TIC LC-MS trace (top), (ii) non-deconvoluted LC-MS trace (upper middle), (iii) deconvoluted MS data (lower middle, wide range), (iv) deconvoluted MS data (bottom, zoom in range). *Note*: the TIC peak on the right is PNGase, but as the sample is dilute it has not been deconvoluted as otherwise it dominates the mass spec.

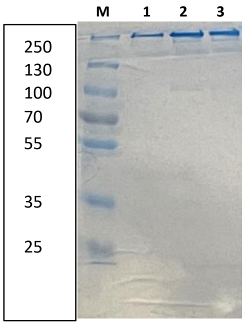

Figure S105: SDS-PAGE gel: M. Ladder, 1. Conjugate **27**, 2. Conjugate **28**, 3. Conjugate **29**.

HC S378C PD conjugate S26 (HC S378C Thio-trastuzumab conjugated to diEt PD 4)

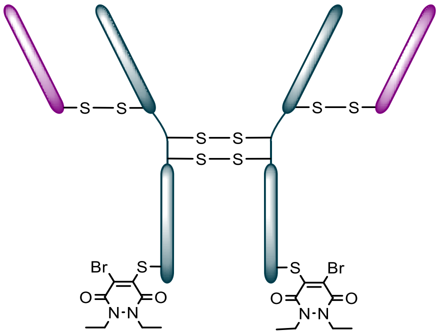

S26

To a solution of uncapped HC S378C thio-trastuzumab **7** (45 µL, 20 µM) in BBS (25 mM borate, 25 mM NaCl, 2 mM EDTA, 2% DMSO, pH 8.0) was added diEt PD **4** (0.18 µL, 20 mM in DMSO, 4 eq.) and the reaction incubated at 37 °C for 3 h under constant agitation (300 rpm). After this time, excess reagents were removed to give conjugate **S26** (expected mass 145,635 Da, observed mass 145,632 Da) which was analysed by LC-MS (method 1a), UV-Vis spectroscopy and SDS-PAGE.

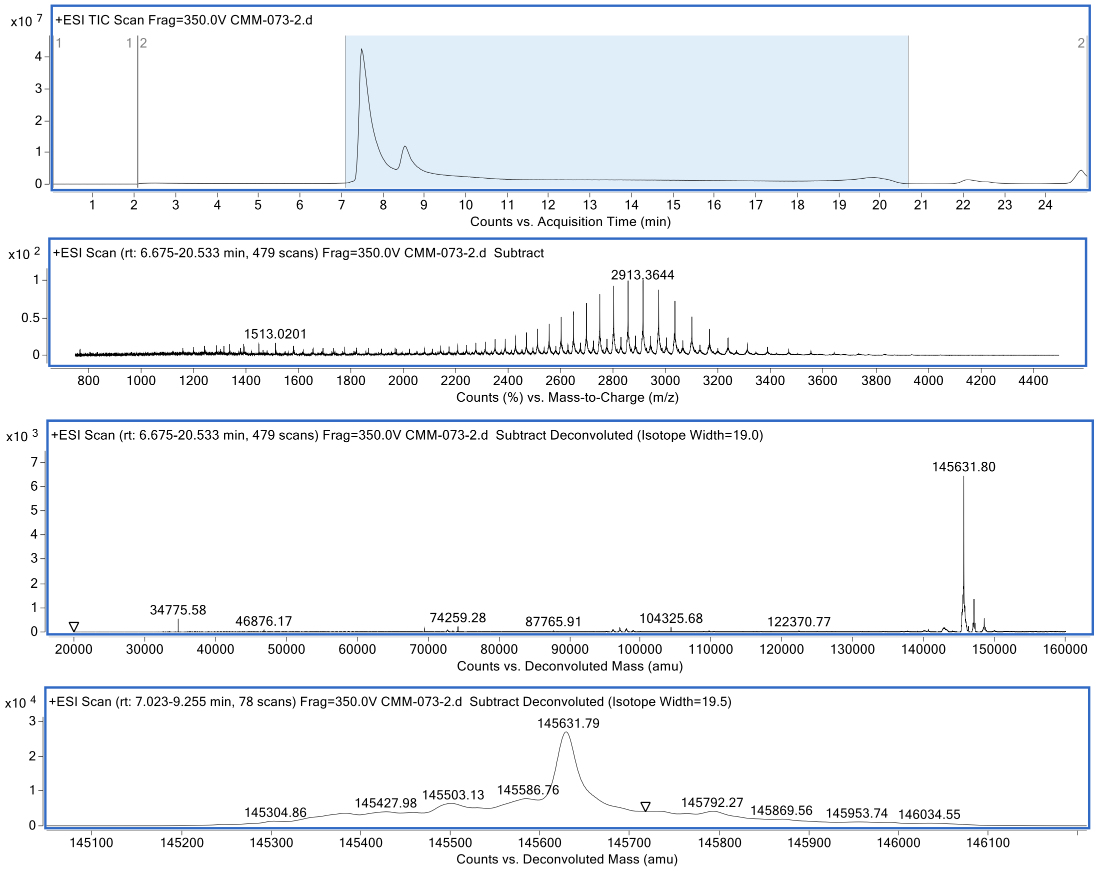

Figure S106: (i) TIC LC-MS trace (top), (ii) non-deconvoluted LC-MS trace (upper middle), (iii) deconvoluted MS data (lower middle, wide range), (iv) deconvoluted MS data (bottom, zoom in range).

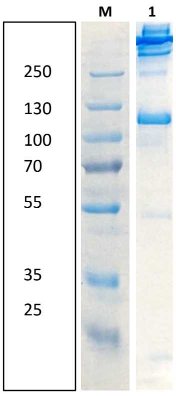

Figure S107: SDS-PAGE gel: M. Ladder, 1. Conjugate **S26**.

HC S378C PD conjugate S27 (HC S378C Thio-trastuzumab conjugated to diEt PD 4 and *p*-anisidine)

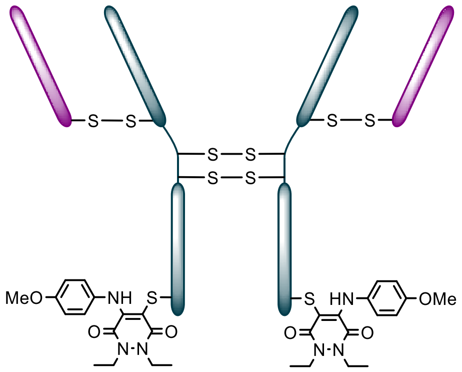

S27

To a solution of HC S378C thio-trastuzumab conjugated to diEt PD **S26** (50 µL, 20 µM) in BBS (25 mM borate, 25 mM NaCl, 2 mM EDTA, 2% DMSO, pH 8.0) was added *p*-anisidine (1 µL, 1 M in DMSO, 1000 eq.) and the reaction incubated at 37 °C for 16 h under constant agitation (300 rpm). After this time, excess reagents were removed to give conjugate **S27** (expected mass 145,715 Da, observed mass 145,718 Da) which was analysed by LC-MS (method 1a), UV-Vis spectroscopy and SDS-PAGE.

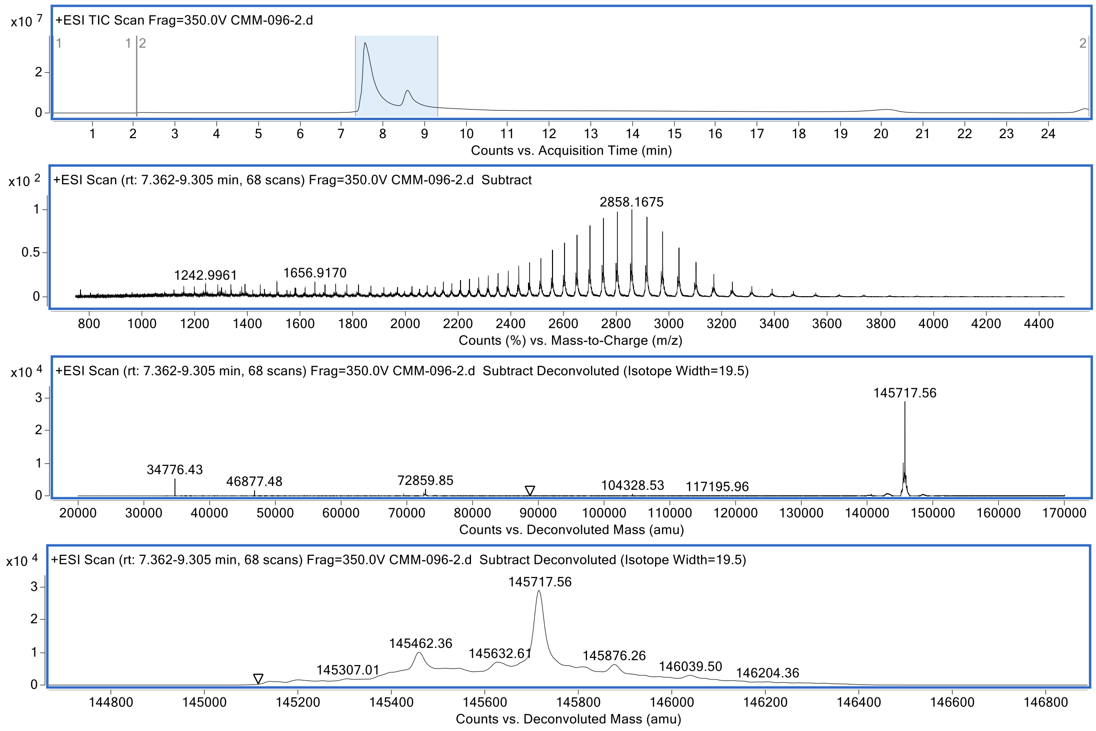

Figure S108: (i) TIC LC-MS trace (top), (ii) non-deconvoluted LC-MS trace (upper middle), (iii) deconvoluted MS data (lower middle, wide range), (iv) deconvoluted MS data (bottom, zoom in range).

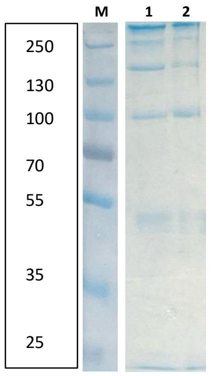

Figure S109: SDS-PAGE gel: M. Ladder, 1. Conjugate **S26**, 2. Conjugate **S27**.

HC S378C PD conjugate S27 (HC S378C Thio-trastuzumab conjugated to diEt PD 4 and *p*-anisidine) TCEP Stability Test

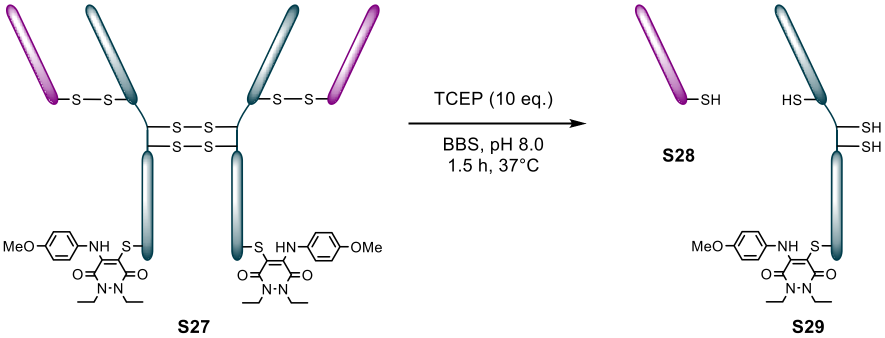

To a solution of conjugated HC S378C thio-trastuzumab **S27** (30 µL, 20 µM) in BBS (25 mM borate, 25 mM NaCl, 2 mM EDTA, 2% DMSO, pH 8.0) was added TCEP.HCl (0.3 µL, 20 mM in DMSO, 10 eq.) and the reaction incubated at 37 °C for 1.5 h under constant agitation (300 rpm) to give light chain **S28** (expected mass = 23,440 Da, observed mass = 23412 Da) and conjugated heavy chain **S29** (expected mass = 49,420 Da, observed mass = 49,422 Da). After this, excess reagents were removed and the sample analysed by LC-MS (method 1a).

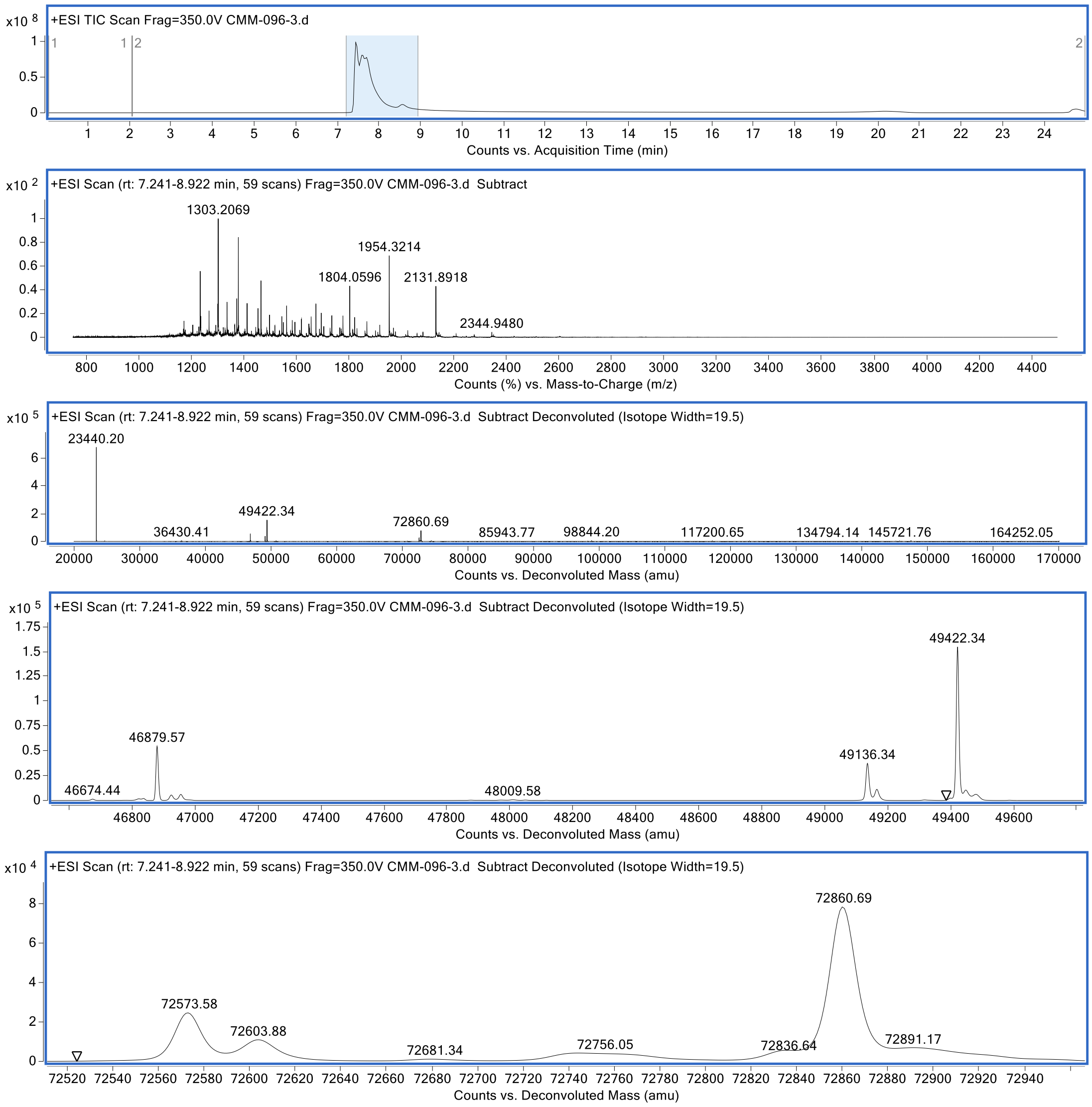

Figure S110: (i) TIC LC-MS trace (top), (ii) non-deconvoluted LC-MS trace (upper middle), (iii) deconvoluted MS data (lower middle, wide range), (iv) and (v) deconvoluted MS data (bottom, zoom in range).

GFP S147C S30

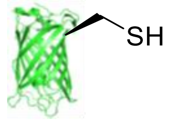

S30

To a solution of GFP S147C dimer (150 µL, 20 µM) in BBS (25 mM borate, 25 mM NaCl, 2 mM EDTA, 2% DMSO, pH 8.0) was added TCEP.HCl (3 µL, 50 mM in 5 × BBS, 50 eq.) and the solution incubated at 37 °C under constant agitation (300 rpm) for 2 h to give GFP S147C **S30**. The product was characterised by LC-MS (method 1a*).

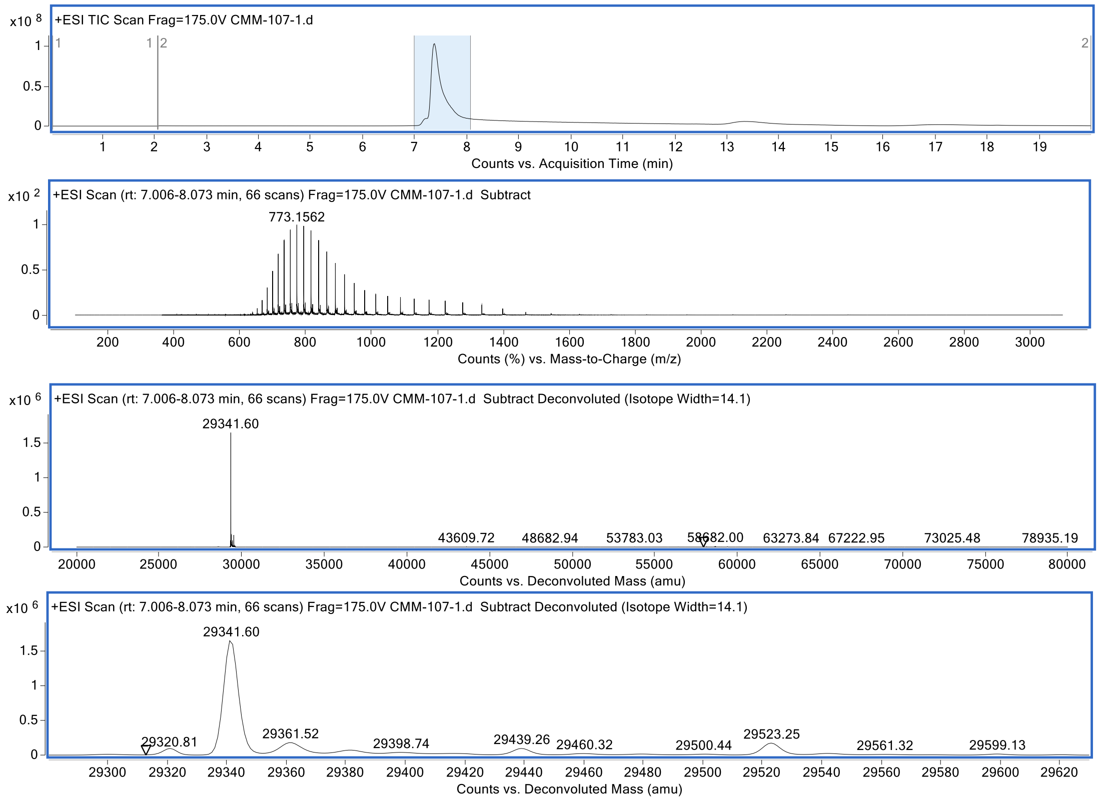

Figure S111: (i) TIC LC-MS trace (top), (ii) non-deconvoluted LC-MS trace (upper middle), (iii) deconvoluted MS data (lower middle, wide range), (iv) deconvoluted MS data (bottom, zoom in range).

GFP PD conjugate S31 (GFP S147C S30 conjugated to BCN PD 1)

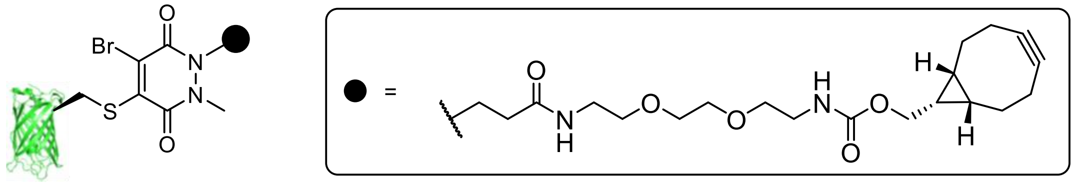

S31

To a solution of GFP S147C **S30** (144 µL, 20 µM) in BBS (25 mM borate, 25 mM NaCl, 2 mM EDTA, 2% DMSO, pH A) was added BCN PD **1** (20 mM in DMSO, 50 eq.) and the reaction incubated at 37 °C under constant agitation (300 rpm) for 4 h to give GFP PD conjugate **S31** (expected mass 29,925 Da, observed mass 29,923 Da). The product was analysed by LC-MS (method 1a*).

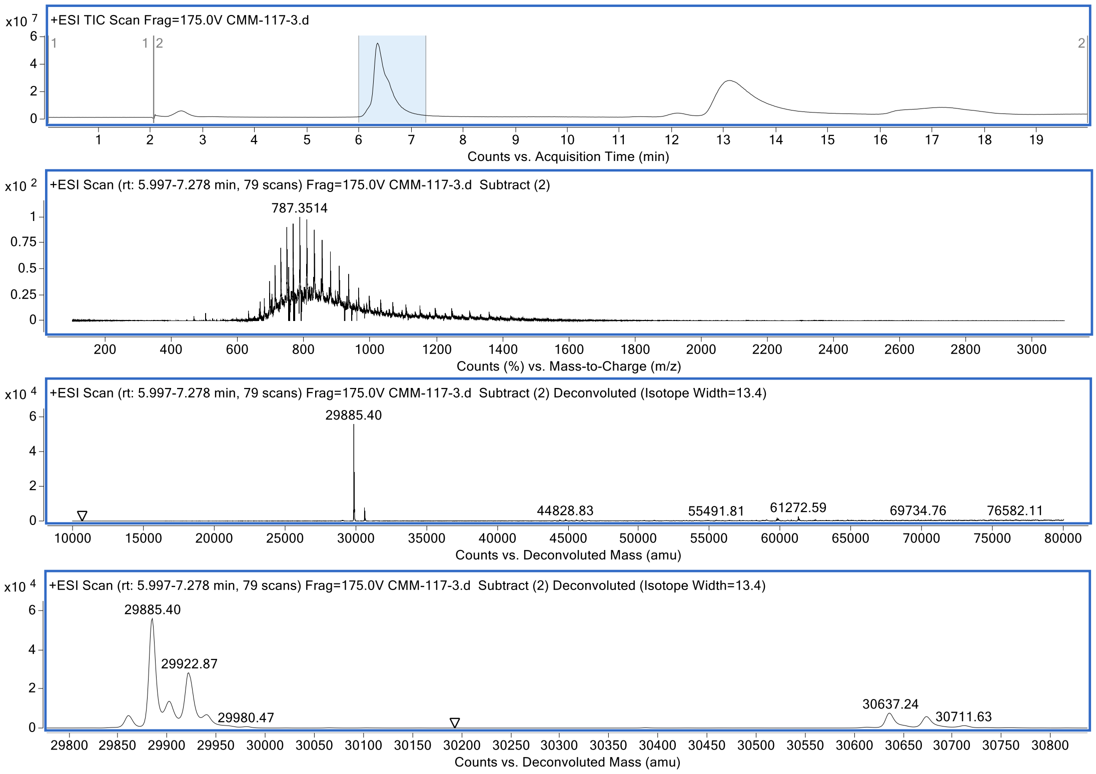

Figure S112: (i) TIC LC-MS trace (top), (ii) non-deconvoluted LC-MS trace (upper middle), (iii) deconvoluted MS data (lower middle, wide range), (iv) deconvoluted MS data (bottom, zoom in range)

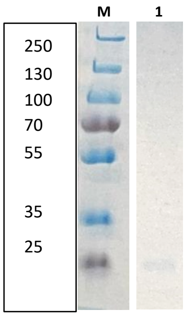

Figure S113: SDS-PAGE gel: M. Ladder, 1. Conjugate **S31**.

GFP S147C PD Conjugate S31 (GFP S147C Conjugated to BCN PD 1) TCEP Stability Test

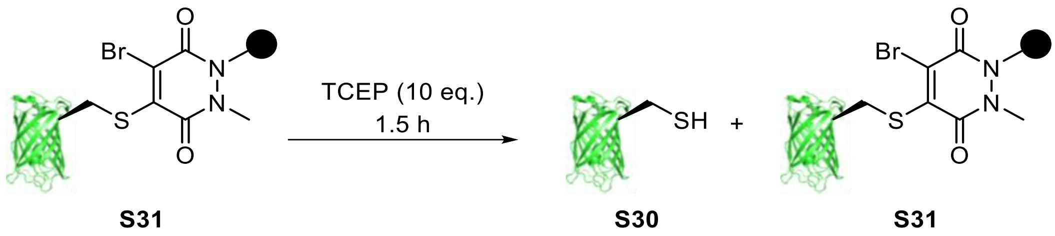

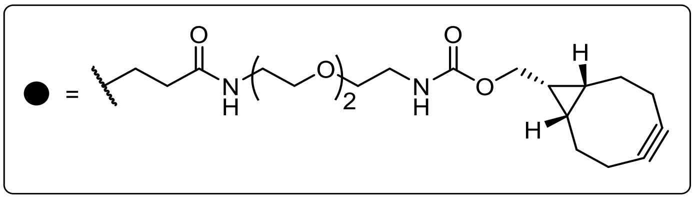

To a solution of **S31** (30 µL, 20 µM) in BBS (25 mM borate, 25 mM NaCl, 2 mM EDTA, 2% DMSO, pH 8.0) was added TCEP.HCl (0.3 µL, 20 mM in dH_2O, 10 eq.) and the reaction incubated at 37 °C for 1.5 h under constant agitation (300 rpm). The reaction was analysed by LC-MS (method 1a*).

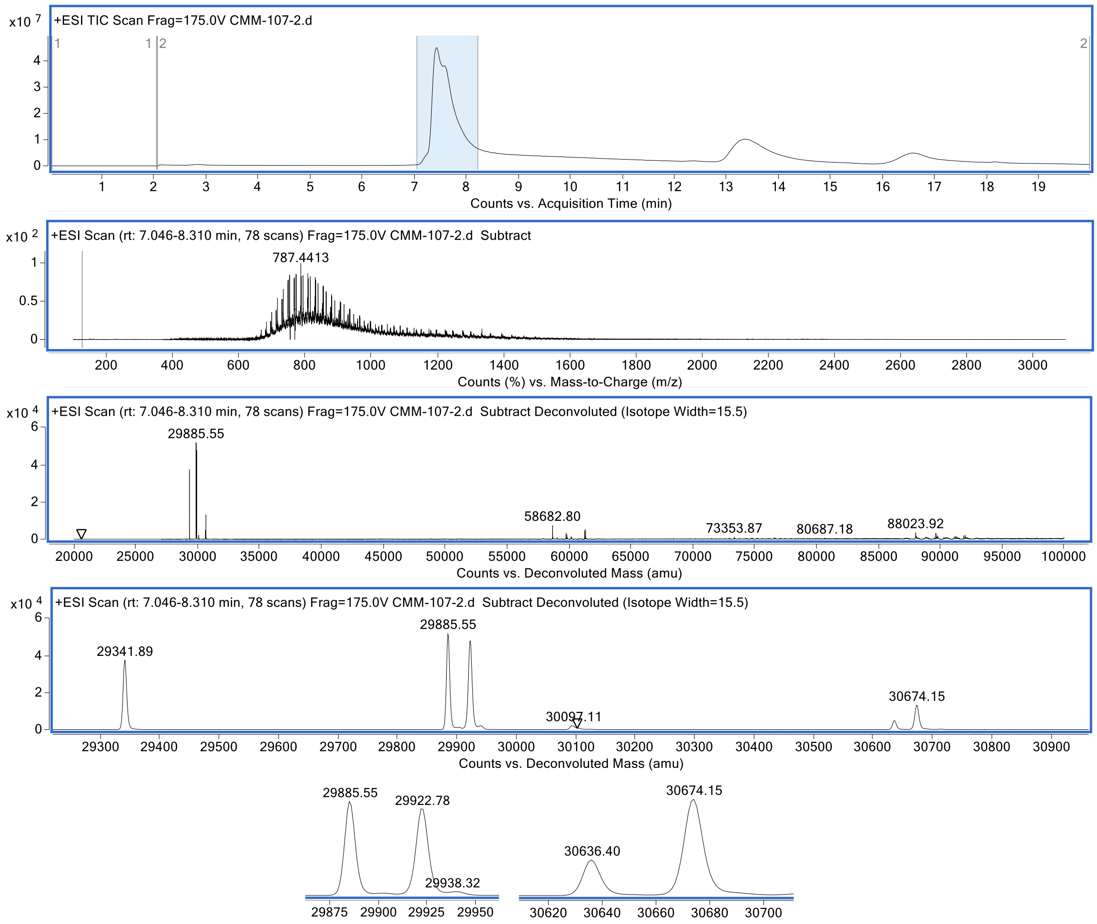

Figure S114: (i) TIC LC-MS trace (top), (ii) non-deconvoluted LC-MS trace (upper middle), (iii) deconvoluted MS data (lower middle, wide range), (iv) and (v) deconvoluted MS data (bottom, zoom in range).

GFP PD aniline conjugate S32 (GFP S147C S30 conjugated to BCN PD 1 and *p*-anisidine)

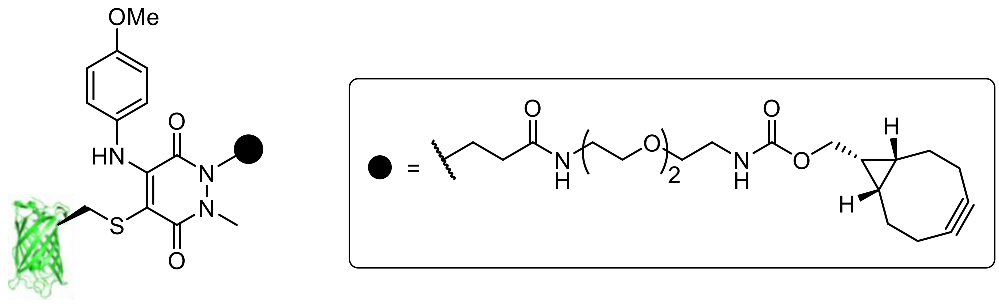

S32

To a solution of GFP PD conjugate **S31** (108 µL, 20 µM) in BBS (25 mM borate, 25 mM NaCl, 2 mM EDTA, 2% DMSO, pH 8) was added *p*-anisidine (1.16 µL, 1 M in DMSO, 1000 eq.), and the reaction incubated at 37 °C under constant agitation (300 rpm) for 16 h to give GFP PD aniline conjugate **S32** (expected mass 29,967 Da, observed mass 29,966 Da). The product was analysed by LC-MS (method 1a*).

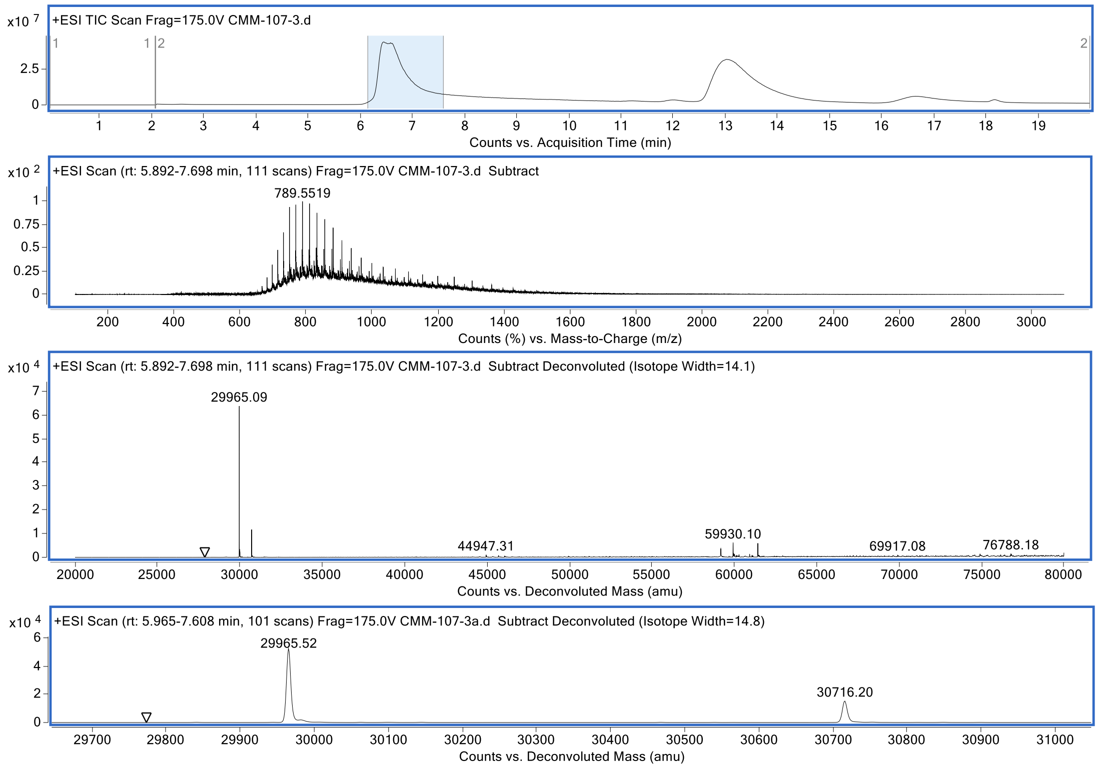

Figure S115: (i) TIC LC-MS trace (top), (ii) non-deconvoluted LC-MS trace (upper middle), (iii) deconvoluted MS data (lower middle, wide range), (iv) deconvoluted MS data (bottom, zoom in range).

GFP S147C PD aniline conjugate S32 (GFP S147C Conjugated to BCN PD 1 and *p*-anisidine) TCEP Stability Test

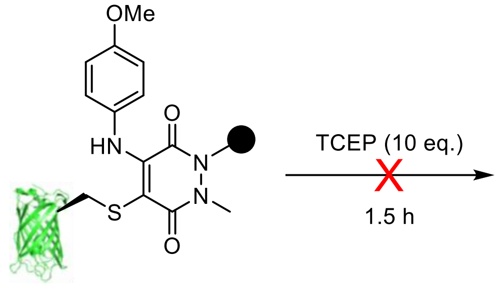

S32

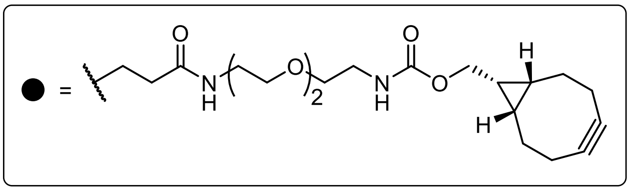

To a solution of GFP PD aniline conjugate **S32** (30 µL, 20 µM) in BBS (25 mM borate, 25 mM NaCl, 2 mM EDTA, 2% DMSO, pH 8.0) was added TCEP.HCl (0.3 µL, 20 mM in dH_2O, 10 eq.), and the reaction incubated at 37 °C for 1.5 h under constant agitation (300 rpm). No reaction was observed by LC-MS (method 1a*).

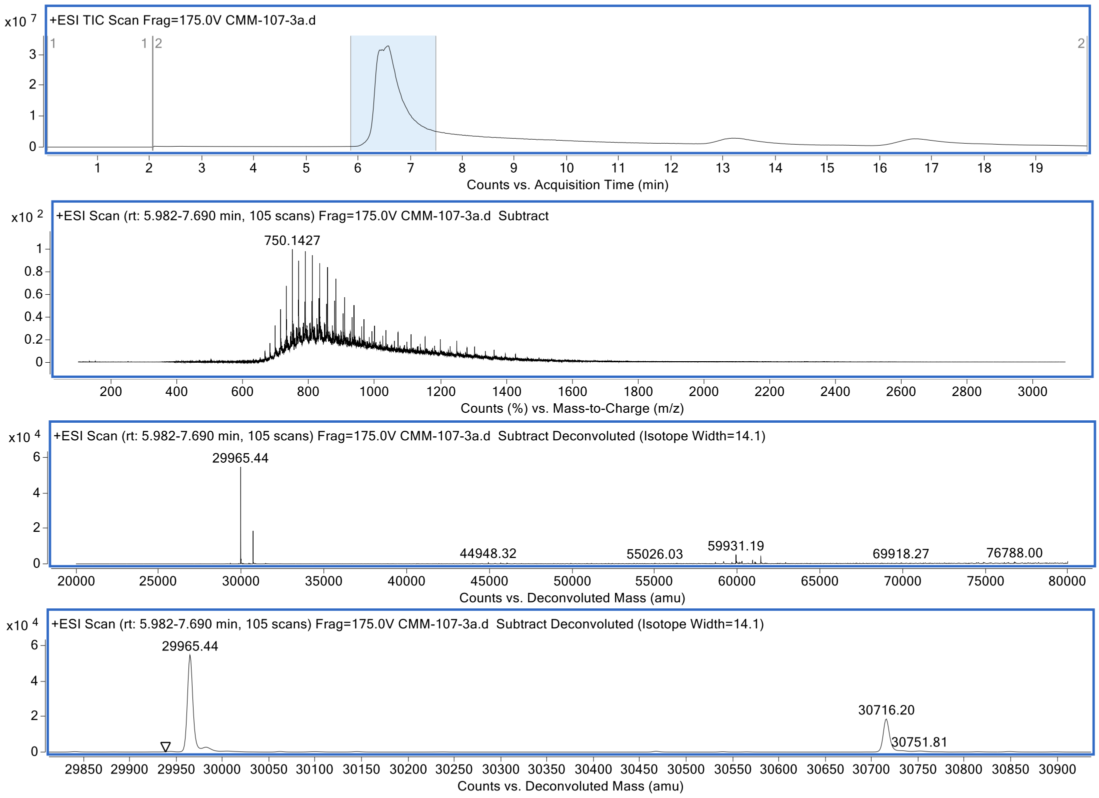

Figure S116: (i) TIC LC-MS trace (top), (ii) non-deconvoluted LC-MS trace (upper middle), (iii) deconvoluted MS data (lower middle, wide range), (iv) deconvoluted MS data (bottom, zoom in range).

GFP PD aniline fluorophore conjugate S33 (GFP S147C S30 conjugated to BCN PD 1, *p*-anisidine and clicked with Azide-fluor 488)

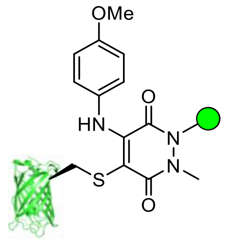

S33

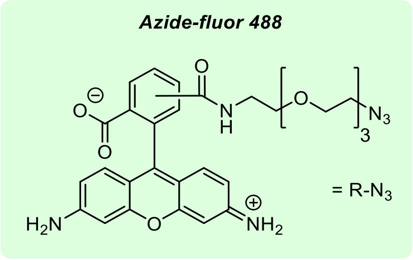

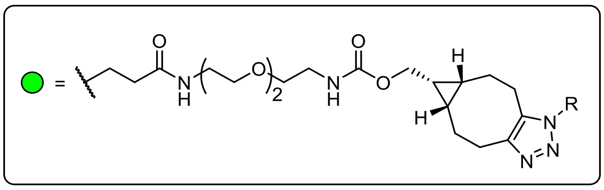

To a solution of GFP PD aniline conjugate **S32** (72 µL, 20 µM) in BBS (25 mM borate, 25 mM NaCl, 2 mM EDTA, 2% DMSO, pH 8) was added Azide-fluor 488 (0.72 µL, 10 mM in DMSO, 5 eq.) and the reaction incubated at 37 °C under constant agitation (300 rpm) for 16 h to give clicked GFP PD aniline conjugate **S33** (expected mass 30,542 Da, observed mass 30,540 Da). The product was analysed by LC-MS (method 1a*).

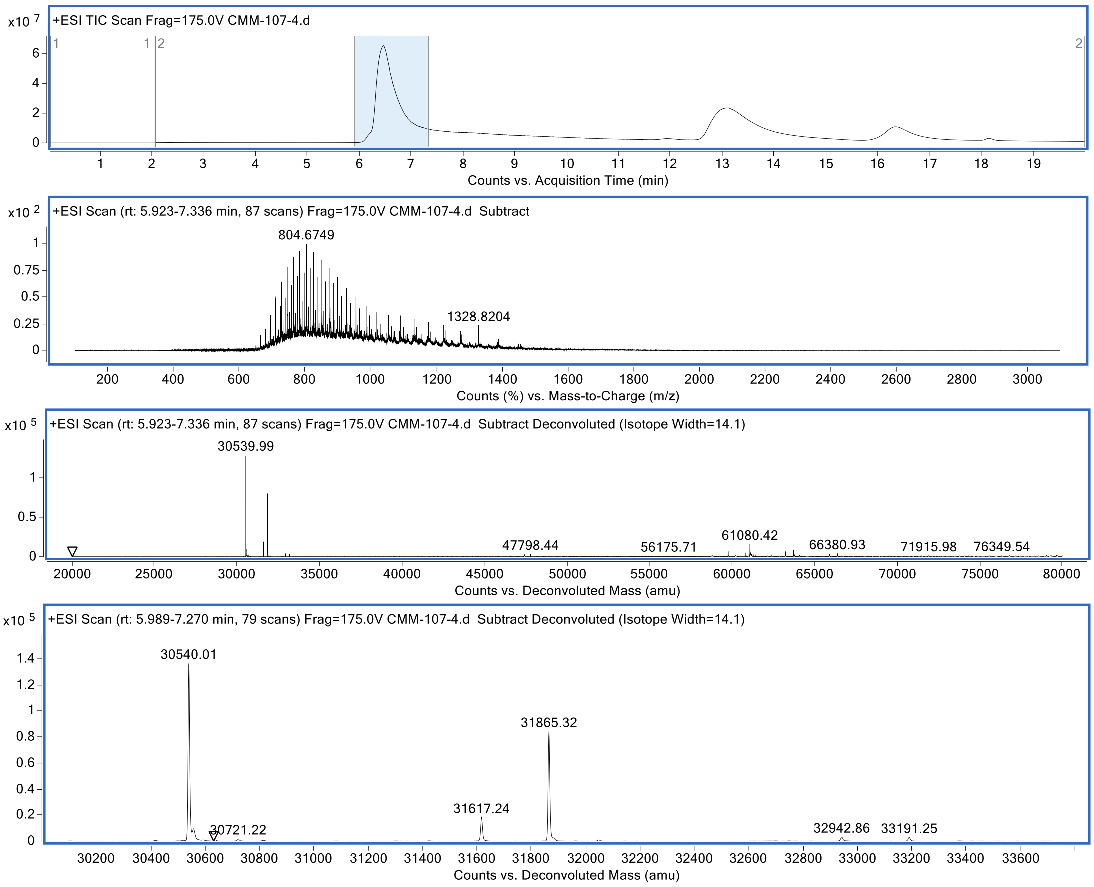

Figure S117: (i) TIC LC-MS trace (top), (ii) non-deconvoluted LC-MS trace (upper middle), (iii) deconvoluted MS data (lower middle, wide range), (iv) deconvoluted MS data (bottom, zoom in range).

Stability of GFP PD aniline conjugate S33 to TCEP

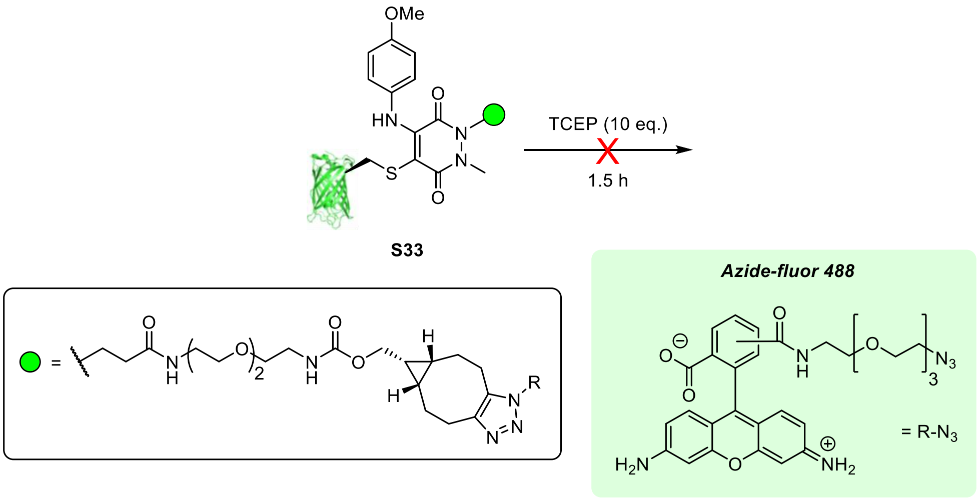

To a solution of **S33** (30 µL, 20 µM) in BBS (25 mM borate, 25 mM NaCl, 2 mM EDTA, 2% DMSO, pH 8.0) was added TCEP.HCl (0.3 µL, 20 mM in dH_2O, 10 eq.) and the reaction incubated at 37 °C for 1.5 h under constant agitation (300 rpm). No reaction was observed by LC-MS (method 1a*).

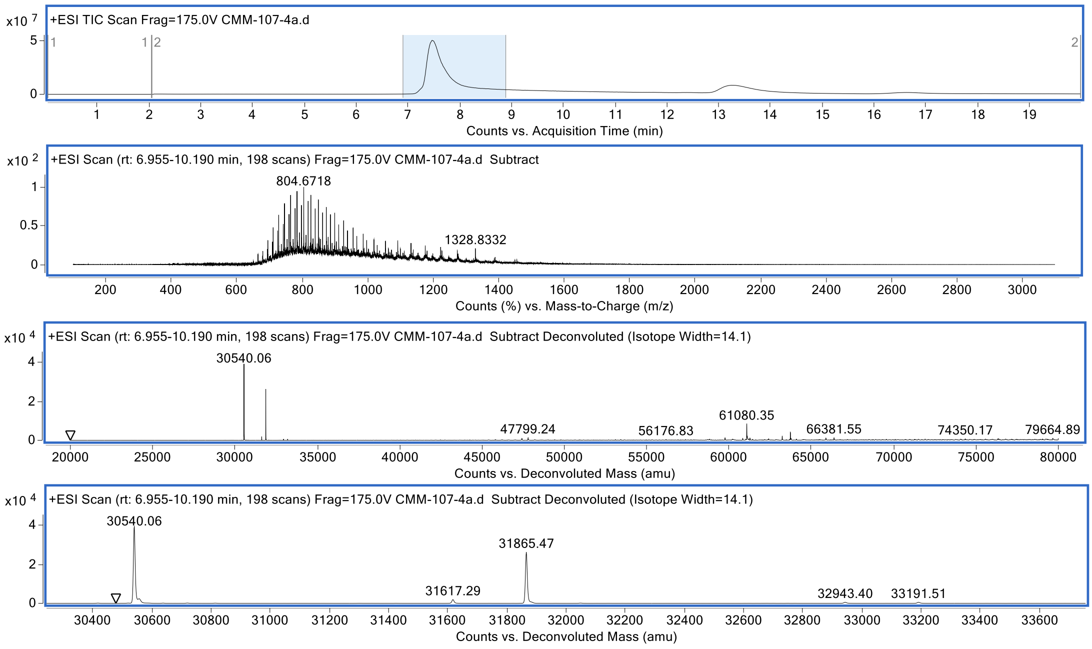

Figure S118: (i) TIC LC-MS trace (top), (ii) non-deconvoluted LC-MS trace (upper middle), (iii) deconvoluted MS data (lower middle, wide range), (iv) deconvoluted MS data (bottom, zoom in range).

LC S168C PD aniline fluorophore conjugate 30 (LC S168C thio-trastuzumab conjugated to BCN PD 1, clicked with Azide-fluor 488, reacted with N_3 Aniline 3)

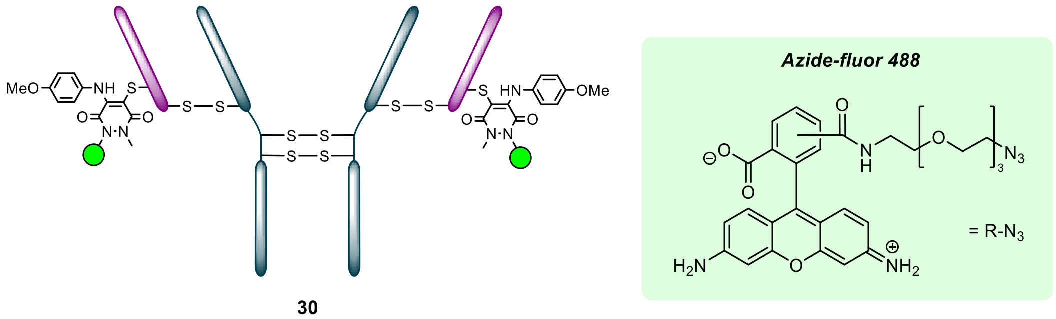

30

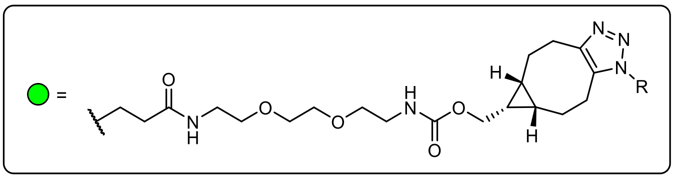

To a solution of conjugated LC S168C thio-trastuzumab **12** (25 µL, 13.2 µM) in BBS (25 mM borate, 25 mM NaCl, 2 mM EDTA, 2% DMSO, pH 8.0) was added *p*-anisidine (0.33 µL, 1 M in DMSO, 1000 eq.) and the reaction incubated at 37 °C for 16 h under constant agitation (300 rpm). After this time, excess reagents were removed to give conjugate **30** (expected mass 147,543 Da, observed mass 147,543 Da) which was analysed by LC-MS (method 1a), UV-Vis spectroscopy and SDS-PAGE.

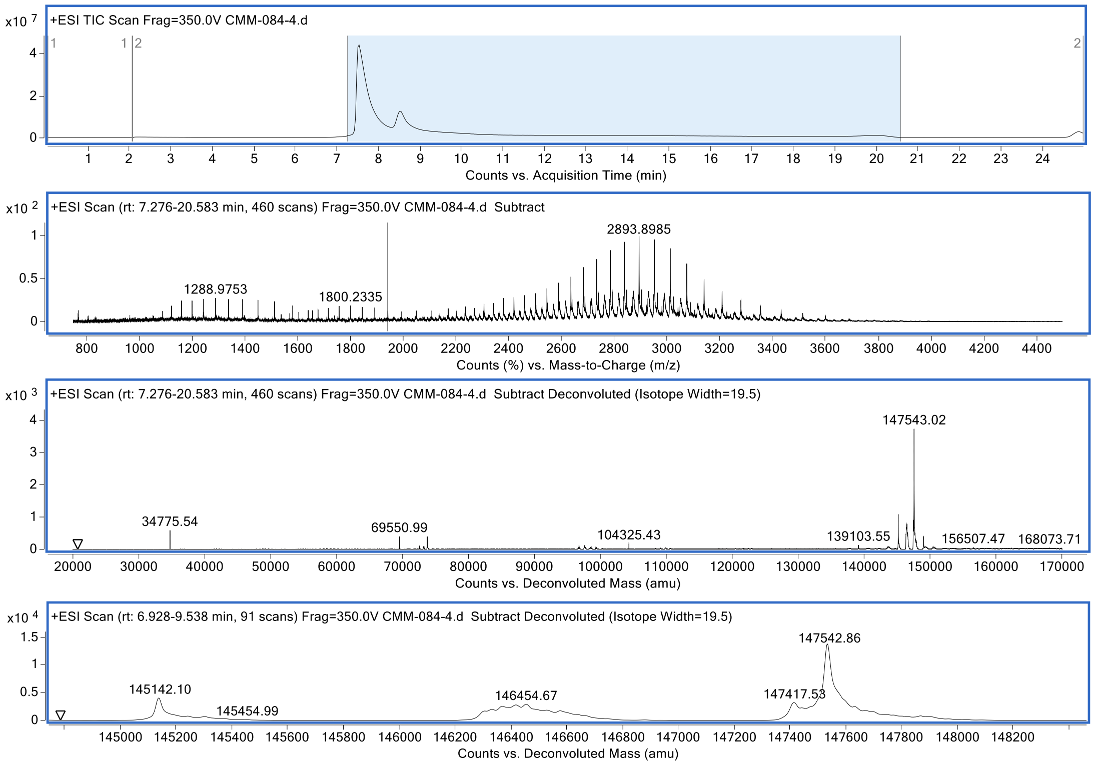

Figure S119: (i) TIC LC-MS trace (top), (ii) non-deconvoluted LC-MS trace (upper middle), (iii) deconvoluted MS data (lower middle, wide range), (iv) deconvoluted MS data (bottom, zoom in range).

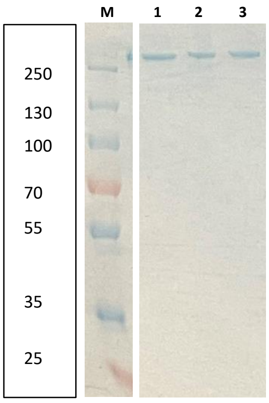

Figure S120: SDS-PAGE gel: M. Ladder, 1. Conjugate **9**, 2. Conjugate **12**, 3. Conjugate **30**.

LC S168C PD aniline conjugate 31 (LC S168C Thio-trastuzumab conjugated to BCN PD 1, Azide-fluor 488, and *p*-Anisidine, re-bridged with Mepstra PD 1)

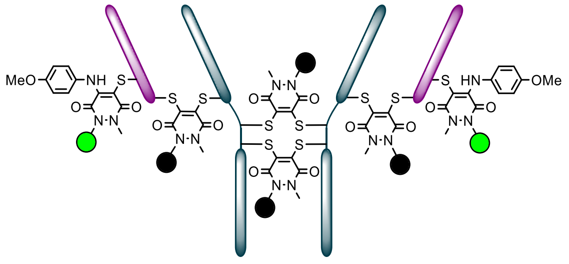

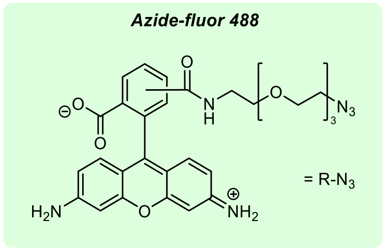

31

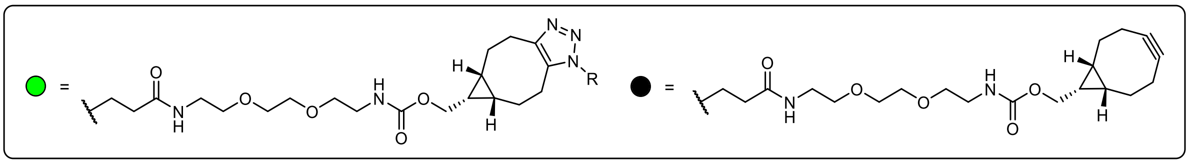

To a solution of LC S378C conjugate **30** (40 µL, 20 µM) was added TCEP.HCl (0.4 µL, 20 mM in dH_2O, 10 eq.) and the reaction incubated at 37 °C under constant agitation (300 rpm) for 1.5 h. After this time, excess TCEP was removed and BCN PD **1** added (0.4 µL, 20 mM in DMSO, 10 eq.) and the reaction incubated at 37 °C under constant agitation (300 rpm) for 3 h. After this time, excess reagents were removed to give conjugate **31** (expected mass 149,555 Da, observed mass 149,557 Da) which was analysed by LC-MS (method 1a), UV-Vis spectroscopy and SDS-PAGE.

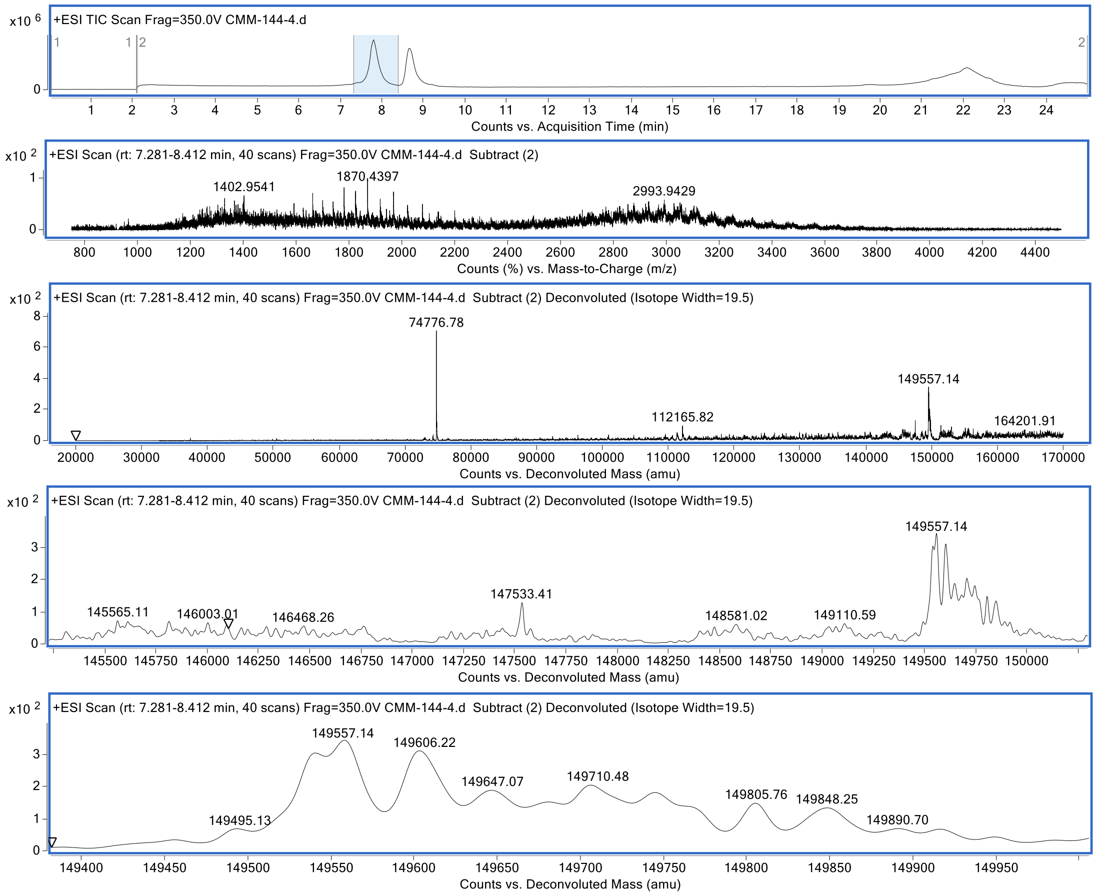

Figure S121: (i) TIC LC-MS trace (top), (ii) non-deconvoluted LC-MS trace (upper middle), (iii) deconvoluted MS data (lower middle, wide range), (iv) and (v) deconvoluted MS data (bottom, zoom in range). *Note*: the TIC peak on the right is PNGase, but as the sample is dilute it has not been deconvoluted as otherwise it dominates the mass spec.

LC S168C PD aniline fluorophore conjugate 32 (LC S168C Thio-trastuzumab conjugated to Mepstra PD 1, Azide-fluor 488, and *p*-Anisidine, re-bridged with Mepstra PD 1, clicked with Azide-fluor 488)

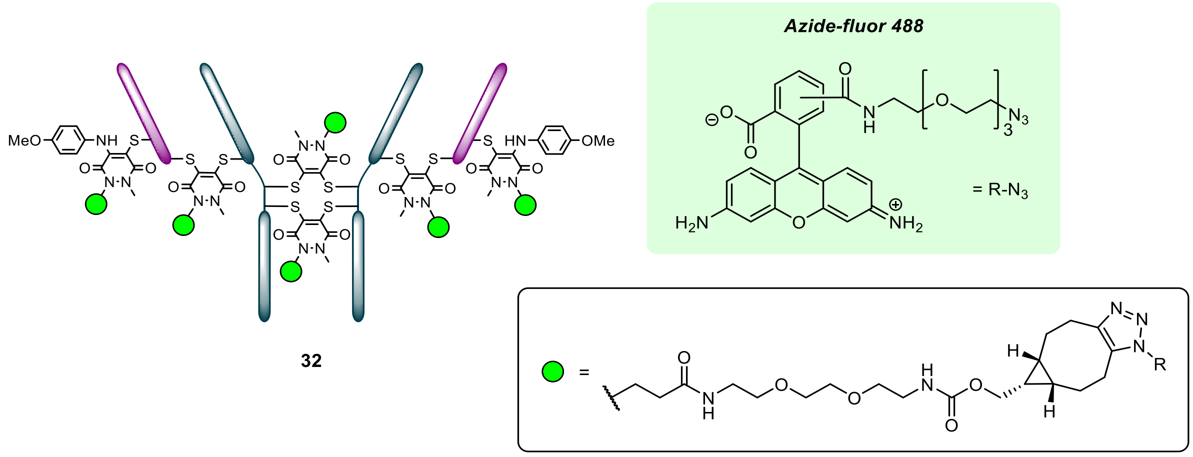

32

To a solution of LC S168C conjugate **31** (30 µL, 20 µM) was added Azide-fluor 488 (0.6 µL, 10 mM in DMSO, 10 eq.) and the reaction incubated at 37 °C under constant agitation (300 rpm) for 16 h. After this time, excess reagents were removed to give conjugate **32** (expected mass 151,855 Da, observed mass 151,852 Da) which was analysed by LC-MS (method 1b), UV-Vis spectroscopy and SDS-PAGE.

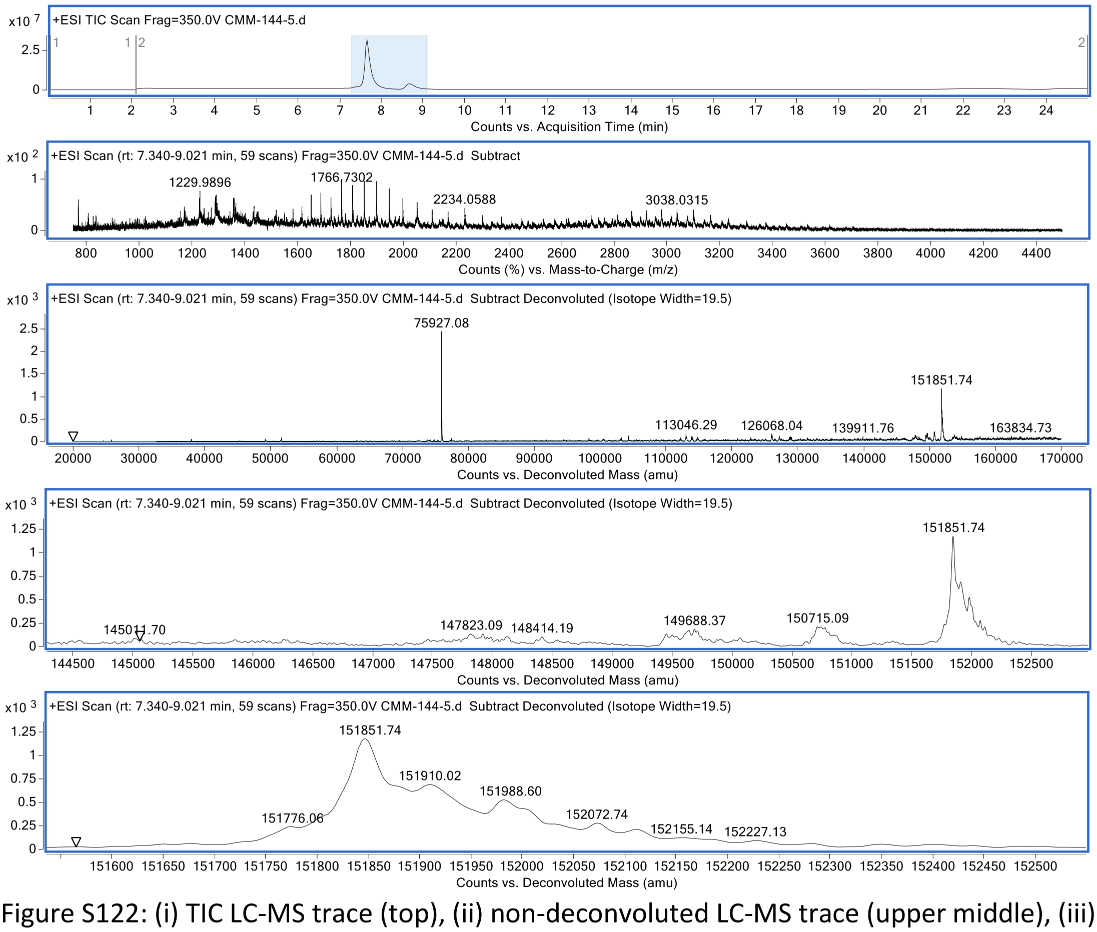

Figure S122: (i) TIC LC-MS trace (top), (ii) non-deconvoluted LC-MS trace (upper middle), (iii) deconvoluted MS data (lower middle, wide range), (iv) and (v) deconvoluted MS data (bottom, zoom in range).

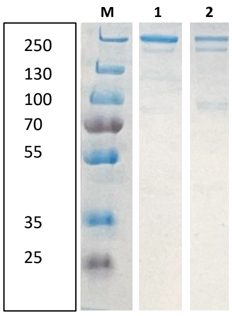

Figure S123: SDS-PAGE gel: M. Ladder, 1. Conjugate **31**, 2. Conjugate **32**.

LC S168C PD aniline fluorophore conjugate 33 (LC S168C Thio-Trastuzumab Conjugated to BCN PD 1, Azide-fluor 488, N_3 Aniline 3 and BP Fluor 568, Re-bridged with ArN_3 bisPD 2)

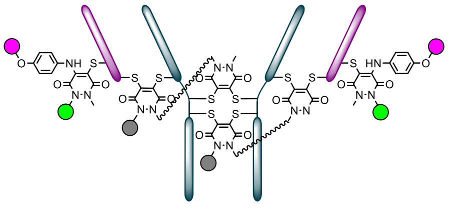

33

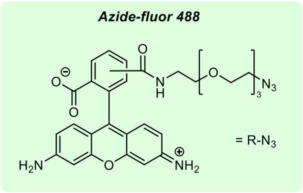

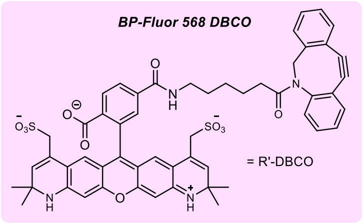

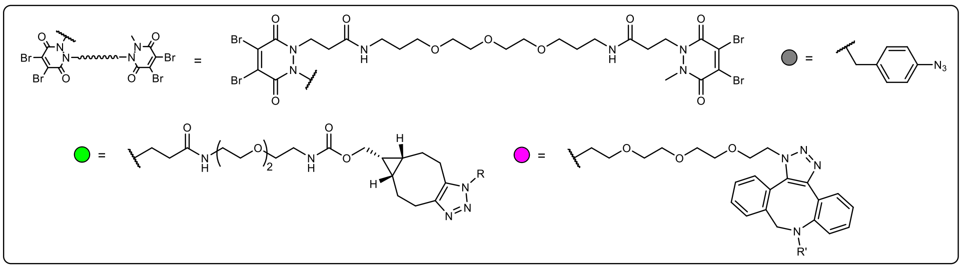

To a solution of conjugated LC S168C thio-trastuzumab **15** (107 µL, 15.8 µM) in BBS (25 mM borate, 25 mM NaCl, 2 mM EDTA, 2% DMSO, pH 8.0) was added TCEP.HCl (0.85 µL, 20 mM in dH_2O, 10 eq.) and the reaction incubated at 37 °C for 1.5 h under constant agitation (300 rpm). After this time, excess TCEP was removed and ArN_3 bisPD **2** (0.85 µL, 20 mM in dH_2O, 10 eq.) was added. The reaction was incubated at 37 °C for 3 h under constant agitation (300 rpm). After this time, excess PD was removed to give conjugate **33** (expected mass 151,211 Da, observed mass 151,220 Da) which was analysed by LC-MS (method 1b), UV-Vis spectroscopy and SDS-PAGE.

*Minor peaks are visible on LC-MS, these are likely to be minor amounts of the fully disulfide re-bridged, 1 mutant cysteine conjugated species (Expected Mass = 148,871 Da) and the fully disulfide re-bridged, no mutant cysteines conjugated species (Expected Mass = 146,530 Da).

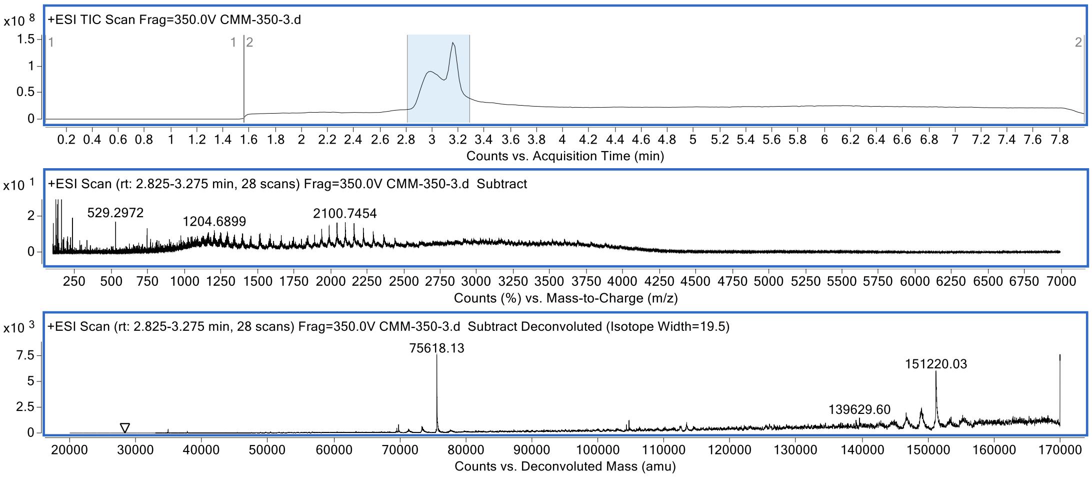

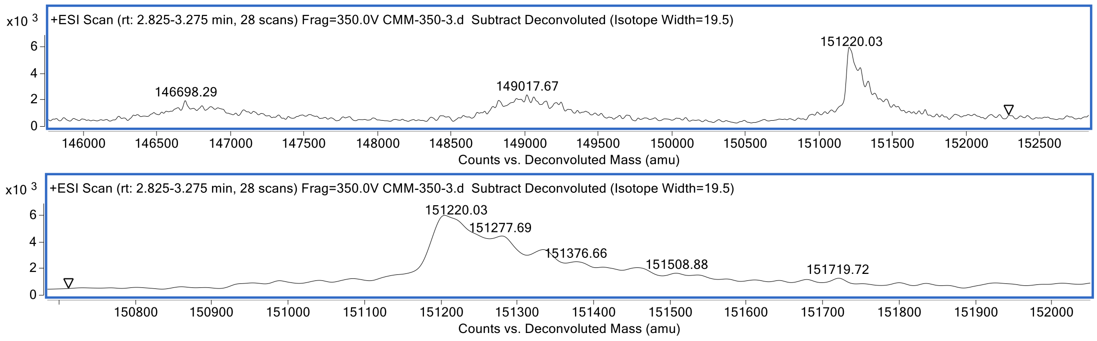

Figure S124: (i) TIC LC-MS trace (top), (ii) non-deconvoluted LC-MS trace (upper middle), (iii) deconvoluted MS data (lower middle, wide range), (iv)and (v) deconvoluted MS data (bottom, zoom in range).

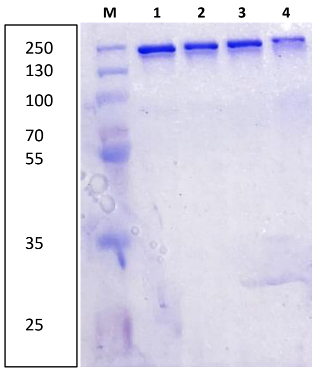

Figure S125: SDS-PAGE gel: M. Ladder, 1. Conjugate **12**, 2. Conjugate **14,** 3. Conjugate **15**, 4. Conjugate **33**.

LC S168C PD aniline fluorophore conjugate 34 (LC S168C thio-trastuzumab conjugated to BCN PD 1, Azide-fluor 488, N_3 Aniline 3 and BP Fluor 568, re-bridged with ArN_3 bisPD 2, clicked with BP Fluor 647)

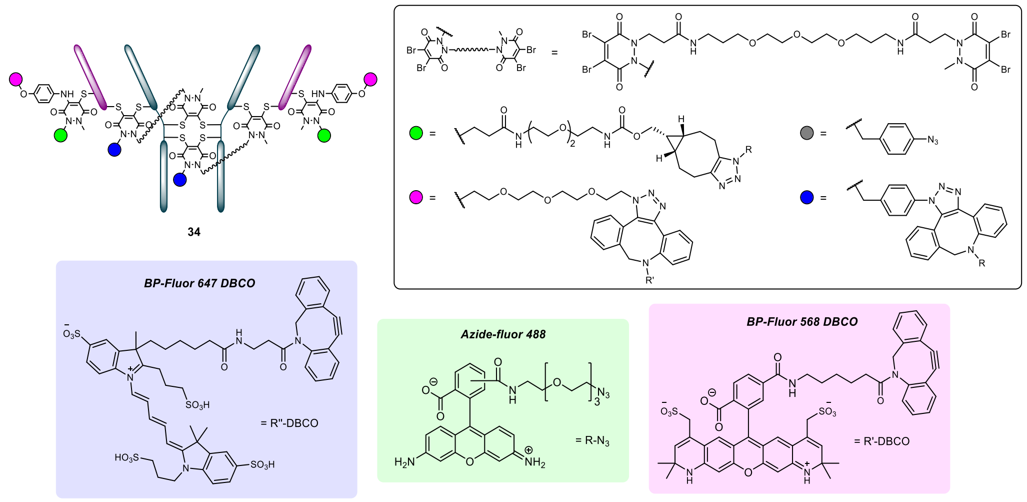

To a solution of conjugated LC S168C thio-trastuzumab **33** (100 µL, 7.4 µM) in BBS (25 mM borate, 25 mM NaCl, 2 mM EDTA, 2% DMSO, pH 8.0) was added BP Fluor 647 DBCO (1.5 µL, 20 mM in DMSO, 40 eq.) and the reaction incubated at 37 °C for 16 h under constant agitation (300 rpm). After this time, excess reagents were removed to give conjugate **34** (expected mass 153,474 Da, observed mass 153,450 Da) which was analysed by LC-MS (method 1b), UV-Vis spectroscopy and SDS-PAGE.

*A minor peaks is visible on LC-MS, this are likely to be minor amounts of the fully disulfide re-bridged, 1 mutant cysteine conjugated species (Expected Mass = 151,134 Da). PNGase is also visible in the LC-MS, from deglycosylation.

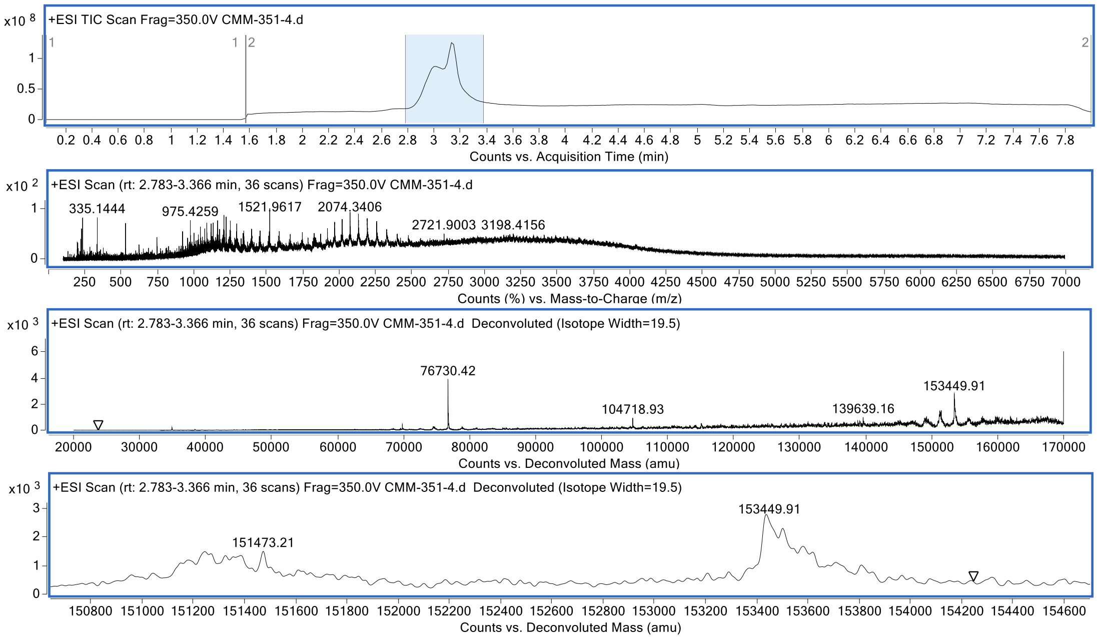

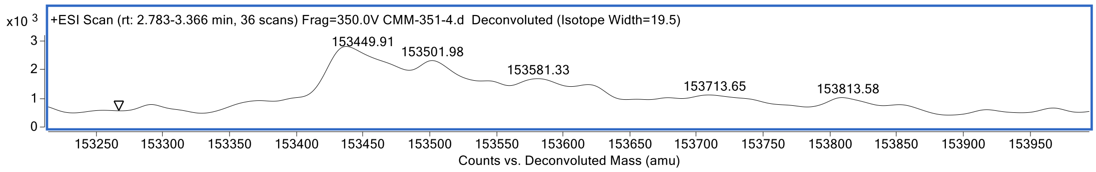

Figure S126: (i) TIC LC-MS trace (top), (ii) non-deconvoluted LC-MS trace (upper middle), (iii) deconvoluted MS data (lower middle, wide range), (iv) deconvoluted MS data (bottom, zoom in range).

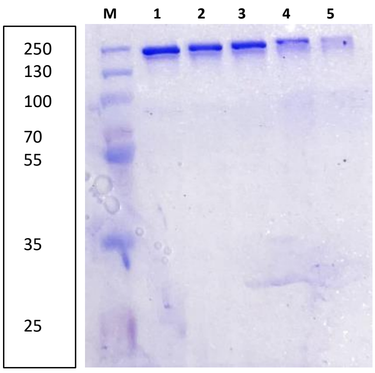

Figure S127: SDS-PAGE gel: M. Ladder, 1. Conjugate **12**, 2. Conjugate **14,** 3. Conjugate **15**, 4. Conjugate **33**, 5. Conjugate **34**.

HC S378C PD aniline fluorophore conjugate 35 (HC S378C thio-trastuzumab conjugated to ArN_3 bisPD 2, DBCO-biotin, N_3 Aniline 3 and BP Fluor 568 DBCO, re-bridged with ArN_3 bisPD 2)

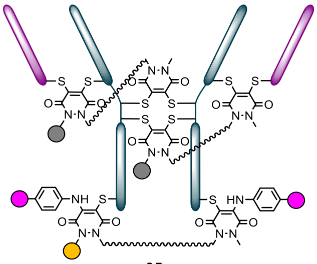

35

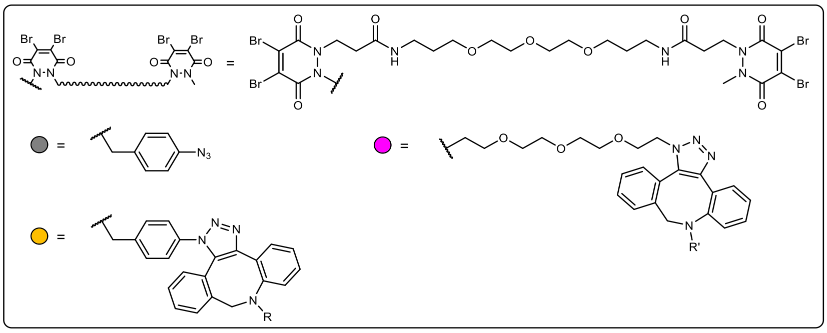

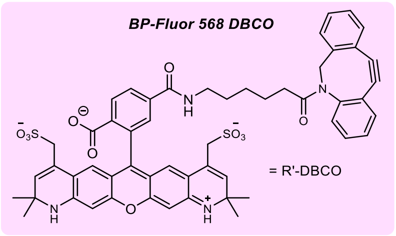

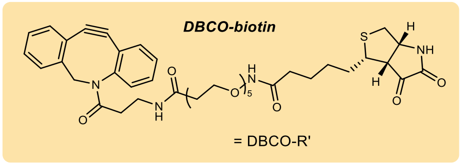

To a solution of HC S378C conjugate **20** (95 µL, 9.4 µM) was added TCEP.HCl (0.45 µL, 20 mM in dH_2O, 10 eq.) and the reaction incubated at 37 °C under constant agitation (300 rpm) for 1.5 h. After this time, excess TCEP was removed and ArN_3 bisPD **2** (0.45 µL, 20 mM in MeCN, 8 eq.) was added. The reaction was incubated at 37 °C under constant agitation (300 rpm) for 3 h. After this time, excess reagents were removed to give conjugate **35** (expected mass 150,501 Da, observed mass 150,530 Da) which was analysed by LC-MS (method 1b), UV-Vis spectroscopy and SDS-PAGE.

*Minor peaks are visible on LC-MS, these do not match to any particular expected conjugate but could potentially be HC S378C reacted only with DBCO-biotin clicked ArN_3 bisPD **2** (Expected Mass = 146,756 Da).

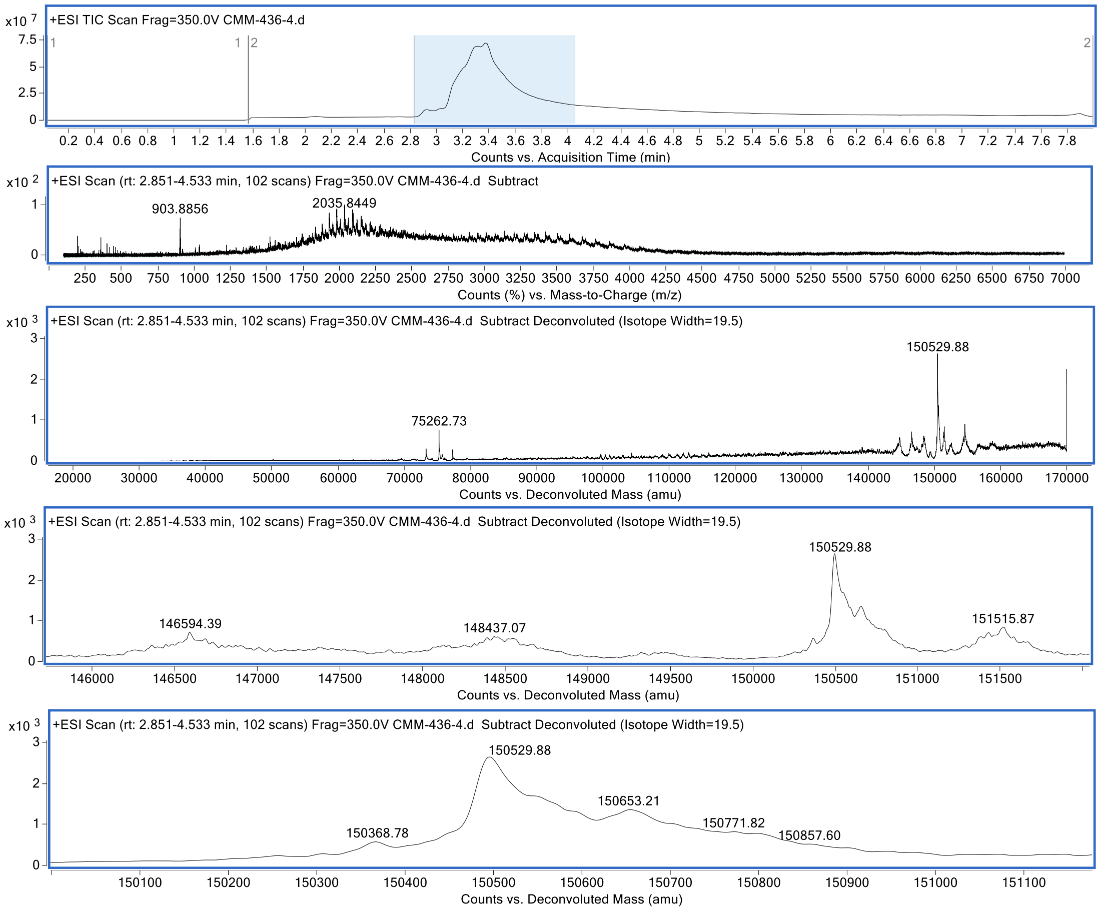

Figure S128: (i) TIC LC-MS trace (top), (ii) non-deconvoluted LC-MS trace (upper middle), (iii) deconvoluted MS data (lower middle, wide range), (iv) deconvoluted MS data (bottom, zoom in range).

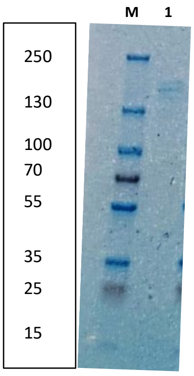

Figure S129: SDS-PAGE gel: M. Ladder, 1. Conjugate **35**.

HC S378C PD aniline fluorophore conjugate 36 (HC S378C thio-trastuzumab conjugated to ArN$_3$ bisPD 2, DBCO-biotin, N$_3$ Aniline 3 and BP Fluor 568 DBCO, re-bridged with ArN$_3$ bisPD 2, clicked with BP Fluor 647 DBCO)

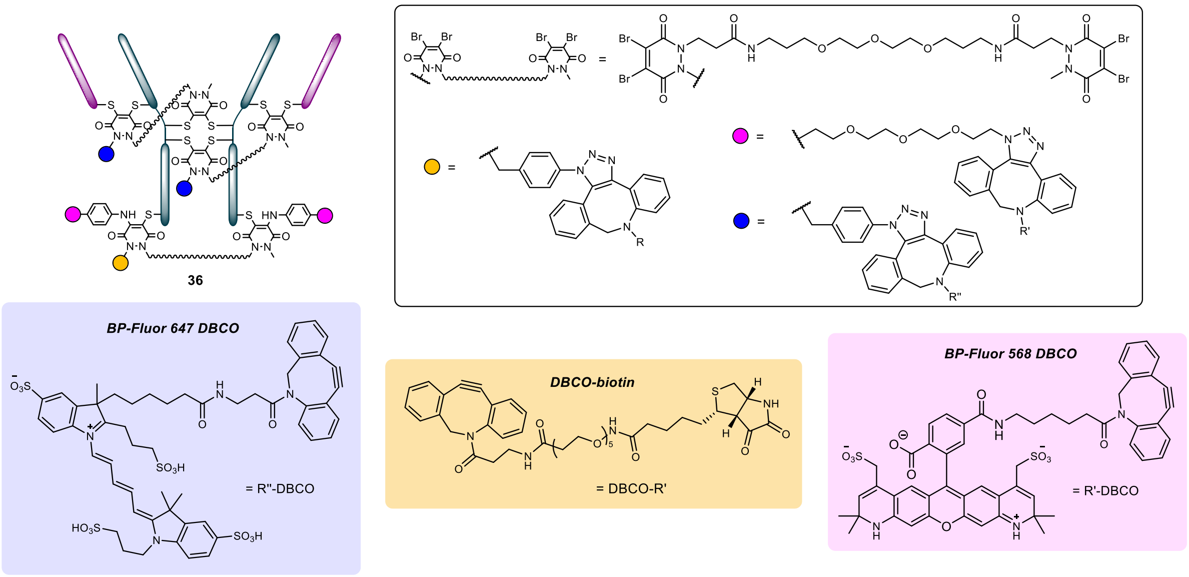

To a solution of HC S378C conjugate **35** (90 µL, 9.3 µM) was added BP Fluor 647 DBCO (1.7 µL, 20 mM in DMSO, 40 eq.) and the reaction incubated at 37 °C under constant agitation (300 rpm) for 16 h. After this time, excess reagents were removed to give conjugate **36** (expected mass 152,764 Da, observed mass 152,735 Da) which was analysed by LC-MS (method 1b), UV-Vis spectroscopy and SDS-PAGE.

*Minor peaks are visible on LC-MS, these do not match to any particular expected conjugate but could potentially be conjugate **35** re-bridged/clicked with BP Fluor 647 DBCO only once (Expected Mass = 150,939 Da) or not at all (Expected Mass = 149,114 Da). PNGase is also visible on the LC-MS.

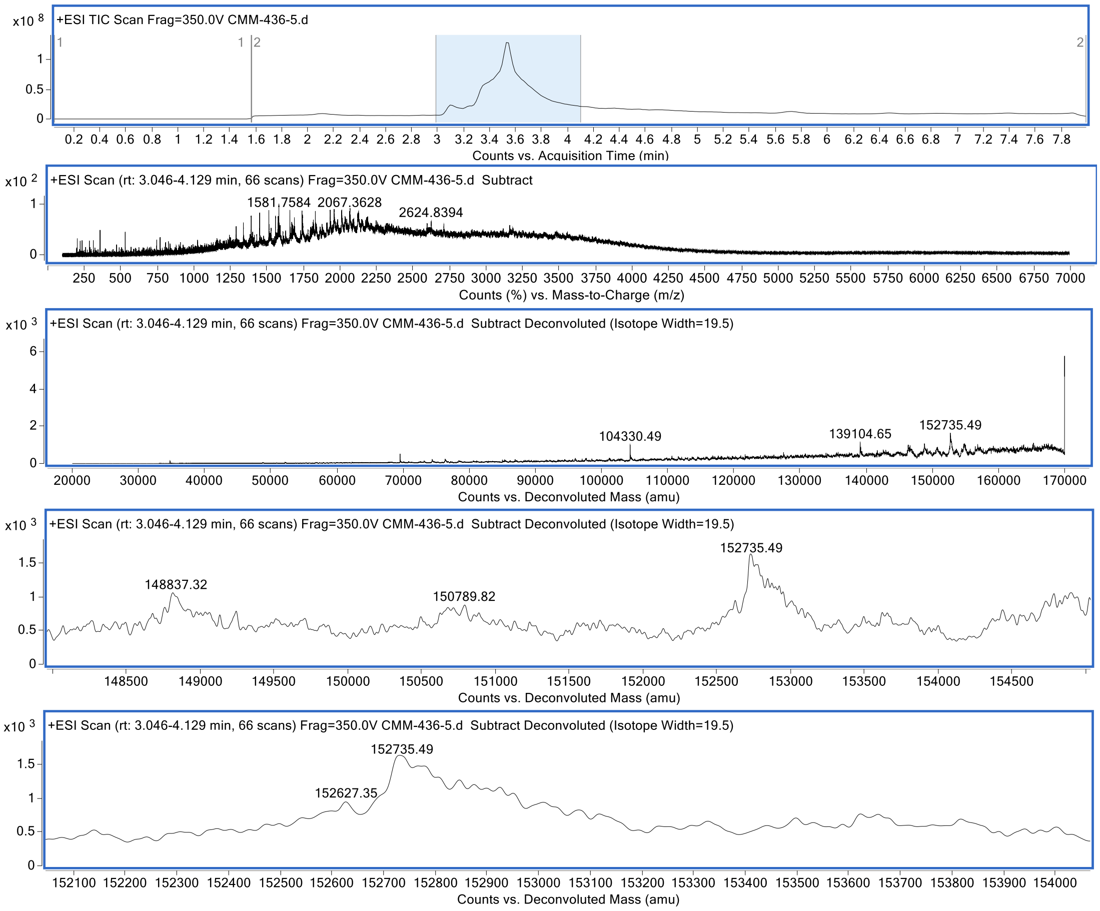

Figure S130: (i) TIC LC-MS trace (top), (ii) non-deconvoluted LC-MS trace (upper middle), (iii) deconvoluted MS data (lower middle, wide range), (iv) deconvoluted MS data (bottom, zoom in range).

HC S378C PD aniline fluorophore conjugate 37 (HC S378C thio-trastuzumab conjugated to ArN_3 bisPD 2, BP Fluor 568, N_3 Aniline 3 and BP Fluor 647 DBCO, re-bridged with BCN PD 1)

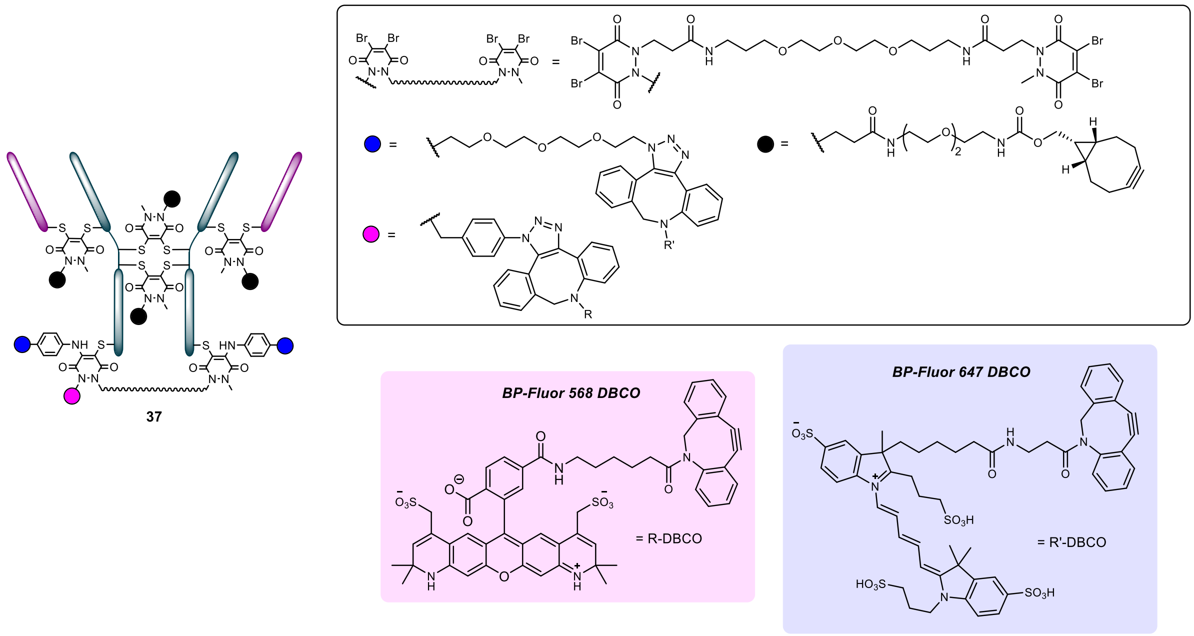

To a solution of HC S378C conjugate **19** (170 µL, 14.3 µM) was added TCEP.HCl (1.22 µL, 20 mM in dH_2O, 10 eq.) and the reaction incubated at 37 °C under constant agitation (300 rpm) for 1.5 h. After this time, excess TCEP was removed and Mepstra PD **1** added (1.22 µL, 20 mM in DMSO, 10 eq.) and the reaction incubated at 37 °C under constant agitation (300 rpm) for 3 h. After this time, excess reagents were removed to give conjugate **37** (expected mass 151,682 Da, observed mass 151,666 Da) which was analysed by LC-MS (method 1b), UV-Vis spectroscopy and SDS-PAGE.

*Minor peaks are visible on LC-MS, but these do not match to any expected extra unwanted addition and are expected to be due to incomplete deglycosylation.

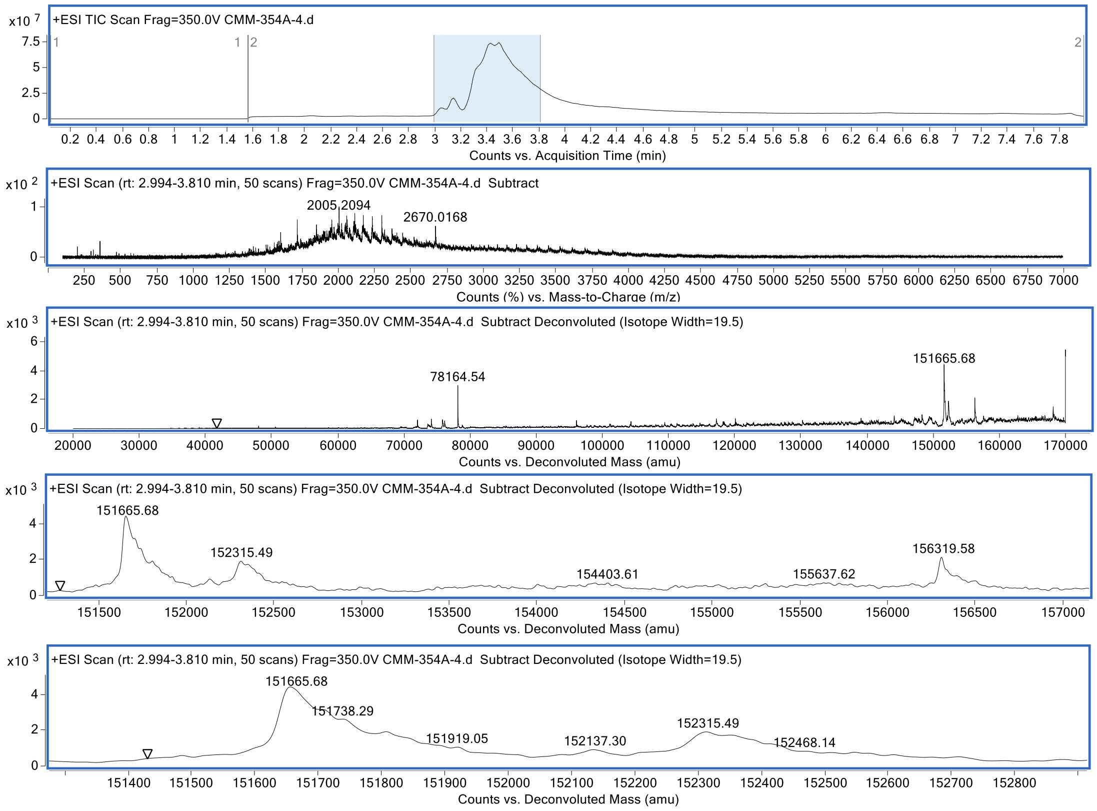

Figure S131: (i) TIC LC-MS trace (top), (ii) non-deconvoluted LC-MS trace (upper middle), (iii) deconvoluted MS data (lower middle, wide range), (iv) and (v) deconvoluted MS data (bottom, zoom in range). *Some incomplete deglycosylation observed.

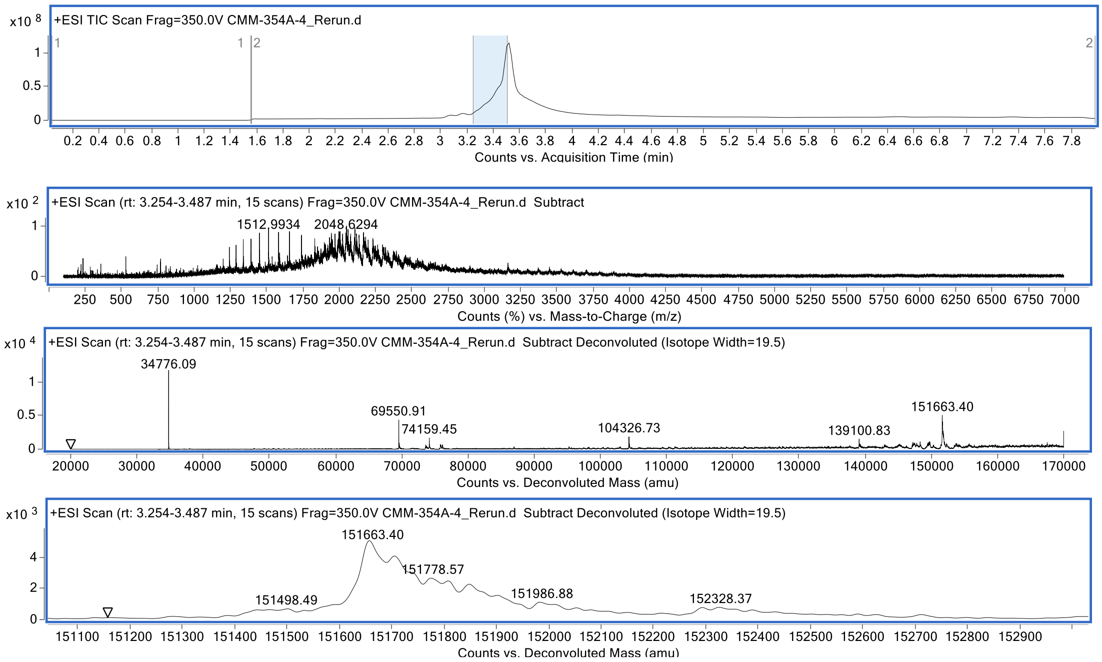

Figure S132: (i) TIC LC-MS trace (top), (ii) non-deconvoluted LC-MS trace (upper middle), (iii) deconvoluted MS data (lower middle, wide range), (iv) deconvoluted MS data (bottom, zoom in range).

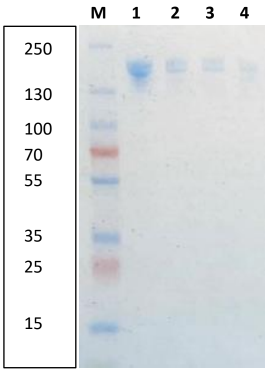

Figure S133: SDS-PAGE gel: M. Ladder, 1. Conjugate **17**, 2. Conjugate **18**, 3. Conjugate **19**, 4. Conjugate **37**.

HC S378C PD aniline fluorophore conjugate 38 (HC S378C thio-trastuzumab conjugated to ArN₃ bisPD 2, BP Fluor 578, N₃ Aniline 3 and BP Fluor 647, re-bridged with BCN PD 1, Clicked with Azide-Fluor 488)

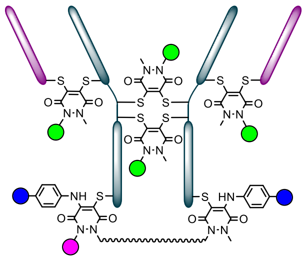

38

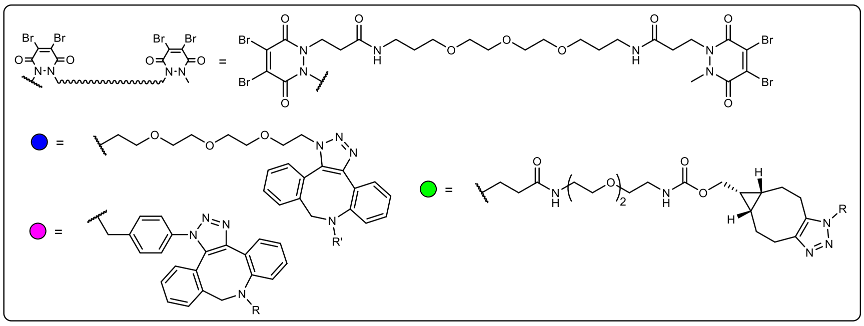

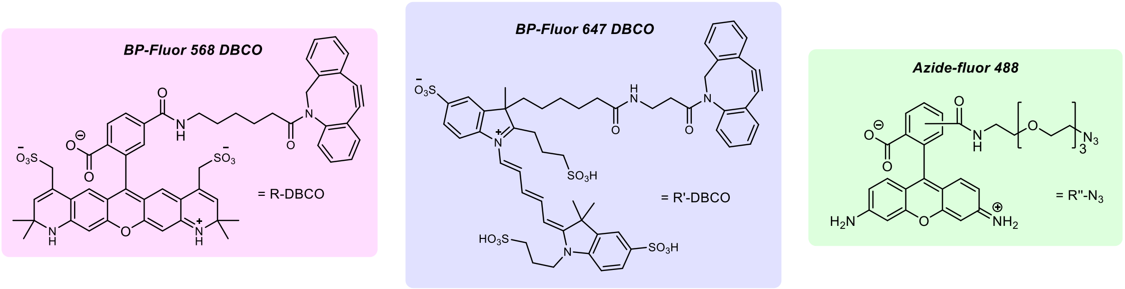

To a solution of HC S378C conjugate **37** (135 µL, 12.1 µM) was added Azide-Fluor 488 (1.63 µL, 10 mM in DMSO, 10 eq.) and the reaction incubated at 37 °C under constant agitation (300 rpm) for 20 h. After this time, excess reagents were removed to give conjugate **38** (expected mass 153,980 Da, observed mass 153,954 Da) which was analysed by LC-MS (method 1b), UV-Vis spectroscopy and SDS-PAGE.

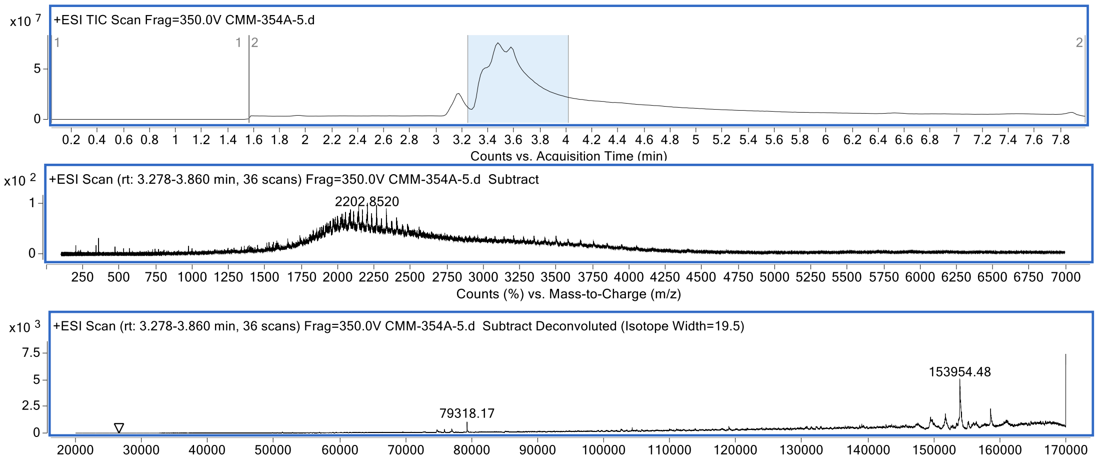

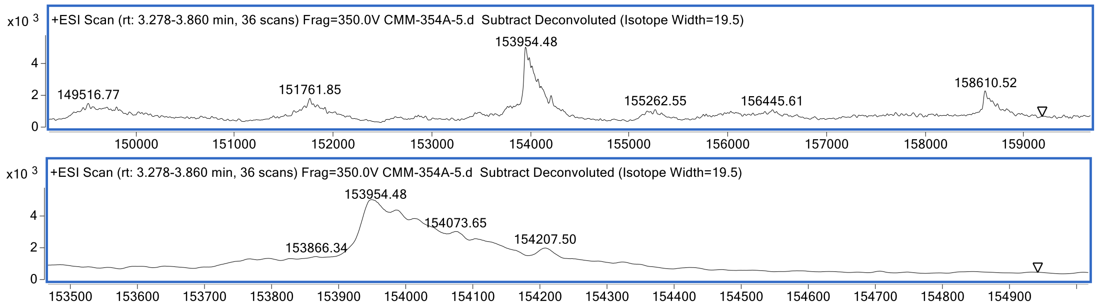

Figure S134: (i) TIC LC-MS trace (top), (ii) non-deconvoluted LC-MS trace (upper middle), (iii) deconvoluted MS data (lower middle, wide range), (iv) deconvoluted MS data (bottom, zoom in range).

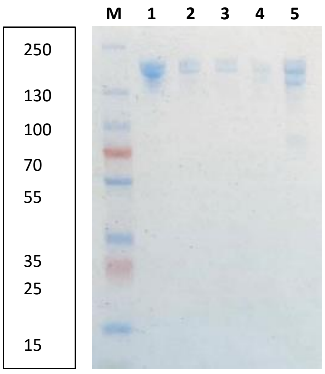

Figure S135: SDS-PAGE gel: M. Ladder, 1. Conjugate **17**, 2. Conjugate **18**, 3. Conjugate **19**, 4. Conjugate **37**, 5. Conjugate **38**.

LC S168C PD aniline conjugate S34 (LC S168C thio-trastuzumab one-pot reaction with BCN PD 1, reacted with Azide-fluor 488 and N_3 Aniline 3)

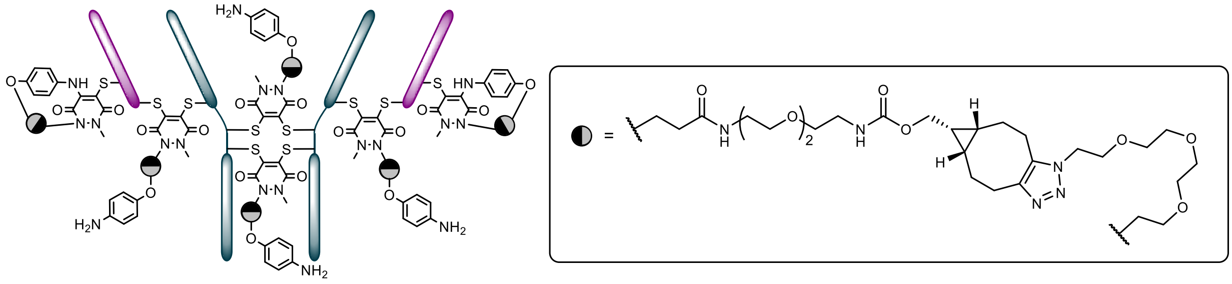

S34

To a solution of conjugated LC S168C thio-trastuzumab **26** (100 µL, 20 µM) in BBS (25 mM borate, 25 mM NaCl, 2 mM EDTA, 2% DMSO, pH 8.0) was added N_3 aniline **3** (1 µL, 1 M in DMSO, 1000 eq.) and the reaction incubated at 37 °C for 16 h under constant agitation (300 rpm). After this, excess reagents were removed to give conjugate **S34** (expected mass 150,019 Da, observed mass 150,024 Da) which was analysed by LC-MS (method 1b), UV-Vis spectroscopy and SDS-PAGE.

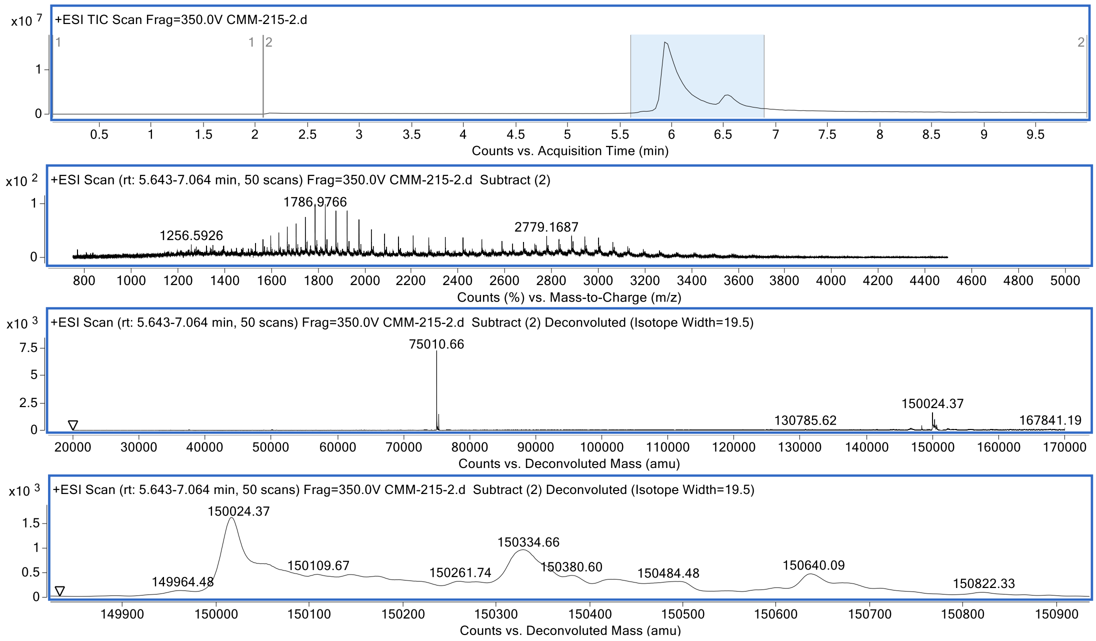

Figure S136: (i) TIC LC-MS trace (top), (ii) non-deconvoluted LC-MS trace (upper middle), (iii) deconvoluted MS data (lower middle, wide range), (iv) deconvoluted MS data (bottom, zoom in range).

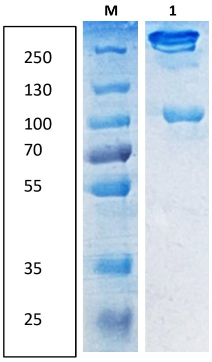

Figure S137: SDS-PAGE gel: M. Ladder, 1. Conjugate **S34**.

Enzyme-Linked Immunosorbent Assay (ELISA)

A 96-well plate was coated for 1 h at RT with HER2 (Sino Biological, 100 µL/well, 0.25 µg/mL solution in PBS). After washing (3 × 0.1% Tween® 20 in PBS, followed by 3 × PBS), the wells were blocked for 1 h at RT with 5% Marvel milk powder (Premier foods) in PBS (200 µL/well). The wells were then washed and the following dilutions of unmodified antibody and conjugate 10 (made using reagents 4, 7 and 8 respectively) were applied: 270 nM, 90 nM, 30 nM, 10 nM, 3.33 nM, 1.11 nM, 0.37 nM, 0.123 nM, 0.0412 nM, 0.0137 nM, prepared in 1% Marvel solution in 0.1% Tween® 20 in PBS (100 µL/well). The assay was then incubated at RT for 1 h, washed and the detection antibody (AntiHuman IgG, Fab specific-HRP antibody, Sigma Aldrich, 1:5000 in 1% Marvel solution in 0.1% Tween® 20 in PBS) was added (100 µL/well). After 1 h at RT, the plates were washed and *o*-phenylenediamine dihydrochloride (Sigma-Aldrich, 100 µL/well, 0.5 mg/mL in a phosphate-citrate buffer with sodium perborate) was added. Once a yellow-orange colour was observed, the reaction was stopped by addition of HCl (4M, 50 µL/well). Absorbance was immediately measured at 450 nm and was corrected by subtracting the average of negative controls (PBS only instead of HER2/conjugate). Each sample was tested in triplicate and errors are shown as the standard deviation of the average. ELISA data were analysed with Graphpad Prism 10.1.24 (including K_D calculations) and the values have been normalised.

ELISA for HC S378C Conjugate 16

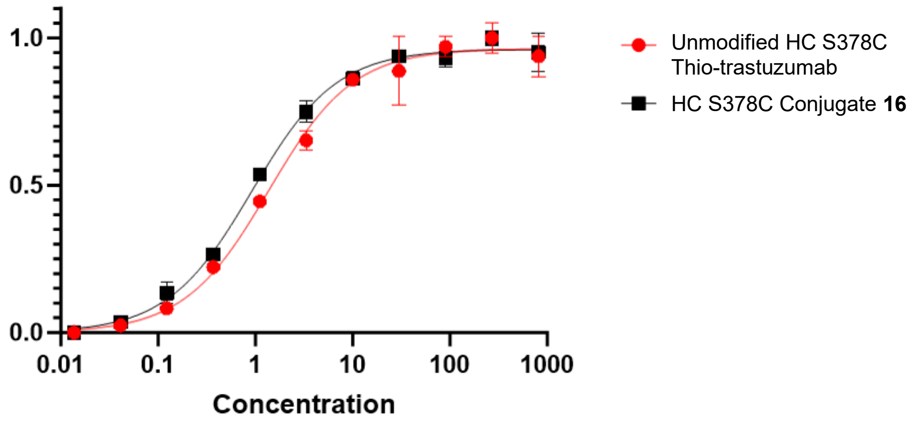

	1	2	3	4	5	6	7	8	9	10	11	12
A	1.957	2.311	2.215	2.108	1.934	1.418	1.035	0.534	0.278	0.138	0.094	0.044
B	2.125	2.314	2.235	2.186	1.983	1.541	1.072	0.614	0.251	0.169	0.106	0.09
C	2.254	2.115	2.1	1.724	1.916	1.551	1.071	0.6	0.318	0.168	0.121	0.087
D	0.071	0.053	0.078	0.072	0.073	0.114	0.071	0.11	0.08	0.096	0.086	0.062
E	2.194	2.132	2.075	2.072	1.914	1.728	1.219	0.631	0.338	0.167	0.108	0.056
F	1.923	2.143	1.966	2.029	1.837	1.581	1.181	0.669	0.35	0.178	0.097	0.052
G	2.084	2.229	2.027	2.022	1.906	1.649	1.237	0.66	0.471	0.2	0.123	0.101
H	0.079	0.086	0.07	0.064	0.059	0.068	0.098	0.098	0.083	0.067	0.07	0.061

Figure S138: Top: Normalised ELISA for conjugate **16**. Bottom: Raw data – readout from each well.

K_D Unmodified Ab = 1.37 nM

K_D Conjugate **16** = 0.92 nM

ELISA for LC S168C Conjugate S34

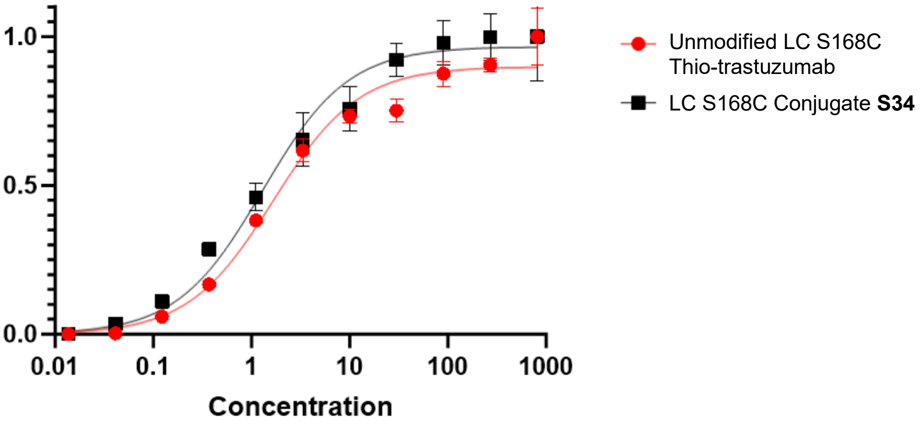

	1	2	3	4	5	6	7	8	9	10	11	12
A	2.001	1.691	1.637	1.463	1.445	1.259	0.765	0.421	0.235	0.132	0.157	0.115
B	1.905	1.738	1.721	1.474	1.387	1.211	0.814	0.406	0.252	0.141	0.138	0.078
C	1.673	1.653	1.575	1.355	1.364	1.128	0.8	0.438	0.22	0.143	0.106	0.127
D	0.055	0.051	0.079	0.071	0.183	0.098	0.107	0.092	0.082	0.081	0.045	0.074
E	1.539	1.578	1.568	1.564	1.246	1.012	0.748	0.574	0.257	0.143	0.11	0.05
F	1.563	1.655	1.606	1.47	1.22	1.101	0.892	0.525	0.253	0.157	0.082	0.065
G	1.958	1.818	1.788	1.646	1.437	1.292	0.841	0.551	0.304	0.158	0.094	0.043
H	0.088	0.101	0.088	0.085	0.11	0.119	0.071	0.126	0.074	0.072	0.051	0.066

Figure S139: Top: Normalised ELISA for conjugate **S34**. Bottom: Raw data – readout from each well.

K_D Unmodified Ab = 1.67 nM
K_D Conjugate **S34** = 1.30 nM

Cell Studies

The cells used in these assays are BT-474 (ATCC / HTB-20) expressing HER2 receptor and, as a negative control, the MCF-7 (ATCC / HTB-22) deficient for the expression of HER2 receptor. The BT-474 cells were cultured with Hybri-Care 46-X medium (ATCC) supplemented with 1.5g/L of sodium bicarbonate (Gibco / 25080-094) and 10% Foetal bovine serum (Gibco). The MCF-7 were cultured in MEM Sigma-Aldrich / M0325) supplemented with 0.01 mg/ml of recombinant human insulin (Merck / I9278) and 10% Foetal bovine serum (Gibco).

The BT-474 and MCF-7 cells were seeded into a µClear (Greiner Bio-One / 781090) 384 well plate at a density of 8,000 and 3,000 cell per well, respectively. The cells were then maintained at 37 °C in an atmosphere containing 5% CO_2 for four days.

15 minutes before adding the antibody, the cells were incubated at 4 °C. Then, the medium was aspirated and replaced by fresh and cold medium containing the conjugated antibodies. The plates were incubated for one hour at 4 °C and washed once with PBS to remove unbound immunoglobulins.

For live cell imaging:

PBS was replaced by the appropriate medium and further incubated for 30 minutes at 37 °C in an atmosphere containing 5% CO_2. Once warmed, the plate was placed in the microscope pre-set up with at constant temperature of 37 °C and a CO_2 level of 5%.

Imaging of fixed samples:

When the appropriate time was reached, the cells were fixed for 20 minutes with a 4 % formaldehyde-solution. The cells were washed three times with PBS and incubated with a solution of PBS-Hoechst 5 µg/ml for 30 minutes. The cells were washed another 3 times with PBS prior imaging.

Cell imaging and image analysis:

The images were acquired with either the automated microscopes Opera Phenix Plus or Operetta both equipped: with a x20 Air (NA 0.8), ×63 (NA 1.2) water lens and 405-, 488-, 561-, and 640-nm excitation lasers. The emitted fluorescence was captured using a set of cameras associated with a set of filters covering a detection wavelength ranging from 450 to 690 nm. For each well, 9 (for x20 lens) to 20 (for x63 lens) adjoining fields containing 4 to 5 Z-stacks separated by 1.5 µm were acquired. We applied a 10% overlap between fields to generate a global image clustering all the fields in a single image. The maximum projection of each field was analysed using a dedicated in-built script developed using the image analysis software Harmony 5.0 or 5.2 (PerkinElmer).

All data were plotted and analysed using Microsoft Excel 2010, GraphPad Prism 10, or Dibco Spotfire.

Initial Dose-Response Experiments

Initial dose response assays were performed with HC S378C-Azide Fluor 488 **11** (VH-AF488) and LC S168C-Azide Fluor 488 **12** (VL-AF488).

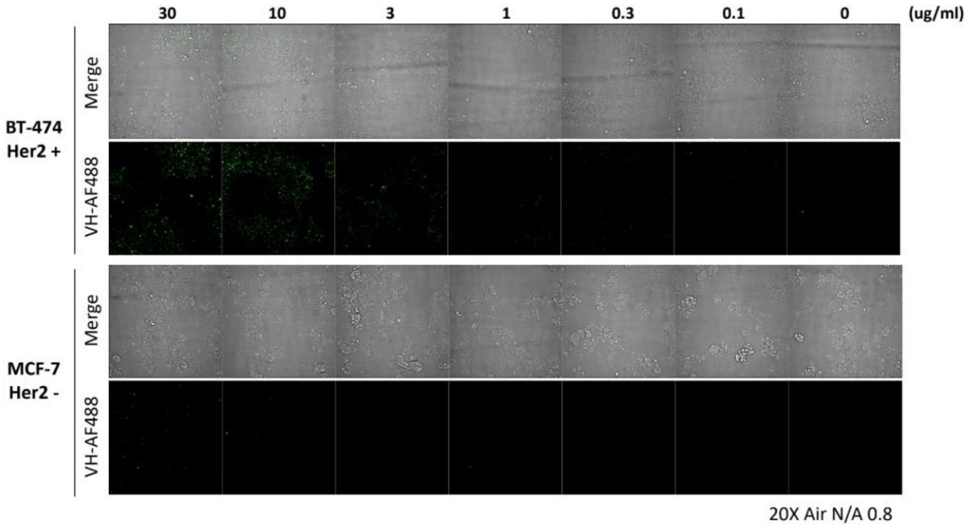

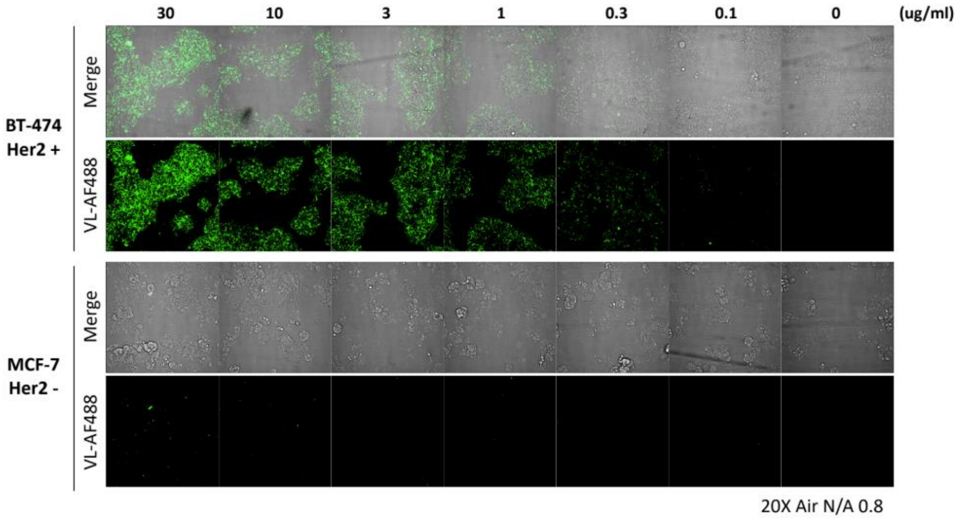

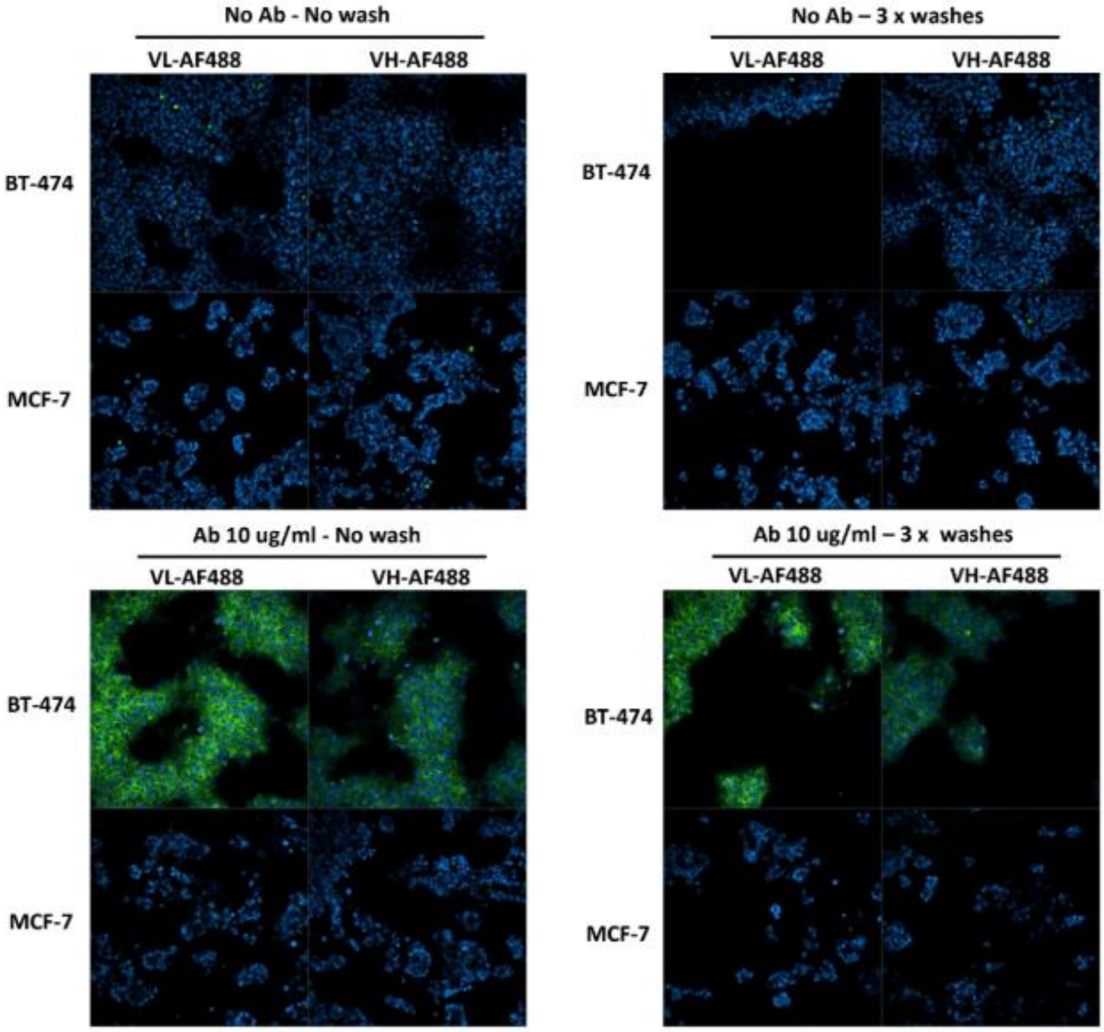

Figure S140: Overview of initial dose-response experiments with HC S378C-Azide Fluor 488 **11** (VH-AF488) and LC S168C-Azide Fluor 488 **12** (VL-AF488). Cells were fixed after 6 h.

Monitoring of antibody uptake overtime (Live cell)

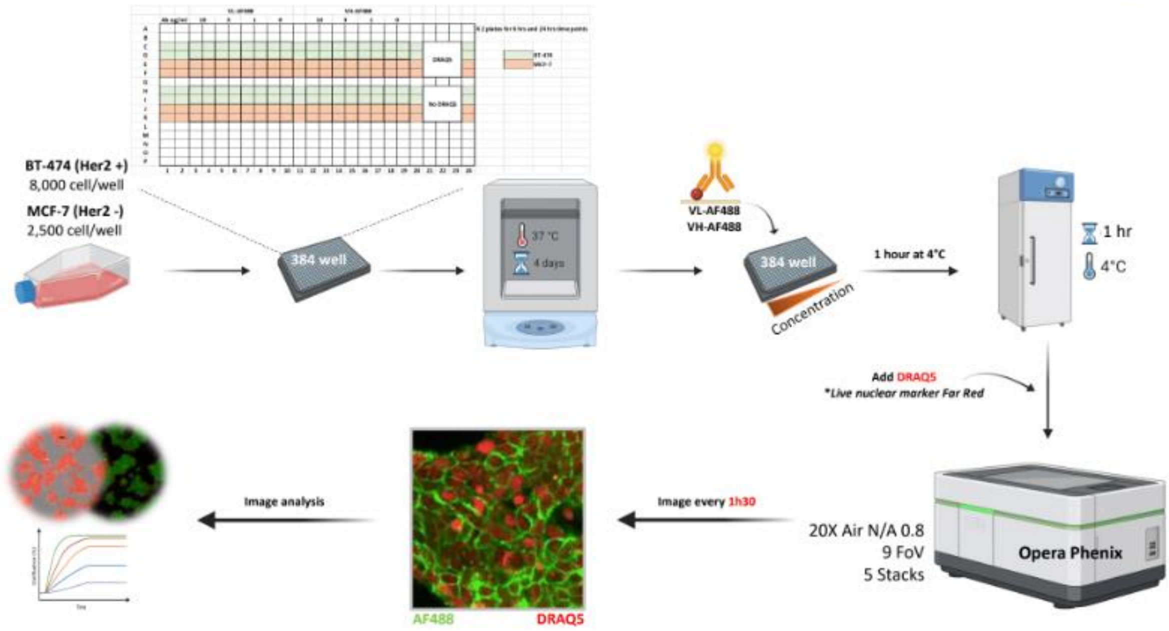

BT-474_VL-AF488

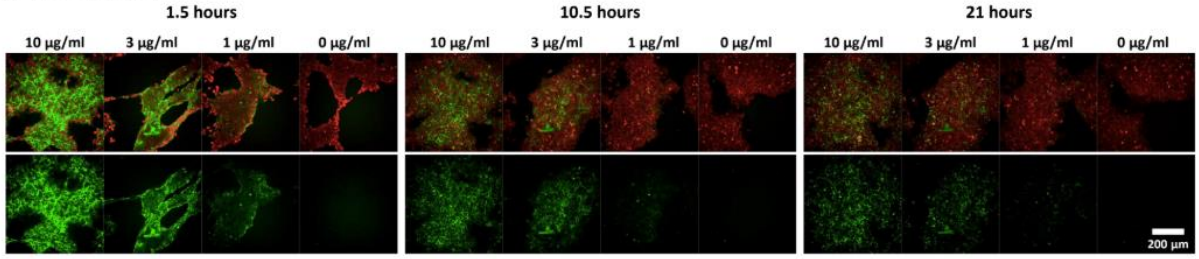

BT-474_VH-AF488

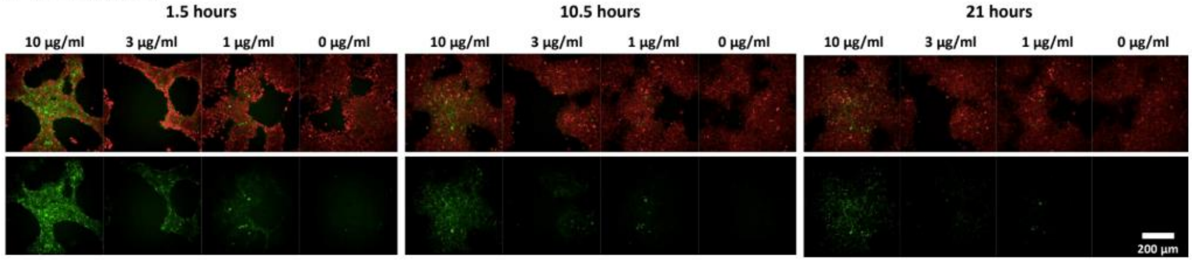

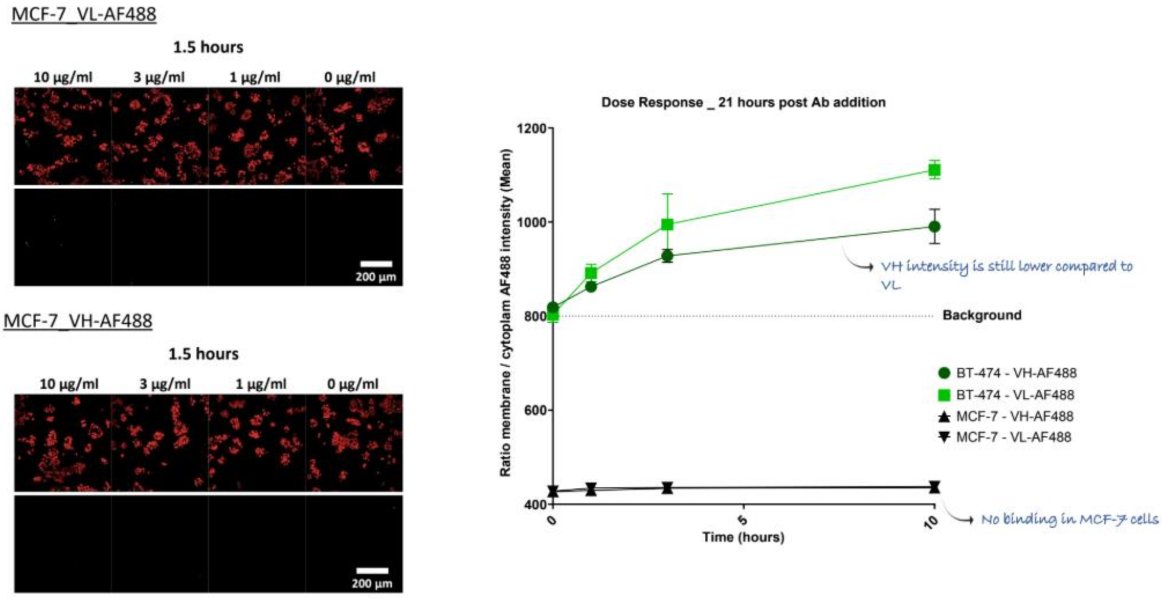

Figure S141: Overview of live-cell antibody uptake dose-response experiments with HC S378C-Azide Fluor 488 **11** (VH-AF488) and LC S168C-Azide Fluor 488 **12** (VL-AF488).

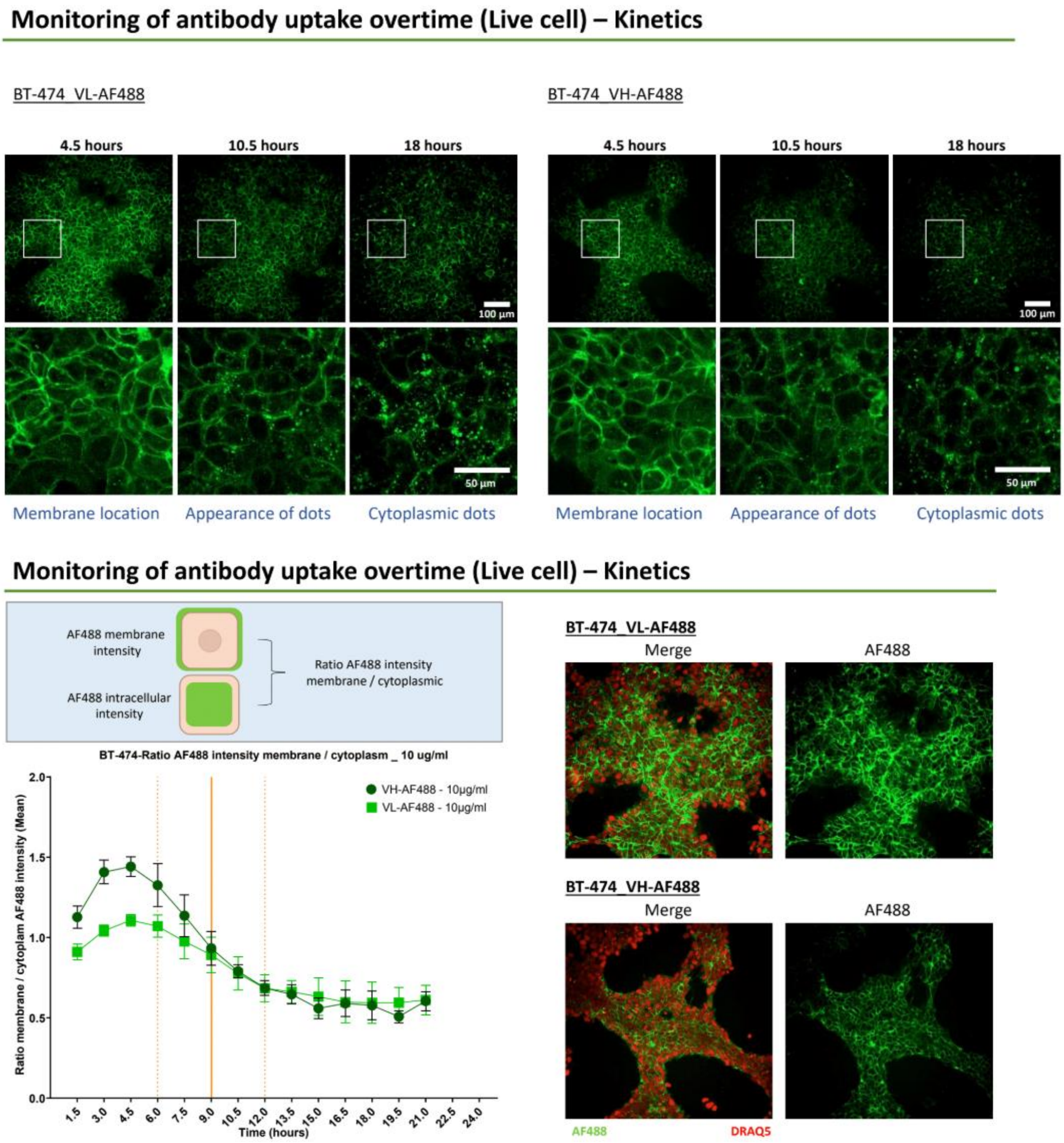

Figure S142: Overview of live-cell antibody uptake kinetic experiments with HC S378C-Azide Fluor 488 **11** (VH-AF488) and LC S168C-Azide Fluor 488 **12** (VL-AF488).

In Vitro Assays with Trifunctional Conjugates

These assays were performed with trifunctional HC S378C conjugate **38** (VH-CMM-395) and trifunctional LC S168C conjugate **34** (VL-CMM-405).

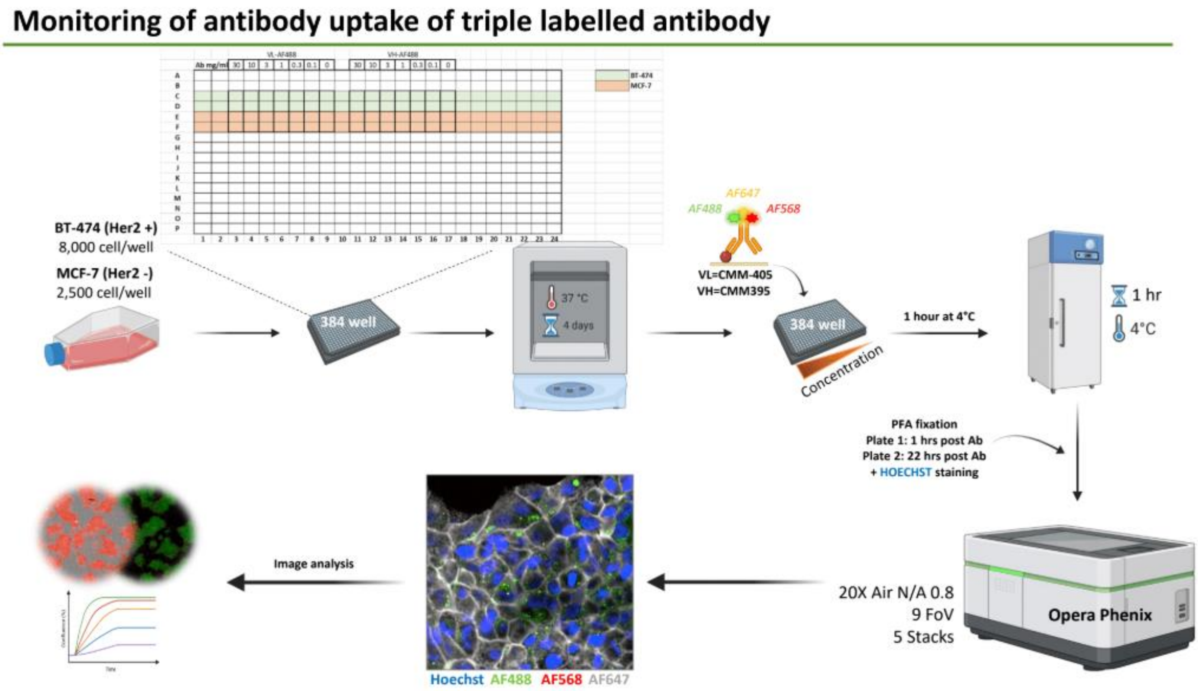

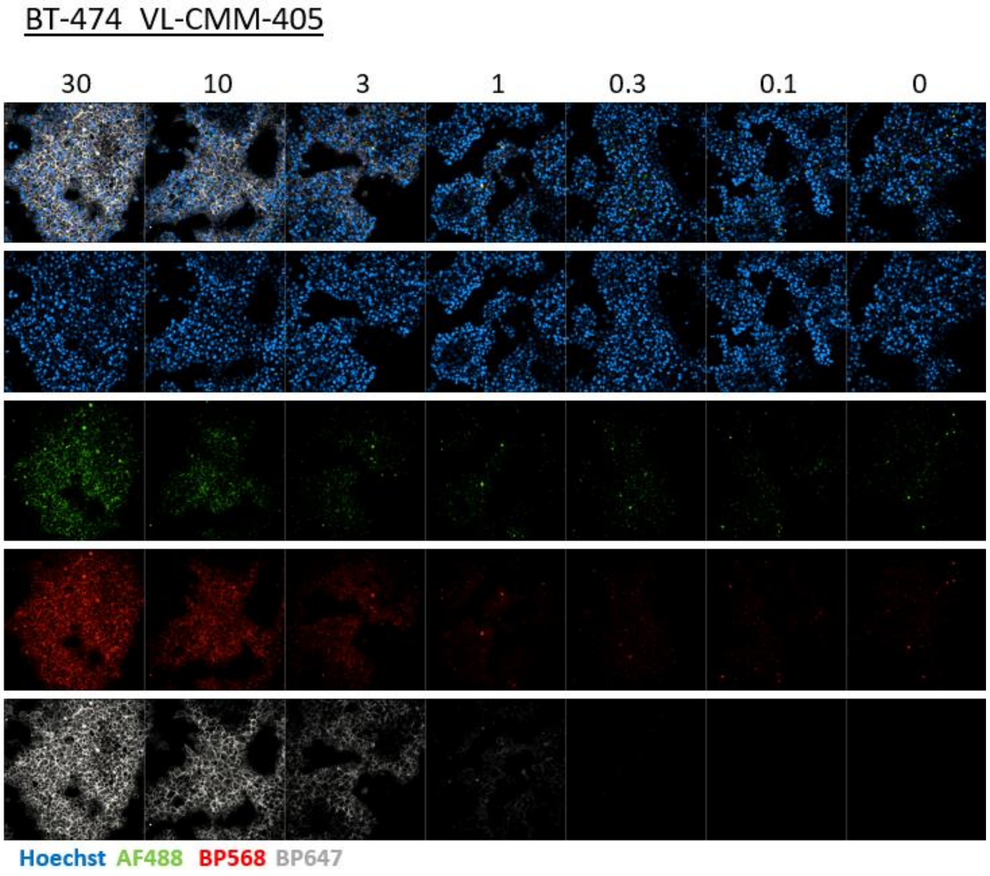

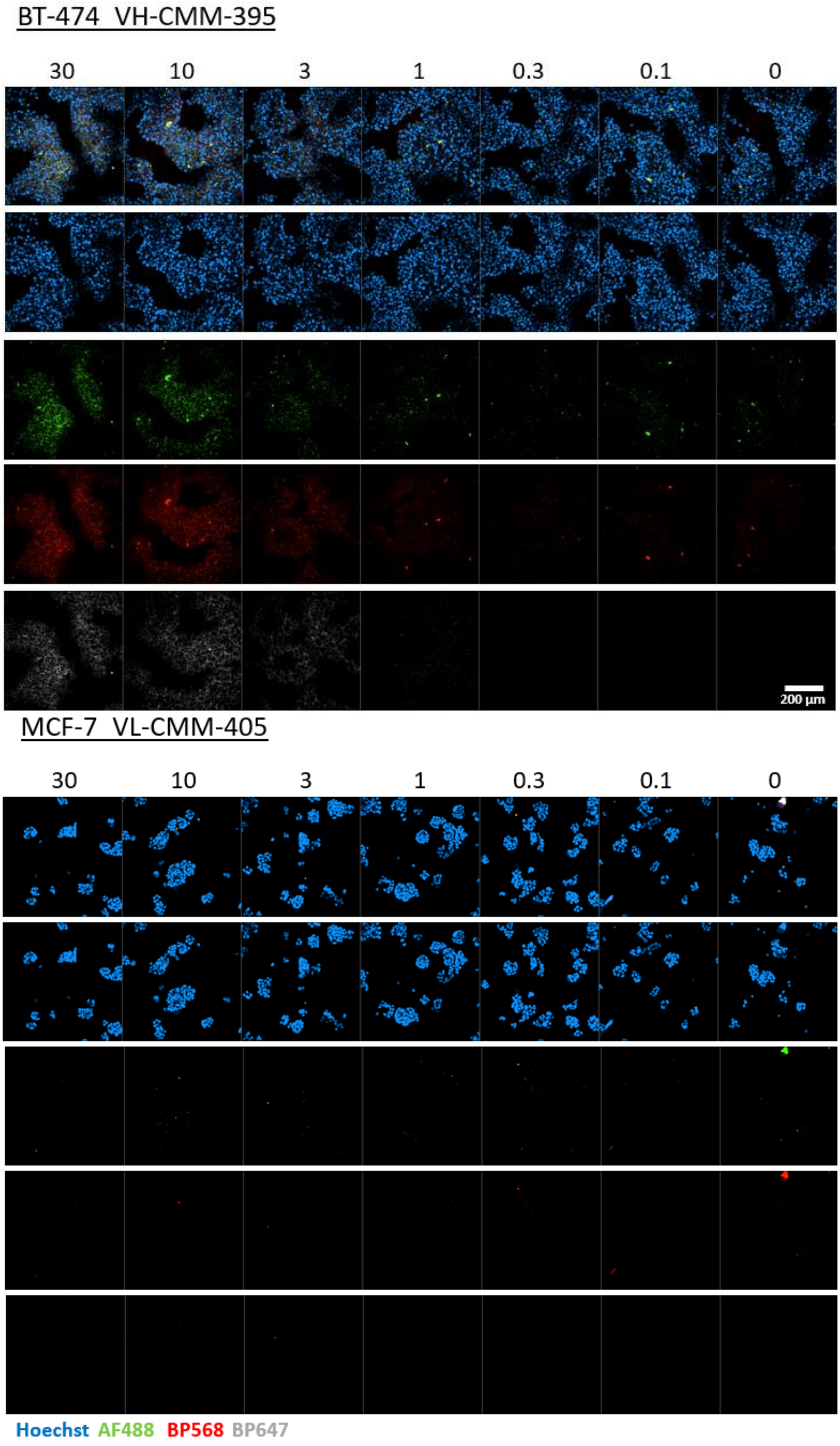
BT-474 VH-CMM-395
30
10
3
1
0.3
0.1
0
200 µm
MCF-7 VL-CMM-405
30
10
3
1
0.3
0.1
0
Hoechst AF488 BP568 BP647

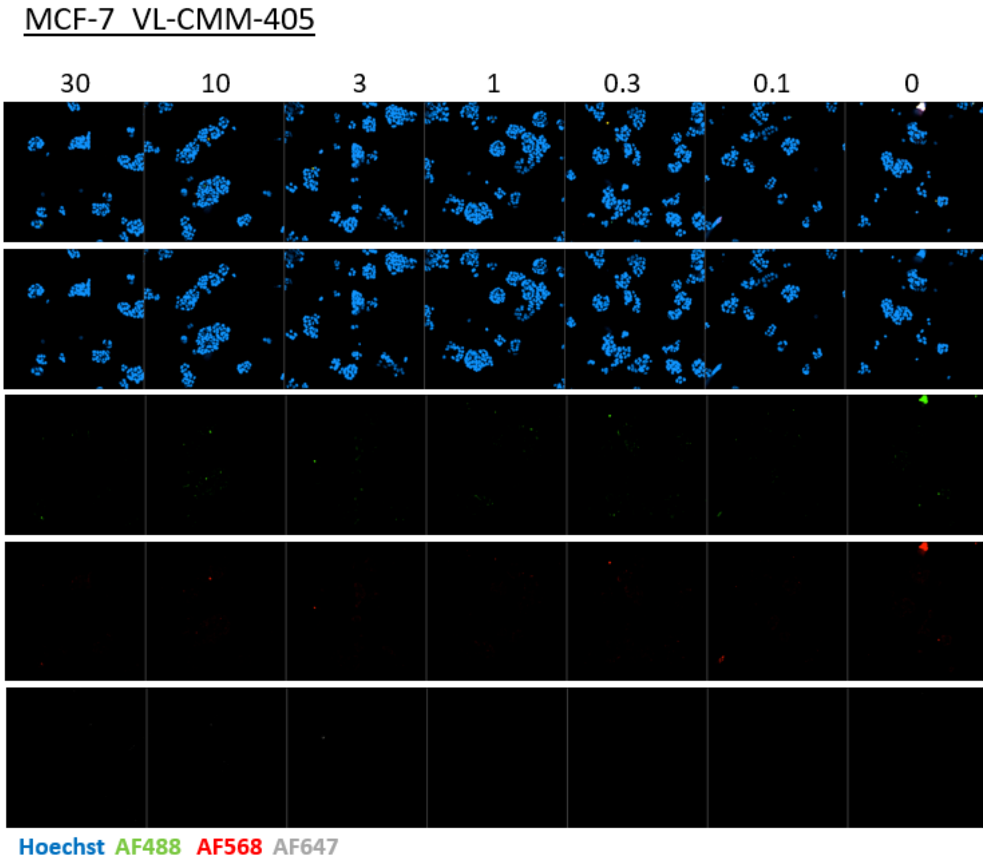

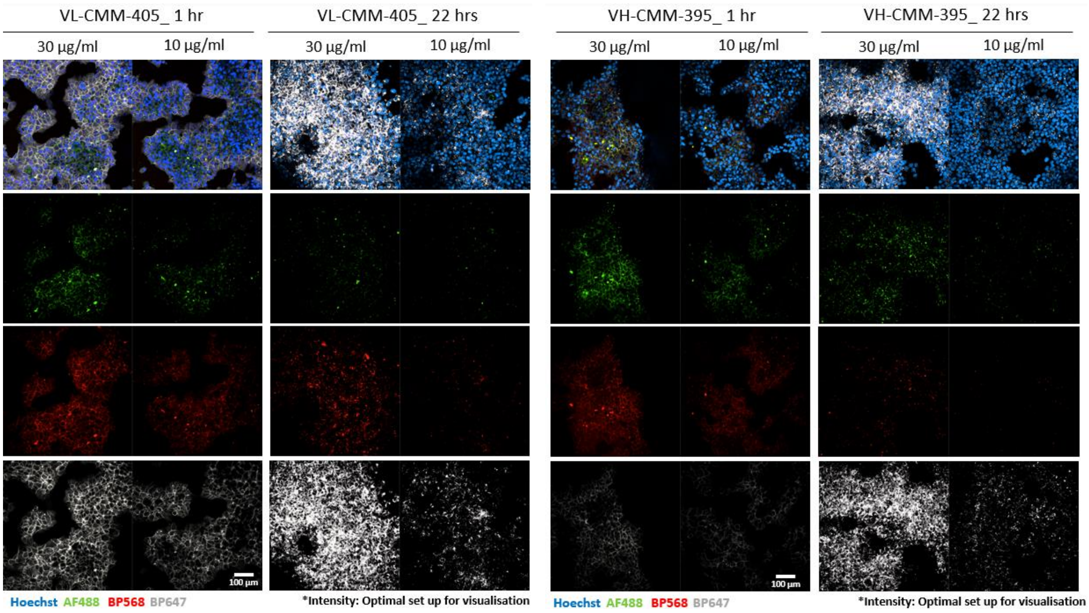

Figure S143: Overview of triply-labelled antibody uptake experiments with trifunctional HC S378C conjugate **38** (VH-CMM-395) and trifunctional LC S168C conjugate **34** (VL-CMM-405).

Triple labelled antibody – intracellular antibody processing

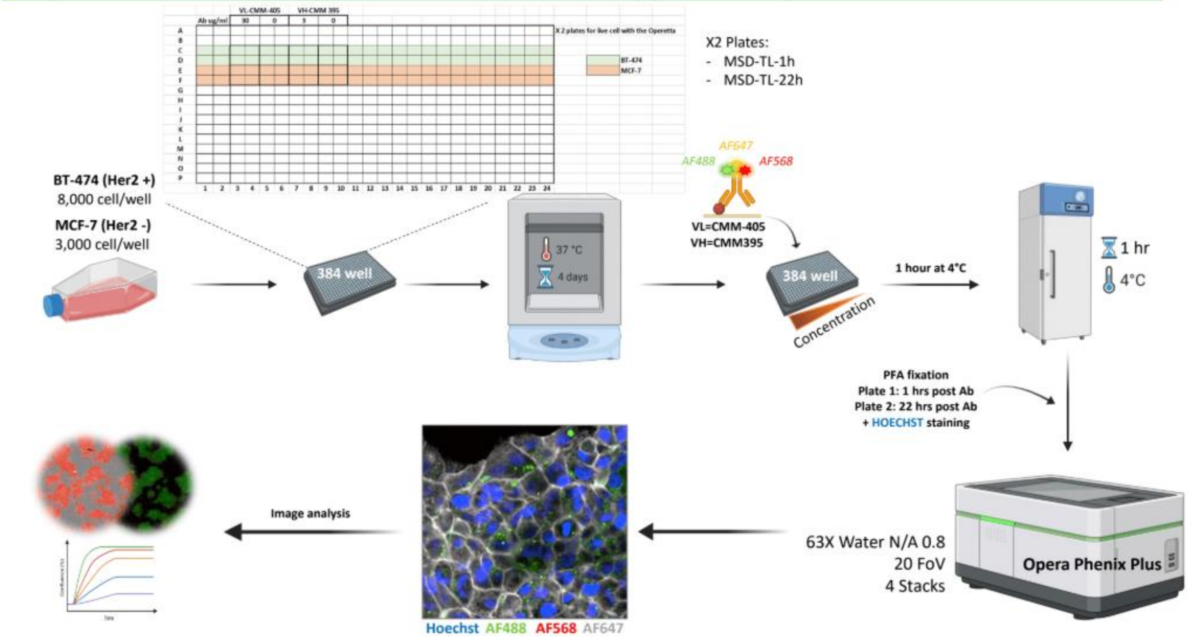

Triple labelled antibody – 63X images

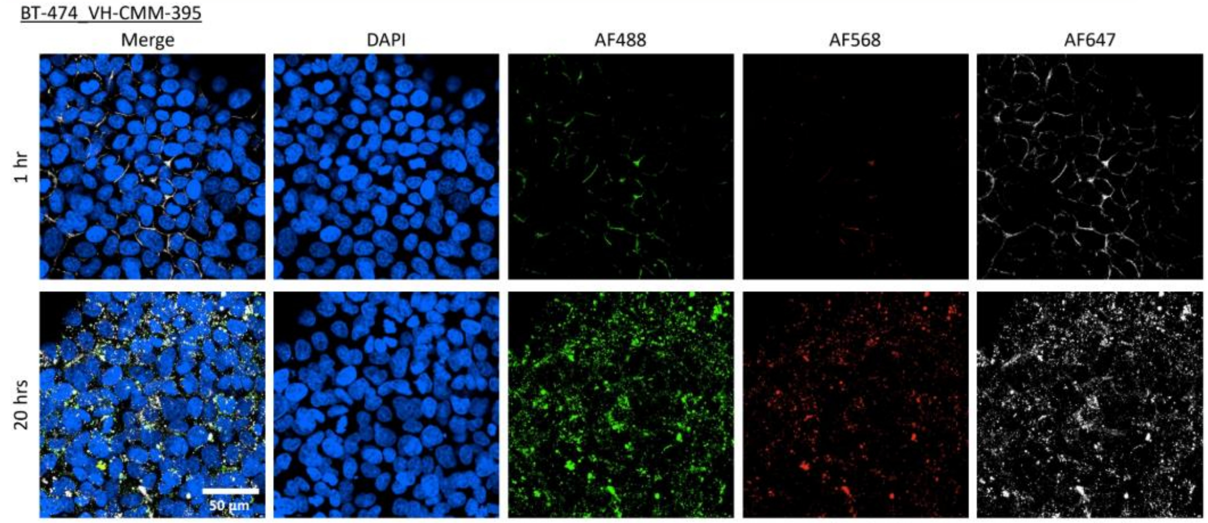

Opera Phenix Plus, 63X water, 4 stacks (4.5 µm)

Triple labelled antibody – 63X images

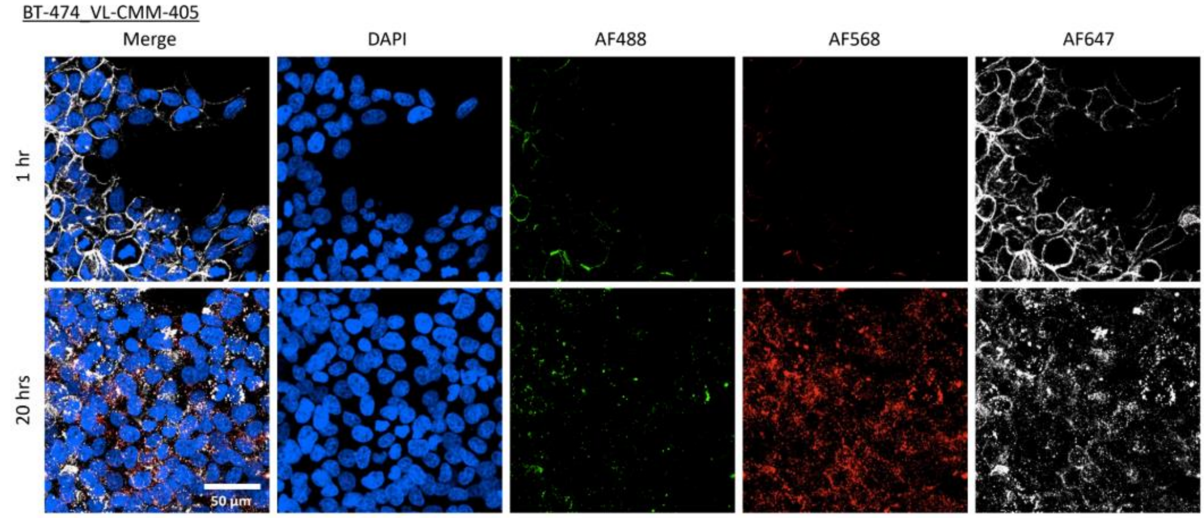

Opera Phenix Plus, 63X water, 4 stacks (4.5 µm)

Triple labelled antibody – Fluorescence intensity per cell – 20 hrs post-treatment

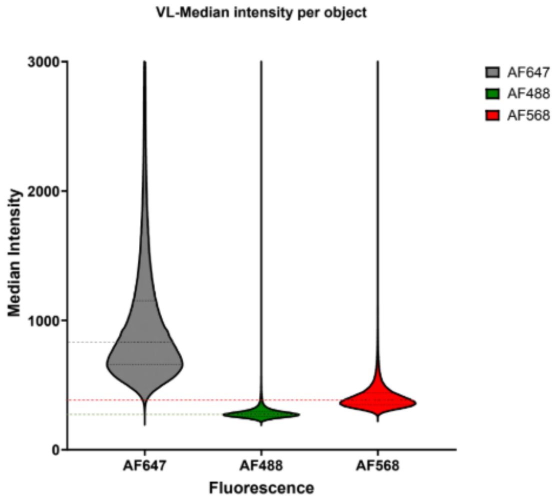

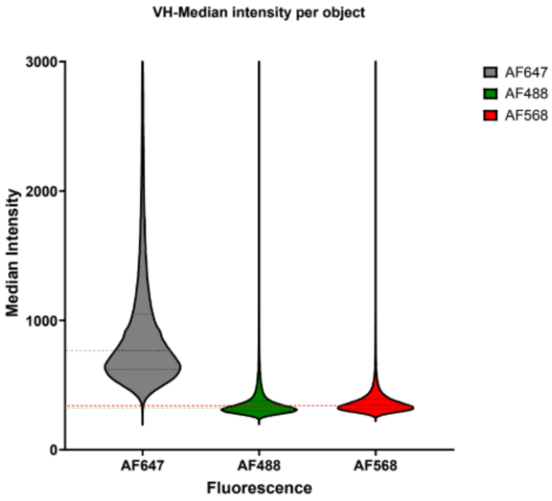

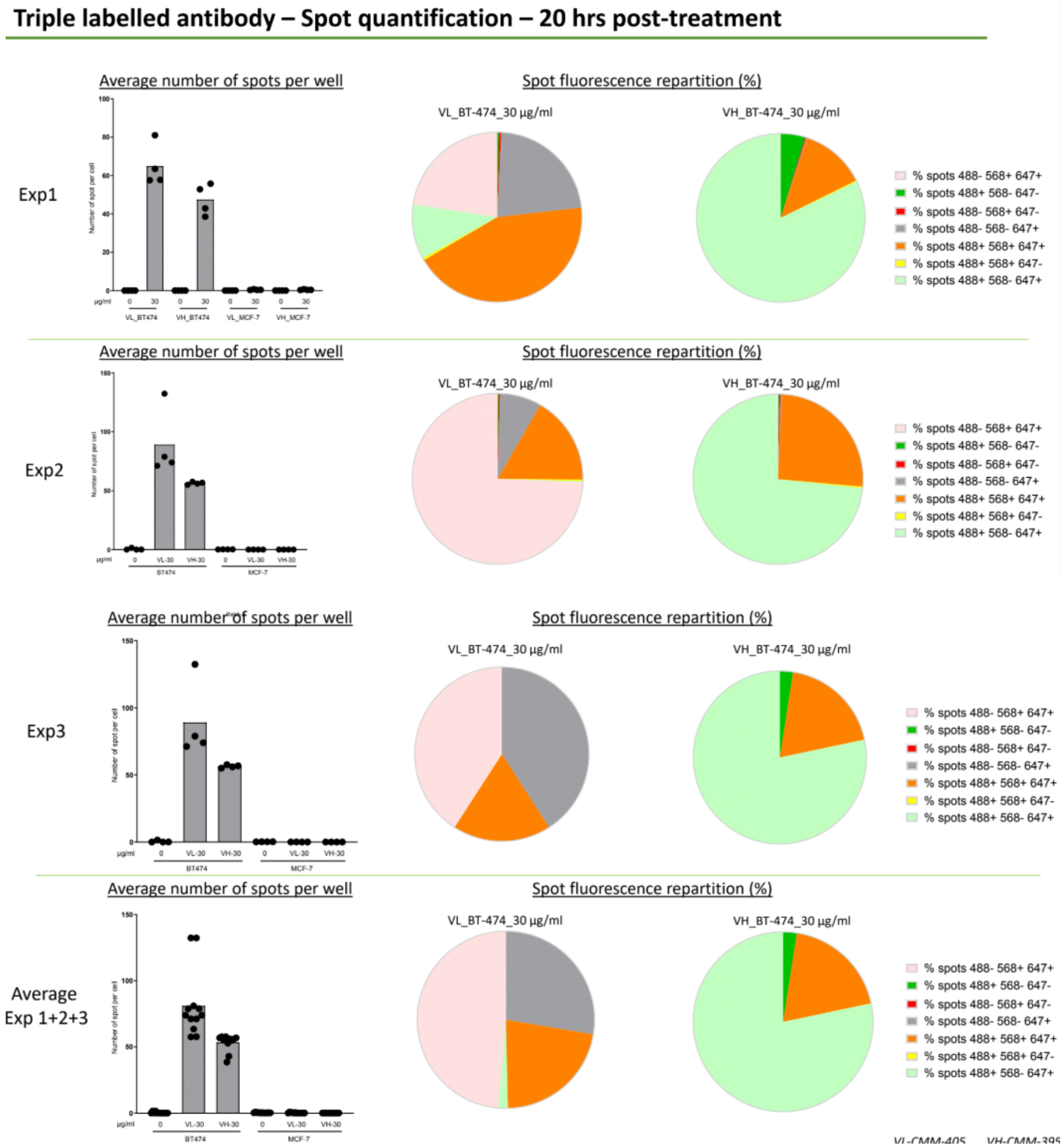

Figure S144: Overview of triply-labelled antibody uptake experiments with trifunctional HC S378C conjugate **38** (VH-CMM-395) and trifunctional LC S168C conjugate **34** (VL-CMM-405) at 63× magnification. Bottom graphs show median fluorescence intensity after 20 h showing comparatively low intensity for BP Fluor 568 and Azide-fluor 488 *vs*. BP Fluor 647 (BP Fluors are labelled as AF in some cases).

References

1 P. Moody, Mark. E. B. Smith, C. P. Ryan, V. Chudasama, J. R. Baker, J. Molloy and S. Caddick, Bromomaleimide-Linked Bioconjugates Are Cleavable in Mammalian Cells, *ChemBioChem*, 2012, **13**, 39–41.

2 C. Bahou, D. A. Richards, A. Maruani, E. A. Love, F. Javaid, S. Caddick, J. R. Baker and V. Chudasama, Highly homogeneous antibody modification through optimisation of the synthesis and conjugation of functionalised dibromopyridazinediones, *Org. Biomol. Chem.*, 2018, **16**, 1359–1366.

3 C. Bahou, P. A. Szijj, R. J. Spears, A. Wall, F. Javaid, A. Sattikar, E. A. Love, J. R. Baker and V. Chudasama, A Plug-and-Play Platform for the Formation of Trifunctional Cysteine Bioconjugates that also Offers Control over Thiol Cleavability, *Bioconjug. Chem.*, 2021, **32**, 672–679.

4 F. Thoreau, L. N. C. Rochet, J. R. Baker and V. Chudasama, Enabling the formation of native mAb, Fab′ and Fc-conjugates using a bis-disulfide bridging reagent to achieve tunable payload-to-antibody ratios (PARs), *Chem. Sci.*, 2023, **14**, 3752–3762.

5 M. T. W. Lee, A. Maruani, D. A. Richards, J. R. Baker, S. Caddick and V. Chudasama, Enabling the controlled assembly of antibody conjugates with a loading of two modules without antibody engineering, *Chem. Sci.*, 2017, **8**, 2056–2060.
